# Kingdom Chromista and its eight phyla: a new synthesis emphasising periplastid protein targeting, cytoskeletal and periplastid evolution, and ancient divergences

**DOI:** 10.1007/s00709-017-1147-3

**Published:** 2017-09-05

**Authors:** Thomas Cavalier-Smith

**Affiliations:** Department of Zoology, University of Oxford, South Parks Road, Oxford, OX1 3PS UK

**Keywords:** Chromist periplastid membrane, Chloroplast protein targeting, Chromist periplastid reticulum, Microtubular centriolar roots, Chromist evolution, Sporozoan conoid origin

## Abstract

In 1981 I established kingdom Chromista, distinguished from Plantae because of its more complex chloroplast-associated membrane topology and rigid tubular multipartite ciliary hairs. Plantae originated by converting a cyanobacterium to chloroplasts with Toc/Tic translocons; most evolved cell walls early, thereby losing phagotrophy. Chromists originated by enslaving a phagocytosed red alga, surrounding plastids by two extra membranes, placing them within the endomembrane system, necessitating novel protein import machineries. Early chromists retained phagotrophy, remaining naked and repeatedly reverted to heterotrophy by losing chloroplasts. Therefore, Chromista include secondary phagoheterotrophs (notably ciliates, many dinoflagellates, Opalozoa, Rhizaria, heliozoans) or walled osmotrophs (Pseudofungi, Labyrinthulea), formerly considered protozoa or fungi respectively, plus endoparasites (e.g. Sporozoa) and all chromophyte algae (other dinoflagellates, chromeroids, ochrophytes, haptophytes, cryptophytes). I discuss their origin, evolutionary diversification, and reasons for making chromists one kingdom despite highly divergent cytoskeletons and trophic modes, including improved explanations for periplastid/chloroplast protein targeting, derlin evolution, and ciliary/cytoskeletal diversification. I conjecture that transit-peptide-receptor-mediated ‘endocytosis’ from periplastid membranes generates periplastid vesicles that fuse with the arguably derlin-translocon-containing periplastid reticulum (putative red algal *trans*-Golgi network homologue; present in all chromophytes except dinoflagellates). I explain chromist origin from ancestral corticates and neokaryotes, reappraising tertiary symbiogenesis; a chromist cytoskeletal synapomorphy, a bypassing microtubule band dextral to both centrioles, favoured multiple axopodial origins. I revise chromist higher classification by transferring rhizarian subphylum Endomyxa from Cercozoa to Retaria; establishing retarian subphylum Ectoreta for Foraminifera plus Radiozoa, apicomonad subclasses, new dinozoan classes Myzodinea (grouping *Colpovora* gen. n., *Psammosa*), Endodinea, Sulcodinea, and subclass Karlodinia; and ranking heterokont Gyrista as phylum not superphylum.

**Electronic supplementary material:**

The online version of this article (10.1007/s00709-017-1147-3) contains supplementary material, which is available to authorized users.

## Introduction: chromist importance and aims of this paper

Chromista is one of five eukaryotic kingdoms recognised in a comprehensive seven-kingdom classification of life (Ruggiero et al. 2015). As here critically reassessed, Chromista comprise eight distinctive phyla, not just three as in the first substantial systematic treatment 30 years ago (Cavalier-Smith 1986)—5 years after Chromista was established (Cavalier-Smith 1981a). Chromista have turned out to include the vast majority of marine algae and of heterotrophic protists, whether marine or in soil or freshwater, and some of the most serious human disease agents such as malaria parasites and agricultural pathogens like potato blight and sugar beet rhizomania disease, making chromists immensely important for ocean ecology, soil biology, climate stability, agriculture, and medicine, as well as for fundamental understanding of eukaryote evolution and biodiversity. They have a greater range in radically different body plans and lifestyles than the entire plant kingdom and more phyla than kingdoms Fungi or Protozoa. Only animals and bacteria have more phyla than chromists, but even they cannot match chromists in their remarkable range of contrasting adaptive zones—from giant brown algal kelps longer than a blue whale to ciliates like *Paramecium*, dinoflagellates that power coral reefs or kill shellfish, the most abundant predators in soil (sarcomonad Cercozoa), parasites like *Toxoplasma* whose cysts are allegedly lodged in a third of human brains and *Plasmodium* that causes malaria, diatoms whose silica frustules were once essential for making dynamite or polishing astronomical telescope mirrors, and foraminifera or haptophyte plankton like *Emiliania* that can be seen from outer space and made the white cliffs of Dover with their calcareous scales and are probably the most speciose photosynthetic oceanic flagellates and exude volatile chemicals that affect cloud formation and global energy balance.

There are probably in excess of 150,000 free-living chromist species, the most speciose being diatoms (estimated at ~ 100,000 species) and foraminifera (~ 10,000 living and ~ 40,000 fossil species), many thousands undescribed. Parasitic chromists could be ten times that, as chromist Sporozoa probably infect every insect and every other animal species, and other chromists to infect numerous plants, and even some protozoa or other chromists. Already named chromist species (over 180,000; Corliss 2000) may be only the tip of the iceberg. There are probably far more species of chromist than of plants or protozoa, conceivably even more than fungi, and certainly more individual chromists than plants and animals combined. Possibly, only viruses and bacteria exceed them in numbers. What are their distinctive features? Why were they established as a kingdom separate from Plantae, Fungi, and Protozoa, where they were once misclassified?

This paper answers both questions in the next four sections and then provides a new synthesis aimed to better establish chromist evolutionary unity, clarify their origin, and outline how their major lineages evolved from a shared ancestral body plan. Two major innovations are a radically revised interpretation of chromist chloroplast membrane evolution and protein targeting, including correcting widespread misconceptions about the character and very limited evolutionary role of tertiary symbiogenesis, and thorough reevaluation of centriolar root evolution and evolutionary diversification of ciliary transition zones across the kingdom, relating both to innovations in cell motility and feeding and to phylogenetic evidence from sequence trees. A new derlin sequence phylogeny shows that eukaryotes ancestrally had two radically different paralogues and chromist nuclei and nucleomorphs (relict enslaved red algal nuclei) kept different red algal derlin paralogues for periplastid protein targeting. My discussions on cytoskeletal and ciliary evolution, though rather detailed in places, are set in the broad context of overall eukaryote cytoskeletal evolution and therefore include some wider implications for eukaryote cell evolution and cell biology in general. For convenient reference in a complex field, I summarise an improved higher-level classification of chromists; by removing a few past confusions, its revisions enable new cell evolutionary insights. As the paper is long, I highlight 15 major novel conclusions at the end.

## Distinction of Chromista from Plantae

In 1981 kingdom Plantae of Haeckel (1866)—equivalent to kingdom Vegetabilia or Regnum Vegetabile of Linnaeus (1767)—was restricted to all eukaryotes having plastids located in the cytosol that originated directly from an internally enslaved cyanobacterium from which they inherited an envelope of only two membranes (Cavalier-Smith 1981a). Plantae comprise subkingdoms Viridiplantae (green plants), using chlorophyll *b* as an accessory photosynthetic pigment, and Biliphyta (red algae and glaucophytes) that retained phycobilisomes from the ancestral cyanobacterial endosymbiont instead (Cavalier-Smith 1982, 1998). The key steps in the symbiogenetic origin of chloroplasts from cyanobacteria were evolution of membrane transporters for exporting photosynthetic products and machinery for importing nuclear-coded proteins (Cavalier-Smith 1982, 2000a, 2013a). Later, multiple gene transfers from the enslaved cyanobacterium into the nucleus and losses of the bacterial cell wall were secondary—peptidoglycan being retained in chloroplast envelopes of glaucophytes and basal streptophyte Viridiplantae (lost three times in plant evolution: in red algae, thus absent also in chromists; in Chlorophyta; and in the fern/seed plant clade) (Hirano et al. 2016). As predicted (Cavalier-Smith 1982), chloroplasts of all Plantae share an evolutionarily homologous protein import machinery (Toc for import across their outer membrane (OM) which evolved from the cyanobacterial OM by replacing its outer leaflet lipopolysaccharide by host phosphatidylcholine (PC) and Tic for traversing their inner membrane; Bölter and Soll 2016). This shared machinery (modified from cyanobacterial protein export machinery) and the fact that chloroplast DNA multigene trees group all chloroplasts as a single subclade of cyanobacteria (Ochoa de Alda et al. 2014) led to general acceptance that chloroplasts originated only once, and Plantae as redefined in 1981 are monophyletic.

Chromophyte algae (those using chlorophyll *c* not *b* as an accessory pigment) were long recognised as rather distinct from green plants (Chadefaud 1950; Christensen 1962, 1989). Only after Manton and Leedale (1961a, b) discovered by electron microscopy that haptophyte chloroplasts share a bounding membrane with the nucleus, and Gibbs (1962) recognised that most chromophytes have two extra membranes around their chloroplasts, did it gradually become clear how radically distinct they are. For a long time, Gibbs’ (1962) initial misinterpretation of both extra membranes as endoplasmic reticulum (ER) was perpetuated by the term chloroplast ER (Bouck 1965). But after Greenwood (1974) discovered the cryptophyte nucleomorph (NM) between these extra membranes and the chloroplast envelope, suggesting it to be a vestigial nucleus of a permanently enslaved algal symbiont, chromophyte membrane topology became better understood. Whatley et al. (1979) explained that only the outermost membrane was continuous with the rough ER forming the nuclear envelope outer membrane, whereas the smooth membrane lying between it and the double chloroplast envelope was topologically distinct and probably the relict plasma membrane of a former eukaryotic endosymbiont. I accepted that but argued, contrary to Whatley et al. (1979), that one enslavement of a eukaryotic algal symbiont made all chromists—both cryptophytes and those without NMs but otherwise identical membrane topology (Cavalier-Smith 1982). NM DNA (Ludwig and Gibbs 1987) and division (Morrall and Greenwood 1982) confirmed its nuclear nature, and Cavalier-Smith (1989) created the name ‘periplastid membrane’ (PPM) for the former algal plasma membrane, stressing that chromist plastids plus surrounding PPMs are inside the rough ER not in the cytosol like chloroplasts of Plantae.

**Fig. 1:**
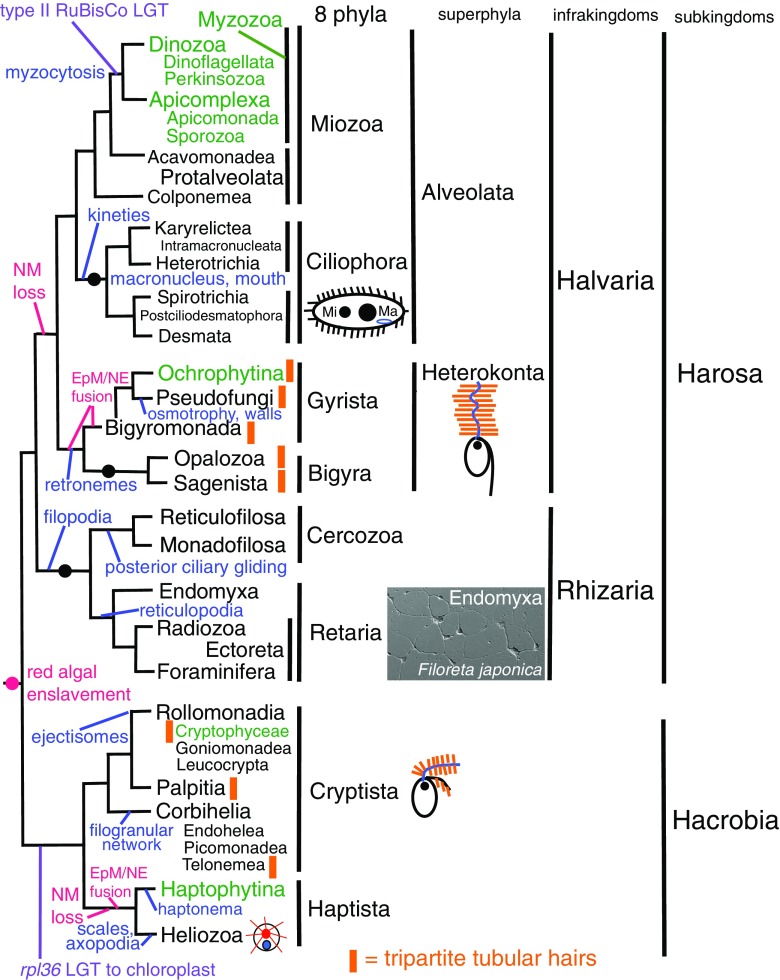
Relationships between major chromist groups inferred from sequence trees mostly using many scores of genes. For taxa ranked as subphyla or lower, clades still possessing the ancestral chromist plastid of red algal origin are shown in *green*, and purely heterotrophic ones without evidence for plastids are shown in *black*. *Black discs* mark inferred extremely early plastid losses. Too little is known about protalveolates, bigyromonads, and heterotrophic Hacrobia to know whether they retain DNA-free colourless plastids like most heterotrophic Dinozoa or not. Paraphyletic bigyromonads (mostly still uncultured) are not broken down into constituent clades. Major harosan innovations discussed here are shown in *blue*; for the detailed treatment of hacrobian cell diversification, see Cavalier-Smith et al. (2015a). The best nuclear, plastid, and mitochondrial trees all show this topology (see text); though topologically accurate, this diagram is temporally extremely misleading: branch lengths do not represent time. Virtually, all bifurcations shown occurred in the Precambrian >600 My ago; the basal stems occupied only a tiny fraction of the ~ 750 My history of Chromista (Cavalier-Smith et al. 2015a; Cavalier-Smith, in prep.). Two lateral gene transfers (*LGTs*) from bacteria (*purple*) prove that ancestral Myzozoa and Hacrobia each had plastids and effectively eliminate the possibility that ochrophytes could have arisen from either of them by a late tertiary symbiogenesis (lateral plastid transfer). The LGT into the ancestral hacrobian plastid is especially important as showing that plastids were present immediately after the very first chromist bifurcation. Ancestral chromists were haploid biciliates with younger anterior cilium (*blue*) and older posterior cilium (*black*, typically with different structures and beat patterns produced by ciliary transformation in its second cell cycle). Ciliates (Ciliophora) multiplied cilia in kineties and evolved separate somatic multiploid macronuclei (*Ma*) and diploid germline micronuclei (*Mi*) and complex mouths to make giant multiciliate cells, whereas some chromists lost cilia altogether, exemplified by the micrograph of an endomyxan rhizarian *Filoreta* (Bass et al. 2009a, b) that evolved a remarkable net-like multinucleate body. Nucleomorphs (*NMs*) were lost twice independently in photosynthetic lineages (phycobilins lost simultaneously) and additionally in all heterotrophs but *Chilomonas*

Kingdom Chromista was established to include all chromophyte algae whose chloroplasts are separated from the cytosol by four topologically distinct membranes as well as all heterotrophic protists that descended secondarily from them by losing plastids (Cavalier-Smith 1981a). It had long been accepted that Oomycetes and Hyphochytridiomycetes (collectively subphylum Pseudofungi; Cavalier-Smith 1986) were more closely related to chromophyte algae than to kingdom Fungi because like the major chromophyte subphylum Ochrophytina (e.g. brown algae, xanthophytes, diatoms, chrysophytes; Cavalier-Smith 1986), they exhibit a heterokont ciliary pattern, but they were formally grouped together only when kingdom Chromista was established (Cavalier-Smith 1981a). Heterokont chromists typically have an anterior cilium bearing one or two rows of rigid tubular tripartite ciliary hairs that reverse its propulsive thrust (so I called them ‘retronemes’; Cavalier-Smith 1986). Thrust reversal ensures when this cilium undulates from base to tip it projects forward during swimming, not backward as does the similarly undulating cilium of opisthokonts (Fungi, animals, Choanozoa; Cavalier-Smith 1987a). Heterokonta was formally established as a taxon by grouping not only Oomycetes and hyphochytrids with heterokont chromophytes but also Labyrinthulea—whose zoospores have the same retroneme-bearing heterokont cilia but were misclassified as fungi, as well as Bicoecida, phagotrophic heterokont flagellates long misclassified as Protozoa (Cavalier-Smith 1981a). Phylum Heterokonta was extended to include all protists with homologous tripartite ciliary hairs restricted to their anterior cilium (Fig. 1) when I argued that losing them would be functionally disruptive by reversing swimming direction and evolutionarily rare, making them an excellent phylogenetic marker easily recognised by electron microscopy (Cavalier-Smith 1986). Phylum Cryptista (originally including only cryptomonads, i.e. photosynthetic cryptophytes with tubular hairs believed to be related to retronemes on both cilia plus phagoheterotrophic goniomonads with different hairs; Cavalier-Smith 1989) were grouped with heterokonts plus the almost exclusively photosynthetic haptophytes (postulated to have lost ciliary hairs) as Chromista.

Therefore, Chromista was originally defined as all eukaryotes that have chlorophyll *c*-containing plastids inside the ER and an additional smooth membrane (PPM) between it and the chloroplast envelope and/or rigid tubular hairs plus all eukaryotes that can be shown to have lost one or both of these characters (Cavalier-Smith 1981a, 1986). The PPM was held to have originated from the plasma membrane of a eukaryotic algal symbiont permanently enslaved to provide chromist plastids (Whatley et al. 1979; Cavalier-Smith 1982). Chromists with that plastid type and peripheral membrane topology were later called euchromists after 18S ribosomal DNA (rDNA) trees hinted that some algae with very different complex membrane topology were phylogenetically chromists (Cavalier-Smith 1993a), a possibility earlier thought unlikely (Cavalier-Smith 1986). Initially, I wrongly assumed that all chromist tubular ciliary hairs reverse ciliary thrust (as they do in heterokonts only) and therefore overestimated the difficulty of non-heterokont chromists losing them; also, I conservatively kept assumptions of a loss of plastids or tubular ciliary hairs to a strict minimum, so for a longish period underestimated the frequency of plastid or hair loss and number of misclassified protozoan groups that were really ancestrally chromists. Pure protozoan-like heterotrophs like bicoecids that were obviously heterokont chromists from the outset (Cavalier-Smith 1981a) were but the tip of the iceberg of misclassified secondarily heterotrophic chromist phagotrophs. For example, Cryptista now include not only plastid-bearing class Cryptophyceae but also six related heterotrophic classes, two with non-thrust reversing tripartite tubular hairs, implying that ancestral cryptists had such hairs but four classes independently lost them; centrohelid heliozoa that lost cilia and photosynthesis belong to Haptista (Cavalier-Smith et al. 2015a).

Since my last major survey of both algal and heterotrophic chromist evolution (Cavalier-Smith 2004a), the taxonomic scope of Chromista greatly increased by adding three major groups previously considered protozoa (Cavalier-Smith 2010), thereby accepting that plastids and tubular ciliary hairs were lost more often during early chromist diversification than originally supposed (Fig. 1). Some who still resist the idea of Chromista do so because they fail to appreciate that such early losses are far easier evolutionarily than multiple independent acquisitions of fundamentally similar chloroplasts. Others do so because they mistakenly suppose that sequence trees contradict chromist monophyly. Both viewpoints stem from superficially attractive fallacies; their deep flaws are explained in great detail elsewhere (Cavalier-Smith et al. 2015a). Here, I focus instead on explaining the positive evidence from molecular cell biology and ultrastructure for the evolutionary unity of chromists and the cell evolutionary processes involved in diversification of their major groups.

## Distinction of Chromista from Protozoa

**Fig. 2:**
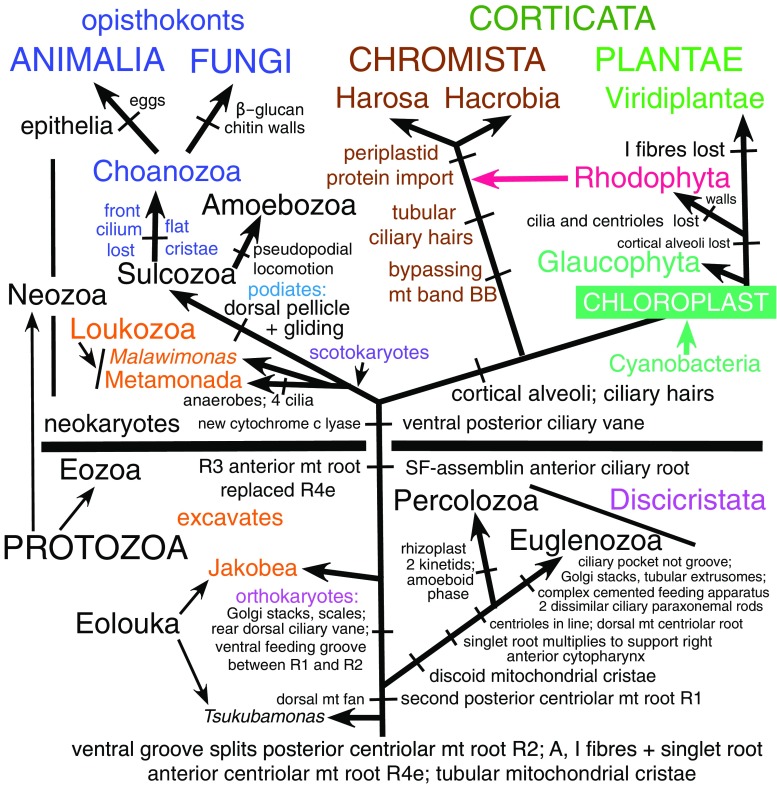
Schematic eukaryote phylogeny fully consistent with 187-protein trees (Cavalier-Smith et al. 2015a), rooted as in a 72-protein archaebacteria-rooted ribosomal tree (Raymann et al.’s 2015 Fig. 1), showing relations amongst the five eukaryote kingdoms (*upper case*). Kingdom Chromista comprising subkingdoms Harosa (Heterokonta, Alveolata, and infrakingdom Rhizaria) and Hacrobia (phyla Haptista and Cryptista) is most closely related to Plantae that consists of three major groups with distinct chloroplast pigments and ultrastructure: Glaucophyta and Rhodophyta (both with phycobilisomes, unstacked thylakoids, and cytosolic starch) and Viridiplantae with chlorophyll *b* instead of phycobilisomes, stacked thylakoids, and plastid starch. Plant chloroplasts evolved by a single primary enslavement of a cyanobacterium with both phycobilisomes and chlorophyll *a* (*green arrow*) and chromist plastids evolved by a single secondary symbiogenetic enslavement of a red alga (*red arrow*). All seven phyla of basal kingdom Protozoa are shown, subdivided into two subkingdoms, Neozoa and Eozoa. The four neozoan phyla (Choanozoa, Amoebozoa, Sulcozoa, Loukozoa) are more closely related to animals and Fungi than to superkingdom Corticata (Plantae plus Chromista) or to Eozoa: collectively animals, fungi, and Neozoa are an entirely non-photosynthetic clade (*scotokaryotes*: Cavalier-Smith et al. 2015a). Scotokaryotes are sisters of corticates if the tree is correctly rooted, forming joint clade *neokaryotes*. Eozoa being a clade sister to neokaryotes (He et al. 2014) or within neokaryotes (Derelle et al. 2015) rather than ancestral as shown is cell biologically improbable. Phyla Eolouka and Percolozoa have the most primitive mitochondrial genomes (Kamikawa et al. 2014) and retain ancestral bacterial cytochrome c biogenesis unlike derived neokaryotes and Euglenozoa (Cavalier-Smith 2010). Irrespective of the precise position of the eukaryote root, excavate protozoa (orange; defined as ancestrally biciliates having posterior ciliary vane and ventral feeding groove with an homologous microtubular/fibrillar cytoskeleton of three distinctive posterior centriolar roots (Simpson 2003), but no cortical alveoli; contrary to past usages, excavates here exclude the cytoskeletally radically different discicristates as well as *Tsukubomonas* with the simplest cytoskeleton of all biciliate Eozoa) are *paraphyletic ancestors* of Sulcozoa (which arose by evolving a dorsal pellicle and posterior ciliary gliding: Cavalier-Smith 2013b; Cavalier-Smith et al. 2014) and Corticata, which arose by evolving cortical alveoli and simple ciliary hairs whilst originally retaining all neoloukan cytoskeletal microtubular roots—all evident in the harosan alveolate subphylum Protalveolata whose orders Colponemida and Acavomonadida still feed by directing prey into the groove by a vaned posterior cilium exactly as in the neoloukan excavate *Malawimonas* (phylum Neolouka here includes secondarily anaerobic subphylum Metamonada: Cavalier-Smith 2013b; Cavalier-Smith et al. 2015a). As the text explains, the ancestors of chromists almost certainly used this groove-based feeding before they evolved BB and tubular ciliary hairs and enslaved red algal plastids. *Orthokaryotes* (named here for the putative clade comprising neokaryotes and cytoskeletally distinct Jakobea, i.e. excavates sensu stricto plus all their descendants) ancestrally had two *orthogonal* centrioles (parallel in discicristates except *Pharyngomonas*), *orthodox* stacked Golgi (arguably ancestrally unstacked in *Tsukubamonas* and Percolozoa), two opposite posterior ciliary roots (*Tsukubamonas* only one, its singlet root inherently part of R2), and always orthodox nuclear gene transcriptional control that evolved in the ancestral eukaryote (lost by Euglenozoa)

Advanced thinkers recognised that some chromists are neither plants nor animals ever since Owen (1858) pioneered the idea of a third kingdom for unicellular organisms by establishing kingdom Protozoa that, as well as heterotrophs, originally included chromistan diatoms as well as other unicellular algae (and even bacteria) and thus was more like kingdoms Protoctista of Hogg (1861) or Protista of Haeckel (1866) than the much more restricted predominantly heterotrophic kingdom Protozoa used in recent classifications (Cavalier-Smith 2010; Ruggiero et al. 2015). I shall not discuss the complex (often misleadingly oversimplified) history of classification of organisms now separated in Chromista and Protozoa, which between 1956 and 1981 in four- and five-kingdom systems (which then began to replace Linnaeus’ classical two-kingdom system), were often lumped together as a single kingdom Protista or Protoctista (Copeland 1956; Margulis 1974; Margulis and Schwartz 1982), whose composition and classification changed time and again, and refer interested readers to Ragan (1997). Chromists—ancestrally eukaryote-eukaryote chimaeras that arose by symbiotic enslavement of a eukaryote (red alga), thus mostly with plastids—and Protozoa that arose ancestrally and monophyletically by the origin of the eukaryote cell from a prokaryote and its enslavement of symbiotic purple bacteria to make mitochondria (Cavalier-Smith 2014b) differ essentially in membrane topology and protein targeting (which played key but different roles in their respective origins) and in their contrasting phylogenetic positions. Even though one advanced protozoan class (Euglenophyceae) later acquired a green algal plastid by an entirely independent symbiogenetic enslavement (with radically different protein-targeting consequences) from the red algal enslavement that formed chromists (Cavalier-Smith 2003a, 2013a), Protozoa ancestrally were not eukaryote-eukaryote chimaeras, unlike chromists. Also, unlike chromists, Protozoa are not a clade but the basal or stem eukaryotic kingdom from which the four derived kingdoms (probably all clades) arose by evolving radically new, non-protozoan properties (Fig. 2).

## Conceptual importance of protein targeting for chromist unity and evolution

Understanding chromist origin was transformed by discovery of a novel mechanism of periplastid protein translocation (Sommer et al. 2007); however, I argue here that the standard interpretation of this discovery is incomplete and partially incorrect. Instead, I propose a detailed new one—effectively a radical synthesis of the best parts of the ideas of Gibbs (1979) and of Maier’s pioneering group (e.g. Maier et al. 2015; Sommer et al. 2007) with my own (Cavalier-Smith 1999, 2003a, 2013a), discarding errors in assumptions we all made. Equally transformative for chromist biology were conceptual innovations (Cavalier-Smith 1999), discoveries of the sporozoan apicoplast (McFadden et al. 1996; reviewed by McFadden 2011) and of shared lateral transfer of gene *rpl36* from a bacterium to hacrobian chloroplasts (Rice and Palmer 2006), and photosynthetic Apicomplexa (Moore et al. 2008), as well as multiprotein sequence trees providing robuster eukaryote phylogeny (Burki et al. 2007, 2008, 2009), stimulating better demarcation between the ancestral eukaryotic kingdom Protozoa and derived Chromista and new subkingdoms (Cavalier-Smith 2010), and confirming monophyly of corticate eukaryotes (the clade comprising Plantae and Chromista; Cavalier-Smith 2003b).

Membranes and cytoskeleton jointly define Chromista. Half the present paper dedicated to Peter Sitte’s memory discusses protein targeting into and across chromist membranes, and evolutionary continuity of membranes during chromist symbiogenesis, in relation to the important conceptual problem of how novel kinds of genetic membranes arise during evolution (Cavalier-Smith 2000a, 2004a, b). Then follows the most detailed treatment yet of the chromist cytoskeleton which exhibits more unity and contrasts with other kingdoms than previously realised. This also yields new insights into the radical cytoskeletal and membrane reorganisation during the origin of the first corticates—the common ancestors of plant and chromist cells.

I first emphasised the central importance of understanding the origin of novel protein-targeting machinery that creates novel genetic membranes (Cavalier-Smith 1995a) in relation to chloroplast and mitochondrial origins (Cavalier-Smith 1980), elaborating it when first explaining why the much more complex yet uniform membrane topology of euchromists (those with plastids inside the rough ER lumen; Cavalier-Smith 1993a) must have resulted from a single symbiogenetic event (Cavalier-Smith 1982). I returned to this problem at intervals, fleshing out details and correcting some early misconceptions (Cavalier-Smith 1986, 1995a, 1999, 2000a, b, 2004a, 2013a), but we still understand the complex molecular cell biology of chromists far too scrappily for the present synthesis to end the story. The concept of membrane heredity, a mode of inheritance in some respects independent of DNA heredity and existing cooperatively with it since cells began (Cavalier-Smith 1995a, 2000a, 2001, 2004a), provides a unifying conceptual approach to understanding the evolution of membranes and protein insertion into and across them. It highlights the fundamental difference in cell organisation between Plantae and Chromista, which is much more radical than that between animals and fungi (essentially the origin of fungal chitin/β-glucan walls causing phagotrophy loss). As emphasised earlier, ‘The numbers of different genetic membranes associated with algal chloroplasts cannot be understood in simple functional or adaptive terms, but are self-perpetuated relics of the historical accidents that led to their formation’ (Cavalier-Smith 1995a, p. 107).

I first met Peter Sitte at a conference where he spoke on membrane continuity and cell compartmentation during symbiogenesis (Sitte 1983) and I first unequivocally advocated a six-kingdom classification with Protozoa and Chromista conceptually distinct kingdoms (Cavalier-Smith 1983a) and first argued that the OM of mitochondria evolved from the OM of an enslaved α-proteobacterium (Cavalier-Smith 1983b, then a new idea in membrane heredity), and introns evolved by insertion of transposable elements (Cavalier-Smith 1983c). All three ideas were then heterodox—the latter two now universally accepted, the first still passionately debated, accepted by some but not all. The initially equally heterodox idea of a single secondary red algal enslavement, however, is now universally accepted for all chromophytes (Gould et al. 2015) 35 years after a single ancestral enslavement was argued for euchromists only (Cavalier-Smith 1982) and two decades since its extension to all chromophytes (Cavalier-Smith 1995a, as a possibility; Cavalier-Smith 1999, as a detailed explanatory theory when we got the first dinoflagellate chloroplast DNA sequences; Zhang et al. 1999). That this took place in the last common ancestor of Chromista and that was a photophagotroph not a heterotroph still arouses controversy because some scientists prefer (mistakenly I recently argued; Cavalier-Smith et al. 2015a) the mechanistically immensely more complex, far less likely, idea of one secondary symbiogenesis followed by multiple lateral tertiary symbiogenetic transfers—an idea that I was the first to float when we knew immensely less about protein-targeting machinery or eukaryote phylogeny than now (Cavalier-Smith et al. 1994). I remember enthusiastically discussing with Sitte and Geoff McFadden (probably at a slightly later German conference) the desirability for better understanding chromist history of sequencing the genome of cryptomonad nucleomorphs, which was eventually achieved through collaboration with Uwe Maier, who followed up the pioneering work of Eschbach in nucleomorph isolation in Sitte’s lab, and with Susan Douglas (Douglas et al. 2001). That nucleomorph sequence enabled Maier’s group to discover the molecular basis for periplastid protein targeting that is crucial for appreciating chromist unity.

I explain below that the only known example of tertiary symbiosis (Tengs et al. 2000) has been misunderstood was a chloroplast replacement that does not support tertiary acquisition by a heterotroph of any canonical chromist plastids. I predict that when the ideas and evidence explained below are more fully assimilated and tested, and different lines of evidence (only superficially seemingly contradictory) more soundly evaluated for their relative strength, my old speculation that tertiary transfers of red algal plastids might possibly account for chromophyte diversity (Cavalier-Smith et al. 1994) will be seen to be the red herring I later judged it to be ever since thinking that alveolate plastids arose in the same secondary symbiosis as euchromists (Cavalier-Smith 1999). The idea of chromist holophyly including alveolates, Rhizaria, and heliozoans (Cavalier-Smith et al. 2015) eventually ought also to become universally agreed, but conservatism and complexity of the issues could delay this another decade.

## Expansion of kingdom Chromista to include alveolates, Rhizaria, and heliozoa

Cavalier-Smith (2010) substantially expanded Chromista because of multiprotein eukaryote phylogenies that confirmed that many former Protozoa are specifically related to chromist lineages (Burki et al. 2008, 2009), as the first taxonomically sufficiently comprehensive rDNA maximum likelihood trees had shown without significant bootstrap support (Cavalier-Smith 1993a, 1995a; Cavalier-Smith et al. 1994). Chromista therefore are distinguished from the other four eukaryote kingdoms by a combination of cell ultrastructure and phylogeny. Chromista now include numerous ex-Protozoa as well as all chromophyte algae, plus the rhizarian chlorarachnids whose chloroplasts originated by enslavement of a green alga and convergently acquired two extra surrounding membranes similarly to euchromist plastids (Cavalier-Smith 2006a; Hopkins et al. 2012). Together with Plantae, chromists constitute the superkingdom Corticata (Cavalier-Smith et al. 2015a), a robust clade on eukaryote multiprotein trees (Fig. 2) initially called corticates (Cavalier-Smith 2003b; Cavalier-Smith and Chao 2003a). Most non-parasitic heterotrophic chromists are phagotrophs, as are many chromophyte algae, only a few of whose lineages evolved cell walls, unlike all Plantae lineages except prasinophytes, one subgroup of which retains phagotrophy.

Initially, Chromista excluded dinoflagellates whose chlorophyll *c*-containing plastids have only three bounding membranes not four and their outermost membrane neither bears ribosomes nor is continuous with the nuclear envelope, unlike algal euchromists (Cavalier-Smith 1981a); my defunct postulate that dinoflagellate triple plastid envelopes arose independently of the euchromist four-membrane pattern and might be related to euglenoid chloroplasts also with a triple envelope and possibly closer to plant chloroplasts than to chromists (Cavalier-Smith 1982) was refuted by sequence phylogeny. Later, I argued that dinoflagellates are related to parasitic superclass Sporozoa (gregarines and Coccidiomorphea) with which they share ampulliform mitochondrial cristae (Cavalier-Smith 1987b), so grouped them together as Miozoa (now a phylum), not specifically related to phylum Euglenozoa (euglenoids, kinetoplastids, diplonemids, postgaardeans) with discoid mitochondrial cristae. Further reevaluating ultrastructural characters led me to group Miozoa and phylum Ciliophora (ciliates, suctorians) as protozoan infrakingdom Alveolata characterised by tubular mitochondrial cristae and cortical alveoli (smooth membrane sacs that strengthen the cell cortex by firm attachment to overlying plasma membrane and underlying microtubules) (Cavalier-Smith 1991). 18S rDNA trees rapidly supported the postulated monophyly of Miozoa and of alveolates (Wolters 1991). Subsequent discovery of plastid DNA in coccidiomorphs (e.g. malaria parasites) showed that the common ancestor of Miozoa was probably photosynthetic, implying that numerous heterotrophic dinoflagellates had lost photosynthesis (Palmer 1992) and that all Miozoa obtained their plastids in the same secondary symbiogenetic event and opened the possibility that alveolates and euchromists might share an algal common ancestor, entailing plastid loss by the ciliate ancestor (Cavalier-Smith 1995a p. 91).

**Fig. 3:**
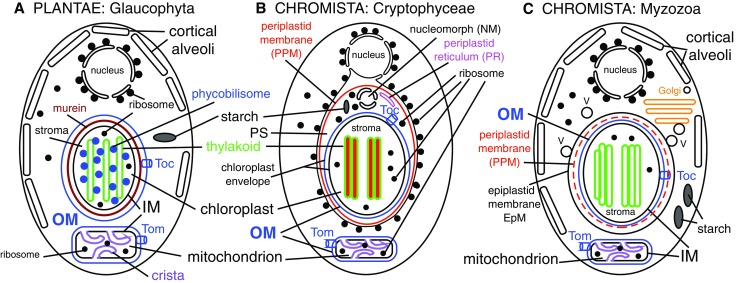
Contrasting membrane topology of Plantae and algal Chromista (superkingdom Corticata). Plantae (**a**) originated by primary enslavement of a cyanobacterium to make plastids and Chromista (**b**, **c**) by secondary intracellular enslavement of a red algal plant. Both target nuclear-coded proteins to plastids by transit peptides (*TPs*) recognised by outer membrane (*OM*, *blue*) Toc receptors and to mitochondria (enslaved α-proteobacteria) by topogenic sequences recognised by OM Tom receptors. For clarity, Golgi shown only in **c** and peroxisomes and lysosomes omitted. **a** *Cyanophora*, from the earliest diverging plant phylum Glaucophyta. Plastid membrane topology is identical to cyanobacteria with thylakoids. The common ancestor of red algae and green plants (not shown) lost cortical alveoli (which grow by fusion of Golgi-derived vesicles), red algae and two green plant subgroups lost chloroplast envelope murein peptidoglycan, and green plants lost phycobilisomes and stack their thylakoids. **b** Cryptophytes retain the enslaved red algal nucleus (simplified to a tiny nucleomorph), starch, and cytosolic ribosomes within the periplastid space (*PS*), and phycobilins (shown in *red* but can be *blue* instead) in the thylakoid lumen; all other euchromists (haptophytes, Ochrophytina, not shown) lost these four components and stack their thylakoids in threes not pairs, but like cryptophytes retained the red algal plasma membrane as the periplastid membrane (*PPM*) and a periplastid reticulum (*PR*) here argued to be the relict *trans*-Golgi network (*TGN*) of the enslaved red alga and topologically distinct from the PPM. **c** Myzozoa lack periplastid ribosomes, phycobilins, and nucleomorph DNA; thylakoids are stacked in threes; PPM (present in Apicomplexa—*red dashed line*; lost in Dinozoa) and plastid are not within the rough ER. The original phagosome membrane (now epiplastid membrane, *EpM*) remains smooth and receives vesicles (*V*) containing nucleus-encoded plastid proteins from the Golgi. Dinozoa lack PR, but Apicomplexa have a likely homologue (not shown)

Discovery of coccidiomorph plastids and alveolates grouping within or as a sister to chromists on our 18S rDNA maximum likelihood and parsimony (but not distance) trees (Cavalier-Smith 1995a; Cavalier-Smith et al. 1994) made it more plausible than before that dinoflagellate chloroplasts had lost the euchromist PPM (Fig. 3). Thenceforth, I seriously entertained the possibility that Miozoa and euchromists had a common origin by one enslavement of a red alga (Cavalier-Smith 1995a), called the chromalveolate hypothesis when more strongly arguing for euchromists plus Alveolata being a clade (Cavalier-Smith 1999). After, it was convincingly shown that coccidiomorph plastids are bounded by four membranes (Kohler et al. 1997) as in euchromists, not three as in dinoflagellates, I accepted that miozoan chloroplasts originated by *secondary symbiogenesis*: the internal enslavement of a phagocytosed eukaryote—in contrast to the primary symbiogenesis of a cyanobacterium that generated Plantae. I therefore argued that alveolates and classical chromists probably share basically the same protein import machinery and form a single ‘chromalveolate’ clade that originated by the same enslavement of a red alga (Cavalier-Smith 1999), not independent enslavement for dinoflagellates (Gibbs 1981a; Whatley et al. 1979; Whatley 1989). The possibility of secondary symbiogenetic origin of triple-membrane plastids (Tomas and Cox 1973; Gibbs 1978) once seemed a less parsimonious explanation than direct descent from the original two-membrane cyanobacterial ancestor of plant plastids by retaining the host phagosomal membrane to make three (Cavalier-Smith 1982) but is now universally accepted.

I proposed the initial step of plastid protein import for both dinoflagellates and Sporozoa to be translocation across ER membranes via an N-terminal signal sequence recognised by the same signal recognition particle (SRP) that initiates protein secretion via ER and Golgi (Cavalier-Smith 1999). If correct, the outermost membrane around miozoan plastids is homologous not with the plasma membrane (PM) of a secondary symbiont, as Gibbs (1978, 1981a) suggested, but with the phagosomal membrane as in Cavalier-Smith (1982); thus, miozoan plastids are topologically within the endomembrane system as in euchromists, entirely unlike plants, since decisively confirmed (Heiny et al. 2014); it follows that dinoflagellates lost the PPM from between the rough ER membrane and chloroplast envelope. By contrast in euchromists and apicomplexans, the PPM is a remarkably persistent evolutionary relic of the PM of the biliphyte alga that was enslaved to make the ancestral chromist chloroplast as Cavalier-Smith (1981a, b, 1982) first argued; dinoflagellates are the only chromophytes that lost it. As argued early on (Cavalier-Smith 1982), evolving novel protein import machinery for secondary plastids is far more difficult than myriad authors who have assumed a polyphyletic symbiogenetic origin of chromists suppose (e.g. Margulis 1970) and the major reason why euchromist chloroplasts could only have originated once, fully justifying a separate kingdom from Plantae (Cavalier-Smith 1986).

This inference gained further strength with discovery of *Chromera* (Moore et al. 2008), an evolutionarily distinctive coral reef alga, which phylogenetically nests within class Apicomonadea that is a sister to Sporozoa and is grouped with them as miozoan infraphylum Apicomplexa (Cavalier-Smith 1993a; Ruggiero et al. 2015). Classical apicomonads are biciliate predators on protists, using apical complex organelles to suck contents of their prey’s PM into a food vacuole for digestion. This predatory method (myzocytosis) excludes prey’s PM from the food vacuole, whereas phagocytosis includes it. Classical apicomonads like *Colpodella* and *Voromonas* are all heterotrophs but phylogenetically diverse (Cavalier-Smith and Chao 2004). As *Chromera* and an ultrastructurally distinct photosynthetic apicomonad *Vitrella* (Oborník et al. 2012) are phylogenetically non-sister apicomonad lineages, photosynthesis was multiply lost by heterotrophic apicomonads; *Voromonas* at least retains a plastid (Gile and Slamovits 2014). The fact that *Chromera*, *Vitrella*, and dinoflagellate chloroplasts uniquely share the same type II CO_2_-fixing single-molecule RuBisCo acquired by lateral gene transfer (LGT) from proteobacteria, unlike the two subunit RuBisCos of all other eukaryotes and cyanobacteria, proves that the common ancestor of apicomonads and dinoflagellates photosynthesised using this particular RuBisCo, and its numerous heterotrophic descendants all lost photosynthesis. These include Sporozoa, six heterotrophic classes grouped with the ancestrally photosynthetic class Peridinea/Dinophyceae (that itself includes many non-photosynthetic lineages) as superclass Dinoflagellata, and the parasitic superclass Perkinsozoa that are sisters of Dinoflagellata (together infraphylum Dinozoa). As Dinozoa and Apicomplexa are robustly phylogenetic sisters, and uniquely amongst eukaryotes feed by myzocytosis mediated by similar apical structures (cytoskeleton and extrusomes), they are grouped together as miozoan subphylum Myzozoa, ancestrally with type II RuBisCo. It is now incontrovertible that the ancestral myzozoan was a myzocytotic alga and that photosynthesis was lost at least a dozen times, the exact number of losses uncertain as we lack a comprehensive well-resolved dinozoan phylogeny (Cavalier-Smith 2013a).

The fact that *Chromera* and *Vitrella* chloroplasts are separated from the cytosol by four membranes as in Sporozoa proves that ancestral Myzozoa had plastids with four membranes and dinoflagellates secondarily lost the PPM, as a later section explains. 135-protein trees (Burki et al. 2008) showed that alveolates are more closely related to the chromist infrakingdom Heterokonta than to either haptophytes or cryptophytes, the two other chromist algal groups, as some rDNA trees had earlier less convincingly indicated. These trees also strongly grouped cryptomonads and haptophytes as a clade, as predicted by their chloroplasts uniquely amongst eukaryotes having acquired the bacterial *rpl36* gene by LGT, necessarily in a common photosynthetic ancestor. A taxonomically more comprehensive 127-protein tree showed the heterotrophic flagellate *Telonema* and non-flagellate axopodial centrohelid heliozoa are also specifically related to the haptophyte/cryptophyte photosynthetic lineage (Burki et al. 2009), confirming evidence from Hsp90 trees that these four groups are a clade designated Hacrobia (Okamoto et al. 2009).

These new trees and the properties of chromeroids collectively showed that alveolates are not the sister group to chromists as previously assumed (Cavalier-Smith 1999) but phylogenetically nest within chromists, exactly as our early 18S rDNA ML trees indicated (Cavalier-Smith 1995a; Cavalier-Smith et al. 1994), as also is the largely heterotrophic infrakingdom Rhizaria (first suggested by 18S rDNA; Cavalier-Smith 1995a), as well as centrohelids and *Telonema* (Burki et al. 2009); see Fig. 1. I therefore formally transferred Alveolata, Rhizaria, centrohelids, and *Telonema* from Protozoa into kingdom Chromista (Cavalier-Smith 2010) and argued that not only a dozen or more myzozoan lineages but also Ciliophora, centrohelids, and *Telonema* had lost photosynthesis and less often also the ancestral chromist plastid. As noted above, from the start (Cavalier-Smith 1981a, 1986) it was recognised that some chromists might have lost both the chromophyte chloroplast and tubular ciliary hairs and thus were initially wrongly put in Protozoa not Chromista (e.g. ciliates; Cavalier-Smith 1995a). Even prior to the 2010 major expansion of Chromista actinophryid ‘heliozoa’ were shown to be heterokont chromists (Nikolaev et al. 2004) that had lost cilia altogether so were transferred to Heterokonta (Cavalier-Smith and Chao 2006), the latest analysis proving them to be relatives of Raphidophycidae that lost photosynthesis (Cavalier-Smith and Scoble 2013).

When expanding Chromista by adding alveolates, Rhizaria, and Centrohelea, I formally made Hacrobia a subkingdom and established the new subkingdom Harosa for the extremely robust clade comprising Heterokonta, Alveolata, and Rhizaria (Cavalier-Smith 2010). Table 1 summarises the latest classification of Chromista at high taxonomic ranks and gives diagnoses for new subgroups recognised here; a more complete classification including all 82 classes (10 new) with examples of genera included in each, plus information on new taxa etymology, is in the supplementary material (Table S1). As alveolates are phylogenetically nested within classical chromists, the interim term ‘chromalveolates’ became redundant and was abandoned as a taxon (Cavalier-Smith 2010), being mainly of historical interest for a subset of Chromista excluding the non-chromophyte Rhizaria.

**Table 1:** Revised higher classification of kingdom Chromista Cavalier-Smith 1981 and its eight phyla

**Subkingdom 1. Harosa** Cavalier-Smith, 2010 (sometimes colloquially called SAR)
**Infrakingdom 1. Halvaria** Cavalier-Smith, 2013
**Superphylum 1. Heterokonta** Cavalier-Smith, 1981 (stramenopiles), a superfluous later synonym (tripartite anterior ciliary tubular hairs) stat. n.
**Phylum 1. Gyrista** Cavalier-Smith, 1998 stat. n.
**Subphylum 1. Ochrophytina** ^a^ Cavalier-Smith, 1986 (heterokont algae and derived heterotrophs)
**Infraphylum 1. Chrysista** ^a^ Cavalier-Smith, 1991 (ancestrally with ciliary supra-tz helix)
**Superclass 1. Limnistia** ^a^ Cavalier-Smith, 1996 emend. 2006 (chrysophytes, eustigs, Picophagea)
**Superclass 2. Raphidoistia** ^a^ Cavalier-Smith, 1986 orth. mut. 2006 (Raphidophycidae and axopodial heterotrophic Raphopoda)
**Superclass 3. Fucistia** ^a^ Cavalier-Smith, 1995 (four classes of non-phagotrophic, walled marine multicellular algae, e.g. brown algae)
**Infraphylum 2. Diatomista** ^a^ Derelle et al. ex Cavalier-Smith, 2017 infraphyl. n. **Diagnosis**: typically unicells, sometimes in diatoms linear loose aggregates of cells; no cell walls; naked or with intracellular secreted silica frustules or siliceous scales; biciliate, anteriorly or posteriorly uniciliate or non-ciliate, without supra-tz helix
**Superclass 1. Hypogyrista** ^a^ Cavalier-Smith, 1995 stat. n. 2006 (Dictyochophyceae and Pinguiophyceae)
**Superclass 2. Khakista** ^a^ Cavalier-Smith, 2000 (as subphylum) stat. n. **Diagnosis**: no ciliary roots; silica frustules or scales; chloroplasts with girdle lamellae, fucoxanthin, diadinoxanthin, diatoxanthin; almost all phototrophs. Classes Bolidophyceae, Diatomeae (syn. Bacillariophyceae)
**Subphylum 2. Bigyromonada** ^b^ Cavalier-Smith, 1998 (marine biciliate phagoheterotrophs). **Developea** cl. n. Aleoshin et al. 2016 ex Cavalier-Smith, 2017 (e.g. *Developayella*, *Develorapax*). **Diagnosis**: biciliate non-amoeboid phagoheterotrophs; cortical alveoli underlie part of cell surface; 6-gyre, obviously double TH; one or two retroneme rows. **Pirsonea** cl. n. (*Pirsonia*) **Diagnosis**: as for sole order Pirsoniida (Cavalier-Smith and Chao 2006 p. 404). Includes also environmental DNA clades MAST1/23, 2 and Aleoshin et al.’s (2016) Ochrophytina-associated grade if heterotrophs
**Subphylum 3. Pseudofungi** Cavalier-Smith, 1986 (walled heterotrophs: Oomycetes, Hyphochytrea)
**Phylum 2. Bigyra** Cavalier-Smith, 1998 em. 2006 (heterotrophs; mostly wall-less phagotrophs; 9 classes, 2 new)
**Subphylum 1. Opalozoa** Cavalier-Smith, 1991 em., stat. n. 2006 (heterotrophs, most phagotrophic)
**Infraphylum 1. Placidozoa** Cavalier-Smith and Scoble 2013
**Superclass 1. Wobblata** Cavalier-Smith and Chao 2006 stat. n. 2013 (3 classes e.g. Placididea, Nanomonadea)
**Superclass 2. Opalinata** Wenyon, 1926 em. Cavalier-Smith, 2006 stat. n. 2013 (Opalinea, Blastocytea)
**Infraphylum 2. Bikosia** ^c^ infraphyl. n. **Diagnosis** as for subclass Bikosidae (Cavalier-Smith and Chao 2006, p. 404)
**Subphylum 2. Sagenista** Cavalier-Smith, 1995 stat. n. 2006 (classes Labyrinthulea and **Eogyrea** cl. n. **Diagnosis**: phagotrophic biciliate planktonic/benthic bigyrans with R3 and R4 anterior and split posterior R2, singlet and R1 mt centriolar roots but no X mt (unlike most Bikosia); undulating anterior cilium with 2 rows of bipartite retronemes. Originally name for clade L (Cavalier-Smith and Scoble 2013; phylogenetically closer to Labyrinthulea than to Opalozoa, Derelle et al. 2016), comprising MAST-4, MAST-6 (e.g. *Pseudophyllomitus* in new order **Eogyrida, Diagnosis:** cylindrical supra-transition helix (TH), typically swimmers, motion spiral, not gliders; subapical ciliary depression, not ventral groove; sole family Pseudophyllomitidae Shiratori et al., 2017) and MAST-7-11 **Bigyra incertae sedis**: **New class Platysulcea**; diagnosis as for naked phagotrophic biciliates, glide on long posterior cilium associated with ventral feeding groove or swim with wobbling motion; R3 and R4 anterior and split posterior R2, singlet and R1 mt centriolar roots; undulating anterior cilium with 2 rows of short bipartite retronemes; no TH. **Etym**: *platy* L. wide, *sulcus* L. groove. Sole family Platysulcidae Shiratori et al., 2015. (*Platysulcus*)
**Superphylum 2. Alveolata** Cavalier-Smith, 1991 stat. n. 2013 (cortical alveoli; 28 classes)
**Phylum 1. Miozoa** Cavalier-Smith, 1987 (ciliary hairs non-tubular; uninucleate, usually haploid)
**Subphylum 1. Protalveolata** ^b^ Cavalier-Smith, 1991 stat. n. 1999 em. (biciliates, myzocytosis unknown; Acavomonadea, Colponemida, and **Palustrimonadida** ord. n. **Diagnosis**: ventrally grooved biciliates differing from Colponemida by being less flattened, more rigid, and anterior cilium emerging from deep pocket separate from main longitudinal ventral groove. Contains only new family **Palustrimonadidae** with same diagnosis; type genus *Palustrimonas* Patterson and Simpson (1966, p. 443)
**Subphylum 2. Myzozoa** ^a^ Cavalier-Smith and Chao 2004 (myzocytotic; cytosolic chloroplasts (type II RuBisCo) or leucoplasts; epiplastid membrane separate from rough ER)
**Infraphylum 1. Dinozoa** ^a^ Cavalier-Smith, 1981 stat. n. 2013 em. (10 classes)
**Parvphylum 1. Perkinsozoa** Norén and Moestrup, 1999 em. Cavalier-Smith, 2014 stat. n
**Parvphylum 2. Dinoflagellata** ^a^ Bütschli, 1885 stat. n. em. (Phycodnavirus-like basic chromatin proteins; 10 classes)
**Superclass 1. Eodina** supercl. n. **Diagnosis**: Free-living ancestrally with ciliary web scales and posterior criss-cross latticed posterior ciliary lattice, two pronounced ciliary grooves; anterior groove separating rounded cell anterior and posterior is oblique or transverse but not a helicoidal cingulum (unlike Syndina and Dinokaryota). Nuclear chromatin ultrastructurally normal. Classes Oxyrrhea and **Myzodinea** cl. n. **Diagnosis**: Laterally biciliate myzocytotic predatory zooflagellates with discrete, often swollen cortical alveoli and extremely pronounced transverse or oblique anterior ciliary groove; rounded cell apex (non-rostrate, unlike most Apicomonadea) with micronemes and/or rhoptry-like dense extrusomes, and pseudoconoid-like short microtubules connected to long band of microtubules bypassing kinetid; ancestrally with ciliary web scales and singlet posterior microtubular root centrally supporting posterior groove floor; anterior ciliary hairs; ciliary transition zone with concave-sided cone, central pair with 2 laterobasal axosomes. Bipartite trichocysts with square cross-section dense basal zone. Unlike Peridinea, Sulcodinea, and *Oxyrrhis*, left posterior ventral centriolar root more strongly developed than right. **Sole order Myzodinida** ord. n. **Diagnosis**: as for Myzodinea. **Colpovoridae** fam. n. diagnosis as for its type genus ***Colpovora*** gen. n. **Diagnosis**: posterior right centriolar root of about 12 microtubules without I fibre; left root with at least 3 microtubules; posterior cilium with paraxonemal rod with cross lattice as in *Oxyrrhis*; anterior cilium with simple hairs. Oblique/transverse binary cell division not within cyst. Centriole angle slightly obtuse. Type species *Colpovora unguis* comb. n. Basionym *Colpodella unguis* Patterson & Simpson (1996 p. 439). **Psammosidae** fam. n. **Diagnosis**: both cilia covered by oval cobweb scales and two hair rows; hairs with thicker, non-rigid shaft and 1–2 terminal filaments. Centriole angle strongly obtuse, much less than 180°, unlike Algovorida and Colpovoridae. Transverse binary division. Type genus *Psammosa* Okamoto et al. (2012)
**Superclass 2. Syndina** Cavalier-Smith, 1993 em. Classes Syndinea, Ellobiopsea, and **Endodinea** cl. n. **Diagnosis**: Parasites of Rhizaria, Alveolata, and fish eggs. Phylogenetically defined as all dinoflagellates more closely related to *Ichthyodinium* and *Dubosquella* than to *Syndinium* or *Oxyrrhis* (i.e. group I marine alveolates). Multiply within sporangia; nucleus with normal chromatin. Without body or ciliary scales. Cilia without paraxonemal rods or vanes. Contains only new order **Ichthyodinida**, diagnosis as for Endodinea. Includes Dubosquellidae Chatton 1920 ex Loeblich II, 1970 (e.g. *Dubosquella*) and new family **Ichthyodiniidae**: **Diagnosis**: Endoparasites of fish eggs; comprises lineages phylogenetically closer to *Ichthyodinium* than to *Dubosquella*. Type genus *Ichthyodinium* Hollande and Cachon, 1952
**Superclass 3. Dinokaryota** ^a^ Cavalier-Smith, 1993 em. (Histone-like protein HLP-II; liquid crystalline nuclear DNA organisation); classes Noctilucea, Peridinea^a^ (subclasses Dinophycidae^a^ (incl. **Spirodinida** ord. n. **Diagnosis**: episomal microtubules terminate substantially subapically at a spiral microtubule bounding an apical spiral groove curving clockwise seen from apex. Includes **Akashiwidae** fam. n. diagnosis as for Spirodinida (Type genus *Akashiwo* Hansen and Moestrup in Daugbjerg et al. (2000 p. 308)) and **Epidinia** infracl. n. **Diagnosis**: episome much larger than hyposome. **Torodinida** ord n. **Diagnosis**: as for the infraclass (*Torodinium*, *Labourodinium*)} and **Karlodinia** ^a^ subcl. n. **Diagnosis**: plastids of haptophyte origin with 19-hexanoyl-fucoxanthin, not peridinin, with atypical envelope; cingulum steeply loop-like; divides small pointed epicone from large rounded hypocone (*Brachidinium*, ‘*Karenia*’, *Karlodinium*, *Takayama*), and **Sulcodinea** ^a^ cl. n. **Diagnosis**: dinokaryotes with either very long anterior sulcal extension so cingulum starts less than one third of cell length from its pointed apex (*Gyrodinium*) or with sulcus merging into an initially longitudinal cingulum about one third from apex that loops steeply round narrowly pointed cell apex and its cytoskeleton passing backward ventrally parallel to sulcus (*Amphidinium*). Plastids triple envelope. **Gyrodinida** (e.g. *Gyrodinium*) ord. n. **Diagnosis**: heterotrophs with spiral cingulum. **Amphidinida** ord. n. **Diagnosis**: plastids with peridinin and triple envelope; cingulum steeply loop-like, divides small pointed epicone from large rounded hypocone (*Amphidinium*, *Bispinodinium*)
**Infraphylum 2. Apicomplexa** ^a^ Levine 1970 em., stat. n. Cavalier-Smith, 2013
**Parvphylum 1. Apicomonada** ^a^ Cavalier-Smith, 1993 stat. n., em. Class Apicomonadea^a^ Cavalier-Smith, 1993 em. Comprises two subclasses: Myzomonadia Cavalier-Smith in Cavalier-Smith and Chao, 2004 stat. n., em. **Diagnosis**: with pseudoconoid or paraconoid; phototrophs or heterotrophs; divide within cysts into 2, usually 4, or 8 cells. **Superorder 1. Chromovoridia** ^a^ superord. n. **Diagnosis**: photosynthetic or heterotrophic myzocytotic predators with preciliary rostrum containing a pseudoconoid of numerous mts, having 2–3 lumenal microtubules; encysted cells divide into four daughters, but in some vegtative cells undergo binary fission. Orders Chromerida^a^ (*Chromera* only), Voromonadida, Algovorida, and **Voracida** ord. n. **Diagnosis**: no trichocysts; unlike all other apicomonads, centrioles extremely short, basally chamfered, not mutually orthogonal, joined by unique lamellate desmose; highly compressed cortical alveoli, not obviously subdivided in thin sections; anterior cilium in pit with a micropore, with lateral paraxonemal rod basally; its single mt root supports cell apex. **Microvoracidae** fam. n. Diagnosis as for type genus ***Microvorax*** gen. n.: cell apex rounded, not pointed as in *Dinomonas*, *Chilovora*, *Colpodella*; cilia only slightly subapical, one points anteriorly; centrioles close, only anterior (slender paraxonemal rod) in shallow pit, about one centriole-width apart with short desmose; small pimple-like cell protuburance between them; without oblique root; unlike *Dinomonas* posterior cilium at cell surface, not in pit. Feed on bodonids or ciliates; freshwater. Type species *Microvorax angusta* sp. n. (Syn. *Spiromonas angusta* sensu Krylov and Mylnikov, 1986; **not** *Heteromita angusta* Dujardin, 1841). **Diagnosis**: elongate cell 8–10(−18) × 3–4(−10) μm; cilia ~ 1.6X cell length; pseudoconoid of 24–5 strongly decorated mts, contains pear-shaped dense bodies, and probably 2 lumenal mts; rhoptries absent. Thin-walled cyst (7–8 μm) divides into 4 daughters. Type strain Spi-2 (Mylnikov, Borok, Russia); type rDNA sequence its KU159286; but morphological description based on a strain (Krylov and Mylnikov 1986, type figures; now lost, unsequenced; see also Mylnikov 1983) isolated from same Borok sewage works (later called S-1: Mylnikov 1991) and ‘very similar’ by LM (Mylnikov pers. com.). Other species: *Microvorax tetrahymenae* comb. n. Basionym *Colpodella tetrahymenae* Cavalier-Smith in Cavalier-Smith & Chao (2004 p. 194); *Microvorax gonderi* comb. n. Basionym *Spiromonas gonderi* Foissner and Foissner (1984). **Dinomonadidae** fam. n. **Diagnosis**: myzocytotic predators on ciliates and other heterotrophs with two subequal posteriorly directed cilia longer than cell body with widely separate centrioles set in distinct pits (anterior deep, posterior shallow) about 2 μm behind pointed tip of rostrum. Rhoptries of two types. Prominent oblique mt root to cell’s right of kinetid (Brugerolle 2002a Fig. 3). Anterior amorphous ciliary paraxonemal rod present basally. Subpellicular microtubules only in anterior third, mainly dorsal, rostral. Anterior root outside pseudoconoid. Desmose several times longer than centriole width. Type genus *Dinomonas* Saville Kent, 1880–1. *D. vorax* Saville Kent, 1880–1 [syn. *Colpodella vorax* Simpson and Patterson, 1996). **Superorder 2. Paraconoidia** superord. n. **Diagnosis**: heterotrophic biciliate predators with small but distinct curved pointed rostrum with numerous evenly spaced subpellicular microtubules attached beneath strongly flattened cortical alveoli; pseudoconoid wall mts absent; bypassing microtubular band with spiral I-fibre-like extension with two attached microtubules at its tip curves round microneme and rhoptry tips and 5-microtubule anterior centriolar root as a ‘paraconoid’ proximal to preparaconoidal ring; divide into four or eight daughters within cysts; shallow ventral longitudinal groove. Sole order Colpodellida. **New subclass Vitrelloidia** ^a^ **Diagnosis**: as for sole order **Vitrellida** ^a^ ord. n.: Phototrophs dividing within sporangia into numerous daughters. Pseudoconoid or paraconoid absent. Outer cortical alveolar layer continuous (not discrete as in *Chromera*’s single cortical alveolar layer); second inner layer of discrete cortical alveoli. (*Vitrella*)
**Parvphylum 2. Sporozoa** Leukart, 1879 stat. n. Cavalier-Smith, 2014 (Cocciodiomorphea, Gregarinomorphea, Paragregarea)
**Phylum 2. Ciliophora** Doflein, 1901 (ciliates, suctorians; nuclear dimorphism; no plastids; 12 classes)
**Subphylum 1. Intramacronucleata** Lynn, 1996 (spindle in macronucleus; kinetodesmal fibre)
**Infraphylum 1. Spirotrichia** Cavalier-Smith, 2004 em. (4 classes)
**Infraphylum 2. Ventrata** Cavalier-Smith, 2004 (ventral mouth; 5 classes)
**Infraphylum 3. Protocruzia** infraphyl. n. and new class **Protocruzea** cl. n. **Diagnosis** for both as for subclass Protocruziidia De Puytorac et al., 1987 (Lynn and Small 2002 p. 421). (*Protocruzia*) Deeper branch on multigene trees than preceding infraphyla (Gentekaki et al. 2017)
**Subphylum 2. Postciliodesmatophora** Gerassimova and Seravin, 1976 (Karorelictea, Heterotrichea)
**Infrakingdom 2. Rhizaria **Cavalier-Smith, 2002 em. 2003 (reticulose or filose pseudopodia; rare ciliary hairs non-tubular; 18 classes)
**Phylum 1. Cercozoa** Cavalier-Smith 2008 em. (cortical alveoli absent; extrusomes mostly globular; 8 classes, 1 new)
**Subphylum 1. Reticulofilosa** ^b^ Cavalier-Smith, 1997. Skiomonadea, Granofilosea and Chlorarachnea Hibberd and Norris, 1984 orth. em. Cavalier-Smith, 1986 **incl**. Chlorarachnida and **Minorisida** ord. n. **Diagnosis and etymology**: as for **Minorisidae** fam. n. **Diagnosis**: Minute marine phagoheterotrophic picoplanktonic bacterivorous flagellates with single long acronematic smooth cilium. Type genus *Minorisa* Del Campo in Del Campo et al. (2013 p. 355)
**Subphylum 2. Monadofilosa** Cavalier-Smith, 1997 (heterotrophic flagellates, amoeboflagellates or amoebae; pseudopods mostly filose)
**Superclass 1. Eoglissa** Cavalier-Smith in Cavalier-Smith and Oates, 2011 em. Metromonadea and **Helkesea** cl. n. **Diagnosis**: apically or subapically biciliate zooflagellates with posterior ciliary gliding and extrusomes, plus related tetraciliate parasites and guttulinopsid lobose amoebae; flagellates either with anterior cilia just a stub without 9 + 2 axoneme or dorsoventrally flattened thecate biciliates with normal anterior cilium and filose pseudopods emanating from a short posterior ventral slit separate from ciliary apertures that are phylogenetically closer to them than to Ventrifilosa. Sole Orders Ventricleftida and **Helkesida** ord. n. **Diagnosis**: biciliate or tetraciliate zooflagellates with anterior cilium of each kinetid reduced to a stub, plus lobose non-ciliate amoebae phylogenetically closer to them than to Ventrifilosa. Centriolar roots highly simplified sometimes to as few as three microtubules. Flat mitochondrial cristae, unlike most Rhizaria. i.e. Sainouroidea Cavalier-Smith in Cavalier-Smith et al., 2009, emended here by excluding *Helkesimastix*, and **Helkesimastigoidea** superfam. n. with families Helkesimastigidae and Guttulinopsidae
**Superclass 2. Ventrifilosa** Cavalier-Smith in Cavalier-Smith and Karpov, 2012 (sarcomonads, imbricates, Thecofilosea)
**Phylum 2. Retaria** Cavalier-Smith, 1999 em. (heterotrophs with reticulopodia; 10 classes, 1 new)
**Subphylum 1. Endomyxa** Cavalier-Smith, 2002
**Superclass 1. Marimyxia** supercl. n. **Diagnosis**: trophically non-ciliate marine amoeboids without central capsule; free-living reticulose cells or amoeboid entirely non-ciliate parasites of marine invertebrates with complex spores with one or more cells and no polar capsules or filaments. Gametes (*Gromia* only) uniciliate. Phylogenetically includes free-living Gromiidea and their parasitic ascetosporan descendants
**Superclass 2. Proteomyxia** Lankester, 1885 ex Cavalier-Smith, 2017 stat. n. **Diagnosis**: Heterotrophic non-ciliate amoeboid free-living reticulose or filose protists (Vampyrellidea), typically mycophagous or algivorous, and amoeboid or plasmodial trophically non-ciliate parasites (of plants or algal chromists) with biciliate dispersal stage (Phytomyxea). Phytomyxea and **Vampyrellidea** cl. n. Diagnosis as for Vampyrellida in Hess et al. (2012 p. 10)
**Subphylum 2. Ectoreta** subphyl. n. **Diagnosis**: ancestrally marine; large-celled, uninucleate or multinucleate, non-ciliate, reticulose trophic phase typically grows manyfold (for weeks or months), then undergoes multiple fission into much smaller cells (binary fission in a few); usually with smaller usually biciliate swimming (not gliding) gametes or zoospores; distinguished from Endomyxa by cells divided by test or capsule into central nuclear region containing mitochondria, Golgi apparatus, and endoplasmic reticulum, and outer ectoplasm of reticulopodia; uniquely use novel 2 tubulins (Hou et al. 2013)
**Infraphylum 1. Foraminifera** D’Orbigny, 1826 ex Cavalier-Smith 2017 stat. n.
**Infraphylum 2. Radiozoa** Cavalier-Smith, 1987 em. 2003 stat. n.
**Superclass 1. Polycystinia** Ehrenberg, 1838 stat. n.
**Superclass 2. Spasmaria** Cavalier-Smith, 1993 stat. n. (Acantharea, Sticholonchea)
**Subkingdom 2. Hacrobia** Okamoto et al. ex Cavalier-Smith, 2010 (biciliates or non-ciliated axopodial)
**Phylum 1. Cryptista** Cavalier-Smith, 1989 em. 2015 (no cortical alveoli; bipartite tubular hairs ancestrally; 7 classes)
**Subphylum 1. Rollomonadia** Cavalier-Smith, 2013
**Superclass 1. Cryptomonada** Cavalier-Smith, 2004 (as subphylum) stat. n. 2015 (cryptophytes^a^; *Goniomonas*) *Hemiarma* Shiratori and Ishida, 2016 type genus of **Hemiarmidae** fam. n. **Diagnosis:** unlike Goniomonadidae Hill, 1991 periplast plates polygonal, not square, and cover only right half of cell, and ciliary transition plate is single. Put in **Hemiarmida** ord. n. with same diagnosis
**Superclass 2. Leucocrypta** Cavalier-Smith, 2004 (as subphylum stat. n. 2015: kathablepharids)
**Subphylum 2. Palpitia** Cavalier-Smith in Cavalier-Smith and Chao, 2012 (*Palpitomonas*)
**Subphylum 3. Corbihelia** Cavalier-Smith in Cavalier-Smith, Chao, Lewis, 2015
**Superclass 1. Endohelia** Cavalier-Smith in Cavalier-Smith, Chao, Lewis, 2015 (*Microheliella*, *Heliomorpha*)
**Superclass 2. Corbistoma** Cavalier-Smith in Cavalier-Smith, Chao, Lewis, 2015 (*Picomonas*, Telonemea)
**Phylum 2. Haptista** Cavalier-Smith, 2003 stat. n. 2015 (cortical alveoli; diverse surface microtubule skeletons)
**Subphylum 1. Haptophytina** ^*^ Cavalier-Smith in Cavalier-Smith, Chao, Lewis, 2015 (3 photosynthetic classes)
**Subphylum 2. Heliozoa** Haeckel 1866 stat. n. Cavalier-Smith in Cavalier-Smith et al., 2015 (Centrohelea)

^a^Taxa that are certainly ancestrally photosynthetic

^b^Probably paraphyletic

^c^Validates this clade name as an infraphylum; Cavalier-Smith and Scoble (2013) inadvertently omitted reference to this diagnosis when introducing it

In Burki et al. (2009), Hacrobia and Chromista were reasonably well-supported clades, but later studies found marked differences in basal corticate phylogeny that depend on taxon sampling and analytic method; Plantae, Chromista, and Hacrobia sometimes seem to be clades, sometimes not. Plantae, Hacrobia, Harosa, and Corticata are maximally supported clades on a site-heterogeneous 478-protein tree, but Harosa appears not as a sister to Hacrobia but (probably artefactually; see later section) one node deeper (Ren et al. 2016). Reasons for these inconsistencies were systematically studied and discussed in detail by Cavalier-Smith et al. (2015a), who found stronger evidence for chromist and especially hacrobian monophyly than most studies and concluded that tree inconsistencies stem largely from corticate primary radiation being explosively rapid after the origin of chloroplasts, so relatively little evidence for their correct ancestral topology remains. This problem may be exacerbated by chromist nuclei necessarily being eukaryote-eukaryote chimaeras genetically, making trees easily influenced by phylogenetic artefacts from any wrongly included red algal genes. Mitochondrial genome trees with no such chimaera problem show Hacrobia and Plantae as clades (Jackson and Reyes-Prieto 2014) as also do chloroplast genome trees (Kim et al. 2015), also not affected by the certainly chimaeric nature of chromist nuclei. Contrary to many assertions, multiprotein trees from all three genomes are congruent if interpreted critically; all are consistent with a single red algal enslavement by the ancestral chromist (Figs. 1 and 2) and its subsequent vertical inheritance except for a single tertiary lateral transfer of chloroplasts from a haptophyte to karlodinian dinoflagellates, replacing the original dinoflagellate plastid (Tengs et al. 2000).

## Rampant losses of photosynthesis and plastids in Chromista

Often when eukaryotes lose photosynthesis, they retain plastids as colourless leucoplasts. As previously explained (Cavalier-Smith 1993b), leucoplast retention occurs because most lineages sooner or later come to depend on plastids for function(s) other than photosynthesis. Algal chromists lost the eukaryotic host fatty acid (FA) synthetase, just as did the ancestor of plants which instead kept cyanobacterial FA synthetase, and evolved FA export from plastid to cytosol. As the enslaved red alga already had the plant FA export machinery, as plastids contain the majority of cellular FAs, this probably predisposed chromists to lose the host rather than red algal FA synthetase—but only if FA export across the PPM to ER membranes improved. Coccidians and other apicomplexans also lost host enzymes for isoprenoid lipid synthesis, iron-sulphur clusters, and haem and therefore had to keep leucoplasts (enclosed by PPM and ER membranes, the whole complex called an ‘apicoplast’; McFadden 2011) for making haem as well as FAs and isoprenoids. One clade of gregarine apicomplexa (subclass Orthogregarinia plus *Cryptosporidium*; Cavalier-Smith 2014a) was able to lose apicoplasts as these parasites could import these essentials from their animal hosts’ gut. It would have been even easier for free-living phagotrophs to have lost plastids altogether if they diverged so early that the host cell had not yet become dependent on plastids for making lipids, haem, or amino acids.

There was therefore no evolutionary obstacle to such lineages easily losing plastids, especially if they evolved novel feeding modes, giving advantages over other heterotrophic protists. Ciliophora achieved giant cell size without prejudicing rapid growth by evolving ciliary rows (kineties), mouth, and macronuclei (Cavalier-Smith 2004a); Rhizaria evolved novel branching pseudopodia for feeding, and axopodial feeding evolved in actinophryid heterokonts (Cavalier-Smith and Scoble 2013), a few Rhizaria, and several Hacrobia (Cavalier-Smith et al. 2015a). Within heterokonts (see Cavalier-Smith and Scoble 2013), Sagenista (Labyrinthulea) evolved a unique net-like scale-covered saprotrophic way of life (Anderson and Cavalier-Smith 2012), Pseudofungi evolved cell walls and osmotrophy, and Bikosia modified their cytoskeleton to facilitate trapping prey brought by basipetal water currents of the anterior cilia (rather than the acropetal currents of the posterior cilium in excavate protozoan ancestors of chromists). Such early diverging heterotrophic chromists could easily have lost plastids, so (contrary to frequent naive assumptions) it is not in the least unparsimonious to suggest several such early plastid losses. For particularly early losses (Fig. 1), there may be no trace of the originally chimaeric nature of the chromist ancestor. On the contrary, late losses of photosynthesis left obvious traces in the form of leucoplasts—in heterotrophic Ochrophytina (e.g. pedinellids, chrysomonads), Cryptista (*Cryptomonas paramecium*), many Dinozoa, and Myzozoa [apicoplasts in *Voromonas*, coccidiomorphs, some gregarines (Paragregarea)]. Thus, early in chromist evolution, photosynthesis and plastids were both easily lost, yielding early diverging heterotrophic lineages, but loss became harder and harder as the host became irreversibly dependent on plastids.

If photosynthesis is lost, relict plastids may retain plastid DNA (e.g. most Sporozoa, chrysophytes, pedinellids) or lose plastid DNA but not plastids (most Dinozoa) or plastids may disappear totally (e.g. Syndinea, Gregarinomorphea). Heterotrophic dinoflagellates easily lose plastid DNA as their chloroplast genomes encode only photosynthetic proteins (always minicircles in dinophytes, mostly single gene; Dorrell et al. 2017; Zhang et al. 1999); the presence of plastid-derived metabolic pathways mediated by proteins with N-terminal topogenic sequences suitable for import across three membranes proves that heterotrophs in three classes (*Oxyrrhis*, *Noctiluca*, and *Dinophysis* in Peridinea; Janouškovec et al. 2017) retain plastids. Similar evidence is needed for the most primitive dinoflagellate class Myzodinea (Table 1) and for actinophryid ochrophytes (Cavalier-Smith and Scoble 2013), both of which lost photosynthesis—almost certainly after their ancestors became irreversibly dependent on plastid metabolism. The first rDNA trees for dinoflagellate chloroplasts could not clarify their evolutionary affinities because minicircle sequences evolve exceedingly fast, yielding hard-to-place long branches. Sequence trees combining all 12 minicircle proteins now show dinoflagellate plastids as a sister to those of apicomplexan *Vitrella* (Dorrell et al. 2017), proving that myzozoan chloroplasts are monophyletic; thus, their common ancestor acquired type II RuBisCo by LGT from a proteobacterium after it diverged from their sister algal group Heterokonta, a unifying feature distinguishing Myzozoa from all other eukaryotes. This 12-photosynthetic protein tree is congruent with nuclear 101-protein trees (Janouškovec et al. 2017) in thecate dinoflagellates being a clade nested within ancestral naked lineages and *Amphidinium* diverging before Peridinea sensu stricto and Myzozoa being holophyletic; it also shows halvarian and chromist plastids both as robust clades nested within red algae. Not only do most dinoflagellates and Apicomplexa have plastids, whether phototrophs or heterotrophs, but so do the parasitic invariably heterotrophic Perkinsea (Fernandez Robledo et al. 2011). *Perkinsus* has nuclear genes with bipartite targeting sequences for plastids for plant-type ferredoxin and its reductase (Stelter et al. 2007) and for isoprenoid biosynthesis (Matsuzaki et al. 2008); though its growth is inhibited by thiostrepton thought to be specific for plastid ribosomes (Teles-Grilo et al. 2007), there is no evidence for plastid DNA. A possible plastid bounded perhaps by four membranes is present apically, but I am not convinced that the multimembrane structures with two to four membranes seen in cell fractions are plastids (Teles-Grilo et al. 2007). An organelle with two or three membranes (none seen with four) in *Parvilucifera infectans* might be a plastid (Norén et al.’s 1999 Fig. 16), as might the unidentified organelle in *Parvilucifera prorocentri* with at least two membranes and dense matrix (Leander and Hoppenrath 2008). If the PPM was lost in the ancestral dinozoan, I would expect Perkinsozoa and other heterotrophic Myzozoa to have plastids with three membranes, but if lost only in the ancestral dinoflagellate, four as in apicoplasts. The presence of two types of targeting sequences in dinokaryote dinoflagellates uniquely amongst chromists (Patron et al. 2005) could be a consequence of PPM loss and/or the fact that their plastids are not inside rough ER but probably require a Golgi-dependent vesicle-targeting step (see below). As *Oxyrrhis* also has two targeting sequence types (Slamovits and Keeling 2008), its membrane topology and plastid targeting mechanisms are likely the same as dinokaryotes; if these are shared by all Dinozoa, their unique membrane topology originated immediately after they diverged from Apicomplexa. If plastid minicircles also evolved then, as they encode only photosynthesis-related proteins all Dinozoa should lose plastid DNA when photosynthesis is lost.

It is now beyond reasonable doubt that the last common ancestor of Myzozoa and Ochrophytina (the halvarian ancestor) was a phagotrophic chromophyte alga, Ciliophora and Bigyra having lost plastids very early in halvarian evolution (Fig. 1). Present evidence best fits the last common ancestor of all chromists having been a biciliate phagotrophic chromophyte alga with cortical alveoli, extrusomes, ventral feeding groove, and cytoskeleton distinct from all other eukaryote kingdoms. Differential loss, modifications, and lineage-specific innovations could readily have made all other chromist phenotypes, as later sections explain.

One argument against ancestral chromists being photosynthetic concerns examples in chromists of metabolic redundancy arising from chloroplast symbiogenesis followed by a differential loss of host and symbiont enzymes that imply widespread selection for simplifying duplicated pathways (Waller et al. 2016). One would therefore expect such differential sorting of duplicates to take place relatively soon after plastids were gained as it seems unlikely that duplicate genes would be retained for many scores or hundreds of millions of years and then undergo sorting immensely later than their origins. The examples cited by Waller et al. for alveolates therefore suggest either (1) that divergence of Dinozoa and Apicomplexa was relatively close to the divergence of Myzozoa from Ciliophora and that of alveolates from the ancestral chromist or (2) that if these divergences were relatively late in chromist evolution, it is likely that myzozoan plastids came by tertiary transfer from euchromists. Waller et al. assume that these divergences are relatively late by reference to hypothetical Fig. 2 that incorrectly shows Hacrobia as polyphyletic and grossly distorts the apparent temporal scale of chromist evolution. That diagram portrays Cryptista and Haptista lineages both as about three times as old as Myzozoa, for which there is not a scrap of evidence. Waller et al. wrote ‘Maintenance of redundant pathways *through all of this time* [my italics] is difficult to reconcile with the rapid losses of different elements of this redundancy evident since apicomplexans and dinoflagellates radiated’. Not so, if you accept my long-standing argument that radiation of these groups was extremely rapid (Cavalier-Smith 1982). Because they make the erroneous assumption that Myzozoa diverged late from Ciliophora compared with the date of the chromist’s last common ancestor, Waller et al. reach the mistaken conclusion that their argument favours a heterotrophic ancestor and tertiary plastid acquisition. It does not, because they made no effort to estimate relative divergence times, which are crucial to their interpretation, allowing themselves to be seduced by a temporally grossly misleading diagram into reaching the wrong conclusion. In fact, sequence trees show hacrobian branches as markedly shorter than myzozoan ones, presumably through accelerated evolution in the latter. Multiprotein trees show that the time elapsed since the alveolate ancestor and the primary myzozoan divergence is a relatively small fraction of alveolate history; correcting for the likelihood of accelerating stems would allow the divergence to be very soon indeed after the origin of chromists (Cavalier-Smith et al. 2015a), so we need to postulate only a relatively *short* period of retention of both versions of each pathway before differential sorting. As Cavalier-Smith et al. (2015a) explain in great detail, the difficulty of resolving corticate branching order on multigene trees implies that all four major chromist and all three major plant groups diverged almost simultaneously, in accordance with my long-standing thesis (Cavalier-Smith 1982).

Erroneous assumptions about relative timing of events underlie all the other papers Waller et al. cited favouring late tertiary transfers (their other serious flaws are discussed below). Their ingenious gene redundancy argument is not a reason for doubting chromist photosynthetic ancestry, but instead rather strong evidence for my repeated arguments for an *extremely rapid evolutionary divergence* of all four chromist groups immediately after their last common ancestor enslaved a red alga by evolving a novel protein-targeting machinery, whose unity is much stronger evidence for chromist unity than Waller et al. had realised.

## The single secondary symbiogenetic origin of algal chromists

As Fig. 3 shows pictorially, membrane topology in chromist algal cells is far more complex than that in plants. In Plantae, chloroplasts are invariably free in the cytosol like mitochondria, whereas in chromists, they are located within the ER. Therefore, all nuclear-coded proteins that function within chromist chloroplasts must, during synthesis, be moved across the ER membrane; in all chromophytes but dinoflagellates, they must also cross the PPM, the former plasma membrane of the enslaved red alga. As I predicted when first discussing chloroplast protein-targeting evolution (Cavalier-Smith 1982), all four chromist lineages with chloroplasts of red algal origin share the same trans-PPM protein-targeting machinery with a single evolutionary origin: their nuclear-coded plastid proteins all have bipartite N-terminal topogenic sequences that are removed by specific peptidases during their two-stage translocation; even the non-photosynthetic malaria parasites (*Plasmodium*) retain ~ 400 such proteins. After protein synthesis starts on cytosolic ribosomes, the N-terminal signal sequences are recognised by SRPs that attach them to rough ER membranes, across which they are then cotranslationally extruded into the ER lumen (He et al. 2001; Waller et al. 2000), where signal peptidase cleaves off the signal sequence (van Dooren et al. 2002). The originally subterminal transit peptide (TP), thereby exposed terminally, is subsequently recognised by a chromist-wide ubiquitin-dependent machinery for translocation across the PPM (Agrawal et al. 2013; Bullmann et al. 2010; Kalanon et al. 2009; Maier et al. 2015; Spork et al. 2009; Stork et al. 2013). In euchromists (Hacrobia, Ochrophytina), this can happen immediately as chloroplasts are inside the rough ER membrane that is continuous with the outer nuclear envelope membrane. Thus, the euchromist PPM and enclosed chloroplast(s) are topologically within the lumen of the nuclear envelope as Whatley et al. (1979) first argued (Fig. 3b). That is true even for the very few ochrophytes where no ribosomes are evident on the outermost membrane around chloroplasts: for example, ultrathin serial sectioning showed that the apparently smooth outermost membrane of *Heterosigma* is connected by slender tubuli to the ribosome-bearing nuclear envelope outer membrane, so the lumen around its PPM is topologically continuous with that of the perinuclear cisterna; proteins would be free to diffuse within this aqueous space without having to cross a lipid membrane (Ishida et al. 2000).

By contrast, in no Myzozoa does the outermost membrane (epiplastid membrane; Cavalier-Smith 2003a) bear ribosomes or ever exhibit continuity with the nuclear envelope or other rough ER. Instead in Apicomplexa (e.g. *Plasmodium*, *Toxoplasma*) and dinoflagellates (e.g. *Gonyaulax*), plastid-targeted proteins pass first into the rough ER and then by vesicular transport to the Golgi (Heiny et al. 2014) from where vesicles carry them to the apicoplast or dinozoan plastid and transfer them across the epiplastid membrane (EpM) by vesicle fusion as chromalveolate theory suggested (Cavalier-Smith 1999). A key innovation for myzozoan plastid targeting must have been a novel Golgi sorting receptor for TPs able to divert thus-tagged proteins into EpM-targeted vesicles (Fig. 3c); another would have been EpM-specific receptor proteins (presumably specific SNARES, a pseudoacronym for Soluble N-ethylmaleimide-sensitive Attachment factor protein REceptor). Without both innovations, most chloroplast proteins would not get to plastids but be secreted outside the cell, as happens if *Toxoplasma* TPs are deleted (Waller et al. 1998). These innovations cannot have been as difficult as one might imagine, for comparable Golgi-routed chloroplast import machinery evolved convergently in two other independent secondary symbiogeneses involving green, not red algae (i.e. Chlorarachnida and Euglenophyceae; Cavalier-Smith 2013a). Their evolution would have been facilitated by the EpM having evolved from the original perialgal vacuole that arose by phagocytosis from the plasma membrane, so it would initially have had all requisite receptors for receiving exocytotic vesicles. Consequently at the outset, as soon as genes for chloroplast-targeted proteins were duplicated in the host nucleus and acquired N-terminal signal sequences, their encoded proteins would automatically have been transported indiscriminately to both the perialgal vacuole and cell surface. Selection against wasteful surface secretion would have made chloroplast targeting more specific by better differentiating the EpM and vesicles carrying TP-tagged proteins. Novel EpM proteins helped recognise specific vesicles bearing TP receptors and mediated small molecule exchange across EpM to ensure their metabolic integration with the cytosol.

Apicoplast EpMs have essential phosphate translocators that counterexchange inorganic phosphate and phosphorylated metabolites (Lim et al. 2016). These and other EpM proteins lack bipartite topogenic sequences but have an internal membrane anchor—a recessed hydrophobic patch that binds them to EpM-targeted vesicles (Lim et al. 2016). Thus, EpM targeting of TP-labelled proteins could have started without a novel targeting machinery, but indiscriminately and wastefully, it would readily have gradually improved by evolving better Golgi vesicle sorting and more specific EpM fusion. What machineries evolved for this and for targeting soluble proteins located inside the EpM but outside the plastid (e.g. a thioredoxin that also lacks bipartite plastid targeting sequences) remain unknown, but labelling shows that thioredoxin-carrying, presumably EpM-targeted vesicles differ in size from exocytotic secretory vesicles (DeRocher et al. 2008).

The apparent relative ease of evolution of the early steps of Golgi-routed protein import pathway into secondary plastids makes it likely that myzozoan membrane topology was the ancestral one for chromists (like that in Fig. 3c with PPM/PR except in the ancestor the nucleomorph would still have been present), as for the other examples of secondary symbiogenesis, and that plastid location within the rough ER in Ochrophytina and Hacrobia (Fig. 3b) was secondary. Accidental but permanent fusion of EpM with the nuclear envelope’s outer membrane would have placed the chloroplast and its PPM inside the lumen of the perinuclear cisterna (Whatley et al. 1979), completely bypassing the Golgi route in a single step, without any new molecular machinery for targeting having to evolve (Cavalier-Smith 1982, 1986, 1999, 2000a). I originally assumed fusion happened once only in a common ancestor of Ochrophytina and Hacrobia, but multigene trees show they are not sisters, so fusion must have occurred independently in ancestral Hacrobia and Heterokonta (Fig. 1) after some EpM differentiation. Though fusion evidently occurred twice (less often than Whatley et al. 1979 assumed), it was most likely very early in each group, possibly before EpM targeting was as efficient as in modern Myzozoa but after TP targeting across the PPM evolved. If so, membrane fusion would immediately have made protein targeting more efficient as chloroplast preproteins would now directly enter the ER lumen without vesicular transport; most would immediately bind to the already efficient PPM TP receptor, very few passing onwards to the Golgi with loss to the cell surface. Assuming fusion was accidental, it did not need evolution of any novel proteins, so two independent fusions are not improbable. Their main consequence would have been markedly better targeting efficiency to the chloroplast, removing the selective advantage of Golgi TP sorting—thereby causing loss of Golgi TP receptors and EpM SNARE system, saving energy and nutrients. Membrane fusion relocating plastids into the ER lumen so as to bypass the Golgi (euchromists) or improving Golgi sorting specificity (Myzozoa) can be regarded as alternative ways of improving the inevitably initially imprecise targeting across the EpM.

Gould et al. (2015) questioned the simple membrane fusion theory just summarised and proposed instead a far more complex one, whose defects a later section explains. They do however accept, like everyone who has carefully considered the protein-targeting evidence (e.g. Keeling 2009; Maier et al. 2015), that just one red algal secondary enslavement yielded all chromophyte chloroplasts and (like me) regard surprisingly widespread scepticism as to the photosynthetic character of the ancestral chromists as unwarranted and arising from overemphasising poorly resolved contradictory sequence trees and/or seriously underestimating the ease of plastid loss early in chromist diversification. As Cavalier-Smith et al. (2015a) explained in detail, there is no need to invoke multiple tertiary chloroplast transfers within Chromista to explain their remarkable mixture of photosynthetic and heterotrophic lineages (Fig. 1) or earlier apparent conflicts in multigene trees; Occam’s razor should erase them. One ancestral secondary enslavement of a red alga, followed by multiple early plastid losses and two secondary acquisitions of green algal plastids (by chlorarachnid Rhizaria and the peridinean dinoflagellate *Lepidodinium*) and one tertiary transfer of haptophyte chloroplasts to a different peridinean lineage (subclass Karlodinia), is sufficient to explain this (Cavalier-Smith 2013a). Gould et al. (2015) also postulate without discussion that ancestral chromists had the cryptophyte membrane topology (PPM, nucleomorph, and plastid inside rough ER) and assume that the mechanistically more complex vesicle transport of plastid precursors in Myzozoa is secondarily derived. They appear not to appreciate the extreme evolutionary difficulties of this heterodox assumption as to evolutionary polarity, as I will explain after discussing the origin of protein transfer into the PS, the most difficult evolutionary step in chromist origin.

## Periplastid membrane functions in chromist biology

When first uniting alveolates and euchromists under the temporary name chromalveolates and arguing that their common ancestor arose by a single intracellular enslavement of a red alga (Cavalier-Smith, 1999), I discussed trans-PPM protein-targeting origin in more detail than before (Cavalier-Smith 1986). I argued against the classical theory of Gibbs (1981b) involving vesicular transport, rejecting her specific protein import model (Gibbs 1979) because it implausibly assumed periplastid vesicle fusion with the chloroplast envelope OM, which would have bypassed the standard Toc75 OM translocon through which all nuclear-coded stromal and thylakoid proteins are imported in plants, and predicted that to be true also for chromists (Cavalier-Smith 1999). Toc75 translocons were eventually identified in diatoms (Bullmann et al. 2010); diatom and apicoplast homologues proved to be essential for chloroplast import, acting after transfer across the PPM (Sheiner et al. 2015) as I predicted. I argued that protein import most likely depended on a PPM translocon and postulated that (a) a TP receptor and preexisting translocon became inserted into the PPM from elsewhere in the cell and (b) a preexisting ATP-dependent chaperone in the periplastid space (PS) provided the motive force for pulling newly made proteins across the PPM. This dual proposal argued that a subterminal TP provided all topogenic information for crossing the PPM and the double chloroplast envelope, now known to be correct, and that in dinoflagellates, the PPM and this machinery were lost after they diverged from Apicomplexa (with four membranes separating cytosol and plastid stroma).

Chaal and Green (2005) removed the N-terminal signal peptide from the bipartite topogenic sequence of nuclear-coded PsbO of the heterokont raphidophyte *Heterosigma akashiwo* and of the dinoflagellate *Heterocapsa triquetra* and found that their originally subterminal TPs function perfectly as TPs; like the TP of the red alga *Porphyra yezoensis*, they mediate import into isolated pea chloroplasts. Thus, the subterminal chromist sequence is undoubtedly a genuine TP, not just *TP like* as is sometimes said. They also found a *Heterosigma* stromal transit peptidase that cleaved TP at a single site, unsurprisingly with different specificities to those of flowering plants: red algal and most chromist TPs have a conserved phenylalanine next to the cleavage site absent in green plants (Stork et al. 2013). Apicoplast stromal transit peptidases are targeted by a bipartite sequence (Sheiner and Striepen 2014). The corresponding part of bipartitely tagged PS proteins is properly called TP like (TPL) as it differs from TP in lacking the phenylalanine, this in diatoms at least being sufficient to ensure retention in PS (Stork et al. 2013). Thus, the present evidence strongly supports two key ideas: TPs mediate transport across both the PPM and plastid envelope and TPLs are evolutionarily related to TPs and cross the PPM only using a shared machinery (Cavalier-Smith 1999).

Though the nature of the PPM transit peptide receptor remains unknown, protein import into the chromist PS involves (1) the transmembrane protein derlin (postulated to be a universal translocon) and (2) a ubiquitin-dependent PS chaperone motor (Cdc48p), both identified as essential for importing plastid and PS proteins (Maier et al. 2015). In diatoms at least, derlin also helps discriminate between TP- and TPL-tagged proteins after they enter PS by more strongly binding TPL proteins, unbound TP proteins being free to cross the plastid envelope (Stork et al. 2013); TPLs are somehow then removed. There is also evidence from Hsp70 binding sites in the *Plasmodium* TP that (as suggested; Cavalier-Smith 2003a) Hsp70 chaperone may also be involved in import (Gould et al. 2006; Sommer et al. 2007), though it might act not in the PS but in the plastid stroma as the same TP must mediate and be subject to selection for transport into both compartments. However, in diatoms, periplastid Hsp70 TPL targets green fluorescent protein (GFP) to the PR region of the periplastid compartment, not to the chloroplast as does the slightly chloroplast-specific TP of a light-harvesting complex protein (Gould et al. 2006).

Functions of the PPM are not restricted to protein import. They must include also bidirectional lipid and metabolite transfer and division. The PPM has to grow by lipid and protein insertion, but nothing is known directly of its lipid composition or where its lipids are made. I previously proposed that PPM lipids are made in the PR of heterokonts/haptophytes or nucleomorph membrane of cryptophytes and move to the PPM by vesicular transport (Cavalier-Smith 2003a). I still envisage a role for vesicular transport in PPM growth (Cavalier-Smith 1999, 2003a) but think it was premature to rule out a role in protein import also—not as Gibbs (1979) imagined across the PPM and plastid envelope, but across the PPM only. A later section argues that identifying the ubiquitin-dependent derlin-related translocon has not made vesicular transport irrelevant to protein import, as was widely assumed.

Periplastid versions of glycerol-phosphate acyltransferase and other glycerolipid synthetic enzymes with inferred bipartite targeting sequences strongly support my prediction of periplastid acylglycerolipid synthesis. Diatoms have a periplastid-specific lipid transfer protein (sSec14) (Moog et al. 2011) that I suggest is involved in such transport and may also transfer PC to the chloroplast OM (an essential function as all chloroplast envelope OMs have PC in their outer lipid leaflet (Botella et al. 2017) that replaced cyanobacterial lipopolysaccharide when chloroplasts originated; Cavalier-Smith 2000a). Sec14 mediates the transfer of PC and phosphatidylinositol between membranes and is essential for the vesicular transport between *trans*-Golgi and endosomal membranes (Curwin et al. 2009) and for the secretion of lipid raft proteins to the plasma membrane (Curwin et al. 2013) and cholesterol transfer, so its discovery in the diatom PS partially corroborates my theory. These key periplastid features emphasise that even the highly reduced heterokont PS is a relict cytosol—not part of the chloroplast. The apicoplast is not a *complex plastid* (the somewhat misleading term ‘complex chloroplast’ was apparently introduced by Whatley (1989)) but a triple chimaera of a plastid, relict symbiont cytoplasm, and host plasma membrane-derived perialgal vacuole.

Diatom PPMs have a triosephosphate translocator different from that of ER and the chloroplast envelope inner membrane (Moog et al. 2015). Other chromists have homologues of all these translocators, but their intracellular locations are largely unstudied. Diatom ER and PPM translocators both came from the red algal symbiont so acquired signal sequences for retargeting via ER after their genes entered the host nucleus. Interestingly, the PPM translocator also has a predicted TPL (much shorter than TPs of the chloroplast envelope translocators; Moog et al. 2015), suggesting it crosses the PPM before inserting into it from the PS. That would conserve its polarity compared with an ancestral chromist that may have inserted the PPM translocator direct from the PS like Der1 in modern cryptophytes. Two evolutionarily divergent triosephosphate translocators are present in the inner chloroplast envelope. None is known for the chloroplast OM; it should not need any as its porin-like β-barrel proteins should be permeable to such small molecules.

Moog et al. (2015) argue that triosephosphate translocators diversified after the PPM protein import translocon evolved. That is reasonable as the original photosynthates used by the host when symbiosis started were probably unphosphorylated sugars. Green algal endosymbionts are thought to provide their hosts primarily with the disaccharide maltose, whereas dinoflagellates provide corals with glucose, glycerol, organic acids, and lipids (Venn et al. 2008). Unfortunately, it is unknown what metabolites’ symbiotic red algae donate to their foraminiferan hosts, though glycerol and galactose are the main sugars they produce (Kremer et al. 1980). The actual sugar used by the enslaved red alga, however, does not affect the key evolutionary principle that, in the numerous symbioses between eukaryotic algae and phagotrophic hosts, both partners are already well set up to exchange nutrients to their mutual benefit without any genetic integration between them or evolution of novel proteins or protein-targeting machinery. The chromist host therefore likely enslaved not a purely incidental prey item but a red alga with which it had a long history of intracellular symbiosis. Unlike random prey, an established symbiosis is preadapted for later, more difficult symbiogenesis by a combination of symbiont genome reduction and insertion of host-encoded proteins (whether originally of host or symbiont origin) by new translocons after symbiont-to-nucleus transfer of gene duplicates (Cavalier-Smith 2013a). The future PPM would therefore already have had the capacity for appropriate nutrient exchange when still a red algal plasma membrane well before host-encoded proteins were inserted.

It also had a division mechanism that may have been inherited by modern PPMs. One likely component of this was dynamin GTPase that catalyses the last scission step in eukaryotic membrane division. Unsurprisingly, diatoms have a periplastid dynamin of the subfamily responsible for plastid division (sDrp; Moog et al. 2011). Alveolates have a different alveolate-specific dynamin paralogue responsible for apicoplast division (van Dooren et al. 2009).

Unfortunately, recent discussions largely ignore evidence for a smooth PR within the PS (Gould et al. 2015; Maier et al. 2015). This oversimplification of periplastid biology severely limits current theories, which have insufficiently precisely defined the position of the ubiquitin-dependent translocon or the exact topology of the PR, and overlooks the apparent ubiquity of periplastid vesicles that I assumed transfer lipid from PR to PPM (Cavalier-Smith 2003a), but which Gibbs (1979) thought were involved in protein import—why not both? As noted above, my new evolutionary synthesis of molecular and ultrastructural evidence led me to a new integrated explanation for protein import in which periplastid vesicle cycling between PR and PPM and a ubiquitin-dependent translocon are both essential, with complementary sequential roles. Before explaining it, I summarise the existing non-vesicular model for protein import and then show that the PR is probably ubiquitous in chromists and more important than is generally assumed.

## Evolution of ubiquitin-dependent protein transport into the PS: the standard model

**Fig. 4:**
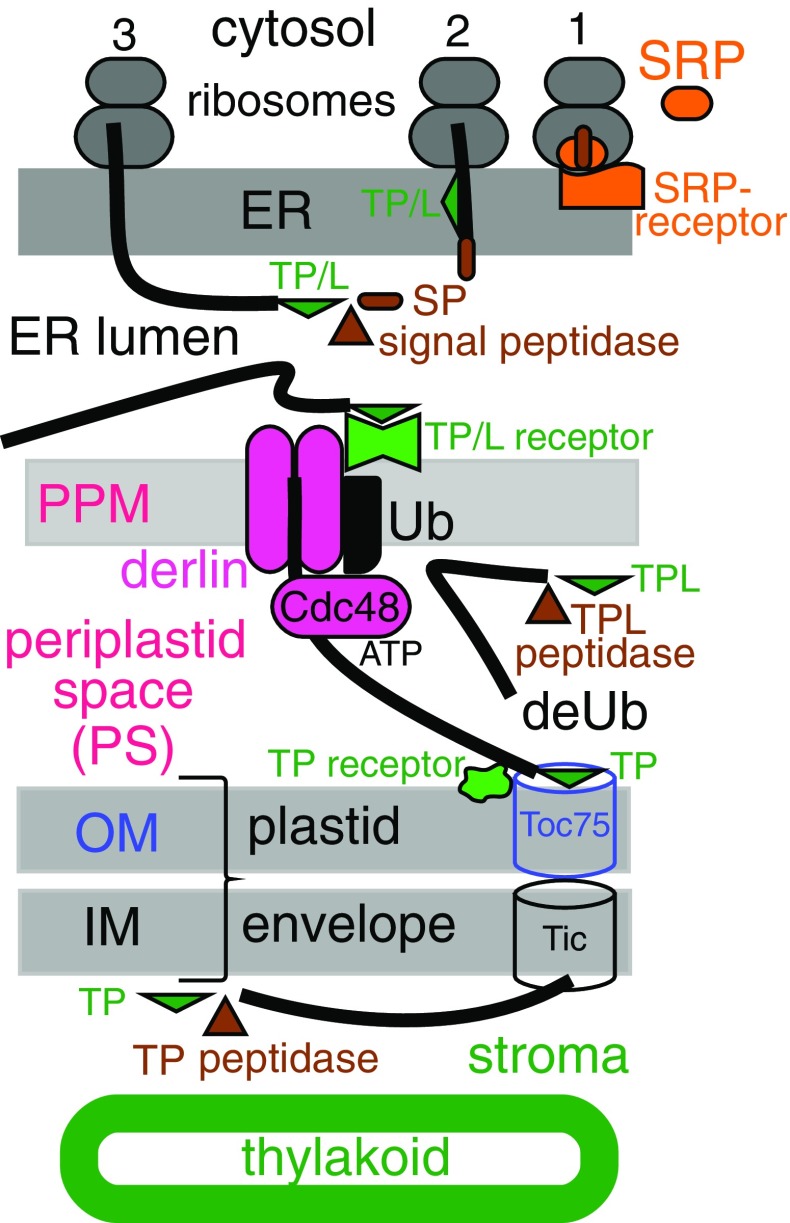
The standard model for import of nuclear-coded proteins into the chromist periplastid space (*PS*) and plastids, ignoring a possible role for the periplastid reticulum (*PR*). Ribosomes are shown in successive stages of protein synthesis and translocation (*1*–*3*). Nascent imported proteins (*thick black line*) have an N-terminal signal peptide (*SP*, *brown oblong*) that projects from the large ribosomal subunit and is recognised by a signal recognition particle (*SRP*) that then binds to an ER SRP receptor, ensuring that the ribosome attaches to the ER and extrudes the whole protein through an ER-embedded Sec14 channel (not shown) into the ER lumen. An ER lumenal signal peptidase cleaves off SP, exposing subterminal TP/TPL (*green triangle*) which is recognised by still unidentified dual purpose TP/TPL receptors (*green*) on the PPM and transferred to a membrane-embedded derlin oligomer essential for transfer to the PS (this stage only is more complex if PR is involved: Fig. 5). Derlin-mediated translocation into the PS depends on preprotein ubiquitinylation by ubiquitinating enzymes (*Ub* within PPM plus PS cofactors) and a PS-specific ubiquitin (*pUb*). pUb-tagged proteins are recognised by a PS-located Cdc48 ATPase, which (helped by cofactors) actively pulls them into the PS where ubiquitin is removed by deubiquitinating enzymes (*deUb*). Proteins that function within PS (e.g. Cdc48, deubiquitinating enzymes, TPL peptidase in all chromists, starch-making enzymes in cryptophytes, proteasomal proteins in cryptophytes, and heterokonts) have their TPL removed by a TPL peptidase, as must nuclear-coded NM proteins like DNA polymerases in cryptophytes. Imported proteins with a TP rather than TPL pass onwards into the plastid stroma through the standard Toc/Tic plastid envelope outer membrane (*OM*) and inner membrane (*IM*) channels, TP being removed later in the stroma by a different TP peptidase. Nuclear-coded intrathylakoid proteins often have tripartite N-terminal topogenic sequences with a second SP downstream of TP for transport across the thylakoid membrane using stromal insertion machinery

My original and more detailed discussions of PPM protein-targeting origins (Cavalier-Smith 1999, 2003a) both suggested for simplicity that a copy of the plastid OM protein translocator Toc inserted into the PPM and became greatly modified through having novel interactors to a new translocator Top. That this was too simple an explanation of PPM translocation became apparent after sequencing the first cryptomonad NM genome (Douglas et al. 2001) led to the discovery in *Guillardia theta* of four NM-encoded components of the ubiquitin-dependent ER protein extrusion machinery apparently located in the PPM or PS as they lacked TPs (Sommer et al. 2007). Sommer et al. (2007) showed that three of these were also present with bipartite targeting sequences in apicomplexan genomes and postulated that in all chromists with four chloroplast-bounding membranes, an ER-derived ubiquitin-labelled extrusion apparatus of red algal origin could provide the hypothetical trans-PPM translocon and motive force. Much laborious work has shown several aspects of this bold idea to be correct, and all chromists with red alga-derived plastids (except of course dinoflagellates that lost the PPM) have a comparable set of proteins, irrespective of whether their plastids are inside the rough ER or as in Myzozoa within a smooth EpM (reviewed by Maier et al. 2015; Stork et al. 2013; here, I highlight only features of key evolutionary importance; Fig. 4).

In the heterokont diatoms, their periplastid location has been well established by protein-specific fluorescence labelling, additional periplastid proteins have been discovered, and the association of several of them in a large macromolecular complex has been demonstrated (Hempel et al. 2010; Lau et al. 2016; Maier et al. 2015). In sporozoan apicomplexans, where gene knockouts are possible, preprotein ubiquitination and the putative PPM channel protein derlin (found in the periplastid compartment of all chromists with a red algal PPM) proved to be essential for importing proteins into the apicoplast and for cell viability (Agrawal et al. 2009, 2013). Derlin is related to intramembrane rhomboid proteases, both having six transmembrane helices (Lemberg and Adrain 2016; Vinothkumar 2011). The periplastid macromolecular complex includes also a rhomboid protease in heterokonts, haptophytes, and cryptophytes (whether any of the apicomplexan rhomboid proteases are in the apicoplast is unclear; Lau et al. 2016); I suggest its role might be to cleave mistargeted or misfolded proteins that get stuck in and block the import channel, releasing them for proteasomal degradation.

Periplastid-specific versions of these proteins are all absent in cercozoan chlorarachnids (e.g. *Bigelowiella*) that long after ancestral Rhizaria arguably lost the red algal chloroplast (Fig. 1), enslaved a green alga instead. Therefore, secondary symbiogenesis does not necessarily require recruitment of algal derlin and a ubiquitin system as a protein import translocon; the chlorarachnid PPM must use a different translocon (conceivably Top as originally proposed; Cavalier-Smith 2003a). This emphasises the uniqueness of the chromist PPM of red algal origin, making its ubiquity very strong evidence for monophyly of all chromophytes [including those like the apicomplexan chromeroids and heterokont Eustigmatophyceae (and a single xanthophyte, *Xanthonema debole*; Gardian et al. 2011) that secondarily lost chlorophyll *c* but may still be regarded as chromophytes sensu lato as their chloroplasts share a common origin]. Unless mentioned otherwise, for brevity, the rest of this paper uses ‘PS’ and ‘PPM’ to refer only to chromists with PPMs of red algal origin, thus excluding those of chlorarachnids whose PPM evolved instead from a green algal PM (Cavalier-Smith 2003a, 2013a). This paper does not use the acronym SELMA (symbiont-specific endoplasmic reticulum-associated-like machinery; Hempel et al. 2009) for the derlin import complex as SELMA is less informative to non-specialists. I also did not want to overemphasise its ER location as derlin is not specific to ER, being also located in endosomes in non-chromists at least (Schaheen et al. 2009).

Universally present in the PS are nuclear-coded AAA ubiquitin-dependent ATPases, Cdc48p, that provide the motive force for pulling preproteins through the membrane in association with derlins and ubiquitinating enzymes (Fig. 4). Like all other eukaryotes, chromophytes also have cytosolic versions of all three proteins that mediate extrusion of misfolded proteins (predominantly soluble, some membrane) from the ER and pass them to proteasomes where they are deubiquitinated and hydrolysed to amino acids. Periplastid versions of Cdc48p are strongly more related to those of red algae than to chromist cytosol or green algal versions, making them highly likely of red algal origin (Petersen et al. 2014). Periplastid derlins are marginally closer to red algae than to greens, as expected from their being encoded by cryptophyte NMs, so both key proteins probably came from red algae (Petersen et al. 2014; see also my more comprehensive trees in the next section) as the Omp85-related Toc channel of diatoms (presumably therefore other chromists also) very obviously did (Bullmann et al. 2010). Derlins are integral membrane proteins with transmembrane helices (once thought to be four, but actually six like their rhomboid protease relatives; Urban and Dickey 2011; Lemberg and Adrain 2016), and (in ER versions at least) both N-terminal and C-terminal on the cytosolic (PS in PPM), not ER side. Chromophytes also have periplastid red algal versions of Ufd1 and Npl4, both cofactors for Cdc48p that help it associate with derlin and ubiquitinated substrates. After Cdc48p pulls preproteins into the PS, they are immediately deubiquitinated and ready for transfer through Toc75 channels into the chloroplast or for folding and functioning in the PS, as appropriate. Which they do depend on a key difference at the N-terminal end of their TP/TPL; plastid-destined TPs usually have an aromatic residue or a leucine as the first TP amino acid, especially a phenylalanine in cryptophytes and heterokonts, to differentiate them from PS proteins (Maier et al. 2015), but this difference is less marked in haptophytes and absent in Apicomplexa which simplified their periplastid functions, and probably have fewer PS proteins (see PR section below). This TP/TPL difference clearly arose during evolution of PPM-specific translocation, being identically pronounced in one major group in each chromist subkingdom.

Sommer et al. (2007) proposed that the key step was transfer of derlin from the red algal ER membrane to the PPM (its original PM). However, there are several unsolved problems and neglected aspects of periplastid function in this standard model, so not all its assumptions need be correct. In particular, diatom GFP labelling has low resolution compared with electron microscopy, so derlin’s precise location is uncertain: instead of in the PPM as generally assumed, it might be located and function for import in the PR which exists in *Phaeodactylum tricornutum* (Flori et al. 2016), the centric diatom that Maier’s group uses for studying targeting, and may be topologically distinct from the PPM (Gibbs 1979; Cavalier-Smith 2003a). The rest of this section discusses PPM evolution assuming the standard model (Sommer et al. 2007; Maier et al. 2015) (see Fig. 4A); later sections elaborate a previously overlooked alternative hypothesis that derlin is in the PR (see Fig. 3b) and that both vesicle transport and a translocon are essential for import.

Even if derlin was historically transferred to the PPM, it was not necessary to move Cdc48p or its cofactors also: they were already in the correct compartment (PS) for their present function. Even in modern cells, derlin is found both in ER membranes and in endosome membranes (Schaheen et al. 2009), showing that it must pass through the Golgi towards the cell surface; thus, transferring it to the ancestral PPM might have been relatively easy. Potentially, a slight leakage from endosomes into exocytotic vesicles, followed by exocytosis, could have put some derlins in the PM (PPM homologue) correctly oriented for importing proteins (the possibility that derlins are already in PMs is not excluded by animal cell labelling). To do that, derlin would have to become able to recognise them or associate with a TP receptor as originally postulated (Cavalier-Smith 1999).

The nature of TP receptors for preproteins crossing the PPM or plastid OM via Toc75 channels is unknown (Maier et al. 2015). That is unsurprising, because one expects standard Toc TP receptors (Toc159 and Toc34) to radically change when PPM targeting evolved (Cavalier-Smith 2003a). I suggest that both receptors were retained for the chloroplast envelope but changed so much that sequence bioinformatics can no longer detect them, though possibly they could have been dispensed with if the PPM translocon can pass preproteins directly to Toc75—but that direct route is unlikely as derlin/Cdc48p machinery must work equally for plastid and PS proteins. Even the more conservative Toc75 (cyanobacterial Omp85 homologue) was initially overlooked because of its divergence until discovered in diatoms (later in others); one expects TP receptors to have changed even more. I now suggest that of the three main Toc proteins, possibly only Toc159 was adapted for PPM import and thereby became differentiated as ‘Top159’ from OM Toc159. Plant Toc159 lacks a TP and is targeted by binding to Toc34 (which also lacks a TP and needs several proteins for its OM insertion). A Toc159 duplicate possibly lost Toc34 binding sites and acquired derlin binding sites instead, and after gene transfer to the nucleus, acquired a signal sequence for entering host ER. This would enable it to pass preproteins directly to derlin after derlin (still made in the PS, so without a signal or TP) relocated to PPM (if it did). Thereafter, TPL and TP for recognition by Top and Toc159 respectively diverged to enable more efficient differential targeting of PS and chloroplast proteins.

The ER-based derlin/Cdc48 translocation system is called ERAD (acronym for ER-associated degradation) because proteins extruded by it from the ER are passed to the cytosolic 18S proteasome cap for deubiquitination and subsequent digestion by 20S proteasomes. Losing this subsequent digestion, whilst retaining deubiquitination capacity, was an essential aspect of adapting ERAD for PS protein import (cryptophytes retain red algal PS proteasomes, which had to be prevented from digesting imported proteins in the ancestral chromist). Diatoms have a PS deubiquitinating enzyme PtDUP different from those of proteasomes that is postulated to deubiquitinate translocated preproteins (Hempel et al. 2010), which would prevent their digestion, but the situation is not straightforward as diatoms also have PS 20S proteasomes, though the 19S cap has been lost (Maier et al. 2015). However, yeast proteasomes have a deubiquitinase in the 20S proteasome as well as a different one in the cap, and capless 20S proteasomes deubiquitinate proteins without digesting them (Guterman and Glickman 2004); this might be true of diatoms also. Haptophytes and Apicomplexa have no PS proteasomes, so completely lost them and must use another deubiquitinase—numerous different ones exist in eukaryotes, so they need not have recruited the same ones as diatoms; this further supports the idea that haptophyte and apicomplexan PS are simplified compared with cryptophytes (the most complex) and heterokonts. A further conundrum is that though cryptophytes have 26S PS proteasomes, no PtDUP homologue was detected, so how do they avoid proteasomal digestion of translocated proteins? Might alternative solutions to this problem have been adopted by different early diverging chromists?

That is certainly true for initial ubiquitination. Cryptophytes have a NM-coded periplastid version of Hrd1, a large (500–600 amino acids) integral membrane protein with six transmembrane domains that is the standard ERAD ubiquitin ligase for proteins with misfolded lumenal or membrane domains. No Hrd1 homologues are known in other chromists, which must instead use different ubiquitin ligases (even yeast has multiple ones). That of diatoms (E3P, 537 amino acids in *Phaeodactylum*) has only one transmembrane helix which should make it easier to import across the ER membrane, and other chromists have similar ones (Hempel et al. 2010). Hempel et al. (2010) suggest that the six transmembrane helices of Hrd1 would make it harder to cross the ER membrane without getting stuck and that this may be why other chromists replaced it by one with fewer transmembrane segments when or before NMs were lost. That is the first plausible explanation of why cryptophytes alone retained NMs for ~ 750 My after chromists evolved (date estimate from Cavalier-Smith 2013b). They could only lose the NM if they could either import Hrd1 or replace it by another ubiquitin ligase that could successfully cross two membranes and be inserted into the PPM (or PS on my new view) periplastid face. From the most likely phylogeny (Fig. 1) NMs must have been lost independently by the harosan and haptophyte ancestors, so different ubiquitin kinases might have been recruited, though it would be simpler if the chromist ancestor adopted the same one. Possibly, it used two kinases, but the ancestor of cryptophytes lost the one with single-membrane helix and so were thereafter unable to lose NMs, whereas both other lineages lost Hrd1. It is important to note that even if Hrd1 could successfully cross ER membranes, it would almost certainly be inserted into the PPM from the opposite side in cryptophytes and so would have the wrong polarity to function in the same way.

That immediately raises the previously undiscussed question of how chromists that lost the NM were able to import derlin across the ER and insert it correctly into the PPM. Rather than assuming that PPM derlin has the opposite polarity in cryptophytes but can function equally well despite that, non-cryptophytes might import derlin not only across the ER membrane but also across the PPM and then reinsert it into the PPM (or PR, see below) from the PS. This would conserve polarity after gene transfer to the nucleus exactly as mitochondria did for many inner membrane proteins whose polarity had to be conserved for function (see Cavalier-Smith 1987c, 2006b). This explanation is plausible because despite derlin having six transmembrane helices, the ER version is short (~ 210 amino acids in chromists, roughly a third the size of Hrd1) and the added presequence in the periplastid version is long enough (111 amino acids in *Chromera*, 50–170 in the other chromists in Fig. 1) to encode both signal and TP and possibly stop helical regions from being trapped in any membrane and thus pass through both membranes. The fact that ER derlin lacks a cleavable signal sequence suggests it might be posttranslationally targeted; if so, its insertion mechanism would be conserved after gene transfer to the nucleus.

Though derlin is essential for ERAD in vivo, a proteoliposome-reconstituted system in which Hrd1 was the only transmembrane protein could bind and polyubiquitinate an unfolded protein substrate (helped by Ubc7 and Cue1) and itself, allowing reconstituted Cdc48 and its cofactor Ufd1 to pull the substrate out of the membrane powered by ATP hydrolysis (Stein et al. 2014). Hrd1 self-ubiquitination appeared more important than substrate ubiquitination; the authors suggested that repeated Hrd1 self-ubiquitination (causing stronger substrate binding) and deubiquitinisation by Otu1 would allow the substrate to slide relative to the hydrophobic Hrd1 helices that bind it. Other ERAD ubiquitinases like Doa10 with 14 transmembrane helices (even larger than Hrd1, thus not recruitable to non-cryptophyte PPM) could similarly act as a hydrophobic greasy pole down which substrates could slide out of the membrane; this seems a more plausible mechanism than the earlier idea of a hydrophobic pore for derlin also, as proteins of the rhomboid superfamily have a very compact 3D structure (Vinothkumar 2011), making an aqueous pore (originally proposed) unlikely as a translocon. Hrd1 oligomers are more effective at binding substrate than monomers but cannot bind all possible substrates; presumably, the greater complexity of actual derlin-containing ERAD complexes compared with this pared-down in vitro core system evolved to enable a broader spectrum of proteins to be extruded. In vivo in yeasts a receptor (Hrd3) is necessary to bind a substrate initially for passing it to adjacent derlin (Mehnert et al. 2015) which presumably passes it on to Hrd1; one must probably envisage a relay of protein-binding molecules that recognise and transfer proteins across the membrane for final extrusion by the Cdc48 complex and then deubiquitination. Single-membrane-helix ubiquitinases of non-cryptophytes may have to act as oligomers to be effective as channels for protein sliding. The overall pathway across the PPM might even vary with substrate as it certainly does for ERAD, and it is likely the actual translocon or channel is a complex of different oligomeric membrane proteins, not a single one. Nonetheless, for simplicity, I refer to the periplastid channel simply as ‘derlin’, whilst recognising that the precise nature of the translocon is controversial. I have now discovered that in chromists genes for a different red algal derlin paralogue moved into the nucleus, acquired PS targeting sequences, and replaced the old NM-coded derlin paralogue.

## Contrasting eukaryote-wide derlin paralogues: derlin A and derlin B and chromist monophyly

The yeast *Saccharomyces cerevisiae* has two very different derlin homologues, Der1 and Dfm1. Both self-associate as oligomers and bind cofactors, most but not all shared, and seemed not to interact with each other (Goder et al. 2008) but were later shown to be part of the same complex (Stolz et al. 2010). The three mammalian paralogues (derlin-1, derlin-2, and derlin-3) are mutually less divergent in sequence, and homo-oligomers form hetero-oligomers to a small degree and are also ER located (Lilley and Ploegh 2005). Previously, it was thought that most chromists have only one major periplastid derlin paralogue, though heterokonts have two which can form hetero-oligomers in a diatom (Hempel et al. 2009). As the derlin sequence phylogeny of Petersen et al. (2014) excluded yeasts and used only 105 amino acids (under half the coding sequence) and only maximum likelihood (ML), not an evolutionarily more realistic and often more accurate site-heterogeneous method (see Cavalier-Smith 2014a), I ran taxonomically more comprehensive sequence trees to clarify derlin history (both site-heterogeneous CAT trees and ML) and discovered that haptophytes also have two ancient periplastid paralogues. Supplementary Fig. S1 is for a more decisive site-heterogeneous analysis of 153 neokaryote derlins (over twice as many as before) and 201 well-aligned amino acid positions, not just 105.

It reveals that yeast Der1 and Dfm1 represent two contrasting ancient derlin paralogues (here called A and B); a taxonomically richer 193-sequence tree that also includes Eozoa (Supplementary Fig. S3) shows that A and B diverged before the last common ancestor of all eukaryotes, as do ML trees (Figs. S2, S4). Statistical support for the A/B dichotomy depends on algorithm and taxonomic sampling and was highest in 122-sequence trees that excluded some longer branches (0.9 and 52% in Figs. S5 and S6) and lowest for 203-eukaryote trees including numerous long-branch sequences (Figs. S7, S8), but the dichotomy was always found and is rather clear in the alignment. All eukaryote groups except the parasitic diplomonad metamonad *Giardia* have both paralogues A and B (Figs. S3, S4, S7, S8). I found only one in *Giardia*, probably A. Its distant relative *Trichomonas* has two extremely divergent paralogues that probably represent A and B (Figs. S7, S8): one like that of *Giardia* wrongly groups with ciliate A paralogues on site-heterogeneous trees (Fig. S7), which must be a long-branch artefact; the other correctly groups with podiate Bs but wrongly attracts the red algal B branch away from Plantae and the diatom ER B branch away from chromists (Figs. S3, S7). In general, paralogue B evolves somewhat faster than derlin A; bipartitions have somewhat lower support and well-established clades appear less consistently, so B may be more optional for *Giardia*. Suppression of Golgi stacks, thus simplifying the endomembrane system of fornicate metamonads (e.g. *Giardia* and other diplomonads), unlike their parabasalian sisters (e.g. *Trichomonas*) with hypertrophied Golgi stacks, might have allowed *Giardia* (and perhaps fornicates generally) to lose derlin B, but loss might have been later when eopharyngian fornicates (e.g. diplomonads) became gut parasites and lost phagocytosis. *Malawimonas*, glaucophytes, and Eolouka are the only groups on Fig. 2 not represented on my derlin trees, which provide the first good overall summary of derlin evolution and diversity. Some lineages, e.g. Ciliophora, budding yeasts, cryptophyte nucleomorph, and sporozoan apicoplast derlins, evolve much faster than others, making them harder to place especially in taxon-rich trees with other long branches.

Goder et al. (2008) asserted that ‘Dfm1p is actually more related to the mammalian derlins than Der1p itself’. Figs. S1-S8 and the alignment clearly disprove that statement based on an ill-sampled tree (only mammalian and two *Saccharomyces* sequences) using an appallingly poor algorithm (Lilley and Ploegh 2004); mammals like almost all eukaryotes have both Der1 and Dfm1 orthologues. Based on these trees and others for 82 animal derlins (not shown, but the full 259-sequence alignment is in supplementary material), I conclude that derlin-2 and derlin-3 arose by gene duplication of the formerly single paralogue A prior to the last common ancestor of jawed vertebrates (Gnathostomata) or in the first vertebrate (no data for Agnatha), not in mammals as Lilley and Ploegh (2004) assumed and are much more closely related to Der1 than to Dfm1; only gnathostome derlin-1 is a B paralogue related to Dfm1. However, my analysis shows that derlin/Der1 nomenclature is thoroughly confused; for example, labelling two mouse sequences (Der1 and Der1–2) studied by Kalanon et al. (2009) was confusing as both are B derlin homologues of yeast Der1 (their Der1 is a derlin-3 and Der1–2 a derlin-2 using *Homo* nomenclature; they did not find the mouse derlin-1/Dfm1 B homologue, as I have). Almost none of the ERAD Dfm1 homologues (whether ER or periplastid) except those of *Saccharomyces* are correctly identified as Dfm1 orthologues in either GenBank or the literature but are usually *entirely incorrectly* called Der1 or else (acceptably) derlins (the original name for all three human paralogues), making Der1 an almost entirely meaningless term outside yeast specialists.

To avoid this misleading and confusing general use of Der1 as a synonym for all derlins, I have introduced A (easily remembered as the only paralogue in the **a**nimal parasite *Gi* ***a*** *rdi* ***a***; it includes yeast Der1 and vertebrate derlin-2 and derlin-3) and B (including yeast Dfm1 and vertebrate derlin-1) for the archetypal paralogues and use derlin to include both. Despite consistent support for separate ancestral A and B paralogues, my eight trees indicate that basal branching order within each is not robust to taxon sampling or phylogenetic method; e.g. periplastid B derlins and ER chromist A derlins are each clades on only two trees; ER rhodophyte derlins are a clade six times and wrongly paraphyletic 10 times. Rapid early radiation causes these differences; see supplementary Fig. S2-S3 discussion.

The chromist host and its red algal symbiont would originally each have had at least one copy of both derlins A and B; the ancestral chromist must have had at least four different derlin paralogues, two of host and two of symbiont origin. This probably facilitated novel periplastid functions. Figs. S1-S8 also show for the first time that cryptophyte periplastid derlin is a Der1 (derlin A) orthologue whereas halvarian and haptophyte periplastid derlins are not but are Dfm1 orthologues, i.e. derlin B, and have wrongly been called Der1 in GenBank and published papers. Clearly therefore, halvarian and haptophyte periplastid derlins were recruited from a derlin B paralogue prior to a loss of the NM and its derlin A gene. In all trees, periplastid nuclear-coded derlins having bipartite leaders for import group with plant sequences rather than with chromist sequences lacking import signals, though whether specifically with red algae or with a mix of reds and greens varies with taxon sampling (support values are low); this suggests that imported periplastid paralogues evolved from red algal derlin B; therefore, no duplication of host derlins or symbiont derlin A was needed to evolve periplastid protein targeting. Nucleomorphs were probably lost very early after Harosa and Hacrobia diverged (Fig. 1) when the ancestral chromist chimaera still had red algal derlin paralogues A and B; after the cryptophyte lineage lost red algal derlin B, it was impossible for it to provide a periplastid-located derlin B to any other lineage by tertiary symbiogenesis. The fact that no non-cryptist periplastid derlins evolved from the NM-encoded derlin A disproves all hypotheses of late tertiary transfers supplying targeting machinery from cryptophytes to other chromist lineages (e.g. Petersen et al. 2014) and thus greatly increases the probability that all arose by vertical descent from the photosynthetic chromist’s last common ancestor.

My discovery that there are two early diverging periplastid paralogues in both heterokonts and haptophytes also independently supports vertical descent from one ancestral secondary symbiosis. It cannot be reconciled with *late* tertiary transfers of derlin machinery from cryptophytes to haptophytes or heterokonts or with *late* serial transfers from or to haptophytes or heterokonts as assumed by most tertiary symbiogenetic ideas (e.g. Baurain et al. 2010; Cavalier-Smith et al. 1994; Petersen et al. 2014; Sanchez-Puerta and Delwiche 2008); such ideas would expect recipient derlin lineages to be robustly nested within donor ones, which is not the case. One heterokont periplastid B paralogue groups robustly with the long-branch alveolate sequences as a strong halvarian clade, whereas the other groups weakly with one of the two haptophyte periplastid B paralogues (Fig. S1); on the most taxon-rich trees, the second haptophyte periplastid B paralogue groups by PhyloBayes CAT-GTR (site heterogeneous) with the haptophyte/heterokont clade (Figs. S1, S3, S7) and by ML with the halvarian clade (Figs. S2, S4, S8). With 122 derlins only (fewest long branches), all three periplastid B clades grouped together as one clade by both ML and PhyloBayes (Figs. S5, S6). The simplest interpretation is that in the ancestral chromist, red algal derlin B moved to the host nucleus and acquired a bipartite leader for retargeting derlin B into the periplastid compartment almost immediately after red algal derlin A (still NM coded) was recruited for periplastid protein import; if so, for a brief period, the ancestral chromist used both red algal derlins A and B in the periplastid compartment. It is also likely that the retargeted red algal derlin B immediately underwent gene duplication and heterokonts and haptophytes kept both duplicates after Harosa and Hacrobia diverged, making these subparalogues almost as old as chromists. By contrast, alveolates lost the B subparalogue kept by both heterokonts and haptophytes, whereas cryptophytes lost all periplastid derlin Bs. Red algal derlin A was lost with NMs (twice; Fig. 1). Gould et al. (2015) already noted it was simpler for red algal proteins to be recruited for periplastid import without duplication but did not realise that derlins were already duplicated in virtually all eukaryotes, including red algae. My discovery that NM and nuclear-coded derlins are different ancient paralogues offers a new perspective on early chromist periplastid evolution.

As noted above, a different ubiquitin ligase from Hrd1 used by cryptophytes must also have been retargeted to PS early prior to NM loss. The use of Hrd1 by cryptophyte derlin A agrees with the sole use of Hrd1 by its orthologue yeast Der1 (A); by contrast, Dfm1 (B) can use Hrd1 or Doa ubiquitin ligase or direct proteins to other degradation pathways (Stolz et al. 2010). Strong preference of derlin A for Hrd1 only, if true of other eukaryotes, especially red algae, might have biased the ancestral chromist against recruiting nuclear periplastid derlin A for periplastid retargeting as its preferred partner Hrd1 could not be retargeted; therefore, they used B paralogues with different retargetable interactors.

Retention of derlins A and B in almost all eukaryotes means their functions are not equivalent, as must also be true of gnathostome derlin-2 and derlin-3 (A) paralogues. In yeast, Der1 (A) is essential for soluble protein ERAD but not for membrane protein ERAD whereas those requiring Dfm (B) are all membrane proteins ( and freeman and Adrain 2016). Nematode derlin-1 (B) knockouts disrupt endocytosis and can be corrected by mammalian derlin-1 (B) or derlin-3 (A), but not derlin-2 (A) (Schaheen et al. 2009), proving both general long-term functional conservation between A and B and divergence within A; neither yeast A nor B could rescue B-mutant nematodes—unsurprisingly given their extreme sequence divergence (Figs. S1, S3). Mouse knockouts also reveal differences; derlin-3 is least essential and expressed in only some tissues (Lemberg and Adrain 2016). ERAD probably evolved before ER in the first stage of the origin of eukaryotes after the loss of the bacterial cell wall and the onset of extracellular digestion of prey: I suggested that plasma membrane derlin originated for importing prey proteins and their concerted digestion by proteasomes even before the origin of phagocytosis and the endomembrane system (Cavalier-Smith 2009a). Though I here emphasise evidence for endosomal as well as ER derlin, labelling of animal cells and mouse cell fractions suggests that even today, some derlin may also be present in the PM, but resolution is insufficient to be sure (Schaheen et al. 2009). Almost certainly, derlins evolved from integral membrane rhomboid proteases widely found in prokaryotes and retained by all eukaryotes, other subfamilies of which independently lost proteolytic function (Urban and Dickey 2011); rhomboid proteases were present in the ancestral eukaryote (Lemberg and Freeman 2007) and are found in Golgi, mitochondria, and one in ER with an ERAD function (Fleig et al. 2012). I now suggest that the duplication producing derlins A and B also happened in the earliest stage of eukaryote endomembrane evolution prior to its differentiation into ER and Golgi membranes.

Ancient paralogue trees like Fig. S1 can, *in theory*, be used for rooting; each paralogue subtree can be considered an outgroup to the other and should place the neokaryote root in the same place. Obviously, they do not: paralogue A puts the root within podiates (insignificant support) so Chromista, Plantae + NMs, and corticates all are clades, but podiates appear paraphyletic; contradictorily, paralogue B puts the root within chromist ER sequences and Plantae (plus derived periplastid sequences) are a clade but both corticate and chromist host derlin Bs appear paraphyletic; podiates would be a clade but for the statistically insignificant misplacement of long-branch Ciliophora. Multigene trees that also include Eozoa show corticates and podiates both as clades (Cavalier-Smith et al. 2015a), placing the neokaryote root within either is wrong—it should be between corticates and podiates on subtrees A and B. Clearly, both are misrooted, presumably because of long-branch attraction: chromist B paralogues have many long branches that may wrongly attract the A outgroup, and the podiate A paralogues include some especially long branches that probably wrongly attract the B outgroup. This exemplifies the fact that *in practice*, such contradictory artefacts make paralogue rooting generally an extremely bad way of trying to root sequence trees; it was severely criticised previously with respect to rooting the tree of life where artefacts are far worse but seldom appreciated (Cavalier-Smith 2002a, 2006a).

## Chromist periplastid reticulum ubiquity and functions

**Fig. 5:**
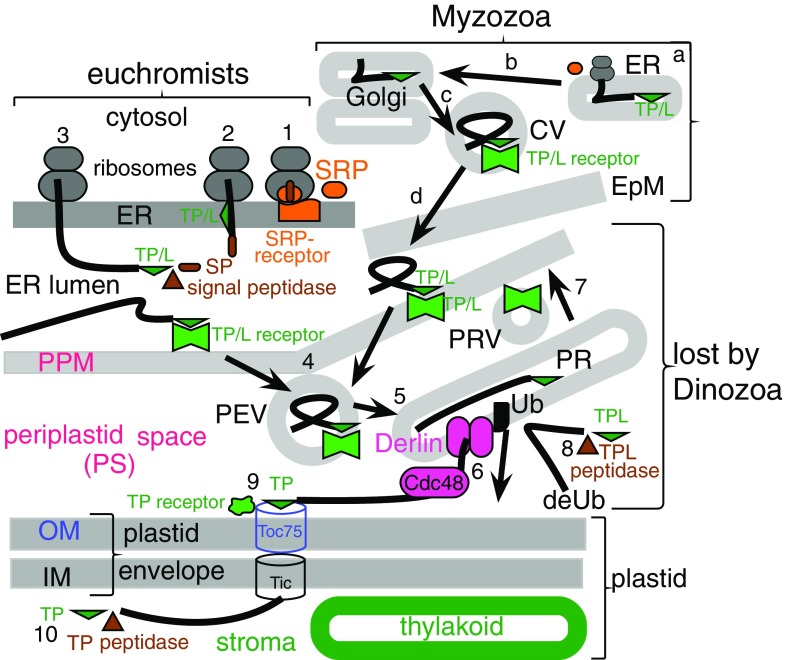
Possible role for periplastid reticulum (*PR*) in protein import to the chromist periplastid space (*PS*) and plastid. The essential difference from the standard model (Fig. 4) is that for preprotein extrusion into the PS, mature oligomeric derlin (A in cryptophytes, B in other chromists) and its associated ubiquitinating enzyme (*Ub*) are postulated to function not in the PPM (Fig. 4) but in the PR membrane where their assembly into the functional derlin/Ub macromolecular complex is postulated to occur. Individual derlin As and Ub are NM coded and made on periplastid ribosomes in cryptophytes and can enter PR by direct insertion from the PS. In other euchromists and Apicomplexa, derlin and Ub are nuclear coded (two distinct derlin B paralogues in heterokonts and haptophytes; one in apicomplexa) and postulated to be carried individually (like all other proteins with bipartite targeting sequences) from the ER lumen by TP/L receptor-mediated endocytic PPM budding (stage 4) to form periplastid endocytic vesicles (PEV) that fuse with PR (stage 5) to place PS- and plastid-destined preproteins into the PR lumen. TP/TPL-labelled preproteins put into the PR lumen by PEV fusion are extruded into the PS via the derlin/Ub complex powered by Cdc48 ATP hydrolysis (stage 6) and further processed (stages 8–9) exactly as in the standard model (Fig. 4). Separate PS transport vesicles (*PRVs*) are assumed to bud from the PR and fuse with the PPM, recycling TP/L receptors and lipids to the PPM (stage 7), including any lipids newly synthesised by the PR. Preprotein entry into the space outside the PPM differs in euchromists (cotranslational: *upper left*) and myzozoan alveolates [posttranslational: *upper right*: via fusion (*d*) of vesicles (CV) with the epiplastid membrane (*EpM*) that arose by TP/L receptor-mediated budding (*c*) from the Golgi, put there by vesicles budded (*b*) from the rough ER where preproteins entered its lumen cotranslationally (*a*)]. Dinozoa secondarily simplified plastid preprotein import by losing PPM, PR, and separate PS proteins, attaching EpM directly to OM

I argue here that the PR is a universal structure in chromists with a PPM, but usually topologically distinct from it and essential for protein import and lipid synthesis. I conjecture that it evolved from the *trans*-Golgi network (TGN, the primary endosomal compartment of plants; Paez Valencia et al. 2016) of the enslaved red alga and is the site of periplastid derlin and associated membrane proteins. Justifying this radically new interpretation of chromist cell biology and evolution (Fig. 5), with several advantages over the standard model, requires considerable detail.

PR was probably first seen by Hovasse and Joyon (1960) in the chrysophyte *Hydrurus*, but although an important part of my arguments for chromist unity and periplastid evolution (Cavalier-Smith 1986, 1989, 1999, 2003a), virtually all subsequent discussions of the subject totally ignored it. Only recently have electron microscopists started to restudy PR—under a superfluous, less informative new name, ‘vesicular network’ (Flori et al. 2016). PR was originally named ‘periplastidal reticulum’ by Falk and Kleinig (1968) who found it in the heterokont xanthophyte *Tribonema* between the plastid envelope and PPM (which they misinterpreted as smooth ER). That name in their abstract tried to anglicise the more complex German name in the main paper; later, I (Cavalier-Smith 2003a; Ishida et al. 2000) adopted a simpler more naturally English and euphonious spelling (periplastid reticulum) for conformity with my terms periplastid membrane (Cavalier-Smith 1989) and periplastid vesicles (Cavalier-Smith 1999)—contrary to Flori et al. (2016), its name was not invented by Gibbs (1979) who adopted Falk and Kleinig’s spelling, never using ‘periplastidial’, a later needlessly longer invention.

First, I will demonstrate PR universality in PPM-bearing chromists. Initially calling it just ‘tubules’, Gibbs (1962) found PR in three other chrysophytes (notably *Ochromonas*), in the brown alga *Pylaiella*, and an alga misidentified as *Olisthodiscus* but now known to be a *Heterosigma* (class Raphidomonadea). She mistakenly believed an organised PR is absent from cryptophytes but recognised that scattered smooth PS vesicles might have the same function. I think the vesicles between the nucleus and starch grains in Gibbs’ (1962) Figure 7 of the cryptophyte *Rhodomonas lens* are probably a typical sheet-like organised PR; the weakly stained vesicles/tubules near the NM of *Cryptomonas abbreviata* (Gibbs’ 1993 Fig. 2) are probably PR patches. I now argue that cryptomonads generally have patches or fenestrated cisternae of organised PR as well as scattered vesicles and regret uncritically repeating (Cavalier-Smith 2003a) her probably erroneous statements (Gibbs 1962, 1979) that cryptophytes lack PR. Though often less strongly stained than in heterokonts, and either planar as in heterokonts (especially along the chloroplast cup’s inner rim) or more broadly clustered (especially around the lateral edges of NMs/pyrenoid necks), I think one can see PR vesicle/tubule clusters in most appropriately fixed/stained electron micrographs of cryptophytes, but they were invariably overlooked by the authors who did not expect them.

For example, one of the first published pictures of a nucleomorph (mistaken for a piece of the leucoplast) in the secondarily non-photosynthetic *Chilomonas paramecium* (since transferred to *Cryptomonas*) shows two putative PR patches between it and the PPM (Sepsenwol’s 1973 Fig. 5, upper right—perhaps also on the left). I think PR is visible in *Chroomonas acuta* (Kugrens and Lee’s 1988 Fig. 8, each side of NM); in *Urgorri* (Laza-Martínez 2012, e.g. Fig. 7c, both ends of the NM); in *Pyrenomonas ovalis* (Kugrens et al.’s 1999 Fig. 20, below the pyrenoid neck); in *Chroomonas* (Gantt et al.’s 1971 Fig. 1, right of the nucleus above NM), though this example differs from heterokont PR in being interspersed with ribosomes; and in the stolen cryptophytes (probably *Teleaulax*) of the ciliate *Myrionecta rubrum* (Hibberd 1977 Plate II in patches in most pyrenoid neck regions and in bulges beside mitochondria in two central plastids; Plate IIIE between left pyrenoid and NM; Plate IVD pyrenoid neck regions; Oakley and Taylor 1978, left and right corners and above NM). Since these examples include species from all five major cryptophyte lineages (Hoef-Emden 2008), PR is likely present in all cryptophytes, especially lateral to NMs and near pyrenoid necks, perhaps less often at the plastid cup inner rim. However, cryptophyte endomembrane structures in the periplastid compartment have never been studied by serial sectioning or tomography; they ought to be and are probably diverse. As *Hanusia phi* and *G. theta* have smooth NM envelope evaginations (Gillott and Gibbs 1980), without serial sections we cannot eliminate the possibility that some smooth membranes near the NM are smooth ER NM extensions, not PPM-associated PR as in heterokonts and haptophytes. In *Cryptomonas* sp. 3C, a large cisterna resembling one Golgi cisterna more than typical PR was near the plastid envelope (Gillott and Gibbs’ 1980 Fig. 3), as well as small PPM invaginations close to PR vesicles suggesting vesicle budding or fusion. In *Chroomonas salina* also some PS endomembranes resemble cisternae, not tubules or vesicles (Gibbs’ 1981a Fig. 5).

Falk and Kleinig (1968) believed the *Tribonema* PR to be in continuity with the PPM via narrow tubules, which is questionable, and cited other papers showing PR in xanthophytes, diatoms, brown algae, and haptophytes. Smith-Johanssen and Gibbs (1972) found that the *Ochromonas* tubules were an extensive reticulum, but because in chrysophytes it lies adjacent to where the PPM abuts the inner nuclear envelope membrane called to ‘perinuclear reticulum’, a misleading name stemming from Gibbs’ (1962) original misinterpretation of the PPM as part of the nuclear envelope. They treated greening *Ochromonas danica* with chloramphenicol to inhibit plastid and mitochondrial protein synthesis, which made the reticulum hypertrophy greatly, surrounding the entire perinuclear ER cisterna and extending widely between the PPM and plastid envelope, and dense granular material accumulated in the PS. Later, Gibbs (1979) adopted Falk and Kleinig’s more accurate name and found that inhibiting cytoplasmic protein synthesis by cycloheximide greatly reduced the amount of PR in greening *O. danica*. The contrasting effects of these inhibitors led her to suggest that PR was involved in protein import into the chloroplast.

Gibbs (1979) suggested that vesicles bud from the PPM and fuse with the plastid OM carrying imported proteins from the ER lumen to the plastid envelope periplasmic space. Initially, I accepted her idea that periplastid vesicles fused with the plastid despite rejecting it as the mechanism of protein import (Cavalier-Smith 1999). Later (Cavalier-Smith 2003a), because such fusion would equilibrate lipid composition between the PPM and plastid OM, contrary to the fact that in all plant chloroplasts only the outer leaflet of the OM has PC and the inner leaflet has galactolipids present only in chloroplasts, I rejected OM fusion, considering it very unlikely that this lipid asymmetry of plant OMs would have been radically changed during the origin of chromists as it is a deeply ingrained invariant feature of plant plastids (Botella et al. 2017). (Note that though all oxygenic photosynthesisers including *Chromera* have galactolipids, coccidiomorph apicoplasts replaced them by glycerophospholipids; Botté and Maréchal 2014). A second reason for rejecting this idea is that all PR studies show vesicles apparently budding from or fusing with the PPM, but virtually, none show any doing so with the plastid OM. Gibbs (1979) herself recognised that, stating that in all papers consulted, she found only one example suggestive of such fusion, and hers illustrated only three possible cases (one almost convincing, but possibly all just vesicles or tubules appressed to not continuous with the plastid OM). That should have made her more sceptical of her hypothesis, for if true (and no other vesicular paths exist in the PS), one would expect equal numbers budding from or fusing with each membrane, which is clearly not so.

I therefore suggested that the PR is a periplastid lipid synthesis factory and vesicles carry phospholipids to the PPM and return with nuclear-coded PR proteins (Cavalier-Smith 2003a). Before that, I had assumed that all phospholipids were made in the ER and imported into the PS (Cavalier-Smith 1999); an ER glycerol-3-phosphate acyltransferase was later characterised in *Plasmodium falciparum* (Santiago et al. 2004). It now appears that both ideas were partially right for Coccidiomorphea where periplastid and ER enzymes share responsibility. Periplastid versions (bipartite targeting signals) of acyltransferase in *Toxoplasma* (Bisanz et al. 2006) make the bulk of cellular phosphatidic acid, and periplastidic glycerol 3-phosphate acyltransferase (by removing phosphate) generates the bulk of diacylglycerol that is exported to the ER as a precursor for making PC and phosphatidylinositol (PI) (Amiar et al. 2016) as well as for the first steps of phosphatidylethanolamine synthesis that is completed in mitochondria. Bulk phospholipid synthesis starts in the apicoplast and finishes outside it, and additional minor salvage pathways exist (Amiar et al. 2016). I postulate that this sharing amongst evolutionarily distinct organelles is true for all chromists with PPS/PPM/PR and that periplastid glycerophospholipid precursors are made in the PR (as Cavalier-Smith 2003a suggested) and all chromists must exchange FAs and bulk phospholipid precursors between PR and ER. As PR vesicles apparently do not fuse with the plastid, OM lipid transfer proteins (Chiapparino et al. 2016) likely transfer PC to the OM outer leaflet only (at least in chromists that retain galactolipids); in all plants, PC is present in the OM outer leaflet only (Botella et al. 2017), which I predicted for all chromists (Cavalier-Smith 2003a) but now modify this to all photosynthetic chromists plus heterotrophs that retain plastid galactolipids. Lipid transfer proteins work most efficiently where membranes for lipid exchange are in close contact with minimal intervening space; I suggest that PC transfer to OM mainly occurs directly from PPM regions in close contact with the plastid OM (i.e. the majority that are free of PR); occasional rare exceptions to this could account for the rare instances of periplastid vesicles in apparent contact with the OM.

If the PR is topologically distinct from the PPM, as Gibbs (1979) and I have assumed (Cavalier-Smith 1989, 1999, 2003a), periplastid vesicles budding from the PR and fusing with the PPM would be needed to carry lipids to the PPM and vesicular budding from the PPM and fusion with the PR essential to import PR proteins made in the host cytosol from the PPM (after import into the ER lumen); in cryptophytes only could PR proteins be partially or wholly made on periplastid ribosomes. Tomography of diatom PR is consistent with this model as it shows no continuity with plastid OM but occasional continuity of PR tubules or vesicles with the PPM (Flori et al. 2016). Unfortunately, the 3D reconstruction was insufficiently precise to show if vesicle exchange is the transfer mechanism between PPM and PR or whether instead the PR is permanently connected with the PPM (as Flori et al. assume) and thus simply a specialised domain of a single topologically continuous membrane, as the interpretative drawing of Falk and Kleinig (1968) supposed. Gibbs (1979) argued that their *Tribonema* not only had a reticulum but also discrete vesicles and that their diagram omitting vesicles was oversimplified. I suspect she may be right, as some apparent vesicles appear to have coats somewhat similar to those of clathrin, but as we know nothing of their chemistry, we cannot yet eliminate the possibility that the PR is a giant reticulose coated-pit-like invagination of the PPM just as Falk and Kleinig (1968) depicted, not a topologically distinct membrane. More precise and fuller tomography of phylogenetically diverse chromist PRs is essential to distinguish between these alternatives, which have radically different cell biological and evolutionary implications (Fig. 5).

Tomography has also been applied to coccidiomorphs where I previously assumed PR is absent, confirming that (like *Toxoplasma*) *Plasmodium* apicoplasts have four membranes (Lemgruber et al. 2013) and that the *Toxoplasma* outermost membrane (EpM); see the next section) is not topologically continuous with the ER but has, on average, one contact site per apicoplast between ER and EpM that would enable lipid exchange proteins to equilibrate phospholipids between them (Tomova et al. 2009) whether made in the ER or PR. That would allow lipid exchange whilst keeping their protein composition distinct; I argued previously (Cavalier-Smith 1999, 2003a) that nuclear-coded periplastid and plastid proteins are carried from the host endomembrane system in vesicles that must fuse with the EpM. Such vesicles that carry the predominantly PS thioredoxin-like protein have now been identified (DeRocher et al. 2008). Tomography has also revealed that the PS of Coccidiomorphea is not evenly spaced all around the organelle as one might have expected if the PR was genuinely absent. Instead, every apicoplast has at least one region where the periplastid space is wider—wide enough in *Toxoplasma* at least to accommodate a planar PR similar to that of most euchromists (Lemgruber et al. 2013; Tomova et al. 2009). We must now therefore consider the possibility that Sporozoa also have a PR. Structures in the thicker *Toxoplasma* periplastid patches can be interpreted as tiny vesicles and/or tubules (Tomova et al.’s 2009 Fig. 2A, B), though contrast is too low to be totally sure or to say whether or not they are continuous with the PPM. The *Plasmodium* patches (so-called ‘gaps’) are narrower, but in one case, there is a hint of a tiny periplastid vesicle (Lemgruber et al.’s 2013 Fig. 4B), suggesting that *Plasmodium* may have periplastid vesicles at least, but contrast is too low to confirm or refute that.

I suggest that Sporozoa have a relict PR and predict that PR will also be found in chromeroid algae if searched for (existing micrographs of *Chromera* and *Vitrella* are too dense or low resolution to see it if present; Oborník et al. 2011, 2012) and that it will be universally present in all chromists with secondary red algal plastids except for Dinozoa that lost the PPM. I postulate that the PR serves both for lipid synthesis and protein import. In particular, I suggest that derlins and other ERAD-like associated membrane proteins are located not evenly throughout the PPM as traditionally assumed (Gould et al. 2015; Grosche et al. 2014; Maier et al. 2015; Sommer et al. 2007) but specifically in the PR.

Targeting and immunolabelling studies on Sporozoa and diatoms indicate that protein import may be mediated through a periplastid region equivalent to the PR. Immunogold ultrastructural labelling in *Toxoplasma* shows derlin specifically at the broad patches that, I argue, contain PR (Agrawal et al.’s 2009 Fig. 2). The non-membrane ATPase motor Cdc48 that binds to derlin when pulling preproteins through the derlin channel also preferentially labels that region but also has a little more widespread label as expected for an intrinsically soluble protein (Agrawal et al.’s 2009 Fig. 2). The E2 ubiquitin-conjugating enzyme that is essential for import (only proteins temporarily ubiquitinated by it are recognised by Cdc48) also predominantly labels the PR-like region. Thus, the labelling pattern of these key players in ubiquitin-dependent translocation is completely different from that of the soluble PS thioredoxin-like protein that apparently fills almost the entire PS and is not specifically PR associated (DeRocher et al. 2008) and also radically different from a soluble plastid stroma protein (fatty acid synthesising acyl carrier protein, ACP) that pervades the entire stroma (Agrawal et al.’s 2009 Fig. 2). Immunogold labelling is inconsistent with the periplastid translocon being located generally throughout the PPM as formerly supposed, showing instead that it is largely confined to the PR, giving PR an important reason for existing throughout derlin-containing chromists. In *Plasmodium*, even light microscopy localises derlin fluorescence to a patch on one surface of the apicoplast, unlike ACP that extends right across the very same organelle (Spork et al.’s 2009 Fig. 4C); fluorescence also shows that the Uba1 version with bipartite targeting signal that activates ubiquitin before E2 conjugates it is located in the apicoplast but lacked resolution to show precisely where (Spork et al.’s 2009 Fig. 4C). Interestingly, when only the derlin bipartite topogenic sequence was used for GFP targeting (derlin’s multiple membrane-spanning domains being absent), the label was more spread out (Sommer et al.’s 2007 Fig. 3C), but ACP-double-labelled controls still showed slight asymmetry in location on one side of the apicoplast (Spork et al.’s 2009 Fig. 2C). Together, these experiments suggest that *Plasmodium* derlin enters the apicoplast on one side only and is trapped there by its membrane-spanning domains.

In the diatom, *P. tricornutum* GFP-labelled derlin accumulates at the central neck between the two chloroplasts (Sommer et al.’s 2007 Fig. 3A) exactly where PR is located (Flori et al. 2016), and GFP targeted by the bipartite leader only of derlin, Cdc48, and ubiquitin all accumulate in that region (Sommer et al.’s 2007 Fig. 4). Thus, they colocalise with the PR, not PPM generally as previously assumed. I predict that immunogold labelling of derlin will show that it is located ultrastructurally at the diatom PR as in Sporozoa. Furthermore, three other proteins related to ubiquitin-dependent translocation also localise specifically at the central neck (Npl4, the Cdc48 cofactor mentioned above; a UBX domain protein, putatively Cdc48 binding; Pgn1 related to a deglycosylation enzyme) (Stork et al. 2012). Two proteins of the degenerate heterokont periplastid proteasome also localise there. None of the latter four proteins was identified in Sporozoa, but they have periplastid homologues in cryptophytes, except for Png1, also absent in haptophytes, suggesting that Hacrobia (and Myzozoa) have less glycosylated preproteins than heterokonts. Furthermore, only a few cryptophyte proteins needed for periplastid protein import are encoded by the NM: most are encoded by genes transferred to the host nucleus as in other chromists (Stork et al. 2012).

## Endosomal *trans*-Golgi nature of the periplastid reticulum

This new interpretation that the periplastid ERAD-related machinery is in the PR (Fig. 5) not PPM (Fig. 4) generally better explains immunolabelling data and is entirely compatible with all experimental evidence for a derlin/Cdc48 ubiquitin-dependent mechanism for protein translocation into the PS (Maier et al. 2015; Sheiner and Striepen 2014). If translocation takes place across the PR membrane into the PS, it may be spatiotemporally separated from initial TP/TPL recognition at the PPM, whose molecular basis remains entirely unknown but, as suggested above, might involve a highly modified Toc receptor as originally postulated (Cavalier-Smith 2003a). Whether the receptor was derived from Toc159 (evolutionarily simplest) or from another precursor is immaterial to the validity of the spatiotemporal separation proposal. Evidence for PR involvement in protein import seems strong, but our present ignorance allows two contrasting interpretations of its role. Either PR is a specialised highly invaginated PPM domain (Falk and Kleinig 1968) such as never existed in the ancestral red alga or it is topologically distinct from the PPM (Gibbs 1979), and vesicle transport is necessary between PR and PPM. Though it would be nicer if only one of these possibilities applied to all chromists, it could be that PR is topologically distinct in some but not others. We already know that cryptophytes have the NM envelope compartment topologically distinct from the PPM, so it would be unsurprising if they at least retain another topologically distinct endomembrane compartment (PR) and use vesicular transport between them.

Consider five facts: (1) derlins (derlin-1 and derlin-2) in animals are located in endosomes as well as ER and derlin-1 mutants or RNAi inhibit endocytosis (Schaheen et al. 2009); (2) in plants, the TGN is the endosomal compartment (Paez Valencia et al. 2016); (3) the plate-like fenestrated/tubular character of the heterokont PR (Falk and Kleinig 1968) morphologically resembles TGN; (4) in plants, TGNs can exist independently of Golgi stacks as well as associated with them (Uemura 2016); and (5) in diatoms, periplastid derlins are associated with a classical localised PR, not with PPM generally (see above). I therefore suggest that the PR is a relict standalone red algal TGN retained by the ancestral chromist *with derlins A and B already in place* when it lost the red algal Golgi stacks and rough ER. I further suggest that at least cryptophytes still have a stripped-down vesicle formation and fusion system that mediates a two-way vesicle transport between a TGN-derived PR and the PPM. I argue that all chromists retain some TGN features but suspect that in Sporozoa, these are substantially reduced and their PR might have secondarily fused with the PPM retaining its discrete morphology and become a specialised PPM domain (not a topologically distinct membrane) and could have abandoned vesicular transport (analogously to the euchromist fusion of the EpM and nuclear envelope that bypassed vesicular fusion with ER). Other chromists with PPM may have an intermediate degree of simplification between cryptophytes and Sporozoa, which probably represent opposite ends of a simplification spectrum. Our serial sectioning of PR in the heterokont *Heterosigma* showed apparently spherical 35–40-nm vesicles connected to the PPM by narrow necks as well as a much slenderer tubular network (Ishida et al. 2000). I think, it likely that *Heterosigma* vesicles without conspicuous coats bud from and/or fuse with the PPM and are not at that stage connected to PR tubules, though one cannot be sure without EM tomography of these crowded structures.

This hypothesis envisages the first import step at the PPM as akin to receptor-mediated endocytosis where the receptor’s ligand is a TP or TPL, and its attached protein is sequestered within an endocytic vesicle bound for the PR, directly descended from the enslaved alga’s endosomal compartment. Considering the PR as endosomal makes immediate sense of derlin’s likely location in it, as in animals at least derlins are in both ER and endosomes (Schaheen et al. 2009). In discussing early endocytic evolution, it is commonplace to present diagrams based on the complex opisthokont system (e.g. Wideman et al. 2014 Fig. 2) where early and late endosomes are distinct compartments from the TGN. Higher plants, however, have a simpler system with fewer compartments; TGN acts directly as an early endosome, and the elaborate opisthokont multicompartments are absent (Paez Valencia et al. 2016). That could be true also for red algae, the sisters of green plants (and likely also for ancestral eukaryotes); the complex opisthokont system is probably derived and a poor model for early eukaryote evolution—if the eukaryote tree is rooted as in Fig. 2, the ancestral Golgi was not even stacked. If red algae use the TGN as early endosomes like green plants, the red algal TGN could simply have become the PR and would have been reticular from the start. Probably therefore, derlins were originally in both TGN and ER and there was never any need to relocate derlin to the PPM as previously assumed (Sommer et al. 2007; Gould et al. 2015): it was always located in its present location even in the free-living red alga when PR was called TGN.

Therefore, the key innovations for red algal enslavement would have been TP/TPL-mediated endocytosis from the future PPM and loss of proteasomal digestion of translocated proteins, which might simply have involved losing lysine 63 in ubiquitin, normally implicated in ubiquitination for proteasomal digestion, but absent in all chromist periplastid ubiquitins (Stork et al. 2012). The only significant change needed to produce the endomembrane topology of the cryptophyte periplastid complex was the loss of the Golgi stack when glycosylation became unnecessary during symbiogenesis. Incidentally, even in temporary red algal symbiosis, extracellular mucilage secretion is phenotypically suppressed but resumed when symbionts are isolated from their host (Hawkins and Lee 2016).

A cluster of laterally associated PPM-embedded TP/TPL receptors could, in principle, themselves bend the PPM, initiating the formation of an endocytic pit (for the physics, see Johannes et al. 2014, 2015). Dynamin GTPase could then form deeper tubular pits and mediate scission to generate transport vesicles. In vitro dynamin alone can mediate both membrane tubulation and scission (Ferguson and De Camilli 2012). Specialised coated proteins like adaptins, clathrin, or caveolin are therefore not essential for vesicular transport. Viewing the PR as endosomal (not as a NM envelope derivative after its genome was lost; Cavalier-Smith 2003a) readily explains why it coexists with the cryptophyte NM envelope; they are functionally distinct membranes. It also puts a new perspective on retention of red algal Sec14 in cryptophyte and heterokont PS at least. As noted above, Sec14 is a lipid transfer protein able to move PC and PI that mediates the vesicular transport between *trans*-Golgi and endosomes and between endosomes and plasma membrane (Curwin et al. 2009, 2013). If Sec14 is genuinely absent in Sporozoa, that might mean that their PR is no longer topologically distinct from the PPM but became permanently connected to it as Falk and Kleinig (1968) assumed for *Tribonema*; alternatively, it possibly diverged beyond recognition or was functionally replaced by another (diverse lipid transfer proteins abound in eukaryotes). Clathrin-mediated endocytosis (CME) requires PI and internalises a huge variety of ligands recognised by different receptors (Doherty and McMahon 2009) and uses filamentous dynamin aggregates for final membrane scission. Coat morphology of the *Tribonema* vesicles, the presence of Sec14 in some PS, and that of dynamin are all consistent with PPM vesicle budding being mediated by clathrin, but animals have at least five clathrin-independent vesicle budding systems from their plasma membrane; any in principle might exist in red algae and have been recruited instead.

At least one of these based on flotillins is present in angiosperm plants, but another using caveolins is absent; however, caveolae are visible ultrastructurally in many protozoa and chromists so at least two non-clathrin systems are likely in red algae. Non-clathrin endocytosis generally depends on cholesterol not PI, and at least three animal systems probably depend on dynamin scission. CME appears evolutionarily the most versatile. It depends on adaptors that recognise receptors bound to their cargo ligands and links charged receptors to clathrin, thereby initiating clathrin coat polymerisation. As more than one adaptor type exists and many different accessory proteins can modify their specificity, CME is a generalised vesicle-budding system geared up for the easy evolutionary addition of extra ligands by modifying or replacing adaptors or accessory proteins with new specificity. New ligands (e.g. TP/TPL) could be transported by modifying a preexisting CME-related receptor to recognise them or a preexisting non-CME receptor (e.g. red algal Toc34 or Toc159) to allow it to bind a preexisting adaptor or accessory protein. This potential evolutionary flexibility and ease of adding ligands raise the possibility that preexisting CME molecules on the red algal plasma membrane were recruited for trans-PPM protein transport, probably mechanistically easier than my original hypothesis of insertion of Toc channel into the PPM (Cavalier-Smith 1999) or the insertion of derlin (Sommer et al. 2007). However, if clathrin was generally present in the PS, one might have expected it to have been identified in bioinformatic screens of bipartite leader bearing proteins in diatom (Moog et al. 2011) and cryptophyte genomes (Curtis et al. 2012), yet neither study found evidence for periplastid clathrin, adaptin, caveolin, or ESCRT vesicle coat-forming proteins or for SNARES for targeting vesicles prior to fusion.

However, the likelihood that if a relict vesicle transport system exists in the PS, its proteins would have undergone radical change from the ancestral state, and the fact that initially bioinformatics could not identify any Toc/Tic proteins and even now relatively few are known compared with Plantae should make us cautious about accepting this negative evidence at face value. It is also premature to conclude there is no vesicular transport in the PS for two other reasons. First, knowledge of the range of vesicular transport mechanisms even in higher plants is incomplete and effectively zero for red algae and we could not recognise unknown transport proteins. Secondly, though one might expect PS clathrin to have bipartite leaders, PPM-located proteins involved in relict non-clathrin endocytosis need not have bipartite leaders as they do not have to cross the PPM and might insert directly from the ER lumen by another mechanism. We know too little about import signals to the PPM to recognise them generally. Nonetheless, as only one clathrin paralogue was found in *G. theta* (Curtis et al. 2012) and the tiny PPM invaginations next to some larger candidate PR tubules in *Cryptomonas* 3C lack obvious coats and are smaller than typical coated vesicles (Gillott and Gibbs 1980), if vesicle transport exists in the cryptophyte PS, I doubt that it uses clathrin. The presence of PS dynamin in diatoms noted above would provide a mechanism for vesicle membrane scission, but as it may function instead or in addition in plastid division, it does not necessarily indicate vesicle formation. As NMs are often in close contact with PPM and the plastid envelope and PS with the PPM, it could be argued that in principle lipid transfer proteins might be sufficient to exchange lipids amongst these membranes, so vesicle transport may not be strictly necessary.

If periplastid vesicle transport exists, it would be stripped down to bare essentials: machinery for membrane tubulation, scission, and fusion. Elaborate devices for sorting and specifying different vesicles for different target membranes like SNAREs could be lost together with numerous other membranes that otherwise might be a source of confusion; proteins for interaction with the discarded cytoskeleton would also be lost. Peroxisomes, mitochondria, and all endomembranes except TGN would have gone (except for the smooth NM envelope in cryptophytes). However, some proteins additional to dynamin and a TP/TPL receptor might have been retained from one or more red algal vesicle transport systems. In searching for them to test this hypothesis, one must bear in mind the likelihood of substantial simplification (possibly greater in some groups, e.g. Sporozoa) and divergence; for example, trypanosomes lost the standard adaptor AP2 yet still efficiently conduct CME (Manna et al. 2015). If my proposal is correct and derlin functions as a PR not PPM translocon, it would have been unnecessary for euchromists to have evolved a specific TP/L receptor protein for the derlin translocon, as required on the standard model (Sommer et al. 2007). Specificity would reside in the PPM TP/TPL receptor, as only imported proteins with TP or TPL would enter the PR lumen, so they could be indiscriminately extruded across the PR (former TGN) by derlin/Cdc48. Thus, no translocon need be relocated, a much simpler explanation of periplastid-targeting origin than any previously—consistent with experimentally demonstrated location of all tested mediating molecules, unlike any previous explanation. Given the known evolutionary ease of recruiting new ligands to CME, it is possible that during the origin of chromists, a preexisting CME receptor did acquire the ability to recognise TP/TPL, thus readily establishing import of plastid-destined proteins to the TGN/PR lumen where derlin already from the very beginning awaited them for extrusion into the PS. Then later after the red algal cytoplasm was radically simplified, the adaptin/clathrin coat could have been lost and only the receptor and dynamin retained. I return to the likely evolutionary ease of adding new receptors to CME in the next section on EpM origin. One corollary of this model is that PR-resident proteins (? derlin itself) should have a PR-retention mechanism, preventing extrusion into the PS (former red algal cytosol) by derlin/Cdc48.

The ancestral chimaeric chromist would have had multiple host dynamins and several red algal dynamins. This redundancy must have lessened by the differential loss as chimaera components integrated. After ancestral halvarian diverged, alveolates apparently retained a different dynamin paralogue for plastid division from heterokonts; though the diatom dynamin is periplastid (Moog et al. 2011), the precise location of the apicoplast version is unclear (van Dooren et al. 2009)—it would be unsurprising if it were cytosolic, as secondary simplification in diverging lineages can easily happen differently.

Recruiting CME or another receptor-based endocytic mechanism for import was probably easier and then relocating a complete Toc or derlin to the PPM as previously hypothesised (Cavalier-Smith 1999; Sommer et al. 2007). Previously, I suggested that as Toc34 can be self-inserting it could have initiated evolution of a PPM translocon, and insertion of Toc159 and Toc75 (which uses a TP for insertion, so would have required the other two first) could simply follow (Cavalier-Smith 2003a). That would have placed a functional Toc in the PPM, thereby enabling any host-encoded protein with TPs to cross the former PM and enter the plastid using a still symbiont-encoded Toc. As soon as a functional Toc was inserted into the algal PM, its translocation should be as efficient as for a plastid Toc, making the algal PM thenceforth a functioning PPM. The weak link in that scenario was the assumption that Toc75 with a TP could have been inserted into the nascent PPM in the absence of preexisting PPM Toc75, merely using an incomplete Toc34/Toc159 receptor. I now conclude that never happened, for if it had chromists would at once have evolved a functioning PPM translocon and never needed to recruit derlin/Cdc48 for protein import.

A PPM derlin translocon without vesicular involvement (Sommer et al. 2007) is conceptually simpler than my present hypothesis, involving first endocytosis, then vesicle fusion to PR, then derlin/Cdc48 extrusion out of PR. But a conceptually simple solution cannot evolve if the path to it is harder (either mechanistically or selectively insuperable) and far less likely than a roundabout, conceptually messy, route exploiting preexisting cellular locations of mediating molecules. If true, it will be a nice example of evolution explaining complexity better than intelligent design. For a designer, a simple result matters; for evolution, not simplicity of the result but an easy path is what matters, however meandering and counterintuitive, however complex the result; no designer/creator would have made the immensely complex derlin phylogeny (Fig. S1) or chromist membrane topology (Figs. 3 and 5).

Not only the red algal Golgi but also its ER underwent simplification in the ancestral chromist, which appears to have lost the SRP and therefore essentially changed from rough to smooth ER (Cavalier-Smith 2003a). Sequences of other cryptomonad NM genomes also failed to find SRP-RNA genes, and no periplastid-targeted SRP proteins or periplastid Sec61 cotranslational trans-ER protein-translocation channels were found in the *G. theta*’s nuclear genome (Curtis et al. 2012). Unless overlooked through divergence, red algal/periplastid cotranslational protein translocation was lost and protein insertion into the NM envelope, PR, and PPM must all be post-translational in cryptophytes, a considerable cytoplasmic simplification. Possibly derlin was coopted for inserting proteins into the PR as well as translocating them through it as Maier et al. (2015) suggested for the PPM. Sec14 might equilibrate lipids between NM and PR membranes as they are often close together, so vesicular transport between them might be unnecessary if both have lipid scramblases (proteins catalysing bidirectional lipid flipping between bilayers, thus allowing equilibration in both despite insertion from the synthesis side only; Pomorski and Menon 2016) and NM-specific proteins can be directly inserted. However, ~ 2400 (mostly nuclear-coded) proteins are present in the NM, PS, PR, and PPM (Curtis et al. 2012) so they are much more complex than was once assumed. It was incorrect to state that the cryptophyte periplastid complex has no ER (Gould et al. 2015); the NM envelope is ER but not rough ER. My suggestion that its envelope was not lost in other chromists but was modified to become PR (Cavalier-Smith 2003a) is wrong if my present thesis that cryptophytes all have PR is correct. It now seems clear that cryptophytes retain seven distinct types of red algal membrane—three in the chloroplast plus nuclear envelope/ER, TGN (PR), transport vesicles, and PPM.

My new interpretation also readily explains PR hypertrophy when plastid protein synthesis is blocked and retrogression when cytosolic ribosomes are inhibited in heterokont algae (Smith-Johanssen and Gibbs 1972; Gibbs 1979). Cycloheximide will block synthesis of cytoplasmically made proteins with bipartite leaders, both resident PR proteins (e.g. derlin, ubiquitinating enzymes) as well as those being carried from the PR lumen into the PS (many later destined for the NM, e.g. histones, DNA replication enzymes; hundreds only for onward passage into the plastid). Vesicles recycling lipid and TP/L receptors to the PPM (Fig. 5, PRV) will deplete the PR membranes, greatly reducing their extent. Conversely, blocking plastid protein synthesis must prevent plastid growth but derlin will continue to import PR, PS, and plastid proteins, which will accumulate in both the PS and PR, greatly extending the latter, and if feedback controls also reduce import of plastid proteins, these could back up and exacerbate PR hypertrophy.

PR position appears well conserved in heterokonts. In the majority with one or two plastids per cell and obvious ribosomes around the entire peri-PPM ER, it abuts the nucleus, as noted above. A small minority in several classes, exemplified by raphidophyte *Heterosigma* and xanthophyte *Tribonema*, secondarily evolved numerous chloroplasts mostly not directly associated with nuclei. Some like *Heterosigma* lack ribosomes on the periplastid ER abutting the chloroplasts, which was once mistakenly thought not to be connected to the nuclear envelope, though others such as *Tribonema* with numerous plastids retain periplastid ER ribosomes. In *H. akashiwo*, serial sections reveal slender tubules connecting peri-PPM smooth ER to rough ER cisternae and others connecting them indirectly to the nuclear envelope (Ishida et al. 2000). The PPM does not penetrate these narrow ER channels, so proteins destined for the PS are made on ribosomes attached far away from the PPM and must diffuse up these tubules before encountering PPM TP/TPL receptors. In *Heterosigma* therefore, the PR cannot abut the nucleus but is located against the inner face of the inward projecting pyrenoids (Ishida et al. 2000), close to where diffusing periplastid preproteins first encounter the PPM. *Heterosigma* may have been predisposed to lose peri-PPM ER ribosomes by its chloroplasts becoming fixed to its cortical alveoli far from the nucleus. Close ER attachment to cortical alveoli necessarily precludes ribosome retention there; the great distance from the nucleus may have made it more economical not to have them on the inner plastid face either. *Tribonema* perhaps retained ribosomes on its peri-PPM ER because it lacks cortical alveoli and its chloroplasts are surrounded by normal cytoplasm and may be more mobile.

The *Heterosigma* geometry is likely to apply to all multiplastid heterokonts with smooth peri-PPM ER and strongly supports Gibbs’s (1979) thesis that PR is involved in protein import—somehow! But, it does not contradict my hypothesis that PR also makes lipids—though it would not need to make lipid in that particular location; siting manufacture at the point of import of any lipid-synthesising enzymes makes PR growth more efficient. These specific locations of PR imply specific proteins for anchoring or making it. The fact that the PPM in multiplastid heterokonts is not associated with the nucleus strikingly emphasises the erroneous nature of Gibbs’ (1962, 1981a, b) initial assumption that it was part of the nuclear envelope and therefore part of the ER as it makes it abundantly clear that the PPM is located in the ER lumen, proving that Whatley et al. (1979) and I (Cavalier-Smith 1982) were right in arguing that the PPM is NOT topologically connected to the ER and, almost certainly, a relic of the red algal plasma membrane. Eventually after our criticisms, Gibbs (1983, 1993) accepted the now standard view that the PPM is a former plasma membrane, but still regarded the origin of peridinean chloroplasts as a mystery through wrongly assuming an independent origin from euchromists (and multiple origins of them!). If I am right that derlin is in the PR and reaches it only by quasi-endocytic PPM vesicles (Fig. 5, PEV), then even today the PPM has no ER-like character.

Nonetheless, Zimorski et al. (2014) in an otherwise exceptionally lucid and rational discussion of endosymbiotic origins of organelles pictorially refloated Gibbs’ (1962, 1979) implicit idea that the PPM and EM both arose from ER, without giving any reason for proposing such an immensely more complicated scenario than the simple standard model, apparently not realising that she had already effectively proposed it or after criticism rejected it (Gibbs 1983). Their model altogether ignores the PR and, contrary to Cavalier-Smith (2003a) and the present synthesis, suggests that myzozoan EpM vesicular targeting arose secondarily without explaining how or why. Before explaining its serious defects, I must outline and extend the sounder standard explanation of the origin of the EpM.

## Origin of the myzozoan EpM

As emphasised above, the myzozoan EpM is most likely a relic of the perialgal vacuole (PV) membrane that surrounded the original red algal symbiont (Cavalier-Smith 2003a). PV membranes (sometimes called symbiosome membranes) are not just unaltered pieces of PM but are modified from phagosomes (themselves non-identical to PM) or digestive vacuoles that arise from phagosomes by fusion of lysosomal vesicles carrying digestive enzymes. PVs around symbiotic *Chlorella* in ciliates arise by reproducibly timed budding from digestive vacuoles (Kodama and Fujishima 2009) and, unlike phagosomes or digestive vacuoles, can divide and be maintained over many cell cycles by interactions between the chromist host and plant symbiont and are specifically anchored in the cell cortex. PV budding depends on the host recognising the large size of *Chlorella* compared with bacterial prey that are immediately digested and is probably effected by dynamin (Kodama and Fujishima 2012), which might also mediate later PV divisions. *Chlorella* is postulated to secrete a maltose transporter into the PV membrane, and an inhibitor of lysosomal fusion; if symbiont protein synthesis is blocked, its cells degenerate and lysosomes fuse with PV and digest *Chlorella* after it ceases to provide maltose (Kodama and Fujishima 2011). In the *Chlorella*/hydra symbiosis, low-maltose secretors are digested not cultivated (Davy et al. 2012).

Ciliate and hydra *Chlorella* symbioses mediated by maltose export to the host and the coral/dinoflagellate symbiosis where very diverse organics pass from the chromist *Symbiodinium* to the animal host (Davy et al. 2012; Venn et al. 2008) are best thought of not as a mutualism, as is usual (Venn et al. 2008). Instead, they are probably intracellular cultivation of an alga by an exploitative host—*temporary enslavement* conditional on the symbiont providing the host with enough photosynthate to make its retention worthwhile (Wooldridge 2010). All three provide excellent practical and conceptual models for the first stages of the *permanent enslavement* of a red alga that made chromists. The details differ, for example the sugars provided to the prechromist by a red alga were likely different, possibly floridosides (Kremer et al. 1980; symbiotic red algae isolated from foraminifera are ultrastructurally indistinguishable from free living ones; Hawkins and Lee 1990). Cultivating *Symbiodinium* profoundly alters cnidarian host gene expression as it shifts from heterotrophy to phototrophy, giving it diverse food and probably protection from oxidative stress (Oakley et al. 2016). The cnidarian symbiosome membrane proteome includes many host PM receptors that may be involved in recognition and signalling but apparently also proteins of symbiont origin (Peng et al. 2010). Whether the latter insert into the symbiosome membrane by host manipulation of the symbiont or result from selection on the symbiont to allow it to colonise the host as a beneficial habitat is unclear. Either way these symbioses are preadapted for later evolution of permanent enslavement by insertion of host proteins into the PV membrane to make it an EpM and into the symbiont PM to make it a PPM. This makes it highly probable that the initial step in red algal permanent enslavement to make a chromist was not extraction of photosynthate; that had already been achieved and must have been relatively easy to evolve as there are innumerable symbioses where heterotrophs cultivate algae intracellularly.

Most likely the first step was insertion into the EpM of a novel receptor enabling more reliable delivery of host proteins to the PV lumen to increase efficiency of extraction of photosynthate or the diversity of useful molecules from the red alga or better control this or algal growth/division. Inserted proteins could be soluble proteins active inside the PV lumen (e.g. shuttling lipid transfer proteins that could move symbiont lipids from algal PM to EpM) or membrane proteins that could insert into the algal PM as metabolite translocators or signalling receptors. In a long-standing well-adapted symbiosis sooner or later numerous symbiont genes would inevitably be transferred to the host nucleus (Cavalier-Smith 1982, 1999; McFadden 1999), after which, the repertoire of host-synthesised proteins available for insertion into the PV membrane would include genes of symbiont origin duplicated in symbiont and host. Thereafter, symbiont proteins made by the host could be inserted into the symbiont PM, giving the host more control over them because host benefit not symbiont benefit would decide when they were expressed. At this transitional stage, hundreds of former symbiont proteins could have been made in the host cytosol, both soluble ones and others bearing symbiont TPs or signal sequences. Those with signal sequences would enter host ER (perhaps being extruded by ERAD and digested by proteasomes if misfolded), but those with TP would either just stay in the cytosol or be mistargeted to other organelles probably often being stuck into their cytosolic face. Most such errors would be selected against, variants being favoured with rogue genes deleted or modified to reduce mistargeting. By chance, some such intra-endomembrane proteins might enter vesicles able to fuse with the PV membrane and thus enter the PV lumen and, in some cases, insert into the red algal PM.

We shall never know which nuclear-coded proteins were the first to be thus inserted, but as suggested before (Cavalier-Smith 2003a), such proteins would inevitably have included symbiont Toc TP receptors which could therefore have been inserted into the algal PM from the PV lumen, enabling them to be recruited for a PPM import system as discussed above. However, initially, a weak link would have been PV membrane fusion of Golgi-derived vesicles bearing proteins with TP, which would inevitably be initially inefficient and of low specificity, and thus a bottleneck. But as long as it worked well enough to import some proteins whose new location benefited the host, it could have been rapidly improved through selection for greater specificity.

A key innovation was probably a novel SNARE system for targeting host endomembrane fusion with the PV membrane that became specific for proteins bearing terminal signal sequences and subterminal TPs (Cavalier-Smith 2003a), an idea developed further here, but in a way compatible with existence of periplastid derlin. Specific SNARE-mediated vesicle fusion depends on tight zipping of SNARE proteins on the vesicle (v-SNAREs, each a complex of membrane-inserted syntaxin and SNAP-25 bound to its free tail) together with complementary t-SNAREs (e.g. synaptobrevin) in the target membrane with a protruding syntaxin-binding tail. This zipping precedes membrane fusion and follows first contact mediated by membrane tethering factors. Every eukaryote cell has 20 or more SNAREs specific for different membranes; only cognate SNAREs allow membrane fusion (Baker and Hughson 2016). Duplicating and modifying SNARE genes must be the main way eukaryotes evolve new genetic membranes (Cavalier-Smith 2000b, 2004b). Tethering factors on target membranes are also specific for different destinations (e.g. golgins on Golgi membranes) so trap vesicles at the right membrane; having two successive selective steps increases specificity and reduces mistargeting, but a novel SNARE pair would be sufficient to initiate evolution of a novel genetic membrane like the EpM. Small GTPase Rab proteins act as switches both in forming and fusing vesicles to which they can bind (also to target membranes and tethering proteins); though different membrane pairs typically have different Rab proteins (Chua and Tang 2015; Liu and Storrie 2012), Rab differentiation is less likely than SNARE modification to be the primary step in evolving a novel target membrane.

That requires not only modifying vesicle fusion but also generating the novel vesicle at the donor membrane. Apparently, apicoplast-destined vesicles form at the Golgi (Heiny et al. 2014) as originally postulated (Cavalier-Smith 1999), not at the ER as early empirical evidence suggested (Cavalier-Smith 2003a). I now postulate that budding is at the host TGN and initially at least used the same budding machinery (most likely clathrin-mediated) and transit receptor as the PPM discussed above. Later, they may have diverged, but postulating a *single shared receptor in the ancestral chromist* greatly simplifies origin of periplastid targeting compared with all previous hypotheses. Both opisthokont and plant clathrin-coated vesicles bud from the TGN (Paez Valencia et al. 2016) and can transfer their cargo to the PM; they bud from the PM carrying cargo directly to the TGN in plants (indirectly via separate endosomes in opisthokonts). Therefore, clathrin-coated vesicles could initially have been responsible for host TGN to EM transport as well as from symbiont PPM to PR and from PR to PPM recycling transport: three birds killed with one stone. The first two types would necessarily carry ligand-charged TP receptors that mediated budding, whereas recycling from PR to PPM should recycle discharged TP receptors. Early coevolution of the three TP receptor-dependent vesicle types could have started chromist origin in a way impossible on other views.

This simplification depends on accepting (1) that PPM and EpM arose from endosymbiont and host PMs, respectively; (2) that PR is homologous with TGN; (3) derlin/Cdc48 is located and function at the PR; (4) Myzozoa have PR; (5) the transport between PPM and PR originally depended on coated vesicles (probably now simplified as suggested above); (6) the transport from host TGN to EpM depends on coated vesicles; and (7) myzozoan-like TGN to EpM targeting is ancestral for chromists, but Ochrophytina and Hacrobia secondarily lost it as independent simplifications by two fusions of EpM and nuclear envelope that placed their PPMs directly inside the rough ER lumen. There is no good evidence against any of these assumptions and some direct or indirect evidence for all of them, as explained above; direct labelling evidence exists for (3).

Despite there being no good reason to abandon the standard interpretation of the origin of chromist membrane topology, Zimorksi et al. (2014) depicted without any explanation a radically different model that instead assumes that myzozoan vesicle targeting to the EpM evolved secondarily from euchromist ancestors with PPM already inside the rough ER lumen. That makes no evolutionary sense whatever; the euchromist import system functions perfectly well, so there would be no selective advantage in making it more complicated by (a) separating periplastid ER from rough ER and (b) losing ribosomes on it and then interpolating (i) novel TGN vesicle budding and (ii) novel vesicle fusion to EpM (i.e. stages c and d in Fig. 5). Not only would that gross complication be selectively disadvantageous and prevented by selection, but mechanistically immensely complex. Neither they nor Gould et al. (2015), who adopted it as if it were a plausible possibility, suggested a selective advantage or tried to explain how such a complicated change and interpolation could be achieved mechanistically. Had they begun to try to explain it, they probably would have realised its extreme evolutionary implausibility. Unless they can provide advantages and mechanisms more plausible than those explained here, this speculation should be ignored. Maier, the only coauthor of Gould et al. (2015) not also involved in Zimorski et al. (2014) wisely ignored its dubious novel phylogenetic scenario and stuck to the standard model that regards the PPM as PM in his parallel paper (Maier et al. 2015).

The scenario implied for the origin of euchromist membrane topology by Zimorski et al. (2014) and Gould et al. (2015) is also incredible. They assume that after the red alga was phagocytosed into a PV, permanent enslavement was initiated not by converting the PV into EpM as explained above but by losing the PV placing the red alga in the cytosol, followed by surrounding it by rough ER extensions from the nuclear envelope that supposedly fused together in one step to make two functionally differentiated membranes: outer rough ER and inner smooth membrane. Then, the red algal PM was supposedly lost and endosymbiont derlin transferred to the smooth membrane supposedly derived from host ER by ribosome loss. So, symbiogenesis supposedly started with four surrounding membranes, reduced it to three, then increased it to five, then reduced it to four—‘a great investment of cell biological activity for a conspicuous lack of evolutionary change’ as Gould et al. (2015) themselves put it when rightly criticising the similarly hugely complicated serial tertiary transfer hypothesis demolished by the next section. I once proposed precisely the Zimorski-Gould mechanism for the simultaneous origin of PPM and its surrounding ER in a non-symbiotic context (Cavalier-Smith 1977) but, after deeper thought on protein targeting, abandoned it as too sudden and traumatic (Cavalier-Smith 1980) and accepted instead a symbiotic origin from the algal PM (Whatley et al. 1979) that allows gradual change in small steps. Later, I explained more fully why this ‘superficially plausible’ idea suffers from the ‘fundamental problem’ that it envisaged far too many complex evolutionary innovations happening simultaneously at the stroke of a designer’s pen (Cavalier-Smith 1986, pp. 312–5).

Despite its entirely unnecessary complexity, the Zimorski-Gould scenario makes no mention of the PR or how it evolved or its functions, despite it being known in chromists for over half a century, a flaw also of the standard model of Sommer et al. (2007) that did accept that the PPM is the former red algal PM. Not only does the Zimorski-Gould scenario grossly contravene Ockham’s razor (*entia non multiplicanda sunt*), but it is implausible that an enveloping ER cisterna could simultaneously encapsulate the red alga, surrounding the red alga by two new membranes not previously adapted for harbouring symbiotic algae (unlike the PV); give them competence to exchange metabolites (thus avoiding instant death); and evolve novel protein targeting involving protein transfer to a membrane that never had it *in a single step*. Analogous sudden encapsulation by two host membranes and simultaneous instant evolution of transenvelope protein targeting were entailed by the autogenous theory of the origin of mitochondria and chloroplasts (Cavalier-Smith 1975, 1977) and a major reason why I abandoned autogenous origins of chloroplasts (Cavalier-Smith 1980) and mitochondria (Cavalier-Smith 1983a, b, c) soon after I thought deeply about chloroplast protein targeting (Cavalier-Smith 1982). The other reasons were phylogenetic, but in my thinking evolution of protein targeting and cell biology always had at least as much weight as phylogeny. Both must be satisfied by sensible evolutionary explanations.

The Zimorski-Gould scenario treats the PPM as of ER origin as supposed by the defunct early ideas of Gibbs (1962) about euchromist membrane topology and my defunct autogenous theory (Cavalier-Smith 1977). The only reason given for this retrograde step was to avoid a necessity for fusing the PV with the nuclear envelope. But, such fusion is mechanistically an extremely simple membrane accident requiring no DNA mutation, avoiding it is a paltry ‘benefit’ of the excessively complex scenarios criticised in the preceding three paragraphs. Defects are

1. Failure to mention, still less explain, the origin and role of PR.
2. Lack of evidence for the assumption that derlin is in PPM not PR.
3. The complexity and oddity of going from four to six to five then to four membranes for no good reason, instead of starting and ending with four.
4. Failure to appreciate cell biological complexities of each assumed intermediate step.
5. Totally ignoring selective advantages of steps; some clearly disadvantageous.

I first met the peculiar membrane topology around chromist chloroplasts in a 1961 lecture by Irene Manton (who with Mary Parke had just discovered it in haptophytes) to the British Association for Advancement of Science at Norwich. She said we have no idea what is ‘the function’ of that remarkable topology with four bounding membranes. Even then, aged 18, I thought that the wrong question. They have no function in the standard engineering design sense. Peridinean dinoflagellates lost PPM and PR and their chloroplasts still function perfectly well physiologically. Later sections argue that when haptophyte plastids replaced peridinean chloroplasts surrounding membrane topology was probably substantially simplified, indicating that some extra membranes of haptophytes are functionally unnecessary: just evolutionary relics of the complex meandering evolutionary pathway (but with individually simple steps) that made chromists. So also is the fifth unnecessary chromist membrane: the cryptomonad NM envelope, lost twice—independently in haptophyte and harosan ancestors. Sometimes evolution succeeds in simplifying, thereby hiding evidence of its complex meander, sometimes it does not, getting stuck in local optima—phylogenetic constraints make complete ‘design optimisation’ impossible. Darwin was right and design-obsessed creationists like Paley wrong. A tortuous and implausible panselectionist answer to that wrong question of ‘the function’ of chromist membrane topology by Lee and Kugrens (1998) (supposed competitive superiority in low CO_2_) was evolutionarily muddled, as it wrongly lumped euchromist PPM and peri-PPM ER, and euglenoid and dinoflagellate EpMs all together under the defunct term ‘chloroplast endoplasmic reticulum’, wrongly assumed the PPM arose from the PV, and wrongly equated PV and digestive vacuole; and so postulated that PS therefore would still be an acidic compartment that could help CO_2_ uptake!

## Tertiary symbiogenesis and its evolutionary implications

When publishing 18S rRNA ML and parsimony trees showing alveolates and chlorarachnid Rhizaria branching within euchromists, Cavalier-Smith et al. (1994) pointed out than even if euchromists all use the same periplastid protein-targeting machinery (as we now know they and myzozoan alveolates do), that would not prove that euchromists were ancestrally photosynthetic and evolved by one secondary enslavement as I had long argued (Cavalier-Smith 1982, 1986, 1989). That is because serial lateral transfer of an early photosynthetic chromist cell (e.g. a cryptophyte) to unrelated heterotrophic hosts combined with fusing its nucleus with that of the host could *in theory* have moved not only its plastid but also essential nuclear-encoded periplastid targeting proteins into an unrelated heterotrophic host to make other euchromist algae (Ochrophytina, haptophytes) and also dinoflagellates in one to three higher-level symbiogenesis events. Later, I called such then purely hypothetical enslavements of a preexisting chromistan alga *tertiary symbiogenesis*, when showing a fastML 18S rDNA tree depicting Rhizaria (then still called Rhizopoda), including heterotrophic and algal Cercozoa, as sisters to Halvaria (Alveolata + Heterokonta), thus first showing clade Harosa (Cavalier-Smith 1995a)—but computers were then too puny to calculate bootstrap values for 64 rDNAs.

I did not consider serial tertiary transfer the best explanation of chromist plastid distribution but suggested it as *possibility* primarily because it offered a potential alternative explanation of the puzzle that none of the four chromophyte groups appeared to have split anciently into two deeply divergent algal lineages in rDNA trees and none appeared unambiguously in the fossil record before the Mesozoic, whereas divergence between these chromistan lineages and the deep branching of cryptomonad NMs within red algae (Cavalier-Smith 1995a) implied that chromist lineages collectively diverged from Plantae in the Precambrian, at least twice as long ago. My perspective now is very different, for three independent reasons:

First, discovery of deeper-branching plastid-bearing lineages than then known in two of the four chromophyte lineages (e.g. chromeroids and apicoplasts in Myzozoa) removed my reason for postulating serial transfer to explain the seemingly late fossil appearance of chromophytes. Secondly, discovery of a genuine case of tertiary transfer from a haptophyte to a dinoflagellate to generate the aberrantly pigmented dinoflagellate family ‘Kareniaceae’ with only four genera (Daugbjerg et al. 2000; Tengs et al. 2000) has shown that the only known tertiary symbiogenesis *did not mimic* the membrane topology found across chromists, making it exceedingly improbable that one to three independent ones could all have done so (references below). (Kareniaceae is an invalid name, so I call them Karlodinia: Table 1, S1). Thirdly, if my hypothesis of relatively late serial tertiary transfers had been correct, multigene chloroplast trees should show serial nesting of the supposedly derived three chromophyte groups within the supposedly ancestral one, with the same confidence as such methods have shown three entirely independent secondary enslavements of green algae to generate Euglenophyceae, the rhizarian Chlorarachnida, and dinoflagellate *Lepidodinium* (Matsumoto et al. 2011b). This prediction is firmly refuted by the best 34-protein chloroplast genome trees where all four chromophyte groups are separate clades not nested within each other (Janouškovec et al. 2010).

Furthermore, the chromist branching order for that chloroplast tree is exactly the same as for our recent 187-protein tree based on even more chromists and the richest taxon sampling for eukaryotes and the most intensive investigation of the effects of differential sampling for nuclear genes to date (Cavalier-Smith et al. 2015a). Within chromists, both that nuclear-coded protein tree and the chloroplast genome tree show clades Halvaria (Heterokonta plus Alveolata) and Hacrobia. Widespread claims that chromist nuclear and chloroplast phylogeny are incongruent (e.g. Baurain et al. 2010; Bodyl et al. 2008) were premature and reflected temporary apparent conflicts between ill-sampled or technically inferior trees, which several authors misread as a genuine evolutionary problem. In fact, the best multiprotein trees show that nuclear and plastid evolution are so strongly congruent across the whole of chromists that monophyly of both subkingdoms Harosa and Hacrobia is firmly established. They decisively refute my tentative hypothesis of late serial tertiary symbioses into heterotrophic hosts (Cavalier-Smith et al. 1994). The congruence of most of the best nuclear and chloroplast trees supports my earlier thesis that all euchromists (Cavalier-Smith 1982, 1986), all chromalveolates (Cavalier-Smith 1999), and all Chromista as expanded to include alveolates, Rhizaria, and heliozoans (Cavalier-Smith 2010) as well as the new heterotrophic cryptist subphylum Corbihelia (Cavalier-Smith et al. 2015a) originated from a single ancestral secondary enslavement of a red alga and vertical inheritance of all chloroplasts using ubiquitin-dependent derlin/Cdc48 periplastid protein targeting.

As noted above, late tertiary transfers are also refuted by derlin sequence trees which show the phyletic depth of crown halvarian and haptophyte periplastid derlins as comparable to that between the red algal, heterokont, and haptophyte ER derlins (Fig. S1; Petersen et al. 2014); thus, there is no significant mismatch between divergence times of alveolate, heterokont, and haptophyte host cells and those of their chloroplasts. The derlin tree robustly refutes the much repeated idea that heterokont and haptophyte periplastid derlins had a cryptophyte not red algal origin (Fig. S1). The periplastid Cdc48 tree also indicates that divergences amongst halvarian, haptophyte, and cryptophyte periplastid targeting were close to the base of all chromists; those of Halvaria and haptophytes did not nest within those of cryptophytes (Petersen et al. 2014). Though Petersen’s ML derlin tree weakly put heterokonts deeply within Myzozoa (certainly false as Myzozoa have a derived RuBisCo) and Cdc48 put Myzozoa weakly within heterokonts in their trees, my far better sampled ML trees (Figs. S2, S4, S6) instead put the heterokont clade within paraphyletic alveolates with weak support (clearly wrong), but my site-heterogeneous derlin trees did weakly show an alveolate periplastid derlin clade within one of the two heterokont B paralogues (Figs. S1, S3, S5). These statistically insignificant contradictions are probably largely random error because these proteins are short; overall, halvarian derlin trees do not exclude heterokonts and alveolates being sisters. All derlin trees are fully consistent with vertical inheritance of chromist periplastid machinery and refute the theory of late serial tertiary transfer (Cavalier-Smith et al. 1994). Despite that, and in seeming ignorance of my 1994 theory, Petersen et al. (2014) advanced a similar one, dignified as the ‘rhodoplex hypothesis’, but substantially worse as it absurdly imagines multiple independent tertiary transfers into Myzozoa (one for Apicomplexa; one to two for dinoflagellates and *Perkinsus*), giving three to five supposed tertiary transfers, and made it almost untestable by not specifying the source of any of them. It lacks respect for Ockham’s razor, fails to engage with chromist cell biology in any way, and makes no evolutionary sense, despite erroneously claiming to ‘make sense of all the available evidence’; it lacked significant scientific content or mention of specifically how it might be superior to the simpler assumption of no lateral transfer of chromist periplastid targeting. Earlier arguments by some of these authors denigrating the now strongly supported thesis of a single vertical inheritance of chromist periplastid targeting were refuted in great detail (Cavalier-Smith et al. 2015a).

The serial tertiary transfer hypothesis of Bodyl et al. (2008) though also invoking four superfluous tertiary transfers was superior in its specificity allowing criticism and refutation and giving rational (albeit unconvincing) reasons for proposing it. One motivation (interim multigene trees for chromists seemingly contradictory between nuclear and chloroplast genes) is invalid. A second motivation was to minimise plastid losses—a very bad reason as early plastid losses were extremely easy yet tertiary transfer to mimic the same membrane topology as the original secondary symbiogenesis must be extremely difficult. Even one successful tertiary transfer mimicking that topology is immensely less likely than 20 independent losses; invoking four though logically possible was bad evolutionary and cell biological judgement. If the Bodyl et al. (2008) tree topology were correct (holophyletic Hacrobia), the two worst assumptions are two transfers into Myzozoa, from haptophytes to Dinozoa and from heterokonts to *Chromera*. The latter did not include the origin of sporozoan apicoplasts which would require a fifth transfer on their scenario. Present evidence strongly confirms that all Myzozoa, which originated very early in alveolate evolution, descend from a photosynthetic common ancestor. Though late chloroplast losses are difficult, two are known in parasitic Myzozoa (in both infraphyla): in *Cryptosporidium*/Orhogregarinea (Cavalier-Smith 2014a) and in *Haematodinium* (Gornik et al. 2015). The fact that *Chromera* and *Vitrella* branch separately amongst heterotrophic apicomonads proves several photosynthesis losses in Apicomonadea, but as heterotrophic *Voromonas* has plastids (Gile and Slamovits 2014), possibly there was no plastid loss. Plastid loss would have been easier in the deeper branching protalveolates (but, like most heterotrophic myzozoans, they might still have relict plastids), stem Ciliophora, and within Heterokonta in the deepest branch (stem Bigyra), so there is no sound reason to reject the idea that stem Halvaria were photosynthetic. The wrong topology at the base of Heterokonta of the Bodyl et al. (2008) tree inflates the number of losses necessary by two: multiprotein phylogeny shows Bigyra as a strong clade (Derelle et al. 2016) not three successive basal branches (bicosoecids, labyrinthulids, and opalinids) as wrongly on the Bodyl tree. One loss in stem Rhizaria and others early in hacrobian evolution, e.g. in stem Corbihelia, almost the deepest branch, would have been equally easy—evolutionarily trivial, so it is wrong to weigh equally one loss against one gain as naive unweighted parsimony assumes. Parsimony arguments merely counting events are unsound if (as here) their probability is grossly unequal; evolutionarily well-judged weighting is mandatory for a sensible conclusion. One must think critically and constructively about mechanisms in addition to being able to count.

The discovery of rappemonad chloroplast sequences, sisters to haptophytes, puts the photosynthetic ancestry of Haptophytina much further back in time and deeper in the chromist tree than it once appeared (Cavalier-Smith et al. 1994), so crown Haptophytina appear substantially older than crown cryptophytes, probably even older than stem cryptophytes. That makes tertiary transfer of plastids from cryptophyte to haptophytes as impossible temporally as Bodyl et al. (2008) mistakenly claimed for the secondary transfer of red alga to the ancestral chromist (based on an almost certainly topologically wrong tree where Harosa and Hacrobia are not sisters and false assumptions about timing of early corticate divergences). Earlier stem cryptists must have been algae to make such hypothetical lateral transfer possible, but if they were algal, we have to accept more plastid losses within cryptists which reduces the perceived ‘benefit’ of postulating tertiary transfer in the first place. Halvarian and haptophyte periplastid derlins are paralogue B of probable red algal origin (Fig. S1) and so could not have come from cryptophytes late in evolution (as Bodyl et al. (2008) and Petersen et al. (2014) assumed) as cryptophytes long ago lost red algal derlin B and it is highly unlikely that both red algal derlins A and B would have been retained for an immense time with duplicate function lost much later after supposed tertiary transfers (similar argument to that used by Waller et al. (2016) but with the opposite conclusion to theirs). Postulating a tertiary transfer from one hacrobian to another is also valueless as the only supposed ‘benefit’ is a reduction in the number of early chloroplast losses (which have no evolutionary penalty and diverse benefits through heterotrophic feeding diversification). But cell biological complications and evolutionary difficulty of this imagined tertiary transfer are immense if they are to end up with exactly the same membrane topology as they started with: another example of ‘great investment of cell biological activity for a conspicuous lack of evolutionary change’ (Gould et al. 2015). Early branching of algae within Alveolata into two lineages (Dinozoa and Apicomplexa) and earlier algal divergence within Haptista mean that two chromophyte lineages radiated much earlier than the fossil record indicates. Late entry into the fossil record is purely a matter of fossilisability evolving later than chromophytes themselves: fossilisable coccolithophores branch shallowly within haptophytes and even more shallowly within the much older rappemonad/haptophyte clade; fossilisable thecate peridinea branch very shallowly compared with deeper branching Perkinsozoa, *Oxyrrhis*, *Chromera*, *Vitrella*, and Sporozoa, all with plastids; the fossil record is so temporally biased by lineage-restricted fossilisability that we do not need serial tertiary transfer to reconcile it with sequence trees.

Although sequence phylogeny firmly refutes my 1994 hypothesis of late intrachromist serial tertiary transfers (and all later variants, often unaware of it), other equally strong evolutionary arguments exist against it—especially against the widespread assertion/assumption that the karlodinian tertiary symbiosis somehow makes it plausible. Far from it, it makes it extremely implausible! This transfer of a 19-hexanoylfucoxanthin-pigmented chloroplast (Tengs et al. 2000) does not exemplify the kind of tertiary transfer envisaged (Cavalier-Smith et al. 1994). First, it was not to a heterotrophic host but mechanistically simpler chloroplast replacement (Saldarriaga et al. 2001)—easier as some preexisting host plastid-related genes could be retained and reused. That actually happened: the tertiary chimaera *Karlodinium micrum* (now renamed *Karlodinium veneficum*) kept genes from both its dinoflagellate host and its haptophyte symbiont (Patron et al. 2006), even though its plastid genome of 70 genes is entirely of haptophyte origin (Gabrielsen et al. 2011) and character, despite about ~ 40 being deleted, gene order being reshuffled, and some peculiar extrachromosomal DNA fragments (Espelund et al. 2012) with some analogies to standard dinoflagellate plastid DNA minicircles (Zhang et al. 1999, 2001, 2002).

Secondly, the tertiary plastid probably has only two surrounding membranes (Dodge 1975, 1989), so although initially surrounded by six membranes if taken up by phagocytosis or five if by myzocytosis, it probably lost three to four surrounding membranes. There is currently no evidence for the EpM, PPM, PR, PS, or derlin/Cdc48 periplastid protein import machinery; unlike haptophytes its outermost membrane apparently lacks cytosolic ribosomes, so it does not mimic chromist membrane topology as I postulated to be theoretically possible. So, nature tells us that such transfer is far harder than one might imagine. Proteins imported to the *K. veneficum* plastid have N-terminal topogenic sequences differing from the bipartite ones of other chromists (and all green secondary symbiogeneses) or typically unipartite ones of Plantae (Patron et al. 2006, using its old name *K. micrum*), which is unsurprisingly given its apparent reversion to two membranes only. Interpreting them is difficult and has been complicated by possibly erroneous assumptions that they are bounded by more than the standard plastid envelope two membranes, so algorithms were set to look for a bipartite sequence even though one expects to find only one grossly altered TP if there are only two bounding membranes as in Plantae (Patron et al. 2006).

Multiprotein trees decisively reveal that Karlodinia diverged from typical peridinean dinoflagellates before mutual divergence of peridinoid (thecate) and gymnodinoid (naked) subclasses, as well as showing thecate peridinoids to be one derived clade nested amongst early diverging naked clades (Bachvaroff et al. 2014; Janouškovec et al. 2017; Orr et al. 2012). Because of this and their unique chloroplast membrane topology, Table 1 establishes new peridinean subclass Karlodinia and Table S1 ranks peridinoids and typical gymnodinoids as infraclasses, grouping them as subclass Dinophycidae that includes all peridinin-containing dinoflagellates except *Amphidinium* that is put in a separate class Sulcodinea with *Gyrodinium* because of their earlier divergence and a different cingular pattern. I conclude from morphology (Gómez et al. 2005) and rDNA trees (Henrichs et al. 2011) that *Karenia* is a junior synonym of *Brachidinium* and formally transfer it to that genus, making Kareniaceae an invalid family name (Table 1). That also eliminates the problem of *Karenia* being invalid under ICZN as a junior synonym of a cicada.

## Karlodinian plastids may have only two bounding membranes

Secondary literature on the number of bounding membranes of Karlodinia is thoroughly confused by inaccurate citations of primary literature, which prevent understanding how they evolved and how their plastids import proteins and perhaps misled interpretation of their N-terminal topogenic sequences (Patron et al. 2006). Having checked numerous primary ultrastructural papers on all genera, my conclusion is essentially the same as Dodge’s (1989, p. 209): ‘The chloroplast envelope might consist of only two membranes although at present this is not clear’. The only difference is in the emphasis: I see no evidence at all for more than two and never see four as in euchromists, and doubt that there are three as is peridinin-containing dinoflagellates. As this conclusion is critical for evaluating tertiary symbiogenesis, this section elucidates the evidence.

Despite the clarity of Dodge’s statement, his paper was incorrectly cited as ‘This tertiary plastid is apparently bounded by three membranes’ (Patron et al. 2006) or in conjunction with Kite and Dodge (1988) as ‘ranging from two to four’ (Tengs et al. 2000). In the latter ‘two’ is accurate, but might ‘four’ stem from confusion with the symbiotic (not symbiogenetic) ‘dinotoms’ (dinoflagellates harbouring fucoxanthin-pigmented diatoms)? Sanchez-Puerta and Delwiche (2008) further simplified and misled by citing Tengs et al. (2000) and Dodge (1975) as indicating ‘a tertiary plastid surrounded today by only four membranes’. A fairly comprehensive but tendentious review of dinoflagellate tertiary symbioses (Gagat et al. 2012, p. 272) misleadingly wrote (no caveats!) ‘electron micrographs published by Steidinger et al. (1978), Kite and Dodge (1988), and Hansen et al. (2000b) indicate that three envelope membranes are present, at least in *K. veneficum* and *K. brevis*’, thus flatly contradicting Tengs et al. (2000). Let me document from primary papers my conclusion that these overconfident assertions of three or contradictorily four membranes may all be mistaken and that, published micrographs do not clearly show more than two membranes.

Hansen et al. (2000) cited to show three do not; their plates are too fuzzy and with too low magnification for counting membranes, and fixation imperfect: in the most magnified (Fig. 39) upper region the Norwegian ‘*Gymnodinium mikimotoi*’ [later *Karenia mikimotoi*; Daugbjerg et al. 2000, now *Brachidinium mikimotoi*] plastid envelope appears as a single dense line thick enough to represent the two typically closely appressed membranes of a standard plastid envelope, but probably not three or just one; certainly not four. Other figures of a Japanese strain and Fig. 40 of *Gymnodinium aureolum* [this was apparently not the true *Gymnodinium aureolum* which has peridinin and three envelope membranes, but a strain also called *Gyrodinium aureolum* later also considered *K. mikimotoi* (Hansen et al. 2000)] albeit of lower resolution are indistinguishable. Steidinger et al. (1978) also had only rather fuzzy low magnification plates of ‘*Gymnodinium breve*’ [later *Karenia*, now *Brachidinium brevis*, sister to *B.* (*K.*) *mikimotoi*] showing a single bounding line of the right thickness for a standard two-membrane envelope, but strictly uncountable. I could not access Kite and Dodge (1988) but as Dodge (1989) cited his own paper when concluding ‘only two’ membranes, I trust his judgement as an excellent electron microscopist more than those who misinterpreted both other papers. Furthermore, Kite and Dodge (1988) is on the same species as Kite and Dodge (1985) where Fig. 10 of ‘*Gyrodinium aureolum*’ [later considered *Karenia* (now *Brachidinium*) *mikimotoi*] also has a single dense uncountable line consistent with two appressed membranes, and they noted that the plastid DNA organisation was unique, with beaded bands of chloroplast DNA unlike haptophytes, other chromists, or plants, which could stem from it uniquely having both a large chromosome and minichromosomes. None of these primary papers mentioned the chloroplast envelope, but all are consistent with *Brachidinium* (*Karenia*) having a two-membrane chloroplast envelope and no PPM or peri-PPM ER or other membranes separating it from the cytosol.

That seems true also of *Karlodinium*, for which Bergholtz et al. (2005) studied closely related *Karlodinium armiger* and *K. veneficum*. The clearest micrograph I found for any Karlodinia is their Fig. 6 of *K. armiger* of one chloroplast totally devoid of PPM or EpM or vacuolar membranes with but a single dense bounding line, signifying an envelope of two appressed membranes but without sufficient magnification to resolve and count them. Figure 16 for *K. veneficum* yields the same conclusion. Dodge (1975, Figs. 9 and 10 for *Gym. micrum* (*K. veneficum*)) showed only one bounding line of the same thickness as the two appressed thylakoid membranes, thus probably just a two-membrane envelope. Only low magnification fuzzy micrographs of chloroplasts of the karlodinian genus *Takayama* seem available (de Salas et al. 2003) and give no reason for thinking that their envelope is any different from *Karlodinium* with which they group on rDNA trees.

Thus, all three karlodinian genera present a uniform face of ordinary plastid with a likely double membrane envelope and no trace of any PPM, ER, vacuolar, or additional membranes beyond those in Plantae. Despite evolving by one tertiary organelle transfer from a haptophyte (the only known tertiary transfer in the history of life), they apparently lost all trace of the usual euchromist membrane topology and thus fail to provide a model for or plausibility to the idea or tertiary transfers being able to mimic secondary symbiogenesis topology. They warn us to beware of such seemingly easy, but actually extremely complex and highly improbable ‘explanations’ of minor incongruences in insufficiently sampled sequence trees that are almost certainly purely technical not evolutionary problems (Cavalier-Smith et al. 2015a).

As no studies countably unambiguously resolve the presumed two plastid envelope membranes, fresh higher resolution studies are essential to test my conclusion and rule out the extremely unlikely possibility that Karlodinia retained only the inner membrane; though totally unprecedented for plastids and mitochondria, OM loss apparently happened for posibacteria (Cavalier-Smith 2006b) so is not impossible. Nonetheless, we can be certain that during or after its enslavement by the dinoflagellate common ancestor of Karlodinia, the haptophyte chloroplast escaped entirely from the enslaved haptophyte cell and any vacuole arising from its engulfment. Whether uptake was by myzocytosis or phagocytosis will never be known (though the host peduncle makes myzocytosis plausible) but is largely irrelevant as so many other membranes were probably lost additionally to the haptophyte plasma membrane. Once the chloroplast found itself in the host cytosol in the presence of the host plastid, existing nucleus-coded chloroplast proteins could immediately be used to implant translocons into it, supplemented by those of symbiont origin after its nuclear DNA entered the host nucleus [whether entirely by nuclear fusion (Cavalier-Smith et al. 1994) or uptake of DNA fragments]. The key problem was to modify host or symbiont bipartite sequences to a single neo-TP for transport across two membranes as in plants. Given that when haptophytes originated any plant Toc receptor proteins still present must already have been grossly modified during secondary symbiogenesis, as discussed above (also Cavalier-Smith 2003a), Karlodinia could not reinvent plant-like TPs—inevitably as complex evolution can never be reversed. Karlodinia instead cobbled together something that worked using uniquely peculiar topogenic sequences (Patron et al. 2006) as a compromise between the partially contradictory haptophyte and dinoflagellate systems they inherited, and which will be a biochemists’ nightmare to elucidate, especially as neither parental system is understood. Only about 90 haptophyte genes were identified as transferred to the dinoflagellate nucleus during this tertiary symbiogenesis (Burki et al. 2014), mostly but not all for plastid proteins. Koreny and Waller (personal communication) now have evidence in *Karlodinium veneficum* and *Brachidinium brevis* for two haptophyte-related derlin paralogues and two haptophyte-related Cdc48 paralogues, all apparently with the odd karlodinian bipartite targeting sequences suggestive of periplastid location, which makes fresh higher resolution studies of their membrane topology and number essential. If both proteins are genuinely plastid-associated, karlodinian plastids may have more than two bounding membranes, but not necessarily—because if periplastid derlins are indeed generally in PR, in theory it would have been possible for Karlodinia to have combined elements of both dinoflagellate and haptophyte membranes and targeting proteins (summarised in Fig. 5) without retaining a PPM or an EpM or the plastid being inside rough ER, e.g. by keeping PR, losing EpM and PPM, and targeting directly CV vesicles to the PR. Other scenarios are possible and must be tested by molecular cell biology.

## Time and place of chromist origin

Chromista are much older than the animal and fungal kingdoms, slightly younger than Plantae and neozoan Protozoa, but probably substantially younger than eozoan Protozoa that I consider the most ancient surviving eukaryotes (Fig. 2). There are more chromist fossils than for all other organisms combined. Especially abundant are foraminifera with calcareous shells and coccolithophorid haptophytes with calcareous scales that jointly built the chalk cliffs that line both shores of the English Channel and immensely thick Cretaceous strata underlying much of Northwest Europe—trillions upon trillions of chromist fossils. No Precambrian chromist fossils have certainly been identified. The oldest I accept are Cambrian: Foraminifera (~ 535 My old) and siliceous-skeleton Radiozoa (both subphylum Ectoreta of rhizarian phylum Retaria). Chromophyte body fossils unambiguously appear only after the Palaeozoic Era was ended by the end-Permian mass extinction [251 My ago (Mya)], the most extensive since eukaryotes began, extinguishing ~ 90% of all species—all trilobites, most corals, brachiopods, and land vertebrates—and amongst chromists, virtually all Radiozoa and the vast majority of foraminifera especially all the largest (e.g. fusulinids). Only four chromophyte algal lineages survived that largely volcanic, anoxia-causing holocaust (Benton 2003); after recovery, survivors radiated into empty niches, but representatives of only three lineages evolved readily identified fossilisable mineralised cell structures (Myzozoa: thecate dinoflagellates; Ochrophytina: diatoms, silicoflagellates, chrysophytes; Coccolithophyceae). Of heterotrophic groups other than Ectoreta, only ebriid Cercozoa have recognisable mineral fossils (hollow siliceous skeletons) which originated and diversified just after the second largest mass extinction (65.5 Mya end-Cretaceous) that extinguished dinosaurs and ammonites, thereby terminating the Mesozoic Era, though a few others can be identified in Mesozoic amber (ciliates, euglyphid Cercozoa). Very likely many of the Palaeozoic spiny acritarchs extinguished in the Permian (unassignable to any modern group) were cysts of chromophyte algae whose adaptive zone was taken over in the Mesozoic by novel chromophyte subgroups generated by surviving lineages, just as happened in animal phyla where we can identify their Palaeozoic representatives. In Ectoreta only can Mesozoic radiation of new classes from sparse survivor lineages be congruently documented by fossils and sequence-tree temporal patterns (e.g. for Foraminifera; Groussin et al. 2011).

Combining dates from fossils with multiprotein and rDNA tree proportions, I previously estimated that Chromista evolved no later than ~ 750 Mya, Plantae ~ 750–800 Mya, and neokaryotes ~ 800 Mya (Cavalier-Smith 2013b). Similar estimates for eukaryote age depend on knowing the position of the root of the eukaryote tree and correct identification of early supposedly ‘eukaryotic’ fossils, both highly controversial. Using only fossils I accept with reasonable confidence as genuinely from specific crown eukaryote groups, I previously estimated crown eukaryote age as ~ 850 My (Cavalier-Smith 2002b) or 900 ± 100 My (Cavalier-Smith 2006a) when supposing the root to be between scotokaryotes and corticates, but as 1000 ± 100 My if the root were between Euglenozoa and other eukaryotes (Cavalier-Smith 2013a) as earlier argued (Cavalier-Smith 2010). New protist discoveries and cytoskeletal information make me reconsider the root position, still I think within Eozoa: from the perspective of cytoskeletal evolution, the root is most likely between recently discovered *Tsukubamonas* (Yabuki et al. 2011) and all other eukaryotes (Fig. 2), as this free-living biciliate phagotroph has a much simpler cytoskeleton than excavates or discicristates (not attributable to secondary parasitic reduction). I now also think mouthparts and pellicles of Percolozoa and Euglenozoa share a common ancestry and Discicristata are probably a clade (as on derlin trees: Figs. S3, S4, S7, S8). Using this assumption and a new ribosomal 51-protein tree for reference, I elsewhere (in prep.) estimate the age of Chromista as ~ 730 My ago, slightly older than the 717 Mya onset of the Sturtian glaciation that initiated the Neoproterozoic snowball earth episode (Hoffman et al. 1998), and the age of crown eukaryotes as ~ 850–900 My ago.

The vast majority of marine phytoplankton are chromists, making them of immense significance for biogeochemical cycles: they generate a high proportion of atmospheric oxygen and fix much of the earth’s CO_2_, and a large fraction of marine carbonate sediments come from foraminiferan shells. They are globally climatically significant both as CO_2_ sink and because chromist algae are the only organisms that make dimethylsulphopropionate (for osmotic stability) which bacteria convert to volatile DMS eventually oxidised to cloud-nucleation particles. For these and other reasons, the origin of chromists with enhanced CO_2_ fixation and carbon burial might have diminished greenhouse effects sufficiently to have been the biological trigger postulated for the near-global kilometre-deep Neoproterozoic ice growth (Tziperman et al. 2011; Ward and Kirschvink 2015). I elaborate that possibility elsewhere, but now explain why I think chromists originated in the sea, whereas Plantae probably originated in fresh water or soil.

Corticates evolved from aerobic biciliate excavate zooflagellates, of which the closest to corticates is freshwater *Malawimonas*. However, both branches of eozoan Jakobea (likely the immediate outgroup to neokaryotes: Fig. 2) include marine and freshwater species, so one cannot safely infer their ancestral habitat, though *Tsukubamonas* being freshwater makes that slightly more likely; thus, early eukaryote evolution including the origin of the excavate groove was possibly in fresh water or soil. Glaucophyta, the most primitive Plantae, are entirely freshwater, but Rhodophyta and Viridiplantae each split basally into ancestrally freshwater and probably ancestrally marine clades; fewer habitat switches need be invoked if we regard Plantae as ancestrally freshwater organisms. Of the two basal clades of Viridiplantae, Streptophyta are entirely freshwater except for derived mangroves and seagrasses; the deepest branches of its sister phylum Chlorophyta are marine, but there are derived freshwater lineages. Within Rhodophyta, the exclusively freshwater branch of red algae (thermophilic subphylum Cyanidiophytina) is probably irrelevant to chromist origins, as most chloroplast multigene trees suggest that the red alga enslaved to make chromists was the earliest offshoot of its sister subphylum Eurhodophytina that are almost all marine [though a tree using nucleotides not amino acids raises the possibility that chromist plastids are sister to all red algae and originated even before the primary red algal bifurcation (Kim et al. 2015); this needs critical restudy by evolutionarily more realistic site-heterogeneous whole-genome trees]. Most likely an early marine red algal unicell was enslaved by a marine planktonic corticate zooflagellate, which diversified to produce the four major chromist clades each probably ancestrally marine, and later multiply colonised freshwater.

One way of diversifying was in photosynthetic accessory pigments which became very different in different subgroups from each other and from those of red algae and other plants, allowing photosynthetic specialisation across the light spectrum in different ecological zones, as Supplementary Discussion 1 (SD1) explains. The other major mode of chromist diversification was through modifying the cytoskeleton in many innovatory ways, as explained in the rest of this paper. That allowed both phototrophs and heterotrophs to exploit different adaptive zones from other eukaryote kingdoms through evolving entirely novel types of organism. In Hacrobia and Heterokonta, a majority of early branching lineages are marine. In alveolates, all deep branching dinozoan classes are marine (only some dinokaryotes are freshwater) as are some Colponemea; chromeroids are exclusively marine; thus, the ancestral myzozoan alga was probably marine. Ciliates and Cercozoa have a large mix from both habitats, making it hard to infer their ancestral one, but Retaria were probably ancestrally marine as are most of their deep branches, Ectoreta almost exclusively so.

## Phylogenetic unity of Halvaria: heterokonts plus alveolates

Infrakingdom Halvaria was established to embrace heterokonts and alveolates (Cavalier-Smith 2013a), the name proposed by Cavalier-Smith (2010) who considered them a clade as first indicated with decisive statistical support by site-heterogeneous 135-protein trees (Burki et al. 2008, 65 eukaryotes). That heterokonts and alveolates are sisters was first weakly hinted by maximum likelihood 18S rDNA trees (Cavalier-Smith et al. 1994) and is strongly supported by more richly sampled site-heterogeneous multiprotein trees (Burki et al. 2009, 2010, 2012, 2016; 162–258 proteins; Cavalier-Smith et al. 2014, 2015a, b, 2016; 187–189 proteins). However, the first sparser multiprotein trees with strong statistical support for chromist subkingdoms Harosa and Hacrobia both being clades, using the evolutionarily less realistic site-homogeneous algorithms only (Burki et al. 2007; 123 proteins, 49 eukaryotes), grouped heterokonts with Rhizaria instead. One discordant study oddly found that 27-protein and 34-protein trees (only 44 corticate taxa) grouped alveolates and Rhizaria as a clade; the authors curiously claimed that the Halvaria clade found with maximal support on their site-heterogeneous 147-protein trees (like everyone else) is a long-branch artefact (He et al. 2016). That remarkable claim was based on the erroneous assumption that the method used to discard the majority of the data to get the topologically inconsistent 27/35-protein trees removed the longest branch sequences. In fact, as I explain in detail elsewhere (submitted), it generated a biased small sample with alveolates and Rhizaria the two longest branches on the tree, which artefactually grouped together; it probably removed most genuine phylogenetic signal! A site-heterogeneous tree for 42 eukaryotes using 478 proteins (selected for the absence of paralogue complications) found maximal posterior probability support for Halvaria and Harosa both being clades (Ren et al. 2016); on that tree, heterokonts have the shortest branch within Harosa so their maximally supported grouping with the long-branch alveolates cannot be a long-branch artefact—however, the deeper branching of systematically longer-branch Harosa than short-branch Hacrobia on that tree could be a long-branch artefact (Cavalier-Smith 2009b, Cavalier-Smith et al. 2015a). Though I myself once suggested that the Halvaria grouping might be a long-branch artefact (Cavalier-Smith 2009b), the weight of evidence now strongly argues against that.

There are no morphological arguments against Halvaria being a clade or for Rhizaria being sister to alveolates. Despite consistent support from every site-heterogeneous tree using >100 proteins for Halvaria being a clade, no shared morphological character has been identified unique to them. That is unsurprising as the shared stem on sequence trees is relatively short, implying that alveolates and heterokonts diverged close to the origin of Harosa ~ 730 My ago; there is no reason why a shared character so important as to never have been lost since should have originated in that short time interval. Compared with their sister infrakingdom Rhizaria, which at the outset evolved filopodia/reticulopodia unique to it and a benthic surface-associated lifestyle, earliest Halvaria were more conservative cytoskeletally and retained a compact biciliate flagellate lifestyle, swimming in marine plankton like the ancestral photophagotrophic chromist.

The three deepest halvarian branches diverged greatly in how they exploited this broad adaptive zone. Most conservative was basal miozoan subphylum Protalveolata comprising eukaryovorous colponemean flagellates (Colponemida and *Palustrimonas*) and *Acavomonas* (Table 1) of which only *Colponema* with hairy anterior cilium and toxicyst extrusomes is ultrastructurally studied (Mignot and Brugerolle 1975; Tikhonenkov et al. 2014). Like the excavate ancestors of all corticates, *Colponema* retains a ventral feeding groove with associated posterior cilium bearing a single vane to increase the water current for sweeping prey into its groove. The vane is ventral as in the neoloukan *Malawimonas* (O’Kelly and Nerad 1999) a deeply diverging branch of scotokaryotes, the sister clade to corticates (Cavalier-Smith et al. 2015a), not dorsal as in the arguably phylogenetically more distant Jakobea. The vane was lost five times independently in other corticates that adopted radically novel feeding modes, many photosynthetic. Like malawimonads, *Colponema* are not diverse in species (six known, from marine or fresh water or soil; Tikhonenkov et al. 2014) but represent an ecologically viable small adaptive zone and ancient organismal type (‘living fossil’) that is a key to understanding chromist cytoskeletal evolution. Class Colponemea comprises two deeply divergent clades: *Colponema* possibly branching more deeply than also ventrally grooved but more elongated hypersaline specialist *Palustrimonas* on 18S rDNA trees that placed *Acavomonas* and then Colponemea as immediate outgroups to Myzozoa (Park and Simpson 2015). rDNA/Hsp90 three-gene trees not including *Palustrimonas* also grouped *Acavomonas* with Myzozoa but probably misleadingly put *Colponema* a node lower than did 18S rDNA as the most divergent alveolate of all (Janouškovec et al. 2013). We cannot yet be sure that Protalveolata as defined in Table 1 are directly ancestral to Myzozoa (as trees timply) not their sisters, as all three published trees have contradictory topology, but there is no reason to consider any myzocytotic rather than phagocytic. Therefore, Cavalier-Smith (2013b) removed Colponemea from Myzozoa, restricting Protalveolata to this class, and reduced Myzozoa to a subphylum within the older phylum Miozoa, taxonomic acts overlooked by Tikhonenkov et al. (2014) who likewise removed Colponemea but unnecessarily made new phyla for *Colponema* and *Acavomonas*; using such high ranks was taxonomically unwise and not justified by the phenotypic differences between them and Myzozoa, which subphylum rank adequately emphasises; until we have ultrastructure for *Acavomonas*, we cannot even be confident that they should be excluded from Myzozoa or Colponemea—Table 1 provisionally accepts Acavomonadea as a distinct class in Protalveolata not Myzozoa.

## Cytoskeletal variants define protist body plans

Different protist body plans are largely defined by the microtubular (mt) cytoskeleton associated with centrioles (ciliary basal bodies) and the more amorphous non-actin fibrillar proteins linking these to each other and to other cell organelles. Their evolution shows marked conservatism with many features constant over hundreds of millions of years, this remarkable stability punctuated by major shifts that generate superficially radically different phenotypes, but which when critically evaluated usually show major modifications of preexisting structures during radical shifts in feeding mode (Cavalier-Smith 2013b). It is now generally accepted that the cenancestral eukaryote had two cilia whose centrioles are linked by one or more specific connectors (Cavalier-Smith 2014b). The two centrioles are of unequal age, the ancestrally anterior one being younger (designated 2; Heimann et al. 1989) and the ancestrally posterior mature one (labelled 1) assembled one or more cell cycles earlier. For brevity, I use C1 to designate mature cilia and centrioles and C2 for the younger ones whose structure and beating pattern often differ. By establishing which is which, one can determine homologies across phyla of the roots that anchor centrioles in cells, both mt (Moestrup 2000) and fibrous (Cavalier-Smith 2013b; Heiss et al. 2013a, b; Yubuki et al. 2013). Some C2 roots are known to transform into dissimilar C1 roots during centriolar transformation whereas others disassemble and fresh different roots replace them (Perasso et al. 1992); partial disassembly may also occur.

**Fig. 6:**
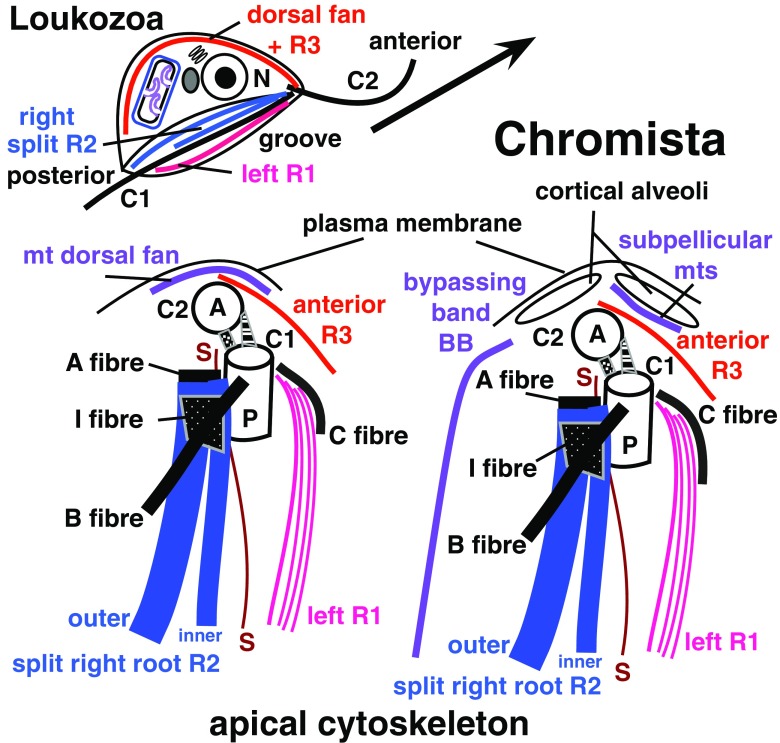
Cytoskeletal innovations during corticate and chromist origins. *Left* diagrams summarise the ancestral condition in excavate ancestors of corticates as represented by the loukozoan *Malawimonas*. *Upper* shows the whole cell seen from the *right* with the feeding groove tilted obliquely to show *left* and *right* mt roots (*R1*, *R2*) that support feeding groove rims and floor. Younger anterior cilium (*C2*) with oar-like beat and older posterior cilium (*C1*) undulating from base to tip simultaneously propel the cell forward (*arrow*) and waft food into the groove for ingestion. *Lower left* (Loukozoa) and *right* (ancestral Chromista) diagrams view the cell apex from the ventral side (so the cell’s right is on the left) to show mt arrays (colour: mt bands *R1*–*R3*; plus a dorsal fan of diverging mts that support the cell’s dorsal surface) and associated fibrous supports (*black*: *A*–*C*, *I*). The orthogonal centrioles (anterior A, posterior P) are interconnected by asymmetric linkers and in Loukozoa (*left*) a dorsal mt fan and anterior left mt band (*R3*) connect C2s to the apical dorsal plasma membrane. R3 is developmental precursor of R1. The ancestral corticate interposed novel cortical alveoli between the plasma membrane and dorsal fan, which split into a right bypassing mt band (BB) and numerous single, diverging subpellicular mts attached to alveolar inner faces. Chromists (*right*) initially kept all these cytoskeletal components, modifying them as centrioles moved subapically as the text explains. Their sister Plantae lost BB, the R2 outer branch, and B fibres. A second anterior right root R4 (not shown; see text) evolved polyphyletically by heterochrony in several chromist and plant lineages as a simplified developmental precursor of R2 (1 or few mts). The text argues that developmentally and evolutionarily the singlet root (*S*, *brown*) is a specialised R2 subcomponent, not a third posterior root as traditionally assumed. Dorsal fan and apical mts are actually longitudinal (as shown for BB only); the *purple line* symbolises a cross section of their mt arrays

Direct evidence of root transformation or replacement requires arduous and rarely achieved electron microscopy of predivision cells when centrioles and roots are being duplicated. To establish root homology distinguishing C1 and C2 is insufficient; one must also allow for changes in mutual orientation of centrioles (ancestrally orthogonal, multiply derived parallel, rarer antiparallel) and rotation on its axis of C2 compared with C1, and use conserved ultrastructural markers (typically distinctive fibrous roots attached laterally to C1 roots, dorsally or ventrally). Centrioles are chiral, every triplet being different and attached to different specific fibrous structures some of which connect to a specific mt root, but an absolute numbering system (likely to be universal) and recognition of virtually all attachments has been achieved only for the green alga *Chlamydomonas* (Geimer and Melkonian 2004) whose centrioles are mutually rotated by 180° and roots have 180° rotational symmetry in ultrastructure (anterior right the same as posterior left and anterior left the same as posterior right) but differ in age and, in which organelles, they attach to (e.g. eyespot, mating structure; Holmes and Dutcher 1989) and in age and ultrastructurally hidden protein markers that render them strictly asymmetric (Mittelmeier et al. 2015) as is the distal acorn structure of centrioles (Geimer and Melkonian 2004). All green plants (Viridiplantae) have 180° centriolar mutual rotational near symmetry, but this is a derived condition found in few other groups [arguably the heterokont Synuridales with secondarily parallel centrioles, e.g. *Mallomonas* (Beech and Wetherbee 1990) and the heterokont oomycete zoospore]. Discicristate centrioles perhaps uniquely both have the same orientation (Brugerolle 1992; Brugerolle and Simpson 2004). Most biciliate lineages, however, appear to have an axial rotational angle of about 90° between C1 and C2, making root geometry markedly more asymmetric: that is true for most chromists, and their ancestral state and probably that for the eukaryote cenancestor. It is generally easier to identify left and right posterior roots correctly; partly because right root R2 ventral face (ventral means facing the ciliary groove if present) has a highly distinctive laminated I fibre and left R1 has a differently laminated C fibre on its dorsal face (both present in most excavates and in some derived Sulcozoa and chromists), but even when one or both is absent, having two opposing roots helps define them relative to the cell’s body axes, all ciliated cells being deeply chiral in cytoskeletal organisation. Identifying anterior roots is harder, especially in numerous lineages with only one, where incorrect assumptions about centriole axial rotational symmetry have led everyone writing on this (including me) to make some errors, and confuse R3 (which in cryptomonads transforms into R1 in the next cell cycle; Perasso et al. 1992) with R4, which I try to correct here. Figure 6 contrasts the centriolar roots of chromists and Plantae and their joint excavate ancestors.

Sorting out these homologies was extremely tedious and time consuming but centrally important for defining body plans of eukaryote groups. Centriole-associated skeletons can be as powerful as sequence trees (often more so) for elucidating relationships and recognising clades, just like vertebrate bones or arthropod exoskeletons, and are crucial for cell evolution and systematics (Cavalier-Smith 2000a, 2013b). When one gets both right, there is remarkable congruence between sequence trees and ciliary and centriolar root defined body plans, mutually reinforcing their validity and credibility. Whenever they disagree, both must be critically reevaluated to identify errors. Earlier, euglenozoan and excavate roots were incorrectly labelled (Moestrup 2000; Simpson 2003), but after that was recognised (Cavalier-Smith and Karpov 2012), there is complete agreement between excavate and other specialists exemplified by the identical independent assignment of roots to the sulcozoan *Apusomonas* by Cavalier-Smith (2013b) and Heiss et al. (2013b) and broad agreement about other Sulcozoa, Amoeboza, excavates, and heterokonts between these papers—not perfect; because they had access to new data for *Breviata* and *Thecamonas*, that of Heiss et al. (2013b) is better than mine in a few respects, though I suspect my identification of the planomonad anterior root as R3 may be better and doubt whether any scotokaryotes have R4. However, I now think that anterior roots (AR) of Eolouka which stem from the posterior edge of C2 are probably not homologous with R3 of Loukozoa (*Malawimonas* plus Metamonada) and neokaryotes generally which start between its anterior edge and the dorsal fan (if present, as it is in most excavates sensu stricto—i.e. Loukozoa plus Jakobea, with an homologous feeding groove, and Sulcozoa with modified groove; Cavalier-Smith 2013b; Heiss et al. 2013a, b). Possibly, the eoloukan anterior root transforms into R2 unlike neokaryote R3 that transforms into R1 (absent, arguably primitively, in *Tsukubamonas*) and unlike the anterior root of Discicristata which transforms into the intermediate root (Brugerolle 1992; Farmer and Triemer 1988) every cell cycle (and may or may not be homologous with neokaryote R3/R1). Though it appears positionally like R4 of corticates and may attach to the same triplet, the absence of R4 in scotokaryotes and in the cytoskeletally apparently most primitive members of all major corticate lineages leads me to think that roots at this position arose polyphyletically within corticates and within chromists and independent of Eolouka whose positionally equivalent anterior root I is therefore call R4e (Fig. 2). Such parallel multiple origins of R4-position roots is mechanistically plausible as it simply entails assembling an R2 protoroot one cell cycle earlier than R2 normally assembles, not evolutionarily onerous. The independent origin of neokaryote R3 and the discicristate dorsal root on probably the same triplet inferred on Fig. 2 can be viewed as comparably parallel developmental heterochrony.

## The chromist bypassing microtubule band

Chromists and their sister group Plantae evolved from excavates following the origin of cortical alveoli. As a result of detailed reevaluations of cytoskeletal evolution in Dinozoa and Apicomplexa to be published elsewhere and a similar reevaluation of hacrobian skeletal evolution (Cavalier-Smith et al. 2015a), I have realised that a major cytoskeletal character distinguishes Chromista from Plantae and all other eukaryotes. This is a band of stable mts that, unlike most others, is not attached at one end to the centrioles and thus not a centriolar mt root; as it bypasses both centrioles on the cell’s right, extending from near the cell apex, I call it the bypassing band (BB). I suggest it evolved from the excavate dorsal mt fan that is absent in chromists. The only other similar band is the apusomonad *ribbon*, also proposed to have evolved from the dorsal fan (Cavalier-Smith 2013b; Heiss et al. 2013a). As Sulcozoa other than apusomonads and Mycetozoa have dorsal fans (Cavalier-Smith 2013b; Heiss et al. 2013a, b), the apusomonad ribbon and chromist BB evolved separately by parallel evolution from an homologous ancestor, so BB is the first recognised cytoskeletal synapomorphy for Chromista, strongly supporting their being a clade. Figure 6 shows this key difference between cytoskeletons of chromists and the loukozoan *Malawimonas* that today best represents the excavate ancestor of corticates.

In *Malawimonas* and *Breviata*, the rightmost part of the dorsal fan between R3 and C2 is more ribbon like than the left portion with closer, less divergent mts (O’Kelly and Nerad 1999; Heiss et al. 2013a). I regard the ribbon-like part as the morphogenetic core of the fan as it duplicates first in *Breviata* (Heiss et al.’s 2013a Fig. 6G), and suggest that the apusomonad ribbon and chromist BB both evolved specifically from this part and that the more fan-like left parts became subpellicular mts (see later sections). This contrast of the left and right parts of these scotokaryote fans is not obvious in the more uniform and simpler jakobid dorsal fans; in the probably ancestral non-loricate genera (Lara et al. 2006; Patterson 1990; Simpson and Patterson 2001), their mt numbers are similar to the core part only of the scotokaryote fan, so I suggest this is also historically older and the more divergent leftward mts were only added during the origin of neokaryotes. Loricate *Reclinomonas* has a broad dorsal ribbon of ~ 40 closely linked mts that do not diverge distally yet were inappropriately called a fan (O’Kelly 1993). The apusomonad ribbon supports the right edge of its groove, and BB is also on the chromist cells’ right, but in the scotokaryote excavate *Malawimonas*, the dorsal fan is predominantly to the cell’s left (O’Kelly and Nerad 1999). By contrast, about half the more symmetric and more ribbon-like jakobid ‘fan’ is on the right. The ancestral neokaryote’s wider fan was presumably also symmetrically on either side of C2 as are the dorsal fans of *Breviata* and Mycetozoa (Heiss et al. 2013a, b), which represent the ancestral state of Sulcozoa better than the derived apusomonad ribbon (I therefore hereby formally transfer Breviatea from subphylum Apusozoa to subphylum Varisulca—also more consistent with multigene trees; Brown et al. 2013; Cavalier-Smith et al. 2014, 2015a). For reasons explained elsewhere (submitted), Fig. 2 assumes pellicle mts of Percolozoa to be homologous with the posteriorly nucleated euglenozoan pellicle mts rather than the probably anteriorly nucleated dorsal mt fan of excavates and podiates.

Later sections outline how BB and R2 were adapted by diverging chromist lineages for a huge array of different cytoskeletal structures to facilitate diverse new feeding strategies. These all involved the cell projecting anteriorly beyond the centrioles, for which BB provided the essential support. By contrast, excavates like jakobids and *Malawimonas* ancestrally had no anterior cytoskeleton: centrioles were at the cell’s very apex and all cytoskeletal mts directed backward, including the dorsal fan attached to C2 whose mts must be antiparallel to centriolar ones, and which was inherited by corticates together with all centriolar mt bands from a *Malawimonas*-like ancestor before chloroplasts evolved. Plantae lack BB so Chromista could not have evolved from a plant ancestor, which have very different cytoskeletons, whose homologies have been partially misinterpreted, especially in glaucophytes, as supplementary discussion SD2 explains. Plant cytoskeletons are highly derived compared with excavates and chromists because all except the tetraciliate prasinophyte green algal class Pyramimonadophyceae (e.g. *Cymbomonas*; Burns et al. 2015) abandoned phagotrophy and focused entirely on photosynthetic nutrition. Yet even plants betray their excavate ancestry (see SD2, which also establishes new order Cyanophorales).

## BB preadapts chromists for evolving axopodia

The BB may be the major reason why the actinopod feeding mode using axopodia (mt-supported slender radial cell projections) evolved only in chromists and did so independently in five phyla and more than once in two [heterokont Gyrista—actinopod heliozoa (Cavalier-Smith and Scoble 2013) and pedinellids; Cercozoa—Phaeodaria and desmothoracids]. Cavalier-Smith and Chao (2012) summarised chromist axopodial diversity and Centroheliozoa in detail, and Cavalier-Smith et al. (2015a) explained their polyphyly in Hacrobia. I suggest that having a BB not directly connected to centrioles mechanistically facilitated the polyphyletic origin of axopodia in a way impossible for Protozoa. Pedinellia (e.g. *Pteridomonas*; Patterson 1985) are the only vegetatively flagellate chromists to have entirely lost centriolar roots when losing the posterior cilium and evolving periciliary axopodial feeding; I suggest they were able to do so by using the multiply duplicated non-centriolar BBs to make a circlet of 3-mt axopodia around the remaining anterior cilium whose water currents drew in prey to them. I earlier suggested that actinophryid axopodia evolved independently by multiplying raphidophyte rhizostyle mts (Cavalier-Smith and Scoble 2013), arguing that the rhizostyle is a composite of a standard root R2 and a non-root (nucleus- and PM-associated) mt structure perhaps antiparallel to it, which I now suggest is a BB (see fuller supplementary discussion SD10). Of the four main chromist lineages, only alveolates never use BB-derived axopodia in that way: Ciliophora lost BB through focusing on multiplying kinetids to make giant multiciliate predators, whereas Myzozoa used BB as ancillary to a novel feeding mode—myzocytosis, which in Apicomplexa became the apicomonad pseudoconoid and sporozoan conoid. Of flagellate alveolates, only Colponemea clung to the old excavate ways of ciliary groove feeding and thus remained similarly lacking in biodiversity.

As Cavalier-Smith (2013b) explained, the single anterior centriolar root supporting the dorsal surface and posterior centriolar mt roots that support the groove of *Colponema loxodes* are identical to those of *Malawimonas* (anterior R3, posterior R2 + S and R1) except that R2 supporting the groove rim lost its outer branch. The central singlet (S) at the base of the posterior groove is also present in *Colponema vietnamica* (Tikhonenkov et al.’s 2014 Fig. 5C) implying it to be a universal feature of *Colponema*. Tikhonenkov et al. not only overlooked S but also that the fibrous band is positionally and ultrastructurally an I fibre like that of *Malawimonas*, a rare example of an alveolate I fibre, supporting the thesis that *Colponema* represents the ancestral cytoskeletal and ciliary condition for alveolates and chromists generally (Cavalier-Smith 2013b).

By the standard definition of excavates as protists with such homologous feeding grooves (Simpson and Patterson 1999), *Colponema* should be included in excavates, but it never is (sensibly) because unlike excavates, it possesses also cortical alveoli just like those of other alveolates. That illustrates the point that to define a paraphyletic group like excavates (certainly not a clade; Cavalier-Smith et al. 2014, 2015a, b, 2016), one must specify both its ancestral morphological innovations (in this case, the groove cytoskeleton) to include members and the later innovations unique to each excluded derived group (in this case, cortical alveoli to exclude corticates, and ventral pseudopodia and gliding motility to exclude Sulcozoa and their amoebozoan and opisthokont descendants; Cavalier-Smith 2013b). Recognising a paraphyletic group like Colponemea or Eozoa or excavates is evolutionarily valuable as it tells us the ancestral phenotype of derived groups like Myzozoa, Sulcozoa, or Chromista (Cavalier-Smith 2013b). Unlike *Malawimonas* or other excavates, *Colponema* has a row of simple protein hairs on its anterior cilium in the same relative position as the vane on the posterior cilium.

## Alveolate ciliary and cytoskeletal diversification

Of key importance for understanding alveolate evolution are free-living flagellate relatives of parasitic Sporozoa classified as parvphylum Apicomonada in infraphylum Apicomplexa (Table 1). Apicomonads include the chromeroid algae *Chromera* and *Vitrella* and a large array of myzocytotic predatory zooflagellates such as *Colpodella*, whose structural diversity was grossly underestimated until Cavalier-Smith and Chao (2004) tried to improve their classification, but which are sufficiently uniform cytoskeletally for all to be included in one class, Apicomonadea, which ancestrally were photosynthetic myzozoan predators sharing some plastid features with dinoflagellates but divergent in others. Subsequent ultrastructural and sequencing work has confirmed that many organisms were excessively lumped under the name *Colpodella* including one really a primitive dinoflagellate not even an apicomplexan (i.e. the new genus *Colpovora*, closely related to *Psammosa*, here grouped with it in new dinoflagellate class Myzodinea: see supplementary discussion SD3 on myzozoan ciliary and cytoskeletal evolution for details and references). Critically reexamining the evidence also reveals numerous misidentifications that previously prevented rational understanding of apicomonad evolution and has allowed a further improved classification concordant with ultrastructure and sequence phylogeny (Table S1). Supplementary discussion (SD4) disentangles confusions and explains reasons for these innovations, allowing a better explanation than hitherto of homologies of apicomonad cytoskeletons and their relationship to those of other alveolates.

Table 1 summarises the new synthesis. Important is the recognition that *Vitrella* merits separation at the subclass level from other apicomonads, that there have been multiple losses of apicomonad photosynthesis and radical changes in their cytoskeleton, and that the concept of a pseudoconoid has been far too loose, and a new concept of a ‘paraconoid’ restricted to *Colpodella* sensu stricto is needed. Implications for evolution of pseudoconoids and conoids and the origin of Sporozoa are discussed in supplementary SD5, where I argue that conoids/pseudoconoids evolved from BB by making its apical nucleation centre annular and discuss the preconoidal significance of the ciliary/centriolar-related protein SAS-6L. Supplementary SD6 explains how ciliate kinetids, like those of Myzozoa, reflect an excavate origin of the chromist cytoskeleton. Dinoflagellate cytoskeletons are less uniform than sometimes thought, and important differences are used here to revise the higher classification of deep-branching dinoflagellates (Tables 1, S1); major differences within Dinozoa are better understood by comparison with apicomonad skeletons and as excavate derivatives following the origin of BB, rather than by comparisons only with phylogenetically more distant heterokont algae and plants as before; supplementary SD6 discusses aspects of dinoflagellate cell evolution (correcting some cytoskeletal misinterpretations) including comparison of cytoskeletal divergence with multiprotein sequence trees, whose congruence confirms the importance of early marked cytoskeletal and nuclear organisational divergences.

## Excavate origin of the halvarian cytoskeleton

Cavalier-Smith and Chao (2010) pointed out that many early diverging heterokonts have a split right centriolar mt root (R2 in the corrected nomenclature; Cavalier-Smith and Karpov 2012) that is positionally homologous to and probably descended from that of excavates. Cavalier-Smith (2013b) noted that all mt roots of *Malawimonas* and jakobid excavates can be identified in Bigyra. This is beautifully exemplified by *Platysulcus* that exhibits almost all mt roots seen in other heterokonts. It has all three posterior roots (split right R2; central singlet or S mt; left R1). R2 is curved in transverse section, and fibres closely resembling an unusually narrow and short excavate I fibre are present in its ventral concavity (Shiratori et al.’s 2015 Fig. 3D, E, which also show an apparent bilaminar ribbon-like B fibre). Whether a C fibre is associated with *Platysulcus* R1 is unclear, but the density near its base in their Fig. 5E suggests one is present at least proximally, in which case apart from the absence of ciliary vanes that some excavates secondarily lost, *Platysulcus* would fully qualify for being called an excavate if anyone wants to stick to the original loose definition (no longer useful I think).

*Platysulcus* confirms that the excavate concept (Simpson and Patterson 1999) has lost all taxonomic utility it once seemed to have (Cavalier-Smith 2002b): recognising that, Cavalier-Smith et al. (2015a) abandoned Excavata as a taxon. The hypothesis that all taxa considered by Simpson (2003) to be excavates are a clade that excludes all other eukaryotes has been decisively falsified: both by multiprotein trees and by a far wider variety of eukaryotes having been discovered to have homologous ventral grooves, ranging from Sulcozoa, though *Colponema* to *Platysulcus*. O’Kelly (1993), originator of the excavate concept (not its name: Simpson and Patterson 1999), believed them to be the ancestral condition for all eukaryotes. His thesis would be confirmed if the eukaryote tree’s root were within excavates (e.g. Derelle et al. 2015), but if between groove-less Euglenozoa and excavates (Cavalier-Smith 2010), excavates would be the ancestral state for all eukaryotes but Euglenozoa. I now think the root is most likely between *Tsukubamonas* and all other eukaryotes and that Discicristata are a clade as ribosomal protein trees suggest (Raymann et al. 2015), but do not regard the *Tsukubamonas* and percolozoan *grooves* as strictly homologous with those of excavates, though some subcomponents are; centriolar root structure is consistent with Eozoa being ancestral to neokaryotes with Jakobea their sister (Fig. 2). Anyway, excavates are ancestral to both podiates and corticates and therefore to chromists. Even if Eozoa were a clade and sisters to neokaryotes (He et al. 2014) or contradictorily to corticates (Derelle et al. 2015), excavates remain paraphyletic and chromists evolved from an excavate ancestor. Simpson now accepts that sulcozoan and heterokont roots are derived from and retain many features previously thought to be specific for excavates (e.g. Heiss et al. 2013a, b), i.e. accepts that excavates are paraphyletic but has not yet accepted that Eozoa are also. The excavate concept as refined here retains great utility, as defining an important ancient grade of organisation that preceded those of podiates and corticates in evolution, making its recognition a major advance in cell evolution.

Unlike excavates and ancestral chromists, *Platysulcus* has an extra mt root (R4) on C2’s ventral side. R4 is widely present in heterokonts (e.g. most but not all Bigyra, and in most Gyrista) but is probably not strictly homologous to R4 of other chromists or Plantae. Retronemes not only reverse propulsive thrust but also increase its power, which can be further increased by elongating the cilium greatly compared with the ancestral excavate-like halvarian. I suggest the heterokont R4 evolved when that happened, to better anchor the anterior cilium and reduce its chance of being broken from the cell body when its power dramatically increased. That accounts for the ancestral presence of R4 in heterokonts only, unlike the other three chromist groups which ancestrally had only the single excavate-derived R3 (Fig. 6); the only other chromist groups to evolve an R4 are coccolithophyte haptophytes when they evolved a long contractile haptonema to catch prey (Cavalier-Smith et al. 2015a) and eudinean dinoflagellates that evolved a transverse groove. R4 evolved also in Plantae, probably independently in Glaucophyta and Chlorophyta. Independent origins of R4 were mechanistically easy by heterochrony, as R4 is serially homologous with R2; initiating R2 assembly one cell cycle earlier would make R4, which would develop into R2 (or perhaps when R4 is a singlet, as it often is, to the R2-associated singlet only) in the next cell cycle. Raphidomonadea lost both R1 and R4 but are the only ochrophytes to retain I fibres on R2; R2 and/or BB probably had a key role in originating actinophryid pseudoheliozoan axopodia for a novel mode of feeding (Cavalier-Smith and Scoble 2013), as supplementary discussion SD10 explains. Centriolar roots were commonly lost in heterokonts, notably in those that lost cilia (e.g. the bigyran *Blastocystis* or coccoid ochrophytes) or suppressed them in vegetative photosynthetic phases (e.g. diatoms; independently lost by Pedinellia when losing just the posterior cilium and groove and evolving symmetric axopodia for catching prey instead). That illustrates my argument that virtually all major changes in chromist cytoskeletons can be understood as concomitants and mediators of radical shifts in feeding mode (also true of such changes in Protozoa: Cavalier-Smith 2013b).

Raphidophytes apparently have broad R2 I fibres; I now realise that Developea R2_i_ has a narrow I fibre (Aleoshin et al. 2016). Recently, I identified I fibres in Hacrobia (Cavalier-Smith et al. 2015a) and so concluded that three main chromist lineages retained I fibres from their excavate ancestor and only some sublineages lost them after early divergences. This chromist situation is analogous to that in Sulcozoa where diphylleids arguably retained I fibres and in apusomonads and planomonads R2 became split in different ways from excavates (multiply in planomonads; Heiss et al. 2011, 2013a, b); these changes happened when Sulcozoa lost the ancestral excavate feeding mode because their ancestor evolved posterior ciliary gliding and lost the mechanistically incompatible posterior ciliary vane, evolving a new dorsal theca making a partially preciliary cytoskeleton (Cavalier-Smith and Chao 2010; Cavalier-Smith 2013b).

## Origin and evolutionary significance of BB: corticate mt/membrane innovation

Previously, I argued that a double mt band (C-shaped or U-shaped in cross section, one curved mt row nested within the other) was a unique synapomorphy for Hacrobia and evolved from excavate split R2’s outer branch by the split becoming complete and the nucleation point of R2_o_ moving forward anterior to both centrioles (Cavalier-Smith et al. 2015a). This hacrobian double band is positioned precisely as the halvarian single BB and thus also a BB. It is unlikely that these structures evolved independently as no protists outside Chromista have a BB. I therefore now argue that the outer C of the hacrobian double BB is homologous to the single BB of Halvaria, so only the extra inner C is a synapomorphy for Hacrobia. If so, the outer C (BB) originated in the ancestral chromist and a BB comprising one mt band is a synapomorphy for all Chromista (supplemented by a second inner band only in hacrobia). As suggested above, BB could have evolved from the ribbon-like part of the excavate dorsal fan when it detached from C2. As a later section explains, this interpretation also allows identification for the first time of a homologue for ventral posterior ‘root’ 2 (vpr2) present in all well-studied ventrofilosan Cercozoa but which previously could not be homologised with roots in any other phyla (Cavalier-Smith and Karpov’s 2012 Table 2). Vpr2 is not actually a centriolar root but a BB, lying beside C2 and running backward parallel to the right side of vp1, previously identified as R2.

But why did the chromist BB evolve and how can we reconcile any explanation with the inference that colponemids retain the ancestral excavate posterior ciliary vane and feeding mode? The key point is that in *Tsukubamonas* and excavates sensu stricto [i.e. those also with singlet roots: Loukozoa (*Malawimonas*; Metamonada) and Jakobea], the kinetid was ancestrally at the very apex of the cell; except for a dorsal mt fan in some lineages, and a short anterior root anchoring the relatively short anterior cilium, the mt and fibrous cytoskeleton is almost entirely postciliary and groove-associated. In many lineages in all four chromist groups this is not so and there is an extensive preciliary cytoskeleton; their kinetid is lateral not apical, in marked contrast to excavates.

I think the origin of BB was causally associated with that of cortical alveoli. In both chromists and glaucophytes, cortical mts are specifically associated with the inner cytoplasmic face of alveolar membranes, not with the plasma membrane as in excavates. Interestingly, alveoli of some Glaucophyta have more varied arrangement than previously realised, being imbricate in some *Glaucocystis* species but tiled in others as in other glaucophytes and chromists (Takahashi et al. 2016a, 2016b). Cortical alveolar origin was probably linked to mt repositioning over the whole cell surface. Cortical mts of excavates, essentially the dorsal fan, necessarily had to be detached from the plasma membrane in order to be reattached to cortical alveoli when alveoli originated. Thus, detachment of excavate dorsal fan mts from the plasma membrane to generate subpellicular mts occurred at the same time as the fan’s detachment from centriole C2: it appears that all linkages between mt fan, membrane, and C2 radically changed in a concerted organisational upheaval that simultaneously made BB (from the more ribbon-like part of the fan) and the non-ribbon-array single subalveolar mts (from the more divergent mt part of the fan). Thus, corticate origin involved not just new alveolar membranes (with at least novel Rab 11B noted above, but I suggest also novel SNAREs for vesicle targeting to them to allow cortical alveolar growth and division) but also associated changes in position and arrangement of mt-nucleating proteins. This major change in cell organisation (greater than the origin of fungal cell walls) is recognised by superkingdom rank for Corticata.

Previously I argued that cortical alveoli arose as cortical rigidifying cytoskeletal elements additional to loukozoan mt roots (Cavalier-Smith 2013b). Earlier still I argued that a key selective advantage of alveoli was ‘allowing larger and more complexly structured cells’ and that they first evolved in protists that were ‘pseudophytoplankton’, i.e. biciliates in the oceanic photic zone that harboured ‘endosymbiotic cyanobacteria instead of true plastids’, and which included the protists that first evolved chloroplasts and became Plantae (Cavalier-Smith 1991). Consequential large cell size enabled cells to contain more and more cyanobacteria and simultaneously eat larger eukaryotic prey, and to help catch them (and defend against other predators); it was accompanied by deployment of extrusomes anteriorly close to the anterior ciliary pocket and head of the ventral groove. Cortical alveoli had to be excluded from pocket/groove regions to allow continued digestion, thus concentrated dorsolaterally; preciliary expansion of the cell could increase its volume without compromising ventral feeding, which compared with loukozoans moved centrioles backward from the cell apex (as in all Miozoa including colponemids). Thus, physical destabilisation of previous anterodorsal root attachments to the PM by the novel interposition of cortical alveoli, coupled with a new selective advantage for greater preciliary cell volume with adequate internal cytoskeletal support, favoured cells that split the dorsal fan core away from C2 and moved its nucleation point more anteriorly to make BB.

On this scenario, Plantae having evolved chloroplasts largely abandoned phagotrophy in favour of autotrophy and thus lost the band-like part of the dorsal fan, having no selective advantage for retaining it as a BB supporting preciliary ingestive structures, whereas their sister chromist precursors kept BB, phagotrophy and extrusomes, and were able to enslave a red alga very early in plant diversification and become chromists. Protalveolates lost photosynthesis and BB and retained the general loukozoan feeding mode despite addition of alveoli that allowed them to focus on eukaryotic prey, not small bacteria like *Malawimonas* and Jakobea. When ciliates evolved kineties and apical mouth BB became unnecessary and was lost. Most other chromists kept BB.

This (or any other) mode of origin of BB entailed changes to ill-understood fibrillar proteins involved in attachment and nucleation of ciliary roots. A century or more of molecular cell biology elucidating their functions and comparative biology may be needed before we shall know whether or not this explanation is correct and if not replace it by a better one. A key group to study thus will be Myzodinea, specially important for understanding the origin of dinozoan BB variants (Okamoto and Keeling 2014b) and the apicomplexan conoid, pseudoconoid, and paraconoid.

## Chromist ciliary hair evolution

Just as Myzozoa evolved apical ingestion instead of posterior groove-based ingestion and ciliates evolved an anterior multikinetid cytostome, a third ancient halvarian innovation causing anterior ingestion was thrust-reversing tripartite tubular hairs (retronemes) that form one or more often two rows on the anterior cilium only of almost all ciliated heterokonts (Cavalier-Smith 1986). Typically the anterior cilium beats symmetrically with waves progressing from base to tip. If hairs were absent, such motion would propel the cell with this cilium pointing backward; but heterokont hairs are sufficiently long and rigid to act like oars of a Roman galley to pull the anterior-pointing cilium and the cell forward (Holwill and Sleigh 1967). That creates a very strong anterior water current towards the cell body bringing bacteria and other small prey to the ciliary base where phagocytosis engulfs them. This swimming novelty radically changed the feeding mode of the ancestral heterokont by moving the cell’s ingestion site anteriorly in an analogous way to the origin of myzocytosis in Myzozoa. Some general consequences of this are discussed in more detail in supplementary SD8; the consequential evolution and diversification of an anterior cytopharynx in the early-branching heterokont phylum Bigyra is discussed in SD 9, and comparative evolution of BB in SD10.

Non-tubular anterior ciliary hairs in myzozoan alveolates and a few Rhizaria (the cercozoan *Aurigamonas* and one foraminiferan gamete) and more distantly in the endohelean hacrobian *Heliomorpha* (and early diverging plant *Cyanophora*) suggests that simple ciliary hairs, like cortical alveoli were ancestral characters for Corticata (Chromista plus Plantae). Thus, hairs attached to the anterior cilium in two rows and developmental restriction to that cilium only (necessarily caused by continued presence of the posterior ciliary vane—whose inner skeleton might use similar attachment sites) were already in place before tripartite retronemes evolved. As soon as retronemes became rigid and long enough to reverse thrust they provided an extremely efficient feeding current to the anterior ciliary base.

Though the molecular nature of some retroneme proteins is being elucidated (Honda et al. 2007) nothing is known of the proteins of harosan simple hairs or the vane skeleton. When it is we may be able to work out whether retronemes evolved from simple hairs, from the vane skeleton or from another cell component. Retroneme proteins have similar cysteine-rich EGF-like domains to the tenascin family of extracellular matrix glycoproteins of animals (Armbrust 1999; Honda et al. 2007), so might have evolved from extracellular glycoproteins widely present on the plasma membrane not from a preexisting hair. Cryptophytes have both tubular and non-tubular hairs, so both can coexist in one cell. When first discussing retroneme origin I argued that they are probably homologous with cryptist tubular hairs (now known in *Lateronema* and *Palpitomonas* as well as Cryptomonada) (Cavalier-Smith 1986). But now that Alveolata, Rhizaria, and Heliozoa have been added to Chromista (Cavalier-Smith 2010), Heterokonta and Cryptista, though both chromists, are evidently less closely related than once thought, making it possible that their tubular hairs evolved convergently, as Moestrup (1982) had supposed; possibly both kinds of tubular hair arose from an homologous chromistan simple hair precursor. If so it probably happened in heterokonts before their ancestor lost the posterior ciliary vane, which must have been lost several times independently within Harosa, but in Cryptista only after the ancestor of Hacrobia independently lost the vane. That would explain why tubular hairs are confined to the anterior cilium in Heterokonta but are on both cilia in Cryptista (often structurally different on each); however, their presence and differentiation on cryptophyte C1 could have been secondary even if they evolved in the ancestral chromist on C2 only as I originally argued—still plausible, but needing biochemical testing. As far as is known, cryptist tubular hairs do not reverse thrust.

As retronemes evolved very early in chromist evolution, Bigyra were able to lose plastids and become the dominant protist suspension feeders in the oceans, whose diversity is only now becoming recognised with the discovery of MH/MAST clades and culturing new zooflagellates like *Incisomonas*. From the same harosan stem, Rhizaria similarly lost plastids marginally earlier through invention of filopodia and reticulopodia enabling them to become dominant benthic feeders, spawning reticulose Retaria, and a host of filose (sometimes reticulose) and/or gliding Cercozoa. Those novel benthic feeding modes replaced excavate groove-feeding, causing loss of the ciliary vane. Likewise in alveolates the origin of ciliate kineties and mouth enabled them to specialise as much larger suspension feeders (and secondarily evolve raptoriality as in Bigyra), and origin of myzocytosis in Myzozoa gave a novel feeding mode to both groups, causing separate losses of the ciliary vane. These new feeding modes (and others in Hacrobia, e.g. haptonema of haptophytes, axopodia of Heliozoa; and abandonment of phagocytosis for autotrophy by Plantae) and new swimming modes associated with the origin of cryptist tubular hairs explain why protalveolates alone amongst corticate eukaryotes, all of which had a common *Malawimonas*-like excavate ancestor, retained the posterior ciliary vane—for over 700 million years of stringent stabilising selection—because they alone retained loukozoan-like groove-based feeding.

## Rhizarian evolution: filose and reticulose pseudopodial body plans and ciliary gliding

In contrast to Halvaria which were probably ancestrally planktonic photophagotrophs leaving both algal and predatory descendants, Rhizaria ancestors became benthic phagotrophs by evolving filose (threadlike) pseudopodia for feeding on surfaces, thus losing plastids altogether. This novel soft amoeboid surface so radically transformed their cytoskeleton that it has been difficult to homologise it with Halvaria and Hacrobia (Cavalier-Smith and Karpov 2012). To better represent the primary dichotomy between filose and reticulose body plans seen on the latest 187-protein trees (Cavalier-Smith et al. 2015a), Table 1 transfers Endomyxa (which include both reticulose and mixed reticulose/filose groups) from Cercozoa to Retaria and makes new subphylum Ectoreta for classical Retaria (Foraminifera, Radiozoa). That allows the simple generalisation that thus revised Cercozoa were ancestrally relatively small gliding flagellates typically using filose pseudopodia for feeding and never have cortical alveoli, whereas sister phylum Retaria were ancestrally large vegetatively reticulose amoeboid forms without cilia whose biciliate spores or sperm (lost by some lineages) never glide.

Some Cercozoa secondarily lost cilia to generate filose amoebae polyphyletically and a very small minority became secondarily reticulose through evolving filopodial fusion; just as a few endomyxan Retaria became secondarily filose. Ectoretan trophic cells are often subdivided internally into reticulose ectoplasm and organelle-containing endoplasm by a central capsule composed of a hollow sphere of membranous alveoli with dense contents. The capsule wall has pore-like gaps between the alveoli through which the mt skeleton penetrates from the inner endoplasmic region containing organelles such as nucleus, mitochondria and Golgi to the outer ectoplasmic region comprising the pseudopodial network specialising in phagocytosis and digestion. I suggest that central capsule alveoli are relics of the ancestral corticate cortical alveoli and the pseudopodial network grew out though gaps between them to fully invest the cell with an ectoplasmic net. By contrast Endomyxa and Cercozoa appear to have lost cortical alveoli independently, which would have allowed relatively small cells compared with Ectoreta to have fed pseudopodially over their whole surface.

I suggest that Radiozoa and cercozoan Phaeodaria when losing cilia and their roots from trophic phases multiplied and modified BB for evolving radial axopodia and that cercozoan desmothoracids and *Tetradimorpha* which evolved axopodia without losing cilia may also have multiplied BB for this job. Likewise foraminiferal reticulopodia and granofilosean filopodia [supported by mts unlike filopodia in general: *Limnofila* (as ‘*Gymnophrys*’; Mikrjukov and Mylnikov 1998)] may independently have adopted the characteristically MAP-reinforced BB mts for internal support in their non-ciliate trophic stages.

The cytoskeleton of ectoretan ciliated sperm (rarely seen in free-living forms) has been scarcely studied. In Endomyxa only parasitic Phytomyxea have biciliate zoospores; the other three classes lost them; centriolar roots are cruciate, two each; they almost certainly became cruciate as discussed above for green algae and heterokonts by heterochrony producing an R4 but accelerated development of R2 and as non-phagotrophs must be radically simplified from the ancestral rhizarian condition.

Cercozoan roots are more extensively studied (reviewed in Cavalier-Smith and Karpov 2012). Some lineages (notably Cercomonadidae) are secondarily more complex than the generality whereas others have undergone secondary simplification (e.g. Helkesida, a new order established here to embrace *Sainouron*, *Helkesimastix*, and *Cholamonas* with simplified roots and guttulinopsids with no cilia, but all related; Bass et al. 2016). One can be confident that ancestrally posterior R1 and R2 and anterior R3 were present as most Cercozoa as in other chromists, but identifying other roots has been problematic.

Previously the identity of ventral posterior root vpr2 (first named in cercomonads; Karpov et al. 2006) and its presumed homologues in other Cercozoa was a puzzle, as though predominantly posterior (and in cercomonads parallel to vpr1 (i.e. R2) it was not nucleated by either C1 or C2 but passed a short way anteriorly of both centrioles (Cavalier-Smith and Karpov 2012). Given the new chromist perspective on BB (Fig. 6), I now identify vpr2 as the cercozoan BB homologue as it is positionally equivalent to halvarian and hacrobian BB to the cell’s right of the kinetid.

Likewise left root lr, passing anteriorly to the left and nucleated between the centrioles, has been problematic. When first drafting Cavalier-Smith and Karpov (2012), I considered lr a homologue of the excavate singlet and supposed that of our Table 2 species only *Katabia* had evolved an R4 (i.e. ur) and that Cercozoa primitively only had anterior R3 like *Malawimonas*; however, Karpov and I were unable to agree a joint interpretation of lr and settled on a conservative compromise assuming that all these genera had an R4 (mostly lr) like advanced heterokonts. I consider that unlikely given the evidence discussed above that Halvaria had only R3 but possessed an ancestral posterior singlet, and argue that cercozoan lr probably represents the excavate singlet reoriented forward when pseudopodial feeding replaced ventral groove feeding to give extra dorsal support to the cell anterior. In most Cercozoa lr has just 1 mt (and thin associated dense fibre) like the excavate singlet, but in a few it has two, which could be secondary doubling, and simply fits this new interpretation. Possibly the double nature of R2 (vpr1) of *Thaumatomonas* represents ancestral split R2 but in most cercozoa a split nature of R2 is not identified, possibly simply because serial sections were not studied sufficiently posteriorly as there was then no obvious reason for doing so.

*Bigelowiella* is especially interesting as its anterior r2 identified by Cavalier-Smith and Karpov (2012) as R4 (2 + 1 mts) and posterior r4 (2 + 3 mt) identified by us as R2 are highly reminiscent of the 3 over 1 configuration of R4/R2 roots in cruciate-root green algae discussed above. I postulate that *Bigelowiella* r2/r4 both evolved from the ancestral R2/singlet complex and both components were conserved in this highly simplified non-phagotrophic algal flagellate when accelerated development of R2/S made a second anterior root (R4/S) and cruciate pattern for exactly the same reasons as in chlorophytes. For better understanding cercozoan root evolution we need more-posterior sections and also tomography and decoration studies to check mt polarity, which I suspect may be opposite for BB (vpr2) than for the three major centriolar roots (as is true for trypanosomatid pellicular mts, and Cavalier-Smith and Scoble (2013) suggested for the BB component of raphidophyte rhizostyles).

I thus now regard the ancestral condition for Cercozoa as anterior pointing R3 + reoriented S, and posterior left R1 and right R2. This needs testing by studying roots in the earliest diverging skiomonads that glide on both anterior and posterior cilium, unlike all others that glide only on the posterior cilium. A tiny minority of Cercozoa polyphyletically abandoned gliding and become planktonic, notably *Katabia*, *Bigelowiella*, *Minorisa*, *Mataza*, *Ebria*, and *Cryothecomonas*. Gliding meant that the preciliary cell anterior typically encountered prey first, so needed a more prominent mt cytoskeleton than in excavates; previously we similarly explained the more complex anterior mts in some cercomonads compared with others (Bass et al. 2009). Whether gliding flagellates, filose amoebae or reticulose amoeboids or axopodial feeders, the ancestral corticate separation of BB from the kinetid preadapted Rhizaria for multifarious feeding modes, as arose in contrasting ways in Halvaria, but early evolution of filose/reticulose pseudopodia radically changed rhizarian coadapted life styles and body plans.

One secondarily uniciliate *Minorisa*-like lineage (del Campo et al. 2013) secondarily enslaved an ulvophyte green alga related to *Bryopsis* (Suzuki et al. 2016) and evolved novel protein import machinery analogously to chromists to make chlorarachnid algae, here ranked as a sister order to new order Minorisida (Tables 1, S1). Even though chlorarachnids retain the green algal nucleus as a nucleomorph (as cryptophytes kept the red algal nucleus) and its PM as a PPM their new import machinery did not recruit a derlin (Hirakawa et al. 2012) in marked contrast to the first chromist. Separate secondary symbiogenetic enslavements clearly evolve in different ways, as also shown by different consequences of green algal replacement of dinoflagellate chloroplasts in *Lepidodinium* (Matsumoto et al. 2011a, b, 2012) that yielded convergently similar membrane topology to chlorarachnids (Watanabe et al. 1987) and by euglenoid enslavement of a green alga that yielded only three integrated chloroplast envelope membranes. Even though secondary symbiogenesis invariably involves an origin of bipartite targeting sequences, the import mechanism was unique in all four known cases (Cavalier-Smith 2013a), reemphasising the unity of chromists that evolved by a single secondary symbiogenesis of a red alga ancestrally evolving derlin-based import machinery.

No Rhizaria have tubular hairs; very few have simple hairs: in Cercozoa (e.g. *Metromonas*) posterior and in Foraminifera (e.g. *Boderia*) anterior. The other three chromist lineages often have simple ciliary hairs and in heterokonts and cryptist Hacrobia tripartite tubular ciliary hairs (Cavalier-Smith et al. 2015a). Unless heterokont and cryptist tubular tripartite hairs are convergent the ancestral rhizarian lost them.

## Chromist ciliary transition zone (tz) evolution

Ciliary axonemes with nine outer doublet and a central pair (CP) of singlet mts and the nine-triplet structure of centrioles with central basal ninefold cartwheel have a highly conserved standard structure across all eukaryote kingdoms. In marked contrast, the intervening ciliary compartment, the transition zone (tz), differs remarkably amongst major eukaryote groups but is often strongly conserved within each. Conservatism of these major differences has been very useful to evolutionists and taxonomists by providing differentiating characters, like the 9-fold star and dense cylinder that help define Viridiplantae. Their functional significance is poorly understood, but might in part depend on whether or not CP rotates relative to the doublets (Mitchell 2007; Cavalier-Smith and Oates 2012), tz requirements for rigid anchoring or constraining rotation to avoid damage being obviously different. Tz length may depend on whether cilia project from a cell apex as in apicomonads, which always have short simple tzs, or are deeply embedded in a cavity within which undulation (thus a 9 + 2 structure) is undesirable as in euglenoids that have some of the longest tzs or within long grooves as in dinoflagellates. The very simple condition in the core excavate *Malawimonas* of a very short tz with little obvious substructure except a small axosome at the base of CP (O’Kelly 1999) may represent the ancestral excavate state prior to chromist origin. Chromist tzs diversified more radically than in other kingdoms.

Many chromists, notably most alveolates, retain short tz and axosomes: single in ciliates, double in Miozoa—an early divergence; but some, notably heterokonts and rhizaria, evolved their own more complex, seemingly unique structures, as supplementary SD11 (specifically on heterokont helices and rings) and SD12 (more general) discuss in detail. Rhizaria have short to medium length tzs with variable dense structures but share characteristic proximal hub-lattice structures at the centriole/tz interface and hub-spoke structures at the tz distal end (Cavalier-Smith et al. 2008, 2009). Though similar structures are apparently absent in other protists, I suggest that the proximal and distal tz boundaries may be defined by proteins conserved more widely, e.g. across all chromists, all corticates or even all eukaryotes, such as SAS-6 paralogues discussed in SD12. Eventually universal tz delimiting principles may be established by comparative molecular cell biology. A second point emphasised is that the distinctively heterokont so-called transition helix (TH) present as a sleeve around the CP complex base probably arose at the same time as retronemes, so its function may be related to mechanical consequences of their origin, but TH is not actually a tz structure as it is at the base of the ciliary shaft proper. SD11 also stresses (1) that this TH sleeve, which might function analogously to the upper basal cylinder of Viridiplantae, is probably not homologous to tz rings (truly tz structures) that help define ochrophyte subclass Hypogyrista, which should no longer be called a TH; and (2) that the classic distinction between a ‘single’ chrysist ochrophyte TH and double pseudofungal/bigyran TH is probably invalid, one of several reasons why I here reduce Gyrista in rank to phylum.

## Major conclusions

I have sought to show that chromists cannot be understood just as algae or just as heterotrophs; only when perceived ancestrally as elaborate photophagotrophs, whose ancestor was a neokaryote excavate protozoan that evolved cortical alveoli and shifted ingestion anteriorly, can one understand their unique cytoskeleton and chloroplast-associated membrane topology in all their complexity and immense diversity. The present synthesis of chromist cell evolution has greater depth and solidity than was possible previously (Cavalier-Smith 2004a) through integrating major advances since then in four key areas: (1) greatly improved understanding of molecular cell biology, especially protein targeting into the PS; (2) more robust eukaryote and chromist sequence phylogenies using scores of genes; (3) more extensive ultrastructural characterisation of excavate and chromist centriolar roots and cilia; (4) discovery of numerous new chromists studied ultrastructurally and by sequencing, especially chromeroids and in Cercozoa and Hacrobia. Yet much chromist cell biology remains largely terra incognita; opportunities for exciting discoveries are legion. The most important novel conclusions are:

1. Chromista are monophyletic and comprise four major clades of distinctive body plans, feeding modes, motility behaviour, and lifestyles: Heterokonta, Alveolata, Rhizaria, Hacrobia. Each split early into two phyla and subphyla with unique cell structures.
2. Despite their remarkable diversity, chromists are unified by a shared common ancestral body plan with (1) a skeleton comprising cortical alveoli with subpellicular microtubules (mt) and a mt bypassing band (BB) distinct from the three major mt centriolar roots inherited from excavate protozoa, and (2) chloroplasts of red algal origin inside the endomembrane system with unique membrane topology and derlin-based periplastid protein import machinery.
3. Multiprotein sequence trees robustly group Chromista and Plantae as the corticate clade. The best ones show both kingdoms as sister clades, and the holophyly of both chromist subkingdoms (Harosa, Hacrobia), although all deep-branching corticate lineages diverged so rapidly that establishing basal relationships has been challenging. Within Chromista all phyla as here revised (only eight needed) are clades, as is Halvaria (Heterokonta, Alveolata), and Rhizaria (Cercozoa, Retaria) sister to Halvaria; within Plantae, Rhodophyta and Viridiplantae are probably sisters and Glaucophyta the deepest branch.
4. Corticates evolved from a neokaryote excavate ancestor by evolving Golgi-derived cortical alveoli with subalveolar mts to make large biciliate planktonic cells. Alveolar origin separated the excavate dorsal mt fan/ribbon from the cell surface and anterior centriole, part of which probably became subalveolar mts (retained by Plantae) and part the unique chromist BB (absent in Plantae).
5. Chromists evolved from the corticate ancestor by (1) evolving BB to support the precentriolar cell anterior as it became extended compared with excavate ancestors, and (2) enslaving a red alga placing it inside the endomembrane system and evolving novel derlin/Cdc48 protein import machinery for protein transport across the periplastid membrane (PPM; former red algal plasma membrane) that was lost in ancestral Dinozoa, which therefore have three membranes separating chloroplast stroma from cytosol, not four as in other chromists or two as in Plantae.
6. Algal chromists other than Dinozoa have a periplastid reticulum in the periplastid space (PS, former red algal cytosol), which is probably a relict red algal endosomal or *trans*-Golgi network compartment that grows by vesicle budding from the PPM and is arguably the site of the derlin-derived protein translocon that evolved from the red algal ER/endosomal protein extrusion machinery and mediates protein import into the PS using a Cdc48 motor for ubiquitinated proteins.
7. The ancestral chromist was a planktonic biciliate photophagotrophic chromophyte with chlorophyll *c*2, cortical alveoli, subpellicular mts, BB, three centriolar roots (R3 anterior dorsal, R1 left posterior, R2 posterior split right root with attached singlet mt) and probably tubular ciliary hairs, which became variously modified in the four main chromist groups. This chromophyte plastid was retained by vertical descent by four phyla (Haptista and Cryptista in Hacrobia; Miozoa in Alveolata, Gyrista in Heterokonta) but several plastid losses (mainly in early branches) generated purely heterotrophic descendants.
8. The ERAD protein translocon derlin underwent gene duplication in the ancestral eukaryote, both paralogues A and B (Der1 and Dfm1 in *S. cerevisiae*) being retained with partially different cofactors in most eukaryotes (B/Dfm1 lost only by fornicate metamonads). Cryptophytes kept the nucleomorph-coded red algal A/Der1 orthologue in the periplastid compartment, but Halvaria and haptophytes lost it (and its associated ubiquitin ligase) and instead retargeted red algal derlin B and a different ubiquitin ligase before independently losing nucleomorphs. Therefore these three algal lineages must have diverged at almost the same time; plastids using derlin for import cannot have been transferred from cryptophytes to other chromist lineages long after the unique secondary red algal enslavement as cryptophytes would have lost redundant red algal derlin B relatively quickly. Vertebrates duplicated derlin B to evolve tissue-specific derlin-3.
9. New dinoflagellate subclass Karlodinia got its chloroplasts from haptophytes by tertiary symbiogenesis, but converted them to unique chimaeras with dinoflagellate and haptophyte plastid proteins, retaining haptophyte periplastid-like derlins and cdc48s, yet paradoxically seemingly are bounded by only a two-membrane envelope as in Plantae. As this is the only known case of tertiary symbiogenesis in the history of life, it shows (contrary to frequent assumptions) that tertiary symbiogenesis is not a credible way of laterally transferring chromist chloroplasts and complete 5-membrane topology from one phylum to another so as to mimic the unique red algal secondary symbiogenesis. No examples exist of tertiary symbiogenetic plastid transfer to a heterotrophic host, so the karlodinian plastid does not support the idea that the chromist last common ancestor was heterotrophic and Myzozoa, ochrophytes, and haptophytes acquired plastids by tertiary transfers from cryptophytes.
10. Rhizaria ancestrally lost the plastid by becoming benthic heterotrophs feeding by filose pseudopodia, as did ciliates when evolving giant planktonic heterokaryotic cells with rows of cilia and anterior mouth with multiciliate mouthparts. Some ciliates cultivate green algae internally as symbionts providing photosynthate, as do many Rhizaria; but only one rhizarian order (Chlorarachnida; sister to new heterotrophic order Minorisida) permanently enslaved a green alga to gain a permanent chloroplast by evolving novel protein import machinery different from chromophytes (no derlin).
11. Heterokonts evolved thrust-reversing tripartite anterior ciliary hairs generating novel water currents that brought prey for ingestion at the anterior ciliary base, and split early into heterotrophic Bigyra that fully exploited that feeding mode and Gyrista that mainly focused on photosynthesis (Ochrophytina, e.g. diatoms, brown algae) or heterotrophic osmotrophy (Pseudofungi).
12. I explain how differences in BB mt structures in alveolate Myzozoa evolved, including how pseudoconoids of free-living myzocytotic apicomonad Apicomplexa in association with two centriolar roots evolved into the invasive conoids of parasitic Sporozoa (e.g. malaria parasite, *Toxoplasma*) and were simplified to *Colpodella* paraconoids; I radically reappraise myzocytotic flagellate evolution, correcting many errors.
13. The outermost of the two nested mt arcs that jointly are an ancestral character for Hacrobia is homologous with BB of Harosa. The uniquely chromist BB, distinct from centriolar roots, provided the ancestor of mt axonemes of axopodia, thus enabling heliozoan-like protists to evolve in each of Hacrobia, heterokonts, and Rhizaria, but never once in any non-chromist.
14. I revaluate chromist ciliary transition zone (tz) evolution, arguing that the heterokont transition helix is always fundamentally double, not homologous with hypogyrist transition rings, and core distal tz elements of Rhizaria may be ancestral eukaryotic features.
15. I tabulate a revised reference classification of chromists that makes all phyla holophyletic (by transferring Endomyxa from Cercozoa to Retaria and treating Pseudofungi and ochrophytes as subphyla of Gyrista); several major improvements better reconcile cell biology, ultrastructure, and sequence phylogeny, especially in apicomonads and primitive dinoflagellates that are cytologically more diverse than previously appreciated.

## Electronic supplementary material

ESM 1 (PDF 1.03 mb)

ESM 2 (GB 873 kb)

ESM 3 (EZDRAW 2631 kb)

ESM 4 (EZDRAW 154 kb)

ESM 5 (EZDRAW 258 kb)

ESM 6 (EZDRAW 112 kb)

ESM 7 (EZDRAW 146 kb)

ESM 8 (EZDRAW 176 kb)
