# Supplementary material for: Kingdom Chromista and its eight phyla: a new synthesis emphasising periplastid protein targeting, cytoskeletal and periplastid evolution, and ancient divergences
Source: Protoplasma. 2017 Sep 5;255(1):297–357. doi: 10.1007/s00709-017-1147-3 (PMC5756292; doi:10.1007/s00709-017-1147-3)
Supplement: Supplementary file 1 — (PDF 1.03 mb) [file 709_2017_1147_MOESM1_ESM.pdf]

Electronic Supplementary material for **Kingdom Chromista and its eight phyla: a new synthesis emphasising periplastid protein-targeting, cytoskeletal and periplastid evolution, and ancient divergences** by Thomas Cavalier-Smith

Contents:

Supplementary figures

Fig. S1 Derlin site-heterogeneous PhyloBayes v. 3.2 CAT tree for 153 neokaryote derlins (no Eozoa) pp. 1-2
Fig. S2 Derlin RAxML GTRGAMMA tree for 153 neokaryote sequences (excluding Eozoa) p. 3
followed by discussion comparing it with Fig. S1 pp. 3-4
Fig. S3 Derlin PhyloBayes CAT-GTR tree for 193 eukaryote sequences including Eozoa. p. 5
Fig. S4 Derlin RAxML tree for 193 eukaryote sequences including Eozoa p. 6
Fig. S5 Derlin PhyloBayes CAT-GTR for 122 neokaryote sequences (excluding Eozoa) p. 7
Fig. S6 Derlin RAxML GTRGAMMA tree for 122 neokaryote sequences (excluding Eozoa) p. 8
Fig. S7 Derlin PhyloBayes CAT-GTR tree for 203 eukaryote sequences (including Eozoa and metamonads) p. 9
Fig. S8 Derlin RAxML GTRGAMMA tree 203 eukaryote sequences (including Eozoa and metamonads) p. 10

Supplementary Table

Table S1 Classification of kingdom Chromista Cavalier-Smith, 1981 and its 8 phyla and 82 classes. pp. 11-18

Supplementary Discussion (SD)

SD1. Chromist algal pigment diversification pp. 18-19
SD2. Plant cytoskeletons differ substantially from chromists pp. 19-21
SD3. Ciliary and cytoskeletal diversification in myzozoan alveolates pp. 21-22
SD4. Apicomonad diversity and evolution: clarifying confusions pp. 22-25
SD5. Pseudoconoid and conoid evolution: cytoskeletal adaptation in the origin of Sporozoa pp. 25-27
SD6. Dinoflagellate cytoskeletal, plastid, and nuclear diversification pp. 27-28
SD7. Ciliate kinetids also reflect an excavate origin pp. 28-30
SD 8. Heterokonts also shifted ingestion anteriorly pp. 30-32
SD9. Heterokont cytoskeletal evolution: the bigyran cytopharynx pp. 32-34
SD10. The bypassing microtubular band in heterokonts pp. 34-35
SD11. Evolution of heterokont ciliary transitional helix (TH) and tz rings pp. 36-38
SD12. Chromist ciliary transition zone (tz) evolution pp. 38-40

Supplementary References pp. 40--44

Includes all references cited in Supplementary Discussion SD1-12 that were not cited in the main reference list

Derlin257.ali.zip An alignment for all 259 analysed derlin sequences in GenBank format. This includes all the sequences used for the 8 trees shown plus 55 extra animal sequences used for an 82-sequence animal tree (not shown) to establish when in animal evolution the gene duplication producing the tissue-specific derlin-3 paralogue evolved - at least as early as the ancestral gnathostome, being present in cartilaginous and bony fish; I found no agnathan (lamprey or hagfish) derlins so cannot exclude the possibility that the duplication took place in the vertebrate rather than gnathostome common ancestor. As it was absent in all invertebrates sampled including protochordates, derlin-3 is vertebrate-specific. Derlin B sequences (upper) are separated from the derlin A sequences (lower) by a mask in which 1 indicates amino acid positions included in the analyses and 0 those excluded.

****

Fig. S1. Site-heterogeneous PhyloBayes v. 3.2 CAT tree for 153 neokaryote derlins using 201 well-aligned amino-acid positions. The two chains run converged well (maxdiff. 0.0926573; burnin cutoff 581; 177,170 trees summed). The tree is rooted between ancestral paralogues A and B, periplastid derlins highlighted in yellow; periplastid derlin Bs have bipartite N-terminal targeting peptides (their seeming absence in *Chrysochromulina* only is likely artefactual or misannotation). It suggests that the ancestral chromist retained both host and red algal derlins A and B, the red algal derlin A kept by cryptophyte nucleomorphs being made on periplastid ribosomes but lost by Halvaria and haptophytes, which instead kept red algal derlin B and retargeted it to the periplastid compartment (and evolving two B periplastid paralogues, perhaps in the ancestor of haptophytes and heterokonts) before independently losing the red algal nucleus. As it is unlikely that both red algal derlins were kept for many millions of years after red algal enslavement, it is highly probable that halvarian,

haptophyte, and cryptophyte algae diverged almost simultaneously with the separation of red and algae as this and Figs S2-8 collectively show; so there can have been no successful tertiary derlin transfers long after initial red-algal enslavement. Support values for bipartitions are posterior probabilities. The ML tree for the same data (RAxML LGF: Fig. S2) also showed the derlin A and B bipartition (extremely weak bootstrap support: 29%; 52% on Fig. S6 for 122 sequences) and the same major clades but with some statistically insignificant differences in topology (e.g. the long-branch halvarian periplastid derlin B paralogue moved two nodes upwards to be sister to one haptophyte periplastid derlin B paralogue; the other haptophyte/heterokont periplastid derlin B paralogue was weakly within red algae, so all periplast Bs group with red algae only - the *Volvox*/*Chlamydomonas* clade moved: green arrow, making green plants also a clade). Nuclear-coded periplastid derlin Bs were one clade on trees with only 122 sequences (Fig. S5, S6), sister only to the red algal clade by CAT (Fig. S5). The alignment (supplementary material **Derlin257.ali**) includes the most relevant sequences from Petersen et al. (2014) and Kalanon et al. (2009; Der1-n labels from that paper) plus several for Eozoa (only on Figs S3, S4, S7, S8 for 193/203 sequences) and other neokaryote sequences from GenBank chosen (by BLAST and word search) to break up long branches and better represent all major taxa. Black arrows highlight *Saccharomyces cerevisiae* paralogue Dfm1 and where Fig. S3 places its Der1.

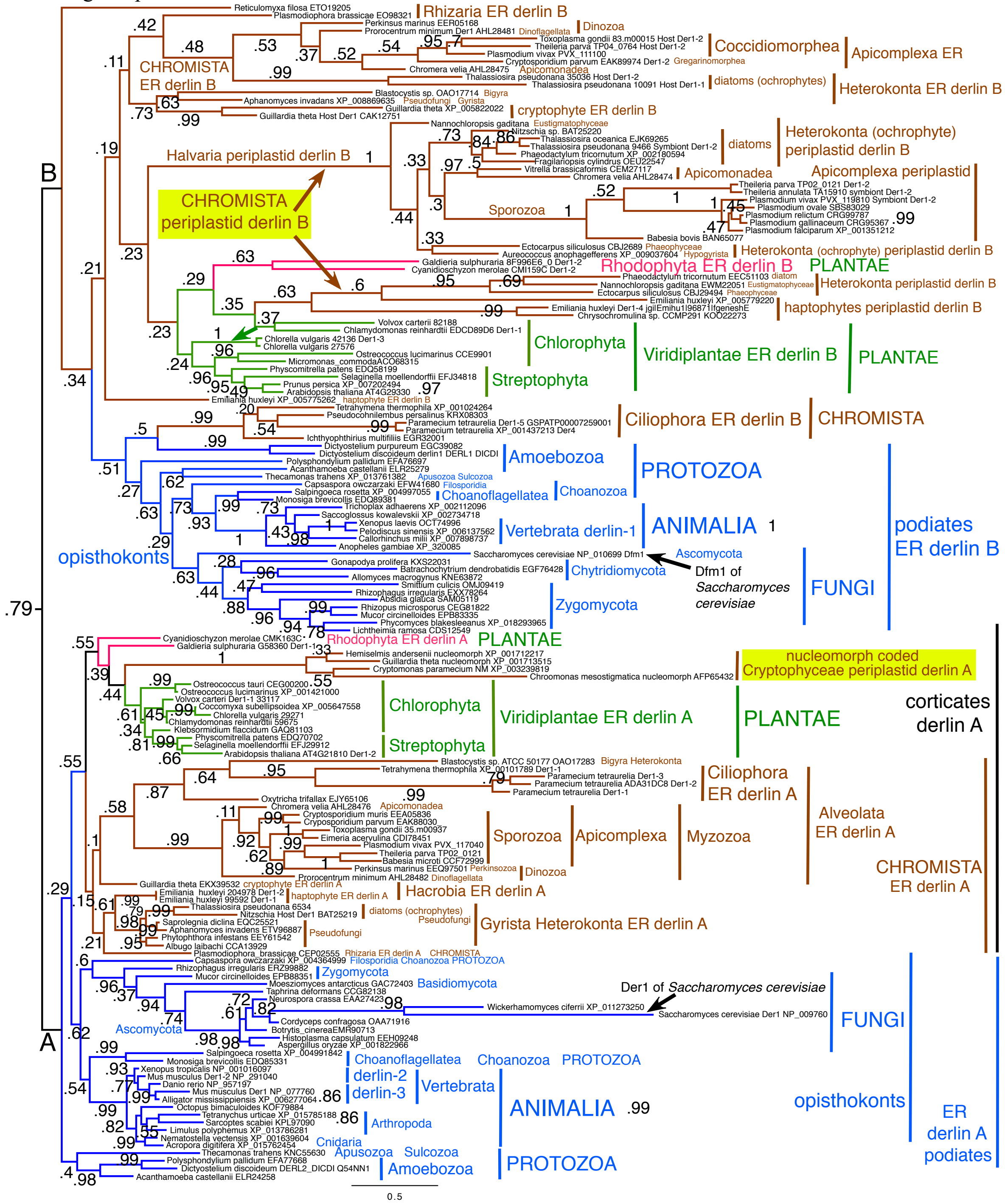

Fig. S1. Site-heterogeneous PhyloBayes v. 3.2 CAT tree for 153 neokaryote derlins using 201 well-aligned amino-acid positions.

Fig. S2. Derlin RAxML GTRGAMMAF tree for 153 neokaryote sequences (excluding Eozoa). Fast bootstrap analysis (1000 pseudreplicates) for the same sequences and alignment used for the **Fig. S1** PhyloBayes CAT-GTR tree.

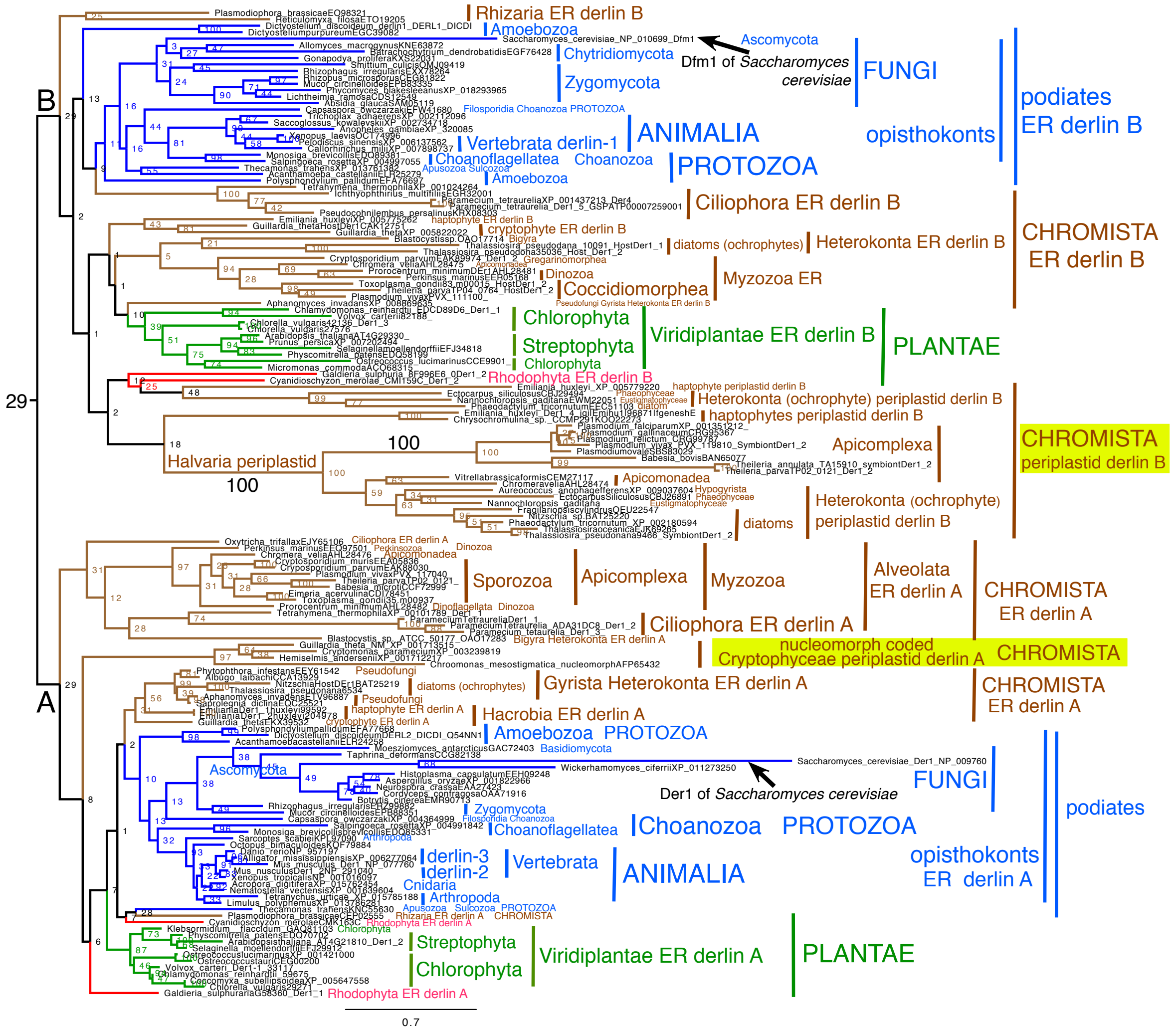

Comparison of Fig S2 (ML) with Fig. S1 (CAT for the same sequences). Both show the bipartition between paralogues B and A (0.79 PP, 29% BS support). CAT wrongly roots B within chromists and A within podiates; ML consistently wrongly roots both B and A within chromists. Neither these nor any other inconsistent features between these trees have statistically significant support. Both wrongly put ciliate derlin B within podiates rather than with other alveolates, obviously because of its long *Paramecium* branch and hugely stretched basal stem, plus the fact that long-branch *Dictyostelium* wrongly branches even lower and does not group with other shorter-branch Amoebozoa. Podiate derlin B is a clade when *Dictyostelium* is omitted on CAT trees (Fig. S5 for 122 sequences) but Amoebozoa remain wrongly paraphyletic and ciliates are wrongly their sisters (because the Fig. S5 B subtree is misrooted within chromists as on Figs S1, S2). This shows that derlin evolves at sufficiently varying rates to bias the trees consistently and also lacks enough data to give a robust basal topology apart from consistently showing the A B dichotomy. Nonethless, as Petersen et al. (2014) also remarked, the trees are surprisingly good for such a short molecule and better than the vast majority of the 187 single-gene trees we ran for our 187-protein trees (e.g. Cavalier-Smith et al. 2015a) and share a large number of well-established features with those multiprotein trees, so are far from useless for evolutionary interpretation if considered critically. They show no evidence for lateral gene transfer independently of the established single red algal symbiogenesis.

Periplastid derlins (highlighted yellow in all trees) have N-terminal bipartite targeting peptides that are absent in typical endomembrane derlins labelled here as 'ER derlins' even though they may also be in endosome membranes.

For periplastid derlin B, both trees maximally support the halvarian subclade (Apicomplexa plus one of the two heterokont paralogues) and both show the same two other periplastid B subclades: heterokont/haptophyte with moderate to weak support (0.6, 48) and haptophyte only (strong) and all three as branching comparably deeply to the red algal/green plant split, i.e. an ancient not recent divergence. Both agree in placing all three periplastid derlin B clades closer to plant ER derlin Bs than to chromist host B paralogues; though statistical support for this is extremely low it suggests that chromist periplastid B derlins more likely came from the symbiont than from the host so did not need to undergo duplication before being retargeted to the periplastid compartment. On Fig. S5 all three

subclades form one clade (trivial 0.31 support) that is weakly sister to the red agal clade, but figs S1 and S2 with extra sequences, some long-branch, neither group them together nor place them consistently. Fig. S2 groups only the haptophyte-only subclade with the halvarian one, as sister to red algae the haptophyte/heterokont one being one node higher within red algae. Fig. S1 instead groups the two non-halvarian clades weakly (0.63) as a clade that wrongly attracts the green algal *Volvox*/*Chlamydomonas* subclade as sister, leaving the much longer halvarian subclade as sister to all plant plus other periplastid B sequences. Adding a lot more fungal sequences, some very long branch yeasts, to clarify the position of Dfm as well as long branch Eozoa and metamonads (*Trichomonas*, *Giardia*) causes further problems (Figs S4,5 with 193 taxa). The extremely divergent *Trichomonas* sequence attracts red algal derlin B togther with the diatom ER derlin B clade entirely wrongly into podiates on the CAT tree, so the periplastid Bs appear to group with green algae on Fig. S3. The ML tree shows the same attraction between these three taxa but the effect is quite different: *Trichomonas* and the diatom ER derlin B clade are attracted towards the red algae which correctly remain outside the podiates together with the periplastid derlin Bs (Fig. S5). Thus although both trees suffer from long-branch problems in this respect (not in all others), the ML tree appears more sensible than the CAT tree - a relatively rare example of CAT being topologically worse than ML (but without statistical support). Fig. S3 groups the two non-halvarian periplastid B clades more strongly (0.83) than Fig. S1 (0.63), but Fig. S4 groups just the haptophyte clade with the halvarian like Fig. S2 with similarly insignificant support (16 versus 18%) so the difference on topology of ML and CAT on the 143 and 193 sequence trees is independent of taxon sampling of podiate sequences - similar differences remain in the 203 sequence trees that include both *Trichomonas* paralogues (Figs S7, S8). However, there is less discrepancy in topology by ML and CAT with 122 taxa where many long branches are omitted. Fig. S6 ML groups all three periplastid B clades together with exactly the same mutual topology as the corresponding Fig. S5 CAT tree; in both the halvarian and heterokont/haptophyte clades are sisters with weak but significant support (0.59, 56%) and the haptophyte-only the deepest branch. However within halvaria both trees wrongly show alveolates as paraphyletic ancestors of heterokonts (insignificant suport) and the ML tree (Fig. S6) groups the periplastid clade not with red algae as did CAT but with reds plus *Volvox*/*Chlamydomonas* and the centric diatom ER clade.

CAT weakly (0.55) groups the **nucleomorph-coded cryptophyte periplastid derlin A** that **must be of red algal origin** with Plantae at the red/green split, showing corticates plus nucleomorphs correctly as a clade but ML wrongly makes corticates paraphyletic and does not group the nucleomorph sequences specifically with plants (but they would be nearby if B subtree were correctly rerooted bewteen corticates and podiates); the nucleomorph branch has evolved 2-3 times as fast as the parental red algal sequences, this and the weak taxon sampling for rhodophytes accounting for these inconsistencies.

The ML tree is less good than CAT in three other respects: (1) showing Apicomplexa periplastid derlin B as ancestral to that of heterokonts, not as a clade; (2) making rhodophyte B paraphyletic; (3) Sporozoa B ER not a clade; but the ML tree is probably better than CAT in showing Sporozoa A, Amoebozoa A, Viridiplantae B, Rhizaria B, and Hacrobia ER B all as clades. These differences reflect random errors because the protein is short.

Fig. S3. Derlin PhyloBayes CAT-GTR tree for 193 eukaryote sequences including Eozoa (maxdiff 0.0804493; burnin 490; 133, 495 trees summed). Eozoa also have distinct A and B paralogues, but branches are long and placed contradictorily: B is wrongly with Ciliophora; its presence plus that of the long-branch *Trichomonas* paralogue disrupt the B subtree. The *Trichomonas* sequence wrongly weakly groups with the host centric diatom clade and red algae; so red algae are wrongly attracted away from green plants and periplastid chromist sequences into podiates.

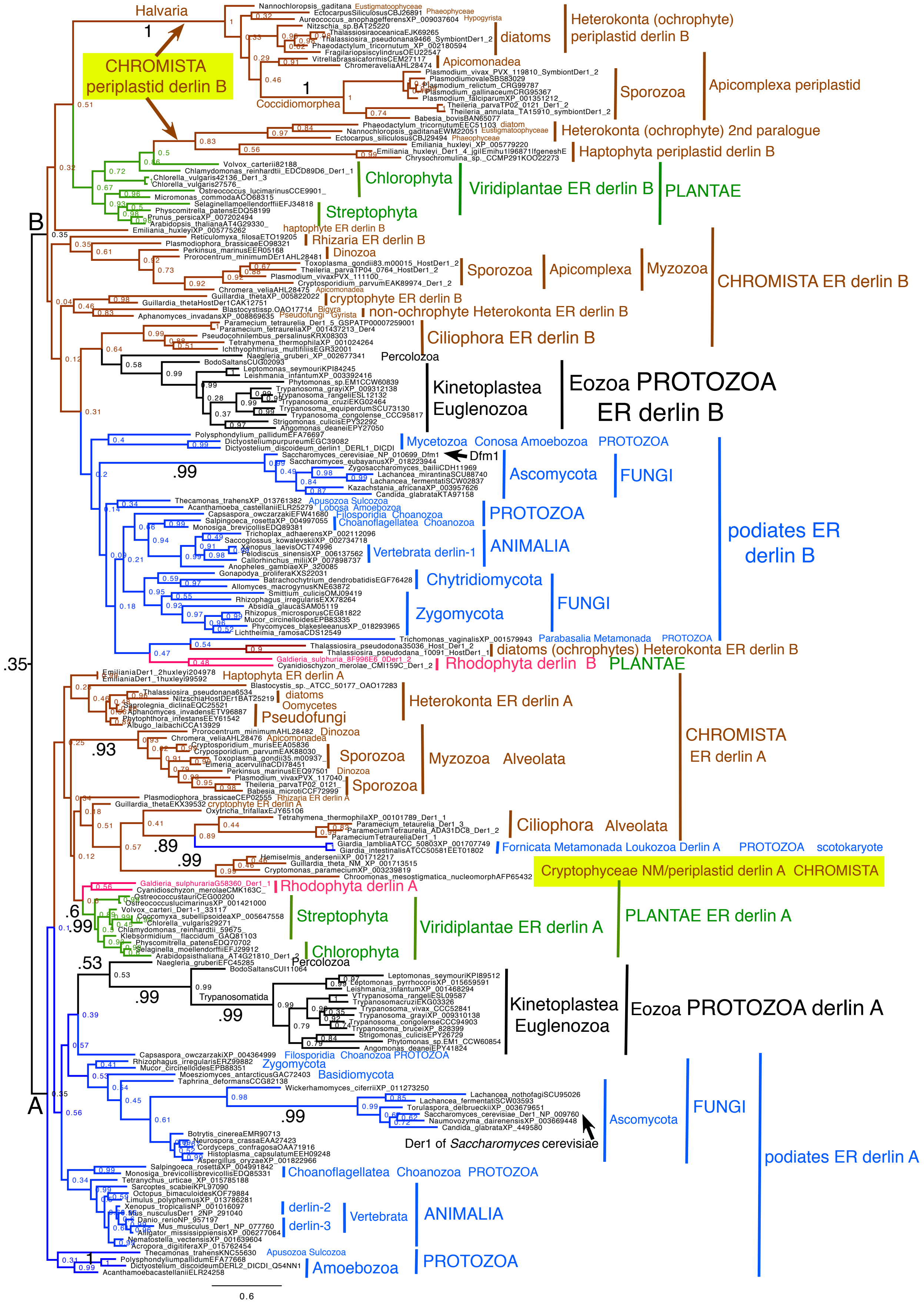

Fig. S4. Derlin RAxML-GTRGAMMAF tree for 193 eukaryote sequences including Eozoa (1000 fast bootstrap analysis). Adding the ancient long branch Eozoa and metamonads greatly reduces bootstrap support for the A/B dichotomy compared with Fig. S2 but does not affect the position of red algae B derlin as badly as with CAT (Fig. S3), but the *Trichomonas* sequence still wrongly groups with diatom ER B derlin. In this tree the *Giardia* sequences are wrongly placed with the B paralogues of budding yeasts, not with the A paralogues of ciliates as in Fig. S3; though both positions are clearly wrong, I assumed in my discussion that *Giardia*'s position as an A paralogue on the theoretically superior CAT tree is more likely to be correct, but sequencing derlins from a variety of short-branch metamonads is needed to test my conclusion that *Giardia* lost paralogue B, not paralogue A, and also to confirm that *Trichomonas* has one A and one B derlin. The putative *Trichomonas* A derlin has an even longer branch than the B paralogue and so was omitted from this tree in case it caused worse long-branch attraction. It is included in a 203-sequence analysis (Figs S7 and S8) omitting the most divergent, longer-branch rhizarian B paralogue (from *Reticulomyxa*) in an attempt to reduce long-branch problems. For Eozoa Fig. S4 ML tree is even worse than Fig. S3: not only are both paralogues clearly and contradictorily misplaced as on Fig. S4, but for the A paralogue the long-branch kinetoplastids do not group with *Naegleria* as they correctly did on Fig. S3 but wrongly moved into chromists to group with the atefactual ciliate/*Blastocystis* long-branch aggregate. Note that Figs S3 and S4 include both *Saccharomyces cerevisiae* paralogues Der1 (A) and Dfm1 (B) and extra yeast derlins; clearly only vertebrate derlin-1 is related to Dfm1, whereas derlin-2 and derlin-3 are both related to the yeast A paralogue Der1.

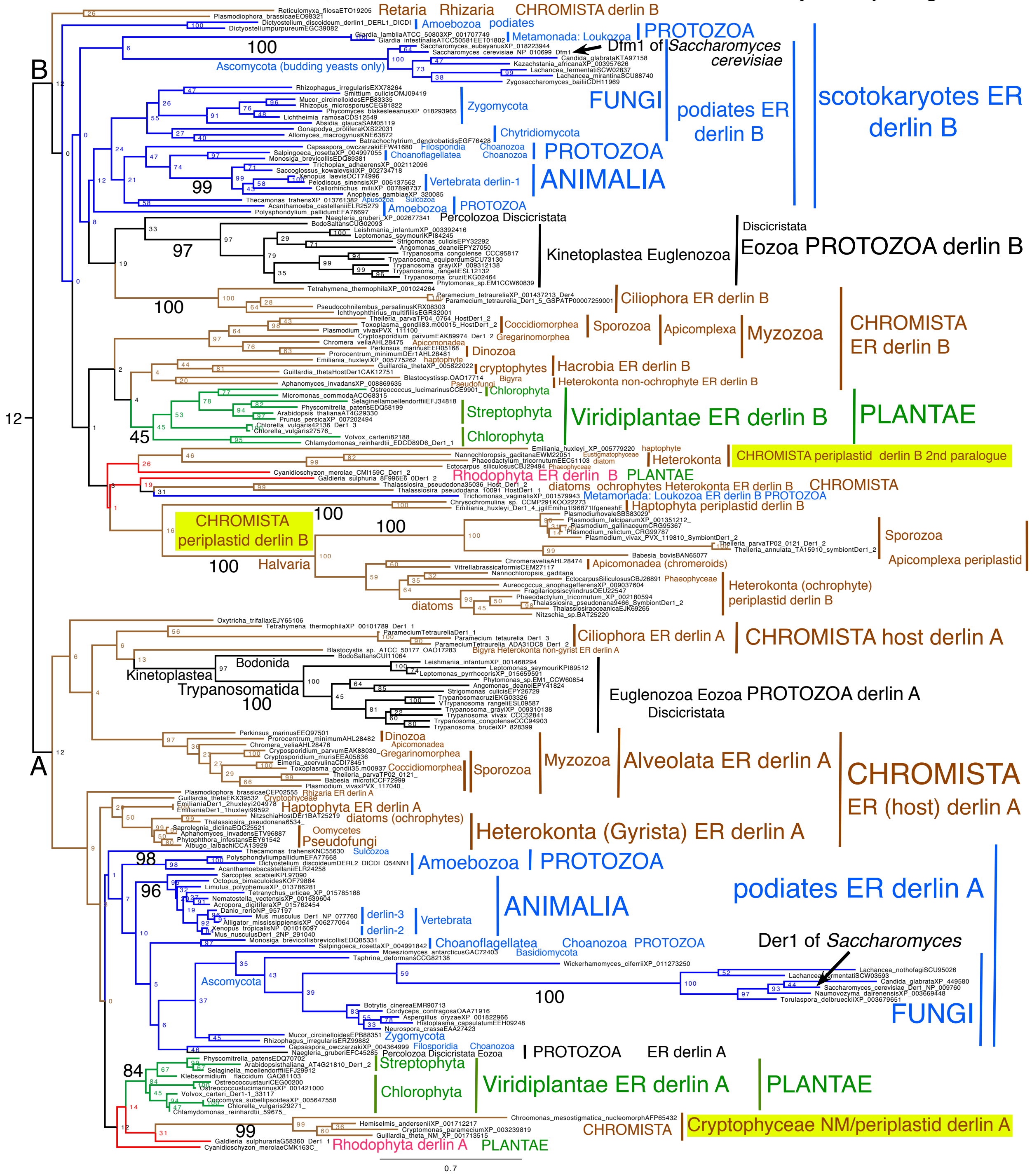

Fig. S5. Derlin PhyloBayes CAT-GTR tree for 122 neokaryote sequences (excluding Eozoa and metamonads) using 201 well-aligned amino-acid positions. Two chains were run as in all PhyloBayes trees in this paper until full convergence was reached (maxdiff 0.0873652; first 342 trees removed as burnin; 277,109 trees summed). Omitting several long-branch sequences included in the other trees (e.g. metamonads, some yeasts) greatly increased support for the A/B dichotomy and shows with weak support all three periplastid B paralogues as a single clade and sister to the red algal clade. Vertebrate derlin-2 and -3 group with 0.98 PP support with other paralogue A animal derlins and all paralogue A derlins including fungal derlins related to yeast Der1 (as shown in Figs S1- S4) are separated from all B derlins with 0.90 PP support, contrary to the statement that all mammalian derlins are more closely related to Dfm1 than to Der1 (Goder et al. 2008). The trees on which that assertion was based were so sparsely sampled that the ultralong Dfm1 and Der1 sequences, which both evolve much faster in budding yeasts than in other fungi, according to all my trees, grouped together as a long-branch artefact excluding the much shorter-branch but equally anciently diverged mammalian A and B sequences. The ML tree for these sequences is Fig. S6.

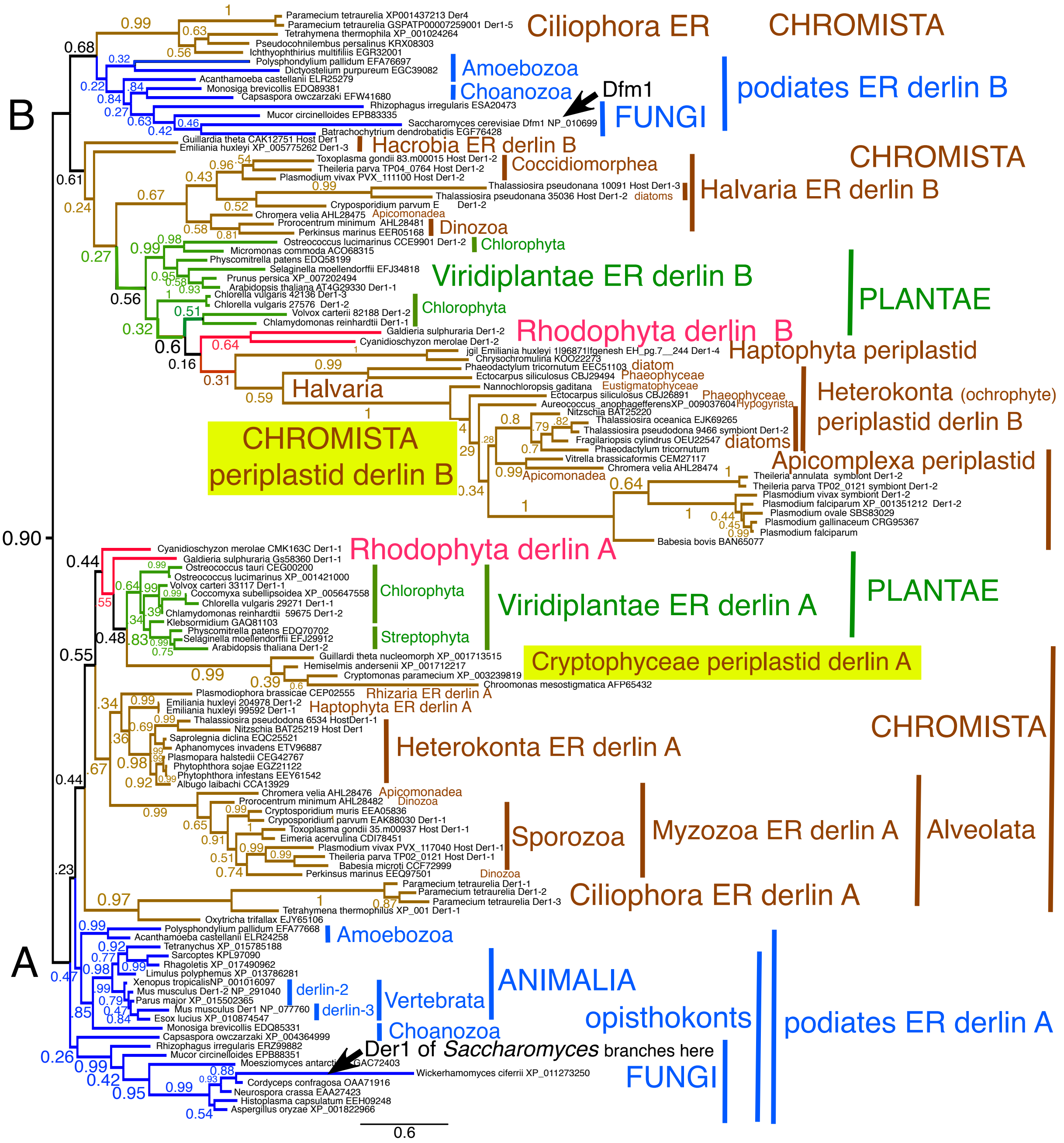

Fig. S6. Derlin RAxML GTRGAMMAF tree for 122 neokaryote sequences (excluding Eozoa); 1000 fast bootstrap analysis. Omitting several long-branch sequences included in the other trees (e.g. metamonads, some yeasts) greatly increased support for the A/B dichotomy and shows with weak support all three periplastid B paralogues as a single clade (same internal branching order as by CAT: Fig. S5); however unlike the probably more accurate site-heterogeneous Fig. S5 this periplastid clade is not sister to red algae alone but to a broader probably aretfactual grouping of red algae, centric diatom ER derlin, and Volvocales incorrectly separated from other green algae. It is however probably more realistic in weakly grouping all nuclear-coded chromist A paralogues extremely weakly in one clade in which ciliates are correctly sisters of Myzozoa as an alveolate clade. This means that for poorly supported clades some grouping by ML may by chance be better than with CAT. Though support for the A/B dichotomy has risen to 52%, within each of subclades A and B basal branching order is statistically entirely insignificant, as is expected for any single-gene tree given the probably explosive rapid radiation of neokaryotes. No single-gene trees robustly show the basal neokaryote dichotomy between scotokaryotes and corticates nor can resolve their basal branching order, which can be done only using hundreds of genes (Cavalier-Smith et al. 2015a).

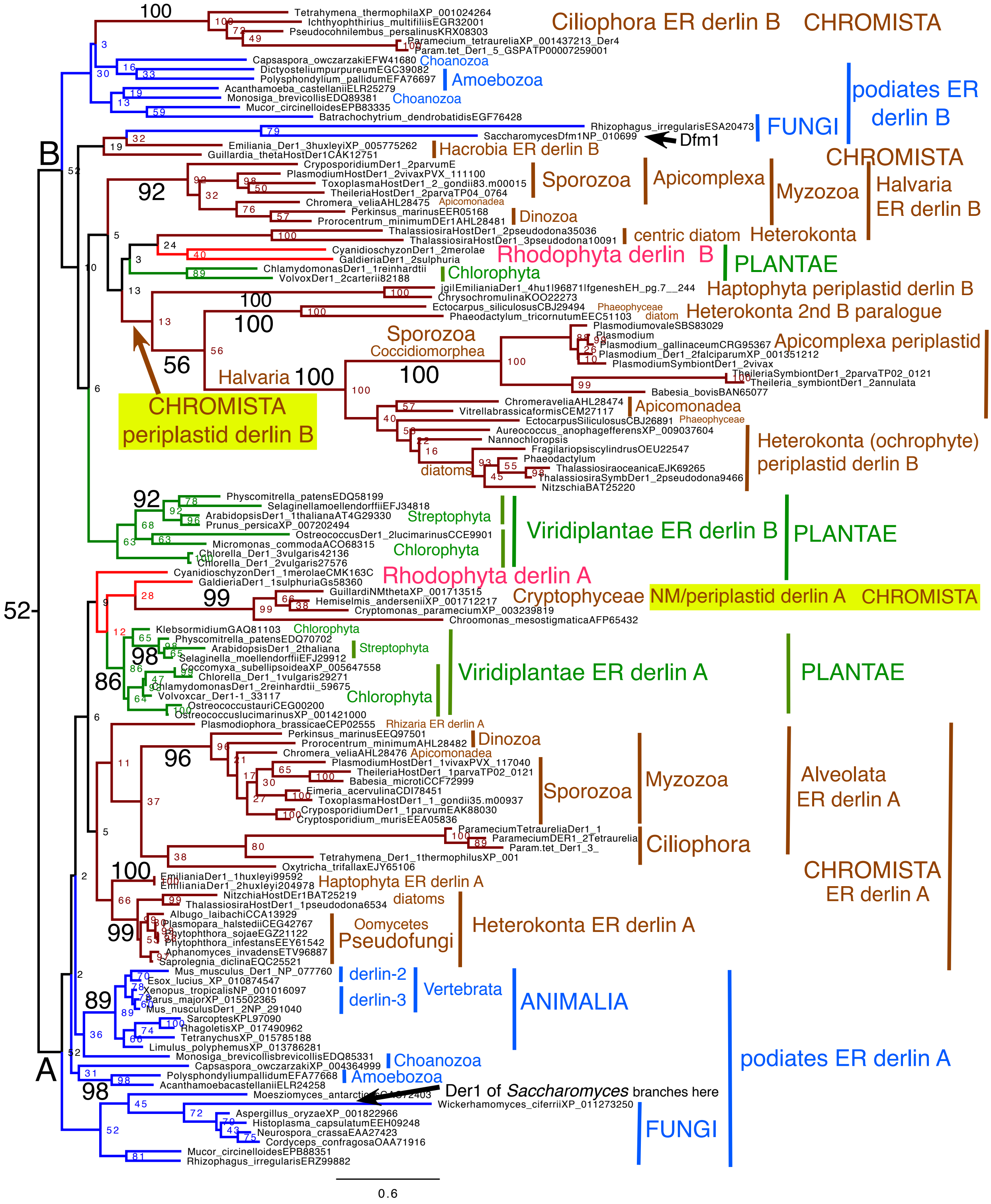

Fig. S7. Derlin PhyloBayes CAT-GTR tree for 203 eukaryote sequences (including Eozoa and metamonads but both *Trichomonas* sequences): maxdiff 0.0938794; burnin 997; 139,892 trees summed. The *Giardia* sequences remain wrongly within the Ciliophora A paralogues as in Fig. S3 for which the extremely long-branch *Trichomonas* putative paralogue A was omitted and *Reticulomyxa* B included: this *Trichomonas* sequence is also placed within ciliates A near to but not with *Giardia*. Including both *Trichomonas* paralogues and extra long-branch budding yeast Dfm1 paralogues and more animals than in Fig. S3 reduces further support for the A/B dichotomy but changes overall topology very little compared with Fig. S3.

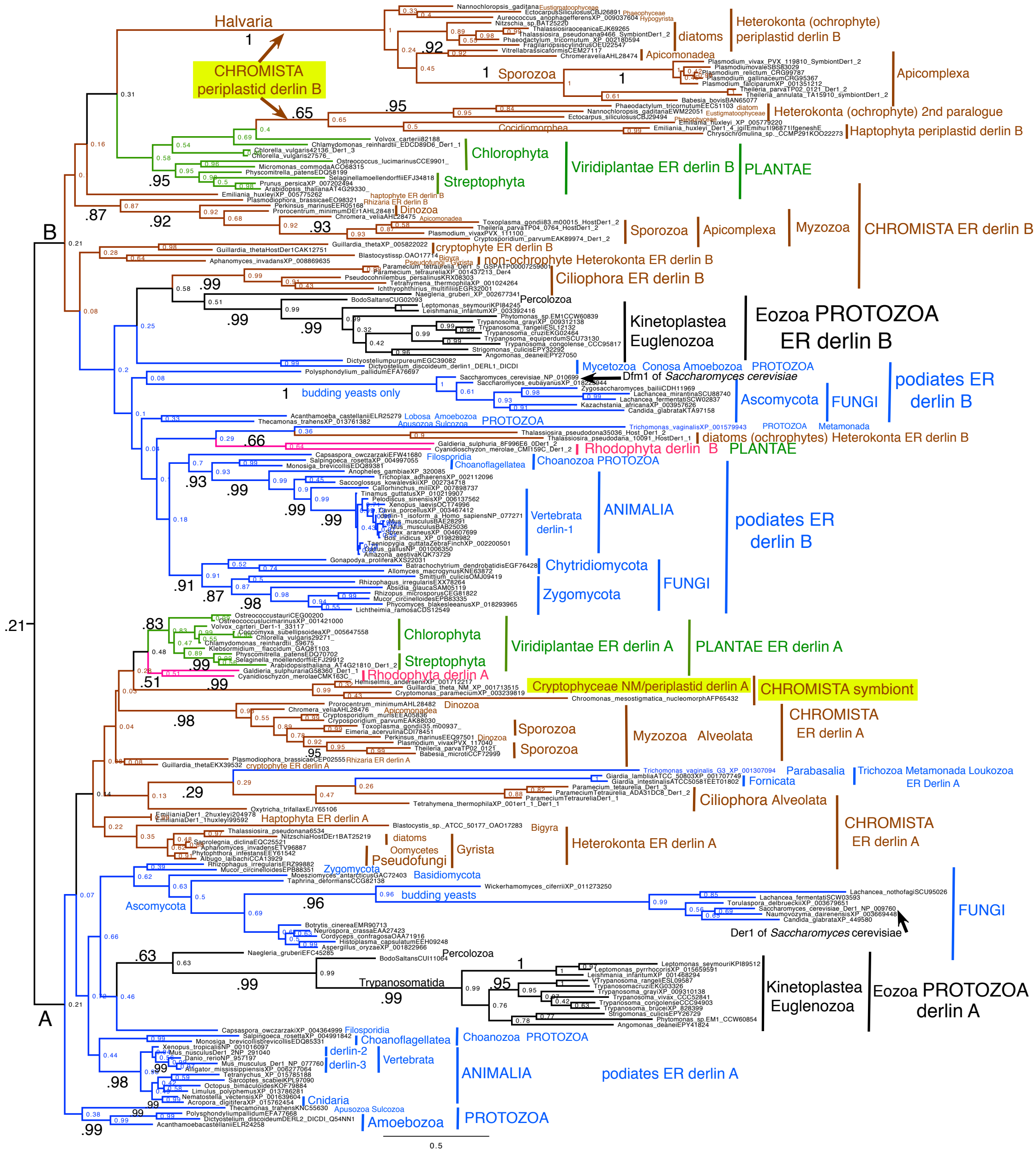

Fig. S8. Derlin RAxML GTRGAMMAF tree for 203 eukaryote sequences (including Eozoa and metamonad same alignment as Fig. S6); 100 fast bootstrap analysis for same sequences as Fig. S7 CAT tree. Compared with Fig. S3 where the extremely long-branch *Trichomonas* putative paralogue A was omitted, this *Trichomonas* sequence is wrongly placed within ciliate A paralogues as on Fig. S7, but the *Giardia* sequences have moved away from their position within Ciliophora A as seen on CAT trees to falsely group with the long-branch budding yeast paralogue B clade (which only groups correctly with other fungi on trees with only 122 or 153 sequences that exclude many of the longest-branch sequences). This probably incorrect position essentially removed support for the A/B dichotomy but did not significantly change the rest of the tree. Future sequences for short-branch metamonads and shorter-branch yeast paralogue Bs are essential for resolving this conflict and testing my assumption that the changed position of *Giardia* in ML trees (Figs S4 and S8 compared with corresponding Figs S3 and S7 CAT trees) is a long-branch artefact. This is the only ML tree where non-halvarian periplastid B sequences (yellow highlights) were not a single clade.

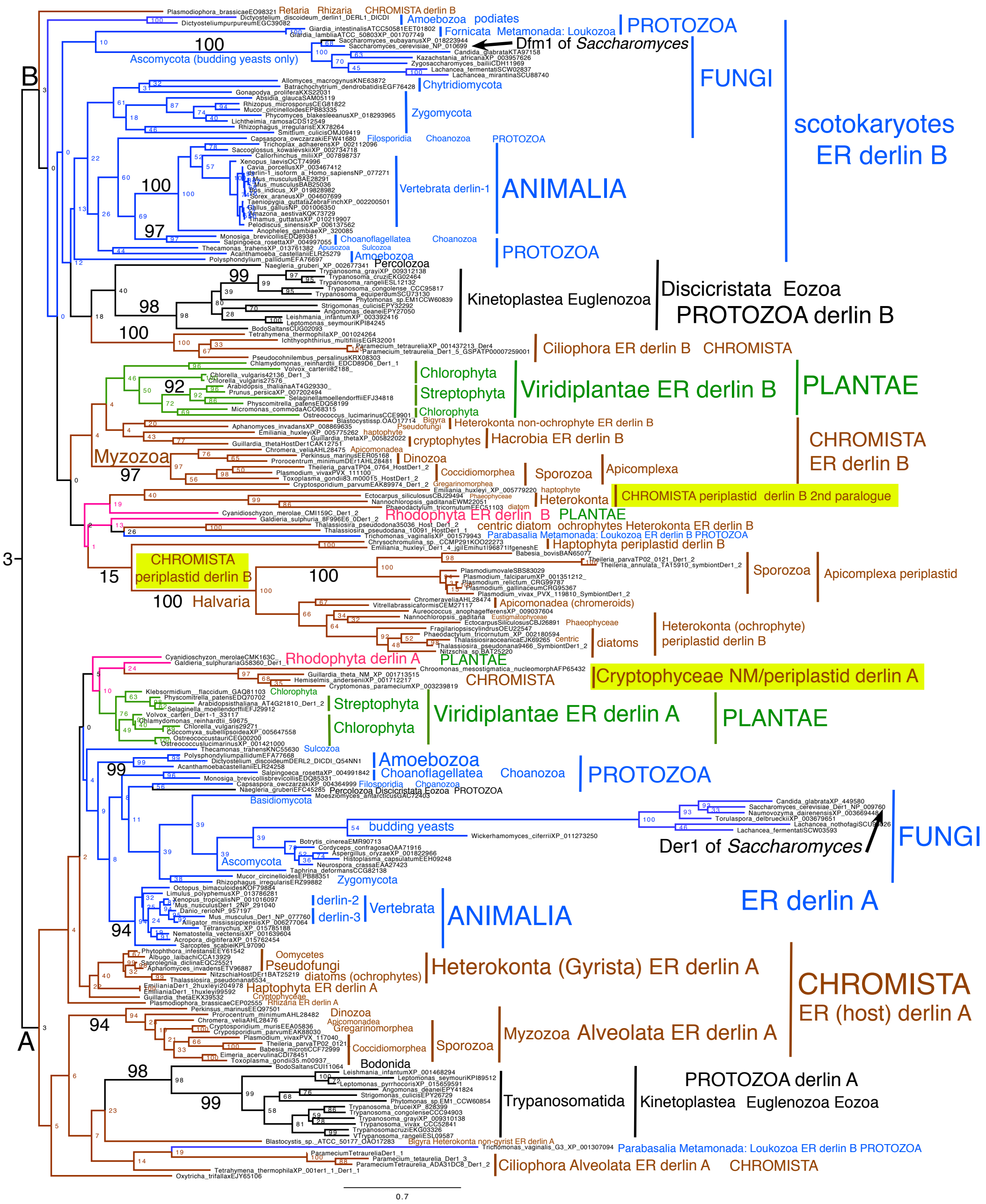

Table S1. Classification of kingdom Chromista Cavalier-Smith, 1981 and its 8 phyla and 82 classes

---

Subkingdom 1. Harosa Cavalier-Smith, 2010 (6 phyla 71 classes)
Infrakingdom 1. Halvaria Cavalier-Smith, 2013 (4 phyla 53 classes)
Superphylum 1. Heterokonta Cavalier-Smith, 1981 (=stramenopiles: tripartite anterior ciliary tubular hairs) stat. n. Diagnosis as for division Heterokonta Cavalier-Smith (1986 p. 338) (2 phyla; 25 classes 4 new)
Phylum 1. Gyrista Cavalier-Smith, 1998 stat. n. (16 classes, 12 mainly algal; 2 new)
Subphylum 1. Ochrophytina* Cavalier-Smith, 1986 (plastids inside rough ER; e.g. diatoms (silica frustules), brown algae, chrysomonads, xanthophytes, raphidophytes (cortical alveoli), Actinophryida; 12 ancestrally photosynthetic classes, 3 including important heterotrophs)
Infraphylum 1. Chrysista* Cavalier-Smith, 1991 (ancestrally with ciliary supra-tz helix)
Superclass 1. Limnistia* Cavalier-Smith, 1996 emend. 2006 (naked, often scaly, or phagotrophic sometimes heterotrophic or walled photosynthetic coccoid unicells; mostly freshwater)
Class 1. Eustigmatophyceae* Hibberd & Leedale, 1971 (e.g. *Vischeria, Nannochloropsis*)
Class 2. Chrysomonadea* Saville-Kent, 1881 stat. n. Pascher, 1910 emend. auct. (=Chrysophyceae) (e.g. *Chromulina*, *Chrysamoeba*, *Oikomonas*, *Paraphysomonas***, *Ochromonas*, *Spumella*, *Dinobryon*, *Uroglena*, *Hibberdia*, *Chromophyton*, *Mallomonas*, *Synura*, *Tessellaria*)
Class 3. Picophagea* Cavalier-Smith, 2006 em. (= Synchromophyceae Horn et al., 2007: *Chlamydomyxa labyrinthuloides* and *montana*, *Chrysopodocystis*, *Guanochroma*, *Leukarachnion*, *Picophagus*, *Synchroma*)
Superclass 2. Raphidoistia* Cavalier-Smith, 1986 orth. mut. 2006 (naked alveolate phagoheterotrophs and phototrophic unicells; marine and freshwater)
Class Raphidomonadea* Chadefaud ex Silva, 1980
Subclass 1. Raphidophycidae*+ Cavalier-Smith in Cavalier-Smith and Scoble, 2013 (phototrophs, e.g. *Chattonella, Fibrocapsa, Haramonas, Heterosigma*)
Subclass 2. Raphopoda Cavalier-Smith in Cavalier-Smith and Scoble, 2013 (axopodial heterotrophs: *Actinophrys*, *Actinosphaerium*, *Commation*, *Heliorapha*)
Superclass 3. Fucistia* Cavalier-Smith, 1995 (non-phagotrophic, walled marine multicellular algae)
Class 1. Chrysomerophyceae* Cavalier-Smith in Cavalier-Smith et al., 1995 (e.g. *Giraudyopsis*)
Class 2. Xanthophyceae* Allorge ex Fritsch, 1935 (e.g. *Vaucheria, Pleurochloridella*)
Class 3. Aurophyceae* Cavalier-Smith in Cavalier-Smith and Scoble, 2013
Subclass 1. Aurearenophycidae* Kai et al. 2008 stat. n. Cavalier-Smith in Cavalier-Smith and Scoble, 2013 (*Aurearena*)
Subclass 2. Phaeothamniophycidae* Cavalier-Smith in Cavalier-Smith & Chao, 2006 em. 2013 (= class Phaeothamniophyceae Andersen & Bailey in Bailey et al., 1998) (e.g. *Phaeothamnion*, *Stichogloea*, *Phaeoschizochlamys*)
Class 4. Phaeophyceae* Kjellman, 1891 (brown algae *sensu lato*)
Subclass 1. Schizocladiophycidae* Cavalier-Smith in Cavalier-Smith & Scoble, 2013 (=Schizocladiophyceae Henry et al. in Kawai et al., 2003) (*Schizocladia*)
Subclass 2. Melanophycidae* Rabenhorst, 1863 stat. n. Cavalier-Smith & Chao, 2006 (brown algae *sensu stricto*) 15 orders (Clayton 1990) e.g. Ectocarpales, Laminariales, Fucales (e.g. *Fucus*)
Infraphylum 2. Diatomista* Derelle et al. ex Cavalier-Smith, 2017 infraphyl. n. **Diagnosis:** typically unicells, sometimes in diatoms linear loose aggregates of cells; no cell walls; naked or with intracellular secreted silica frustules or siliceous scales; biciliate, anteriorly or posteriorly uniciliate or non-ciliate, without supra-tz helix.
Superclass 1. Hypogyrista* Cavalier-Smith, 1995 stat. n. 2006 (ciliary transition zone rings prominent; phototrophs, phagophototrophs. phagoheterotrophs)
Class 1. Dictyochophyceae* Silva, 1980 (naked, sometimes axopodial)
Subclass 1. Pedinellia* (=Pedinellophycidae =Actinochrysea/ia) Cavalier-Smith, 1986 stat. n. (e.g. *Dictyocha*, *Pedinella*, *Pteridomonas**, *Rhizochromulina*)
Subclass 2. Pelagophycidae* Andersen & Saunders 1993 ex Cavalier-Smith in Cavalier-Smith & Chao, 2006 (*Ankylochrysis*, *Aureococcus*, *Pelagomonas*, *Sarcinochrysis*)
Subclass 3. Sulcophycidae* Cavalier-Smith in Cavalier-Smith & Scoble, 2013 (*Olisthodiscus*, *Sulcochrysis*)
Class 2. Pinguiophyceae* Kawachi et al., 2002 (e.g. *Glossomastix*, *Phaeomonas*)
Superclass 2. Khakista* Cavalier-Smith, 2000 (as subphylum) stat. n. (**Diagnosis:** no ciliary roots; silica frustules or scales; chloroplasts with girdle lamellae, fuxoxanthin, diadinoxanthin, diatoxanthin; almost all phototrophs)

Class 1. Bolidophyceae *Guillou and Chretiennot-Dinet, 1999 (Parmales: *Triparma*)
Class 2. Diatomeae* Dumortier, 1822 stat. n. auct. (syn. Bacillariophyceae)
Subclass 1. Corethrophycidae* Round and Crawford, 1990 (e.g. *Corethron*)
Subclass 2. Rhizosoleniophycidae* Round and Crawford, 1990 (e.g. *Rhizosolenia*)
Subclass 3. Eucentricophycidae* Cavalier-Smith, 2000 (e.g. *Coscinodiscus*, *Thallassiosira*)
Subclass 4. Bacillariophycidae* Pennatia Schütt, 1896 (=Pennatophycidae) (e.g. *Asterionella*, *Bacillaria*, *Diatoma*, *Fragilaria*, *Navicula*, *Nitzschia*, *Phaeodactylum*, *Rhaphoneis*)

Subphylum 2. Bigyromonada+ Cavalier-Smith, 1998 (marine biciliate phagoheterotrophs)

Class 1. Developea cl. n. Aleoshin et al. 2016 ex Cavalier-Smith, 2017 (e.g. *Developayella, Develorapax*). **Diagnosis**: biciliate non-amoeboid phagoheterotrophs; cortical alveoli underly part of cell surface; 6-gyre, obviously double TH; one or two retroneme rows. **Etymol:** truncation of names of included genera; replacement name (proposed by Aleoshin et al. (2016) for Biyromonadea Cavalier-Smith (1997) that may not have been validly published as the reference to its validating publication was incomplete through its publication being delayed (Cavalier-Smith 1998) and including no explicit validation of the class). As the sole order and family may be invalid for the same reason, I hereby validate order Developayellida Cavalier-Smith (1987 p. 256) and family Developayellidae Cavalier-Smith (1997 p. 256) under ICZN by stating that both share the same diagnosis with Developea.

Class 2. Pirsonea cl. n. (*Pirsonia*) **Diagnosis**: as for sole order Pirsoniida (Cavalier-Smith and Chao 2006 p. 404). Includes also environmental DNA clades MAST1/23, 2 and Aleoshin et al.'s (2016) Ochrophytina-associated grade if heterotrophs.

Subphylum 3. Pseudofungi Cavalier-Smith, 1986 (walled heterotrophic osmotrophs)
Class 1. Oomycetes Winter in Rabenhorst, 1879 (e.g. *Pythium*, *Phytophthora*)
Class 2. Hyphochytrea Cavalier-Smith, 1986 (e.g. *Hyphochytrium*, *Rhizidiomyces*)

Phylum 2. Bigyra Cavalier-Smith, 1998 em. 2006 (heterotrophs; most wall-less phagotrophs; 9 classes)

Subphylum 1. Opalozoa Cavalier-Smith, 1991 em., stat. n. 2006 (6 classes)

Infraphylum 1. Placidozoa Cavalier-Smith in Cavalier-Smith and Scoble, 2013

Superclass 1. Wobblata Cavalier-Smith in Cavalier-Smith and Chao, 2006 stat. n. 2013
Class 1. Placididea Moriya et al., 2002 (*Placidia*, *Wobblia*)
Class 2. Nanomonadea Cavalier-Smith in Cavalier-Smith and Scoble, 2013 (*Incisomonas*, *Solenicola*)
Class 3. Opalomonadea Cavalier-Smith in Cavalier-Smith & Scoble, 2013 (MAST-12)

Superclass 2. Opalinata Wenyon, 1926 em. Cavalier-Smith, 2006 stat. n. 2013
Class 1. Opalinea Wenyon, 1926 stat. n. Cavalier-Smith, 1993 em. 2013 (e.g. *Cepedea*, *Karotomorpha*, *Opalina*, *Proteromonas*)
Class 2. Blastocystea Zierdt, 1978 (*Blastocystis*)

Infraphylum 2. Bikosia cl. n. **Diagnosis** as for subclass Bikosidae (Cavalier-Smith and Chao 2006 p. 404). Introduced as a clade name in Cavalier-Smith and Scoble (2013); validated here as a new infraphylum, as then intended, by providing a previously omitted explicit reference to a diagnosis.

Class Bikosea Cavalier-Smith, 1986 (as Bicosoecea), orth. em. 2013
Subclass 1. Bikosidia Cavalier-Smith, 2006 orth. em. 2013 [orders Bicoecida (*Bicosoeca*), Anoecida (e.g. *Cafeteria*, *Caecitellus*), Pseudodendromonadida (e.g. *Adriamonas*, *Filos, Nanum, Nerada, Siluania*), Borokida (*Boroka*)]
Subclass 2. Rictidia Cavalier-Smith, 2013 (*Rictus*)

Subphylum 2. Sagenista Cavalier-Smith, 1995 stat. n. 2006 (2 classes, 1 new)

Class 1. Eogyrea class n. Diagnosis: Phagotrophic biciliate planktonic/benthic bigyrans with R3 and R4 anterior and split posterior R2, singlet and R1 mt centriolar roots but no X mt (unlike most Bikosia; undulating anterior cilium with 2 rows of bipartite retronemes. Originally name for clade L (Cavalier-Smith and Scoble 2013; phylogenetically closer to Labyrinthulea than to Opalozoa, Derelle et al. 2016), comprising MAST-4, MAST-6 (e.g. *Pseudophyllomitus* in new order Eogyrida **Diagnosis:** cylindrical supra-transition helix (TH), typically swimmers, motion spiral, not gliders; subapical ciliary depression, not ventral groove. **Etym**. *Eo* Gk early, *gyrus* L. circle, as earliest branch with TH; sole family Pseudophyllomitidae Shiratori et al. (2017), and MAST-7-11 **Comment:** Eogyrida diagnosis is based on marine *P. vesiculosus*; its name is not based on the genus in case type species *Pseudophyylomitus granulatus* which can sometimes glide represents a separate heterokont genus or even family; *Pseudophyllomitus apiculatus* Skuja, 1948 is so different that it probably belongs in another genus, perhaps even order or class.

Class 2. Labyrinthulea (Lister, 1891) Olive ex Cavalier-Smith, 1986 (scaly, predominantly osmotrophic, e.g. *Aplanochytrium*, *Diplophrys*, *Thraustochytrium*)

Bigyra incertae sedis: Class Platysulcea cl. n. Diagnosis as for sole order **Platysulcida** ord. n. **Diagnosis:** naked phagotrophic biciliates, glide on long posterior cilium associated with ventral feeding groove or swim with wobbling motion; R3 and R4 anterior and split posterior R2, singlet and R1 mt centriolar roots;

undulating anterior cilium with 2 rows of short bipartite retronemes; no TH. **Etym**: *platy* L. wide, *sulcus* L. groove. Sole family Platysulcidae Shiratori et al., 2015. (*Platysulcus*)

Superphylum 2. Alveolata Cavalier-Smith, 1991 stat. n. 2013 (cortical alveoli; 28 classes 4 new)

Phylum 1. Miozoa Cavalier-Smith, 1987 (ciliary hairs non-tubular; uninucleate, usually haploid; 16 classes, 3 new)

Subphylum 1. Protalveolata+ Cavalier-Smith, 1991 stat. n. 1999 em. (heterotrophic phagotrophic biciliates; myzocytosis unknown)

Class 1. Colponemea Cavalier-Smith, 1993 ventral groove for phagocytosis

Order 1. Colponemida Cavalier-Smith, 1993 (*Colponema*)

Order 2. Palustrimonadida ord. n. **Diagnosis:** ventrally grooved biciliates differing from Colponemida by being less flattened, more rigid, and anterior cilium emerging from deep pocket separate from main longitudinal ventral groove. Contains only new family **Palustrimonadidae** with same diagnosis; type genus *Palustrimonas* Patterson and Simpson (1966 p. 443).

Class 2. Acavomonadea Tikhonenkov et al., 2014

Order Acavomonadida Tikhonenkov et al., 2014 (*Acavomonas*)

Subphylum 2. Myzozoa* Cavalier-Smith and Chao, 2004 (myzocytotic; cytosolic chloroplasts (type II RuBisCo) or leucoplasts; epiplastid membrane separate from rough ER)

Infraphylum 1. Dinozoa* Cavalier-Smith, 1981 stat. n. 2013 em. (10 classes)

Parvphylum 1. Perkinsozoa Norén and Moestrup in Norén et al, 1999 em. Cavalier-Smith, 2014 stat. n. (normal nuclear chromatin)

Class 1. Perkinsea** Levine, 1878 Orders Perkinsida Levine, 1978 (*Perkinsus*); Rastrimonadida Cavalier-Smith in Cavalier-Smith and Chao, 2004 (*Rastrimonas*, *Parvilucifera*); Acrocoelida Cavalier-Smith in Cavalier-Smith and Chao, 2004 (*Acrocoelus*: transferred here from Apicomonadea).

Class 2. Squirmidea Cavalier-Smith, 2014 (*Filipodium*, *Platyproteum*)

Parvphylum 2. Dinoflagellata* Bütschli, 1885 stat. n. em. (Phycodnavirus-like basic chromatin proteins; 8 classes 3 new)

Superclass 1. Eodina supercl. n. **Diagnosis:** Free-living ancestrally with ciliary web scales and posterior criss-cross latticed posterior ciliary lattice, two pronounced ciliary grooves; anterior groove separating rounded cell anterior and posterior oblique or transverse but not a helicoidal cingulum (unlike cingulate dinoflagellates: Syndina and Dinokaryota. Nuclear chromatin ultrastructurally normal.

Class 1. Myzodinea cl. n. Diagnosis: Laterally biciliate myzocytotic predatory zooflagellates with discrete, often swollen cortical alveoli and extremely pronounced transverse or oblique anterior ciliary groove; rounded cell apex (non-rostrate, unlike most Apicomonadea) with micronemes and/or rhoptry-like dense extrusomes, and pseudoconoid-like short microtubules connected to long band of microtubules bypassing kinetid; ancestrally with ciliary web scales and singlet posterior microtubular root centrally supporting posterior groove floor; anterior ciliary hairs; ciliary transition zone with concave-sided cone, central pair with 2 laterobasal axosomes. Bipartite trichocysts with square cross-section dense basal zone. Unlike Peridinea, Sulcodinea, and *Oxyrrhi*s, left posterior ventral centriolar root more strongly developed than right. **Sole order Myzodinida** ord. n. **Diagnosis**: as for Myzodinea. **Colpovoridae** fam. n. diagnosis as for its type genus ***Colpovora*** gen. n. **Diagnosis:** posterior right centriolar root of about 12 microtubules without I fibre; left root with at least 3 microtubules; posterior cilium with paraxonemal rod with cross lattice as in *Oxyrrhis*; anterior cilium with simple hairs. Oblique/transverse binary cell division not within cyst. Centriole angle slightly obtuse. **Etymology**: *Colpos* Gk womb, vagina, cleft, referring to transverse and longitudinal grooves. *voro* L. I devour, referring to predatory feeding. Type species *Colpovora unguis* comb. n. Basionym *Colpodella unguis* Patterson & Simpson (1996 p. 439). **Psammosidae** fam. n. **Diagnosis**: both cilia covered by oval cobweb scales and two hair rows; hairs with thicker, non-rigid shaft and 1-2 terminal filaments. Centriole angle strongly obtuse, much less than 180°, unlike Algovorida and Colpovoridae. Transverse binary division. Type genus *Psammosa* Okamoto et al. (2012).

Class 2. Oxyrrhea** Cavalier-Smith, 1987 (*Oxyrrhis*)

Superclass 2. Syndina Cavalier-Smith, 1993 em. (parasites; zoospores with helicoidal cingulum; euchromatin)

Class 1. Endodinea cl. n. Diagnosis: Parasites of Rhizaria, Alveolata, and fish eggs. Phylogenetically defined as all dinoflagellates more closely related to *Ichthyodinium* and *Dubosquella* than to *Syndinium* or *Oxyrrhis* (i.e. group I marine alveolates). Multiply within sporangia; nucleus with normal chromatin. Without body or ciliary scales. Cilia without paraxonemal rods or vanes. Contains only new order **Ichthyodinida,** diagnosis as for Endodinea. Includes Dubosquellidae Chatton 1920 ex Loeblich II, 1970 (e.g. *Dubosquella*) and new family **Ichthyodiniidae: Diagnosis:** Endoparasites of fish eggs; comprises lineages phylogenetically closer to *Ichthyodinium* than to *Dubosquella*. Type genus *Ichthyodinium* Hollande and Cachon, 1952.

Class 2. Ellobiopsea Loeblich III, 1970 (*Ellobiopsis*, *Thallassomyces*)

Class 3. Syndinea Chatton, 1920 stat. n. Loeblich, 1976 em. (without plastids or bacterial nuclear histone-like proteins, e.g. *Amoebophrya*, *Haematodinium*)

Superclass 3. Dinokaryota* Cavalier-Smith, 1993 em. (Histone-like protein HLP-II; liquid crystalline nuclear DNA organisation)***

Class 1. Peridinea* Ehrenberg, 1830 (shallow-gyre cingulum, usually equatorial; histone-

like protein HLPI)

Subclass 1. Dinophycidae* Bourelly, 1970 (peridinin plastids, triple envelope)

Infraclass 1. Peridinoidia* Poche, 1913 stat. n. Fritsch, 1935 (thecate, e.g. *Alexandrium*, *Ceratium*, *Crypthecodinium***, *Dinophysis*, *Peridinium*, *Prorocentrum*, *Gonyaulax*, *Heterocapsa*, *Polarella*, *Symbiodinium*, *Thoracosphaera*)

Infraclass 2. Gymnodinoidia* Poche, 1913 stat. n. Cavalier-Smith, 1993 em. (=Gymnodiniphycidae Fensome et al., 1993, non-thecate.

Orders Gymnodiniales Apstein, 1909 (e.g. *Gymnodinium*, *Spiniferodinium*, *Lepidodinium, Dissodinium*, *Togula*); **Spirodinida ord. n. diagnosis**: episomal microtubules terminate substantially subapically at a spiral microtubule bounding an apical spiral groove curving clockwise seen from apex. Includes **Akashiwidae fam. n.** diagnosis as for Spirodinida (Type genus *Akashiwo* Hansen and Moestrup in Daugbjerg et al. (2000 p. 308))

Infraclass 3. Epidinia infracl. n. **Diagnosis:** episome much larger than hyposome.

Torodinida ord n. **Diagnosis:** as for the infraclass (*Torodinium*, *Labourodinium*)

Subclass 2. Karlodinia* subcl. n. **Diagnosis:** plastids of haptophyte origin with 19-hexanoyl-fucoxanthin, not peridinin, with double envelope; cingulum steeply loop-like; divides small pointed epicone from large rounded hypocone (*Brachidinium*, '*Karenia*', *Karlodinium*, *Takayama*)

Class 2. Sulcodinea* cl. n. **Diagnosis:** dinokaryotes with either very long anterior sulcal extension so cingulum starts less than one third of cell length from its pointed apex (*Gyrodinium*) or with sulcus merging into an initially longitudinal cingulum about one third from apex that loops steeply round narrowly pointed cell apex and its cytoskeleton passing backwards ventrally parallel to sulcus (*Amphidinium*). Plastids triple envelope. With 2 orders:

Gyrodinida (e.g. *Gyrodinium*) ord. n. **Diagnosis:** heterotrophs with spiral cingulum. **Amphidinida** ord. n. **Diagnosis:** plastids with peridinin and triple envelope; cingulum steeply loop-like, divides small pointed epicone from large rounded hypocone (*Amphidinium*, *Bispinodinium*).

Class 3. Noctilucea Haeckel, 1866 stat. n. Cavalier-Smith, 1993 (e.g. *Noctiluca*)

Infraphylum 2. Apicomplexa* Levine 1970 em., stat. n. Cavalier-Smith, 2013 (centriolar doublets; conoid or paraconoid with two lumenal microtubules: 4 classes)

Parvphylum 1. Apicomonada* Cavalier-Smith, 1993 (subphyl.) stat. n., em.

Class Apicomonadea* Cavalier-Smith, 1993 em. **Revised diagnosis:** myzocytotic biciliates, predominantly apically rostrate, with posterior pointing cilia and pseudoconoid (apically truncated cone of mts with dense arms, closed as complete or almost complete ring at apex, open at ventral lower side) and 0-5 eccentric lumenal microtubules, rarely simplified to non-microtubular paraconoid (*Colpodella*) or missing (*Vitrella* only), and micronemes; heterotrophic or photosynthetic; ciliary centre pair microtubules ancestrally with separate globular axosomes and surrounded by slender basal cylinder just distal to axosome pair; ciliary transition zone with proximal, not distal dense plate. Multi-microtubule right anterior and posterior ciliary roots; no singlet microtubule between left and right posterior roots.

Subclass 1. Myzomonadia Cavalier-Smith in Cavalier-Smith and Chao, 2004 stat. n., em. **Diagnosis:** with pseudoconoid or paraconoid; phototrophs or heterotrophs; divide within cysts into 2, usually 4, or 8 cells.

Superorder 1. Chromovoridia* Diagnosis: photosynthetic or heterotrophic myzocytotic predators with preciliary rostrum containing a pseudoconoid of numerous mts, having 2-3 lumenal microtubules; encysted cells divide into four daughters, but in some vegtative cells undergo binary fission. **Etym:** *khroma* Gk colour; *voro* L. I devour.

Order 1. Chromerida* Moore et al., 2008 (as phylum!) stat. n. Cavalier-Smith, 2013 (*Chromera*: chloroplasts; pseudoconoid closed by anterior centriolar root; biciliate phase with normal length orthogonal centrioles; inflated cortical; alveoli with dense contents)

Order 2. Voromonadida Cavalier-Smith in Cavalier-Smith & Chao, 2004 (full length orthogonal centrioles; trichocysts; leucoplast). Family 1. Voromonadidae** Cavalier-Smith in Cavalier-Smith & Chao, 2004 (*Voromonas pontica*: discrete inflated cortical alveoli, contents light; micronemes, rhoptries). Family 2. Chilovoridae Cavalier-Smith in Cavalier-Smith & Chao, 2004 (highly compressed cortical alveoli; both cilia with amorphous paraxonemal rods; no rhoptries: *Chilovora perforans*; the structure left of the mitochondrion in figs 5 and 6 of Brugerolle and Mignot (1979) may be an apicoplast).

Order 3. Voracida ord. n. **Diagnosis:** no trichocysts; unlike all other apicomonads, centrioles extremely short, basally chamfered, not mutually orthogonal, joined by unique lamellate desmose; highly compressed cortical alveoli, not obviously subdivided in thin sections; anterior cilium in pit with a micropore, with lateral paraxonemal rod basally; its single mt root supports cell apex. **Etymol:** *vorax* L. voracious. **Microvoracidae fam. n.** Diagnosis as for type genus ***Microvorax* gen. n.:** cell apex rounded, not pointed as in *Dinomonas*, *Chilovora, Colpodella*; cilia only slightly subapical, one points anteriorly; centrioles close, only anterior (slender paraxonemal rod) in shallow pit, about one centriole-width apart with short desmose; small pimple-like cell protuberance between them; without oblique root; unlike *Dinomonas* posterior cilium at cell surface, not in pit. Feed on bodonids or ciliates; freshwater. **Etymol:** *micros* Gk small. Type species *Microvorax angusta* sp. n. (Syn. *Spiromonas angusta* sensu Krylov and Mylnikov, 1986; **not** *Heteromita angusta* Dujardin, 1841). **Diagnosis:** elongate cell 8-10(-18) X 3-4(-10) µm; cilia ~1.6X cell length; pseudoconoid of 24-5 strongly decorated mts, contains pear-shaped dense bodies, and probably 2 lumenal mts; rhoptries absent. Thin-walled cyst (7-8 µm) divides into 4 daughters. Type strain

Spi-2 (Mylnikov, Borok, Russia; ultrastructurally unstudied); type rDNA sequence its KU159286; but morphological description based on a strain (Krylov and Mylnikov 1986, type figures; now lost, unsequenced; see also Mylnikov 1983) isolated from same Borok sewage works (later called S-1: Mylnikov 1991) and 'very similar' by LM (Mylnikov pers. com.). Other species: *Microvorax tetrahymenae* comb. n. Basionym *Colpodella tetrahymenae* Cavalier-Smith in Cavalier-Smith & Chao (2004 p. 194); *Microvorax gonderi* comb. n. Basionym *Spiromonas gonderi* Foissner and Foissner (1984). **Dinomonadidae fam. n. Diagnosis:** myzocytotic predators on ciliates and other heterotrophs with two subequal posteriorly directed cilia longer than cell body with widely separate centrioles set in distinct pits (anterior deep, posterior shallow) about 2 μm behind pointed tip of rostrum. Rhoptries of two types. Prominent oblique mt root to cell's right of kinetid (Brugerolle 2002a Fig. 3). Anterior amorphous ciliary paraxonemal rod present basally. Subpellicular microtubules only in anterior third, mainly dorsal, rostral. Anterior root outside pseudoconoid. Desmose several times longer than centriole width. Type genus *Dinomonas* Saville Kent, 1880-1. *D. vorax* Saville Kent, 1880-1 [syn. *Colpodella vorax* Simpson and Patterson, 1996).

Order 4. Algovorida Cavalier-Smith in Cavalier-Smith and Chao, 2004

Superorder 2. Paraconoidia superord. n. Diagnosis: heterotrophic biciliate predators with small but distinct curved pointed rostrum with numerous evenly spaced subpellicular microtubules attached beneath strongly flattened cortical alveoli; pseudoconoid wall mts absent; bypassing microtubular band with spiral I-fibre-like extension with two attached microtubules at its tip curves round microneme and rhoptry tips and 5-microtubule anterior centriolar root as a 'paraconoid' proximal to preparaconoidal ring; divide into four or eight daughters within cysts; shallow ventral longitudinal groove. **Etymol:** *para* beside, *conoid* E. beyond; indicates that a shadowy relic of the presumably lost pseudoconoid mts is the spiral fibre that in *Eimeria* is within and beside the conoid. Not based on *Colpodella* in case *C. pugnax* does not belong here.

Order Colpodellida Cavalier-Smith, 1993 [*Colpodella* (=*Alphamonas*) *edax, C. pseudoedax, C. pugnax*; heterotrophs, anterior ciliary hairs; continuous compressed cortical alveoli; normal length orthogonal centrioles; dorsal microtubular pellicle].

Subclass 2. Vitrelloidia* subcl. n. Diagnosis: as for sole order Vitrellida:

Order **Vitrellida*** ord. n. **Diagnosis:** Phototrophs dividing within sporangia into numerous daughters. Pseudoconoid or paraconoid absent. Outer cortical alveolar layer continuous (not discrete as in *Chromera*'s single cortical alveolar layer); second inner layer of discrete cortical alveoli. (*Vitrella*)

Parvphylum 2. Sporozoa Leukart, 1879 stat. n. Cavalier-Smith, 2014

Class 1. Paragregarea Cavalier-Smith, 2014 (e.g. *Selenidium*, *Veloxidium*, *Stenophora*)

Class 2. Gregarinomorphea Grassé, 1953 em. Cavalier-Smith, 2014

Subclass 1. Histogregaria Cavalier-Smith, 2014 (e.g. *Rhytidiocystis*)

Subclass 2. Cryptogregaria Cavalier-Smith, 2014 (*Cryptosporidium*)

Subclass 3. Orthogregarinia Cavalier-Smith, 2014 (e.g. *Stylocephalus*, *Monocystis*, *Gregarina*, *Blabericola*, *Porospora*)

Class 3. Coccidiomorphea Doflein, 1901 em. Cavalier-Smith, 2014

Subclass 1. Coccidia Leuckart, 1879 (e.g. *Aggregata*, *Eimeria*)

Subclass 2. Hematozoa Vivier, 1982 (e.g. *Plasmodium*, *Nephromyces*, *Babesia*, *Theileria*)

Phylum 2. Ciliophora Doflein, 1901 (ciliates, suctorians; nuclear dimorphism; no plastids; 12 classes, 1 new)

Subphylum 1. Intramacronucleata Lynn, 1996 (spindle in macronucleus; kinetodesmal fibre)

Infraphylum 1. Spirotrichia Cavalier-Smith, 2004 em.

Class 1. Spirotrichea Bütschli, 1889 (e.g. *Oxytricha*, *Euplotes*, *Strombidium*)

Class 2. Cariacotrichea Orsi et al., 2011 (*Cariacothrix*)

Class 3. Armophorea Lynn, 2004 (anaerobes, e.g. *Metopus, Nyctotherus*)

Class 4. Litostomatea Small and Lynn, 1981 (e.g. *Balantidium*, *Didinium*)

Infraphylum 2. Ventrata Cavalier-Smith, 2004 (ventral mouth)

Class 1. Phyllopharyngea De Puytorac et al., 1974 (4 subclasses incl. Suctoria)

Class 2. Colpodea Small and Lynn, 1981 em., incl. Nassophorea Small and Lynn, 1981 as subclass Nassophoria (e.g. *Colpoda*, *Nassula*, *Obertrumia*)

Class 3. Prostomatea Shewiakoff, 1896 (e.g. *Coleps*)

Class 4. Plagiopylea Small and Lynn, 1985 (e.g. *Plagiopyla*, *Trimyema*)

Class 5. Oligohymenophorea De Puytorac et al., 1984 (e.g. *Paramecium*,

Infraphylum 3. Protocruzia infraphyl n. and new class **Protocruzea** cl. n. **Diagnosis** for both as for subclass Protocruziidia De Puytorac et al., 1987 (Lynn and Small 2002 p. 421). (*Protocruzia*) Deeper branch on multigene trees than preceding infraphyla (Gentekaki et al. 2017).

Subphylum 2. Postciliodesmatophora Gerassimova and Seravin, 1976

Class 1. Karyorelictea Corliss, 1974 (e.g. *Tracheloraphis*, *Loxodes, Geleia*)

Class 2. Heterotrichea Stein, 1859 (e.g. *Stentor*, *Folliculina*, *Blepharisma*)

Infrakingdom 2. Rhizaria Cavalier-Smith, 2002 em. 2003 (reticulose or filose pseudopodia; rare ciliary hairs non-tubular; 18 classes 1 new)

Phylum 1. Cercozoa Cavalier-Smith 1998 em. (cortical alveoli absent; extrusomes mostly globular; 8 classes, 1 new)

Subphylum 1. Reticulofilosa+ Cavalier-Smith, 1997 (3 classes)

Class 1. Chlorarachnea Hibberd and Norris, 1984 orth. em. Cavalier-Smith, 1986

Order 1. Chlorarachnida* Hibberd and Norris, 1984 (e.g. *Bigelowiella*, *Chlorarachnion, Lotharella, Cryptochlora, Gymnochlora, Viridiuvalis*)

Order 2. Minorisida ord. n. **Diagnosis and etymology:** as for **Minorisidae** fam. n. **Diagnosis:** Minute marine phagoheterotrophic picoplanktonic bacterivorous flagellates with single long acronematic smooth cilium. Type genus *Minorisa* Del Campo in Del Campo et al. (2013 p. 355)

Class 2. Granofilosea Cavalier-Smith and Bass in Bass et al., 2009 (e.g. *Mesofila, Clathrulina*, *Massisteria*, *Minimassisteria*, *Reticulamoeba, Leucodictyon*, *Limnofila*, *Tetradimorpha*)

Class 3. Skiomonadea Cavalier-Smith in Cavalier-Smith and Karpov, 2012 (*Tremula*)

Subphylum 2. Monadofilosa Cavalier-Smith, 1997 (5 classes 1 new)

Superclass 1. Eoglissa Cavalier-Smith in Cavalier-Smith and Oates, 2011 em.

Class 1. Metromonadea Cavalier-Smith, 2007 (*Metopion*, *Micrometopion*, *Metromonas, Kiitoksia*)

Class 2. Helkesea cl. n. Diagnosis: apically or subapically biciliate zooflagellates with posterior ciliary gliding and extrusomes, plus related tetraciliate parasites and guttulinopsid lobose amoebae; flagellates either with anterior cilia just a stub without 9+2 axoneme or dorsoventrally flattened thecate biciliates with normal anterior cilium and filose pseudopods emanating from a short posterior ventral slit separate from ciliary apertures that are phylogenetically closer to them than to Ventrifilosa.

Order 1. Helkesida ord. n. Diagnosis: biciliate or tetraciliate zooflagellates with anterior cilium of each kinetid reduced to a stub, plus lobose non-ciliate amoebae phylogenetically closer to them than to Ventrifilosa. Centriolar roots highly simplified, sometimes to as few as three microtubules. Flat mitochondrial cristae, unlike most Rhizaria.

Superfamily 1. Sainouroidea Cavalier-Smith in Cavalier-Smith et al., 2009 em. (*Sainouron*, *Cholamonas*)

Superfamily 2. Helkesimastigoidea superfam. n. Family 1. Helkesimastigidae Cavalier-Smith in Cavalier-Smith et al., 2009 (*Helkesimastix*). Family 2. Guttulinopsidae Olive, 1970 (*Guttulinopsis, Rosculus*)

Order 2. Ventricleftida Cavalier-Smith in Howe et al., 2011 (*Ventrifissura*, *Verrucomonas*)

Superclass 2. Ventrifilosa Cavalier-Smith in Cavalier-Smith and Karpov, 2012

Class 1. Sarcomonadea+ Cavalier-Smith, 1993 stat. n. 1995 (e.g. *Paracercomonas, Kraken, Cavernomonas*, *Cercomonas*, *Allapsa*, *Bodomorpha*, *Neoheteromita, Proleptomonas, Sandona*, *Viridiraptor*, *Agitata*, *Acinetactis*, *Aurigamonas*)

Class 2. Imbricatea Cavalier-Smith in Cavalier-Smith and Chao, 2003

Subclass 1. Placonuda Cavalier-Smith in Cavalier-Smith & Chao, 2012 (e.g. *Clautriavia*, *Nudifila*, *Quadricilia*, *Abollifer*, *Auranticordis*, *Pseudopirsonia*, *Euglypha*, *Paulinella*, *Cyphoderia*, *Corythion*, *Trinema*, *Zoelucasa*)

Subclass 2. Placoperla Cavalier-Smith in Cavalier-Smith and Chao, 2012 (e.g. *Thaumatomonas*, *Reckertia*, *Gyromitus, Ovaloplaca, Thaumatospina, Spongomonas*, *Rhipidodendron*, *Discocelia*; probably also *Acanthoperla*****, *Pompholyxophrys*****)

Class 3. Thecofilosea Cavalier-Smith in Cavalier-Smith and Chao, 2003 em. 2012

Subclass 1. Eothecia Cavalier-Smith in Cavalier-Smith and Chao, 2012 (*Botuliforma*, *Cryothecomonas*, *Ebria, Hermesinum, Mataza*, *Protaspa*, *Rhogostoma, Sacciforma*)

Subclass 2. Phaeodaria Haeckel, 1879 (e.g. *Aulacantha*, *Challengeron*, *Protocystis*)

Subclass 3. Tectosia Cavalier-Smith in Cavalier-Smith and Chao, 2012 (e.g. *Lecythium*, *Lithocolla*, *Pseudodifflugia, Rhizaspis, Trachyrhizium*)

Phylum 2. Retaria Cavalier-Smith, 1999 em. (heterotrophs with reticulopodia; 10 classes, 1 new)

Subphylum 1. Endomyxa Cavalier-Smith, 2002 (4 classes 1 new)

Superclass 1. Marimyxia supercl. n. **Diagnosis:** trophically non-ciliate marine amoeboids without central capsule; free-living reticulose cells or amoeboid entirely non-ciliate parasites of marine invertebrates with complex spores with one or more cells and no polar capsules or filaments. Gametes (*Gromia* only) uniciliate. Phylogenetically includes free-living Gromiidea and their parasitic ascetosporan descendants. **Etymol:** *maris* L. sea; *myxo* Gk mucus.

Class 1. Gromiidea+ Cavalier-Smith in Cavalier-Smith and Chao, 2003 em. (*Gromia*, *Filoreta*)

Class 2. Ascetosporea Sprague, 1979 stat. n. Cavalier-Smith, 2002 (e.g. *Haplosporidium*, *Mikrocytos*, *Minchinia*, *Paradinium*, *Marteilia*, *Paramyxa*, *Paramarteilia*)

Superclass 2. Proteomyxia Lankester, 1885 (class Proteomyxa) ex Cavalier-Smith, 2017 stat. n. **Diagnosis:** Heterotrophic non-ciliate amoeboid free-living reticulose or filose protists (Vampyrellidea), typically mycophagous or algivorous, and amoeboid or plasmodial trophically non-ciliate parasites (of plants or algal chromists) with biciliate dispersal stage (Phytomyxea). **Etymol:** *Proteus* Gk. a sea god able to change shape; *myxo* Gk mucus. Sierra et al. (2016) label this clade Phytorhiza, but did not establish a taxon; I prefer to modify Lankester's historic name than to switch to that or any other unnecessary new name. Proteomyxa the first class to

include *Vampyrella* and *Protomyxa* (Lankester 1885) was refined as class Proteomyxidea by Cavalier-Smith and Chao (2003b) and Bass et al. (2009b); but remained polyphyletic, *Filoreta* now put in Gromiidea, and others in glissomonads (Hess and Melkonian 2013); the holophyletic core residue is now renamed Vampyrellidea for consistency with its sole order Vampyrellida:

Class 1. Vampyrellidea cl. n. Diagnosis as for Vampyrellida in Hess et al. (2012 p. 10) (*Arachnula, Hyalodiscus*, *Leptophrys*, *Penardia*, *Platyreta*, *Theratromyxa*, *Thalassomyxa*, *Vampyrella*, *Vernalophrys*)

Class 2. Phytomyxea Engler and Prantl, 1897 (e.g. *Phagomyxa, Maullinia*, *Plasmodiophora*, *Spongospora*)

Subphylum 2. Ectoreta subphyl. n. **Diagnosis:** ancestrally marine; large-celled, uninucleate or multinucleate, non-ciliate, reticulose trophic phase typically grows manyfold (for weeks or months), then undergoes multiple fission into much smaller cells (binary fission in a few); usually with smaller usually biciliate swimming (not gliding) gametes or zoospores; distinguished from Endomyxa by cells divided by test or capsule into central nuclear region containing mitochondria, Golgi apparatus, and endoplasmic reticulum, and outer ectoplasm of reticulopodia; uniquely use novel 2 tubulins (Hou et al. 2013). **Etymology:** *ectos* Gk outside; *rete* L. net: emphasises their fundamental subdivision into endoplasm and reticulose ectoplasm. (6 classes)

Infraphylum 1. Foraminifera D'Orbigny, 1826 ex Cavalier-Smith 2017 stat. n. **Diagnosis**: microtubules in reticulopodia, irregularly arranged, not nucleated by axoplasts; organic extracellular test with 1-many pores, rarely secondarily absent, 1- or multi-chambered; test usually augmented by aggregated foreign material or calcified; gametes usually biciliate, rarely triciliate or amoeboid.

Class 1. Monothalamea Schultze, 1854 (4 orders including xenophyophores) (e.g. *Reticulomyxa*, *Allogromia*)

Class 2. Globothalamea Pawlowski et al., 2013 (9 orders, e.g. *Ammonia*, *Bulimina*, *Elphidium*, *Textularia*, *Globigerina*, *Globobulimina*, *Nonionella*)

Class 3. Tubothalamea Pawlowski et al., 2013 (e.g. *Ammodiscus*, *Sorites*, *Quinqueloculina*, *Spirillina*)

Infraphylum 2. Radiozoa Cavalier-Smith, 1987 em. 2003 stat. n. **Diagnosis:** cells with radiating axopodia supported by axonemes nucleated by 1-many intracapsular axoplasts; axonemes of cross-linked microtubules ancestrally in open hexagonal array, each hexagon with 6 (Spasmaria) or 12 microtubules (Polycystinea – in some subgroups hexagons incomplete on one side, resembling branching palisades); axopodia thicker and longer than reticulopodia (in *Sticholonche* have basal joints and myonemes that can row cells for active swimming). Mineralised skeleton usually present: largely strontium sulphate (Acantharea) or amorphous silica (Polycystinea, Sticholonchea) with only small amounts of strontium sulphate. Ectoplasm separated from endoplasm by cortical alveoli and dense multiperforated central capsule sandwiched between cortical alveoli and plasma membrane invaginations (Polycystinea) or by extracellular fibrous capsule (Acantharea). Reticulopodia rarely secondarily absent (*Sticholonche*). Zoospores biciliate.

Superclass 1. Polycystinia Ehrenberg, 1838 stat. n.

Class Polycystinea Ehrenberg, 1838 stat. n. Cavalier-Smith, 1993 (e.g. *Collozoum*, *Lampromitra*, *Spongosphaera*)

Superclass 2. Spasmaria Cavalier-Smith, 1993 stat. n.

Class 1. Acantharea Haeckel, 1881 stat. n. Cavalier-Smith, 1993 (e.g. *Acanthocolla*, *Acanthoplegma*, *Astrolonche*, *Conacon*, *Phyllostaurus*)

Class 2. Sticholonchea Poche, 1913 stat. n. Petruschevskaya, 1977 (*Sticholonche*)

Subkingdom 2. Hacrobia Okamoto et al. ex Cavalier-Smith, 2010 (biciliates or axopodial and non-ciliated; plastids inside rough ER: 2 phyla 11 classes)

Phylum 1. Cryptista Cavalier-Smith, 1989 em. 2015 (pellicle lacks cortical alveoli; bipartite tubular hairs ancestrally on one or both cilia; 7 classes)

Subphylum 1. Rollomonadia Cavalier-Smith, 2013

Superclass 1. Cryptomonada Cavalier-Smith, 2004 (as subphylum) stat. n. 2015

Class 1. Cryptophyceae* Fritsch in West and Fritsch, 1927 (e.g. *Guillardia*)

Class 2. Goniomonadea Cavalier-Smith, 1993 (*Goniomonas*; *Hemiarma* Shiratori and Ishida, 2016 type genus of **Hemiarmidae** fam. n. **Diagnosis:** unlike Goniomondidae Hill, 1991 periplast plates polygonal, not square, and cover only right half of cell, and ciliary transition plate is single. Put in **Hemiarmida** ord. n. with same diagnosis)

Superclass 2. Leucocrypta Cavalier-Smith, 2004 (as subphylum stat. n. 2015: kathablepharids)

Class Leucocryptea Cavalier-Smith, 2004 (e.g. *Kathablepharis*, *Roombia*)

Subphylum 2. Palpitia Cavalier-Smith in Cavalier-Smith and Chao, 2012

Class Palpitea Cavalier-Smith in Cavalier-Smith and Chao, 2012 (*Palpitomonas*)

Subphylum 3. Corbihelia Cavalier-Smith in Cavalier-Smith, Chao, Lewis, 2015 (pharyngeal baskets or centrosome-nucleated radiating axopodia)

Superclass 1. Endohelia Cavalier-Smith in Cavalier-Smith, Chao, Lewis, 2015

Class Endohelea Cavalier-Smith in Yabuki et al., 2012 (*Microheliella*, *Heliomorpha*)

Superclass 2. Corbistoma Cavalier-Smith in Cavalier-Smith, Chao, Lewis, 2015

Class 1. Picomonadea Seenivasan et al., 2013 (*Picomonas*)
Class 2. Telonemea Cavalier-Smith, 1993 (*Telonema, Lateronema*)

Phylum 2. Haptista Cavalier-Smith, 2003 stat. n. 2015 (cortical alveoli; diverse surface microtubule skeletons; 4 classes)

Subphylum 1. Haptophytina* Cavalier-Smith in Cavalier-Smith, Chao, Lewis, 2015 (ancestrally photosynthetic biciliates with microtubule-supported haptonema 'fishing rod' containing extensions of cortical alveoli)

Class 1. Coccolithophyceae Casper, 1972 ex Rothmaler, 1951 (=Prymnesiophyceae Hibberd em. Cavalier-Smith, 1996) (e.g. *Emiliania*, *Isochrysis*)
Class 2. Pavlovophyceae Cavalier-Smith, 1986 (e.g. *Diacronema*, *Pavlova*)
Class 3. Rappephyceae Cavalier-Smith in Cavalier-Smith, Chao & Lewis, 2015

Subphylum 2. Heliozoa Haeckel 1866 stat. n. Cavalier-Smith in Cavalier-Smith et al., 2015

Class Centrohelea Kühn, 1926 ex Cavalier-Smith, 1993 (e.g. *Oxnerella*, *Polyplacocystis*, *Acanthocystis*, *Raphidiophrys*) (= unranked Centroheliozoa: Dürrschmidt and Patterson 1987)

---

*Taxa that are certainly ancestrally photosynthetic; ** heterotrophs with leucoplasts.
*** I suggest vernacular eudinea for clade Peridinea plus Sulcodinea. This is compositionally equivalent to superclass Dinokaryota sensu Cavalier-Smith 1993, a sense still used by Gómez et al. (2010); later (Cavalier-Smith 2003) I adopted the broader circumscription for Dinokaryota (including also Noctilucea, as here) by Fensome et al. (1993) who independently invented the same name at subphylum rank; I retain my original superclass rank but circumscribed as by Fensome et al.).
****Sequence data are needed to verify that these two genera really belong here rather than amongst podiate eukaryotes
+Probably paraphyletic

Except in the larger ciliate subphylum, this classification is comprehensive down to subclass rank; lower rank taxa are mentioned only where changes are made significant for the broad evolutionary questions discussed here. Some phycologists split ochrophyte classes Aurophyceae, Phaeophyceae and Diatomeae as delimited here into more than one class each, which I consider undesirable rank inflation.

Supplementary Discussions SD1-SD12

SD1. Chromist algal pigment diversification

Accessory pigments broaden the spectrum that algae can use for photosynthesis, adapting them to different light regimes. Like cyanobacteria, red algae have blue and red phycobiliproteins organised as phycobilisome particles attached to photosystems I and II on the surface of single thylakoids. The first chromists evolved chlorophyll c_2 and lost phycobilisomes, transferring the single remaining phycobiliprotein to the thylakoid lumen; that enabled thylakoid stacking to increase pigment density. Chromists have various carotenoids, some photoprotective, some harvesting light for photosynthesis. Cryptophytes are the only chromists that kept phycobiliprotein and stack thylakoids in pairs; other chromists lost phycobiliprotein and typically stack them in threes, a small modification presumably occurring independently in Haptista and Harosa when their ancestors independently lost the NM (former red algal nucleus kept only by cryptophytes) after its essential genes were transferred to the host nucleus. Algal chromists typically have chlorophyll a/c_2-binding proteins that form light harvesting complexes (LHC); which in cryptophytes at least form higher order structures (Kereïche et al. 2008). Haptophytes and some ochrophytes evolved chlorophyll c_1 also by hydrogenating one c_2 double bond or c_3 also by methoxycarbonylating c_2; each species typically has c_2 plus either c_1 or c_3, not both. Most synurid chrysophytes lost c_2 while retaining c_1, though *Synura sphagnicola* kept c_1 instead (Mizoguchi et al. 2011); that confirms it was unwise to regard c_2 loss as a reason for making them a separate class (Andersen 1987; Cavalier-Smith 1986) - a separate order suffices (Ruggiero et al. 2015). Chromeroids lost c_2 making them the only chromists retaining a red algal chloroplast that lack chlorophyll c; they appear to be strictly coral-associated and not planktonic as

are most chlorophyll c chromists other than brown algal seaweeds that dominate the littoral and sublittoral in higher latitudes.

Chlorophylls c_{1-3} are chiral like amino acids. All chromists use the same (13^2R)-enantiomer, consistently with c_2 having evolved once only in the ancestral chromist (Mizoguchi et al. 2011). Chromists evolved a much greater variety of LHC proteins than Plantae or cyanobacteria, with a bewildering array of paralogues from which it is hard to extract a phylogenetic conclusion beyond major paralogues having diverged early in chromist evolution, some shared by several lineages and some unique to one, e.g. the *Chromera* clade (Pan et al. 2012). Carotenoids are very diverse. In ochrophytes (ancestrally marine) fucoxanthin was the ancestral LHC carotenoid, lost polyphyletically by several freshwater lineages. Haptophytes use fucoxanthin and 19'-hexanoyloxyfucoxanthin. Dinokaryotes other than Karlodinia use peridinin. *Chromera* has three LHC protein types, one of red algal character, one related to fucoxanthin/chlorophyll-c LHCs; and at least two LHC complexes (Tichy et al. 2013), one especially adapted to far red absorption (Bina et al. 2014), but instead of fucoxanthin or peridinin using a unique iso-fucoxanthin-like carotenoid (Llansola-Portoles et al. 2016), consistently with its early divergence from Dinozoa and vertical inheritance of chromeroid plastids.

Overall this great diversity fits an early divergence of five separate photophagotrophic chromist lineages that evolved different pigments and LHCs that differentiated them ecologically from marine red algae that are largely confined to littoral shaded situations, and better fitted chromists to the open ocean photic zone than are most green algae or cyanobacteria. Lineages kept their distinctive antenna pigments and associated proteins conservatively for hundreds of millions of years with no tertiary transfers except the single ecologically and systematically insignificant karlodinian replacement of peridinin plastids by haptophyte 19'-hexanoyloxyfucoxanthin plastids. One reason chromists may be so successful in oligotrophic regions is that unlike most plants many retain phagotrophy and can get nutrients by predation, like insectivorous plants in nutrient-starved bogs. But for chromists phagotrophy is the ancestral state, not a rare derived curiosity. Another reason for their success may be that their photosystem I for unknown reasons traps light twice as fast as in higher plants (Belgio et al. 2017).

SD2. Plant cytoskeletons differ substantially from chromists

I originally argued that the biciliate host that made Plantae had a ciliary root with a mulilayered structure (MLS) as this is present in both Viridiplantae and the glaucophytes *Glaucocystis* and *Gloeochaete* (Cavalier-Smith 1982). O'Kelly (1993) the co-describer of *Malawimonas* (O'Kelly and Nerad 1999) postulated that the green plant MLS (found in both major clades: widely in streptophytes; in chlorophytes only in order Halosphaerales of Pyramimonadophyceae) was homologous with excavate left posterior R1 and possibly also left posterior R1 MLS found in very few dinoflagellates (Wilcox 1989). Comparative ultrastructure has shown that both suggestions are correct and that plant and dinoflagellate MLS are indeed homologues of excavate R1 plus its **dorsal** fibrillar C fibre, which probably first evolved even earlier than excavates in the last common ancestor of orthokaryotes and discicristates: Fig. 2). By contrast the so-called 'MLS' found by Moestrup (1978) in the euglenoid *Eutreptiella* is more likely homologous with a structurally distinct 'multilayered' structure comprising

the arguably phylogenetically older R2 plus its associated **ventral** I fibre, as is seen by its association with striated fibres in the equivalent structure in *Percolomonas* (in Percolozoa the sister phylum of Euglenozoa: Fig. 2) - see Cavalier-Smith (2017).

R1- and R2-associated multilayers were also confused in Glaucophyta; neither Kies (1976, 1979, 1989) who characterised them nor I (Cavalier-Smith 1982, 1987d, 2013b) realised there are two different kinds and Moestrup (2000) failed to establish root homologies. I now argue for the first time that *Glaucocystis* with four 'MLS' not only has two root R1-like true MLSs homologous with those of green plants (Kies and Kremer 1990 Fig. 17 MLS1) but also two non-homologous ones ultrastructurally more like R2 with I fibres (Kies and Kremer 1990 Fig. 17 MLS2), as does *Gloeochaete* (Kies 1976: Fig. 48 GW1 and Fig. 50 in R2 with I fibre and A fibre, GW2 is R1). By contrast *Cyanophora paradoxa* has only one 7-mt R2 with I fibre and A fibre which is in the canonical postion on the right side of its conspicuous posterior groove (Mignot et al. 1969); its left posterior root (R1) of 9 mts is **not** a classical MLS and probably develops from its 9 mt right anterior root when the anterior cilium becomes posterior (Heimann et al. 1989), and the 3 mt anterior root must add extra mts and A/I fibres to become R2. *Cyanoptyche* (Kies 1989) has two non-classical 'MLS': a large one (~30 mts) that is ultrastructurally R2 with A/I fibres and numerous mts (Kies 1989 Fig 16) and a small one with 5 mts (erronously stated to be 30) and a more rudimentary I fibre (Fig. 15); Kies half realised there was a problem with his erroneous asumption of homology with green plant MLS by writing that compared with other MLS it is 'Astonishingly ....oriented inside out', but could not have understood why until the I fibre was recognised as a pervasive ancient structure (Simpson 2003). R1 has a slender striated fibre as does R1 of chlorophyte green algae Halosphaerales (O'Kelly 1992) and *Nephroselmis* (Suda 2003) (Nephrophyceae: Cavalier-Smith 1993b), representing the deepest and second deepest branches respectively of flagellate chlorophytes on 7-gene chloroplast trees (Leliaert et al. 2016), both classified in subphylum Prasinophytina (Ruggiero et al. 2015). These R1-associated striated fibres (SF) have the SF-assemblin periodicity; in the heterokont *Phytophthora* assemblin is present instead alongside anterior root R3 (Harper et al. 2009), which I argue in a later section of this paper is the general condition in chromists, unlike plants where the *Nephroselmis*/Halosphaerales/*Cyanoptyche* R1 location is arguably the ancestral assemblin state. Clearly the C-fibre multiple layers on R1 were lost by *Cyanophora*/*Cyanoptyche*, a deep-branching sister clade to *Glaucocystis*/*Gloeochaete* by mitochondrial DNA (Jackson and Reyes-Prieto 2014); because of this deep ultrastructural divergence I separate *Cyanophora* and *Cyanoptyche* from Glaucocystales (*Gloeochaete*/*Glaucocystis*) as new order Cyanophorales (**Diagnosis:** glaucophytes with well developed posterior right root I fibre but posterior left root with no dorsal multilayered structure) and new family Cyanophoraceae (same diagnosis: type genus *Cyanophora* Korshikov, 1924).

Green plants ancestrally lost the I fibre; *Nephroselmis* with two anisokont cilia like *Cyanophora* and the inferred ancestral corticate and just three roots best represents the ancestral green plant condition - its R2 has a ventral band of three mts plus a dorsal singlet in line with the middle one and attached to the end one by an oblique slender lamina (Suda 2003). Most chlorophytes have the same 'one over three' 3+1 arrangement but in some the ventral band may increase to four or five mts, rarely more (Moestrup 1978). As the primitive streptophyte *Mesostigma* has 3+1 (Melkonian 1989), that is almost certainly ancestral for green plants. I argue that the dorsal singlet of the 3+1 array is the homologue of the posterior singlet which is identically connected to

R2 in *Tsukubamonas* which does not have R1 (and similarly in jakobids). Thus the singlet is not a third posterior root as widely assumed (Simpson 2003), nor diagnostic for excavates, but an integral part of R2 that I suggest evolved before the excavate groove and persisted long after excavates gave rise to corticates and Sulcozoa - it is an ancestral eukaryote character that is remarkably persistent.

All green plants except Nephrophyceae have evolved an R4 by heterochrony to develop the mature R2 structure in C2 as well as C1, creating a cruciate pattern, presumably thrice independently (subphylum Chlorophytina, Pyramimonadophyceae, *Mesostigma*), an easy change once I-fibre-associated complexities were lost. In Halosphaerales roots R3/R1 retained root transformation, mature R1 remaining more complex through retention of phagotrophy, but in Pyramimonadales, *Mesostigma*, and Chlorophytina R1 lost visible ultrastructural transformation so anterior and posterior roots look alike (despite differing molecularly). Most streptophytes lost R2 or reduced it to one mt, but R1 became a broad MLS to make the sperm skeleton, lost by angiosperms and most gymnosperms. Note that in green plants, before their root homology with excavates was recognised, an arbitrary convention was adopted where those homologous with excavate left roots were called dextral (d) and those that were ancestrally right were called sinistral (s), a nomenclatural contradiction which must constantly be kept in mind when reading green plant papers to avoid confusion.

Plant cytoskeletons are relatively more conservative than chromists because most evolved cell walls and lost vegetative centrioles and so used cellulose walls not mt roots as their major skeleton; only scaly Prasinophyceae and alveolated *Cyanophora* remained wall-free and thus cytoskeletally more chromist-like. In chromists walls became morphogenetically dominant only in superclass Fucistia (brown algae and relatives), Eustigmatophyceae, and Pseudofungi. Diatoms evolved siliceous frustules as their major skeleton and lost all centriolar roots (and even cilia in pennates). All other chromists made even more extensive and diverse use of the mt skeleton than any other kingdom. Major reasons for that are their feeding versatility (almost every major change in body plan is linked to novel feeding modes) and the huge morphogenetic potential of their unique BB.

Note added in proof: Since I received proofs of this paper, Aaron Heiss told me he independently discovered the glaucophyte root misinterpretations by Kies, and kindly gave me a preprint on *Cyanophora cuspidata* centriolar roots (Heiss et al. in press) - a more thorough study than any previously. His conclusions are essentially like mine, but he found many hitherto unknown ultrastructural details, which strengthen my thesis that numerous loukozoan-like excavate ciliary root characters were inherited by the last common ancestor of Plantae. In particular he found a singlet 'X mt' adhering to the dorsal face of posterior 6-8 mt root R2 which I regard as homologous with excavate singlet roots and with the dorsal singlet of the viridiplant 3+1 root structure discussed above. This strongly supports my arguments above that (1) the posterior singlet is fundamentally an extra component of all R2s from *Tsukubamonas* to Plantae and (2) that it was inherited by the last common ancestor of Plantae as I previously argued to be the case for Chromista (Cavalier-Smith 2013b). It seems likely that the 9 mt wide AR of *C. cuspidata* is the R3 precursor of split (9 outer + 2 inner mt) left R1 and the narrow 3 mt AR is the R4 precursor of unsplit posterior R2; if correct, *Cyanophora* anterior and posterior centrioles are mutually rotated by 180°, as in Viridiplantae, implying this was the ancestral condition for Plantae, another contrast with the predominantly 90° mutual rotation in chromists. The 'fan' mts associated with *C. cuspidata* wide AR (putative R3) appear to be homologues of the dorsal secondary mts often nucleated along R3 in

heterokont and cercozoan chromists, rather than of the dorsal fan of excavates that is more directly associated with the anterior centriole and present also in excavates lacking R3. Note that Heiss et al. use MLS in a new broader looser sense that does not imply homology.

SD3. Ciliary and cytoskeletal diversification in myzozoan alveolates

Subphylum Myzozoa evolved from an ancestor similar to colponemids (differing primarily in retaining plastids and BB) by adopting a new predatory way of feeding using a sucking anterior rostrum (Apicomplexa) or peduncle (peridinean dinoflagellates) or simpler structure (Myzodinea, Perkinsozoa) that can extract the cytoplasmic contacts of prey into a food vacuole without phagocytically engulfing the whole cell. Schnepf and Deichgräber (1984) called this novel mode of feeding myzocytosis, which I partially incorporated into the name Myzozoa. Myzocytosis made groove feeding by posterior ciliary currents less important so Myzozoa lost the ciliary vane. Most simplified the groove cytoskeleton by losing the central 1-mt posterior root, but this singlet (previously overlooked) remains in *Colpovora unguis* (Mylnikov 2009 Fig. 4f) a myzocytotic flagellate wrongly identifed as a *Colpodella* (here transferred to new genus *Colpovora* now grouped with *Psammosa* with identical ciliary transition region and related rDNA in a new ancestrally myzocytotic dinoflagellate class Myzodinea, not in Apicomplexa: Table 1). Myzocytosis entailed modifying the cytoskeleton to roeorient the anterior cilium to point away from the prey being sucked dry, laterally in Myzodinea and other dinoflagellates and typically backwards in Apicomplexa, in contrast to its forward-pointing in excavates and Colponemea, and evolving a distinctive subapical or apical region containing novel extrusomes (micronemes, rhoptries) that mediates myzocytosis, as well as uniquely myzozoan bipartite trichocysts (Cavalier-Smith and Chao 2004), supported by diverse arrays of BB mts on the cell's right of the centriole pair (Okamoto and Keeling 2014b).

Ancestral Myzozoa were mixotrophs retaining chloroplasts, later lost independently in some lineages of early diverging infraphyla Apicomplexa (Sporozoa and Apicomonadea) and Dinozoa (planktonic dinoflagellates plus related parasites like Ellobiopsida and Perkinsozoa). Apicomonads are free living biciliates that include myzocytotic *Colpodella*-like heterotrophs and *Chromera*-like phototrophs (at least two distinct clades of each, proving multiple losses of photosynthesis) and an asymmetric apical complex somewhat similar to that of Dinozoa with peduncles. Here I call apicomonad algae chromeroids (not a taxon or clade but an important paraphyletic organisational grade) to embrace both existing order Chromerida, now restricted to *Chromera* which is related to heterotrophic myzomonads (Table 1), and new order Vitrellida (*Vitrella*) which is not (Cavalier-Smith 2014a; Janouškovec et al. 2015; Mikhailov et al. 2015) and therefore now placed in separate apicomonad subclass Vitrelloidia (Table 1). It is conceivable that the mystery 3-4 membrane vesicle near the centrioles Füssy et al. (2016 Fig. 1c) is an apicoplast derivative and *Vitrella* underwent chloroplast replacement from a heterokont, but sequence trees suggesting this (Ševčiková et al. 2015) may be distorted by long-branch problems and are strongly contradicted by 13-protein chloroplast trees that show myzozoan plastids (dinoflagellates, *Vitrella*) as sisters of heterokonts (consistent with vertical inheritance) (Dorrell et al. 2017) not branching within them as replacement by lateral transfer would predict. Note that neologism chrompodellid (Janouškovec et al. 2015) is an entirely unnecessary junior synonym of apicomonad, which currently embraces chromerids, *Colpodella* and other related heterotrophs (Ruggiero et al. 2015); apicomonad is perfectly

appropriate for them all. rDNA trees (Cavalier-Smith 2014a; Park and Simpson 2015) show that at least two apicomonad lineages lost photosynthesis independently of Sporozoa that became obligate parasites but uniquely evolved a symmetrical conoid when cilia were suppressed in infective cells. For general sporozoan phylogeny and classification see Cavalier-Smith (2014a); when writing that paper I discovered novel homologies within Myzozoa that greatly illuminate their diversification and that of Halvaria. I explain these in full in separate papers on dinoflagellate and apicomplexan evolution (Cavalier-Smith in preparation); here there is only space for a brief summary after first reducing the confusion still surrounding apicomonad and '*Colpodella*' nomenclature as sparse ultrastructural and sequence data hampered our first attempt at reform (Cavalier-Smith and Chao 2004).

SD4. Apicomonad diversity and evolution: clarifying confusions

My earlier view that *Colpodella* suffered from excessive lumping (Cavalier-Smith and Chao 2004) and sensu Simpson and Pattterson (1996) was polyphyletic is vindicated by an 85-protein tree showing that '*Colpodella angusta*' is sister to *Voromonas* (once *Colpodella*) *pontica* and both closer to *Chromera* rather than '*Alphamonas*/*Colpodella edax*' (Janouškovec et al. 2015) and the demonstration by *Psammosa* that some Dinozoa are superficially apicomonad-like myzocytotic predators (Okamoto et al. 2012) and by new ultrastructure for several supposed *Colpodella*.

'*C. angusta'* had a confusingly chequered nomenclatural history: biciliate *Heteromita angusta* (Dujardin 1841), was transferred to *Spiromonas* (*angustata* sic) by Saville Kent (1880-2), who rightly in my view did not consider it a *Colpodella*, but *Spiromonas* Perty, 1852 is inappropriate for a flagellate (Patterson and Zölffel 1991). Therefore, Patterson and Zölfell (1991), who considered *H. angusta* a bodonid (not a *Colpodella*), made new genus *Dingensia* for it, possibly unnecessary as *Heteromita* might be its valid name (Howe et al. 2009). Contradictorily, Simpson and Patterson (1996) moved *H. angusta* to *Colpodella*, I think mistakenly: the earlier judgement that it was a *Cryptaulax*-like bodonid better fits its morphology (see Howe et al. 2009 illustration and comments). By then '*Spiromonas angusta*' had been used for an ultrastructurally studied apicomonad (Krylov and Mylnikov 1986; Mylnikov 1991) which morphologically was neither *S. angustata* nor *H. angusta*! Based on light microsopy (Mylnikov 1983), Simpson and Patterson (1996) conjectured that Mylnikov's '*Spiromonas angusta'* strain S-1 was the same as '*Colpodella*' (=*Dinomonas*) *vorax* (Saville Kent) Simpson and Patterson, 1996. I disagee, as the non-feeding form is narrower (3-4 μm) and a different shape from *D. vorax* (straight not curved, parallel-sided not tapering/pyriform; not pointed anteriorly); especially given the marked difference in apical form, I consider it a new genus, *Microvorax* (Table 1). Brugerolle (2002a) ultrastructurally studied a '*C. vorax*' and reasonably thought it the same as *Dinomonas vorax* (Saville Kent 1880-2 who sensibly did not consider it a *Colpodella*). Mylnikov and Mylnikova (2008) renamed the Russian *S. angusta*(*ta*) '*Colpodella angusta* Simpson and Patterson, 1996', contrary to the latter's assumption that it was *D. vorax*. Ultrastructurally studied *S. angusta* S-1 (Krylov and Mylnikov 1986; Mylnikov 1991) has short centriolar connectors and is certainly not the same species or genus as *C. vorax* of Brugerolle (2002a) which had centrioles far apart and a pointed rostrum about twice as long as the rounded one of *S. angusta* S-1. Moreover the pseudoconoid of Mylnikov's is much smaller than Brugerolle's and their cells are different shapes and sizes; both cannot be *D.*

vorax. Though smaller than *Dinomonas vorax* (~16 µm), for continuity with previous ultrastructural papers I accept Brugerolle's 12 µm strain as *Dinomonas vorax,* which it well resembles in shape and equal cilia, though they are more subapical than Saville Kent depicted. I, as first reviser of *Dinomonas*, here designate *D. vorax* the type species (described earlier in Saville Kent's book than *D. tuberculata*, which I exclude from *Dinomonas* as it clearly is not a myzocytotic apicomonad but phagocytic, probably unidentifiable paracercomonad-like cercozoans and thus useless as a type for the genus). *Chilovora perforans* (Brugerolle and Mignot 1979) is a similar shape to *D. vorax* but smaller (7-9 µm) so not the same species; it differs so radically ultrastructurally from *Dinomonas* and *Microvorax* that I consider them ordinally distinct. Table 1 establishes a new genus and species for Mylnikov's strain S-1 and later sequenced Spi-2 strains: *Microvorax angusta*. I use *angusta* for continuity with Russian studies, but stress it is **not** '*Heteromita*/*Dingensia*/*Colpodella angusta*', which may be a bodonid but is not a *Colpodella* or other apicomonad. Though substantially different, *Dinomonas* and *Microvorax* are definitively related by exceptionally short chamfered centrioles unlike those of most apicomonads and morphologically unique lamellate centriolar connector, so are put in the new order Voracida (Table 1). Ribosomal DNA trees show *C. tetrahymenae* to be closely related to *Microvorax angusta,* both grouping distantly with *Voromonas* not *Colpodella pseudoedax* (Cavalier-Smith 2014a; Mikhailov et al. 2015: wrongly called *edax* - see next two paragraphs), so *C. tetrahymenae* and similar *C. gonderi* that also feeds on ciliates are transferred to *Microvorax*. Consistently with this revised classification and the ultrastructural distinctions elucidated here, a 2-gene rDNA tree shows *Microvorax angusta* and *M. tetrahymenae* as sisters within a large environmental DNA clade (putatively Voracida) that is deeply separated from but sister to a Voromonadida clade (Mikhailov et al. 2015), both sister to the Chromerida clade; all three group with revised Colpodellida to the exclusion of a more distant environmental clade and Vitrellida is most divergent of all.

Nomenclature of '*Alphamonas*/*Colpodella edax*' has been almost as confused. As *Alphamonas* (Aléxéieff 1918) was established later, *D. vorax* cannot [contrary to Patterson and Zölffel (1991)] be rejected as a junior synonym of *A. edax* (Alexeieff 1924), so the original name *Dinomonas* must be retained. Nor is *Dinomonas* a *Colpodella* (contrary to Simpson and Patterson 1996). Of nominate *Colpodella* species studied by both sequencing and ultrastructure, only *Colpodella pseudoedax* Mylnikov and Mylnikov (2007) is light microscopically similar enough to *C. pugnax* the *Colpodella* type species (Cienkowski 1865) to be the same genus, so is the best reference species for true *Colpodella* until genuine *C. pugnax* is restudied. *C. edax* should not have been placed in *Alphamonas* (Cavalier-Smith and Chao 2004); the strain on which that decision was based was studied by scanning EM and its rDNA sequenced as *C. edax* (Leander et al. 2003), but was actually clone BE-2 isolated in 2002 at Borok, Russia (Mylnikov pers. comm.), i.e. the type strain of *C. pseudoedax* (Mylnikov and Mylnikov 2007), thus **not** *C. edax*. By about 2000 Mylnikov (pers. comm.) had lost *Bodo* (=*Colpodella*=*Alphamonas*) *edax* strain BE he studied earlier (Mylnikov 1988; Mylnikov et al. 1998), which was therefore never sequenced.

Thus Leander et al. (2003) and Janouškovec et al. (2015) both actually sequenced BE-2, the *pseudoedax* type strain, not *C. edax* as the papers and GenBank stated. *C. pseudoedax* BE-2 (Mylnikov and Mylnikov 2007; Mylnikova and Mylnikov 2009) is smaller (7-10 X 3-5 µm) than *pugnax* (12 µm), but his *edax* (10-16 µm) overlaps with it; both are less markedly semilunate than *pugnax* and unlike it eat heterotrophic flagellates not

Chlamydomonas. As *C. edax* and *pseudoedax* centriolar roots have different mt numbers and they divide differently, I agree they are separate species, both probably *Colpodella*. If they are *Colpodella*, ultrastructurally none of radically different *Voromonas*, *Chilovora*, *Algovora*, *D. vorax*, or *C. unguis* can be *Colpodella* yet were all once lumped in that genus. My present system (Tables 1 and S1) has only three *Colpodella* species: *pugnax* (contrary to Simpson and Patterson (1996) never studied ultrastructurally), *edax* and ultrastructurally similar *pseudoedax*, but they must be grossly undersampled - 69 different rDNA sequences were found in one hypersaline lake, most differing enough to be separate species (Heidelberg et al. 2013). For clarity I confirm that the marine strain we studied as *Voromonas pontica* (Cavalier-Smith and Chao 2004), the generally accepted name, was G-3 also sequenced by Kuvardina et al. (2002) and studied by Mylnikov et al. (2000) as *Colpodella* sp. (later *C. pontica*: Mylnikov 2000); earlier (Mylnikov 1991) as *D. vorax* J-3). It is neither *Colpodella* nor *Dinomonas*.

Returning to apicomonad phylogeny after that essential nomenclatural digression, 2-gene rDNA (Mikhailov et al. 2015) and 85-protein trees (Janouškovec et al. 2015) congruently show that neither *Voromonas* nor *Microvorax* are sisters of *Colpodella pseudoedax,* confirming that these three should not have been lumped in one genus; Voracida and Voromonadida are distantly related sisters, grouped with *Chromera*. Tables 1 and S1 place this 3-order clade in new apicomonad superorder Chromovoridia, together provisionally with morphologically radically distinct *Algovora* for which sequences are unknown. These multigene trees show *Colpodella pseudoedax* as sister to all three, all four being sister to *Vitrella*. Unlike chromovorids which all have pseudoconoids, *Vitrella* (Füssy et al. 2016) and *Colpodella* lack them and branch successively deeper on the multigene trees (Janouškovec et al. 2015; Mikhailov et al. 2015). 'Conoids' of *C. edax* (Mylnikov et al. 1998) and *pseudoedax* (Mylnikov and Mylnikov 2007; Mylnikova and Mylnikov 2009) were misnamed and misinterpreted; each is not conical and only partially microtubular, apparently comprising some mts plus a curved non-microtubular I-fibre-like extension of a mt root. I call this unusual colpodellid structure a paraconoid and segregate *Colpodella* as new subclass Paraconoidia to emphasise this important difference from their sister chromovorids with which they are grouped as subclass Myzomonadia containing all myzocytotic apicomonads, and a robust clade. Apicomonads now have six orders (two algal, four heterotrophic) all ultrastructurally distinct and five now placed on a robust multigene tree concordant with ultrastructure as here disentangled. The major outstanding problem is *Algovora*; the very long centrioles of *A. pugnax* exclude it from Voracida, and its radically different cell shape and ciliary pattern from all genuine apicomonads (similar to *A. turpis*) show it is certainly not *C. pugnax* (as Simpson and Patterson (1996) had assumed) and suggest that *Algovora* may not be apicomonads; but evidence is too scanty to assign them to Myzodinea (plausible) which they resemble more than Colponemea where Cavalier-Smith and Chao (2004) erroneously put *Algovora*. *Acrocoelus* is a perkinsozoan parasite not an apicomonad, so all apicomonads are free-living and all except perhaps *Vitrella* myzocytotic feeders (even *Chromera*).

SD5. Pseudoconoid and conoid evolution: cytoskeletal adaptation in the origin of Sporozoa

Here I summarise evolutionary conclusions for Apicomplexa. Brugerolle (2002a,b) coined the word pseudoconoid for BB mt ribbons of *Dinomonas vorax* and perkinseans *Rastrimonas* and *Parvilucifera*, which are

gently curved in cross section - **not** cone-like, but called the open-cone structure of *Perkinsus* an 'incomplete conoid'. I think it confusing to call any dinozoan mt arrays conoids or pseudoconoids - only Apicomplexa have them. I therefore restrict 'pseudoconoid' to the open-cone mt structures of chromovoridian apicomonads, all closed at the apex (unlike *Perkinsus*) and open only at the side and base because of the presence of the anterior ciliary root (probably R3). No dinozoan structures are closed at the apex like pseudoconoids or the sporozoan conoid, so should not be called pseudoconoids, simply BBs. I consider the open-cone chromovorid pseudoconoid homologous with the sporozoan conoid; both could easily have evolved from a common ancestral structure when Sporozoa vegetatively lost cilia and R3; R3 loss allowed the partially open cone to be completed to its base. On this view, *Vitrella* lost the pseudoconoid; Paraconoidia partially lost the pseudoconoid, keeping the capacity for myzocytosis; Aconoidia (piroplasms, Nephromycida) independently lost conoids.

The non-microtubular conoid of *Toxoplasma* (Hu et al. 2002) is derived, its novel gutter-like tubulin assemblies probably restricted to family Sarcocystidae. Gregarine diversification including ciliary and plastid loss are discussed elsewhere (Cavalier-Smith 2014a). *Colpodella pseudoedax* appears to have a split R2 (Mylnikova and Mylnikov 2009 fig. i) and is the only alveolate where I plausibly found that. If correct, one cannot simultaneously maintain that the pseudoconoid/paraconoid evolved from $R2_o$ root, which apparently has a distinct nucleating centre from $R2_i$ in neokaryotes, and that conoids and pseudoconoids are homologues. It is preferable to suppose that pseudoconoid/conoids evolved from BB, but if the oblique root of *D. vorax* which has a pseudoconoid were a BB (which its anterior extension into the rostrum might suggest) that would raise a problem for direct transformation of BB to pseudoconoid/conoid. That contradiction is simply resolved if the *D. vorax* oblique root (Brugerolle 2002a) is not BB (Okamoto and Keeling 2014b) but an anteriorly moved detached $R2_o$, and its pseudoconoid is a modified BB; that avoids unparsimoniously postulating a novel nucleating centre duplication. *C. pseudoedax* $R2_o$ could be continous with the spiral end of the paraconoid.

Conoids have two intralumenal mts, and between them and the conoid mt wall runs a spiral fibre resembling the paraconoid spiral fibre extension (putatively both derived from $R2_o$). *Voromonas* also has 2 lumenal mts, easily seen in longitudinal section (Cavalier-Smith and Chao 2004) often overlooked in cross-section. I suggest these are homologous with the conoid intralumenal pair on the one hand and the whole paraconoid of *Colpodella* on the other. No separate pair is visible in *Colpodella* or Voracida, consistently with my suggestion that the paraconoid and *vorax* oblique root are both $R2_o$ whereas pseudoconoid/conoids evolved from BB. Though the intralumenal mt pair is short in some Sporozoa, in others it curves a long distance into the cell. It is much more likely to be related to R2 than to the ciliary centre pair (CP) (de Leon et al. 2013); my first proposal of a conoid/root relationship erroneously used the old R1 name for the right posterior root (Cavalier-Smith 2014a).

In *Eimeria* conoids a striated fibrous root curves around between the conoid wall and subpellicular mts and linking them (Dubremetz 1975), which is probably related to the SF-assemblin root that links conoid and nucleus associated centrioles in *Toxoplasma*. As the heterokont *Phytophthora* has a striated assemblin fibre on anterior root R3 (Harper et al. 2009) and apicomonads have only one anterior mt root (R3: the miozoan ancestral state seen in Colponemea) the conoid/centriolar SF-assemblin is almost certainly a relic of R3. If so this disproves the idea that the conoid became closed by incorporating the apicomonad anterior root mts (Portman

and Šlapeta 2014), as its fibrous relic is still outside the conoid wall. That idea is also mechanistically implausible if conoid mts are nucleated apically as I suspect; centriolar roots probably nucleate at the centriolar end and thus would be antiparallel to conoid mts. Evolutionarily hypothetical R3 mt incorporation is probably the very reverse of the key evolutionary forces operating: as apicomonad pseudoconoids are already apically annular, they did not need to incorporate a mt root to close - R3 mt presence in apicomonads is probably precisely what **stopped** them closing; when R3 mts were lost when cilia were vegetatively suppressed the pseudoconoid could have easily closed basally without adding anything; keeping just the assemblin component of R3 allowed tighter conoid-associated coiling and retained centriolar connection without impeding closure.

The presence of a protein SAS-6L in the preconoid region of *Toxoplasma* and *Plasmodium* (de Leon et al. 2013), related to the globular domain of the SAS-6 hub-spoke protein whose self assembly of nine dimers determines the 9-fold symmetry of centrioles (Cavalier-Smith 1974; Guichard et al. 2012; Guichard and Gonczy 2016; Hilbert et al. 2016; Hirono 2014), is also not evidence that conoids evolved from centrioles/cilia. It does however show that they recruited at least one ciliary component: in *Trypanosoma* SAS-6L is present at the ciliary tz; and likely to be present in the distal tz plate of all ciliated eukaryotes that have one except opisthokonts and diatoms where SAS-6L is absent and thus was lost (de Leon et al. 2013). Preconoidal SAS-6L is annular (Wall et al. 2016) so it may be a component of one or both preconoid rings and have been crucial for their origin. It is important to establish whether SAS-6L is present in both apicomonad pseudoconoids and cilia, where *Voromonas* at least has preconoidal rings (Cavalier-Smith and Chao 2004); if it is, that would show preconoidal and tz SAS-6L can coexist in the same cell.

On this interpretation conoids/pseudoconoids evolved from BB by making its apical nucleation centre annular; their mt wall is effectively sandwiched between relics of R2 and R3, so they incorporated three different ancestral chromist cytoskeletal components, whereas preconoidal rings incorporated SAS-6L from the ciliary tz. Clearly, to clarify the origins of conoids, pseudoconoids, and paraconoids we need high resolution unambigous 3D reconstructions of the root/pseudoconoid complex at least as thorough as those done for metamonad excavates and Sulcozoa by Simpson's laboratory (e.g. Heiss et al. 2013a,b; Yubuki et al. 2013) and preferably using also high resolution EM tomography as for trypanosomes (Lacomble et al. 2009), especially for sequenced heterotrophic strains across the tree (technically hard; co-maintaining food and prey in cultures is tricky). Present data are far are too scrappy and often misinterpreted in a mindset wrongly expecting all former '*Colpodella*' to be the same and with insufficient appreciation of root organisation in Dinozoa and Colponemea, the closest relatives of Apicomplexa.

SD6. Dinoflagellate cytoskeletal, plastid, and nuclear diversification

Within dinoflagellates, contrary to Okamoto and Keeling (2014a), the main posterior root of *Psammosa* labelled R1 is not homologous with the *Oxyrrhis*/Peridinea main left root (R1) but is R2 (its short root labelled R2 is really R1). There are many unsolved problems related to subpellicular mt arrays in Dinozoa to be discussed more fully elsewhere, which the present revised classification of Dinokaryota will facilitate. Tables 1 and S1 treat dinoflagellates that replaced their peridinin-containing plastids by haptophyte ones with double envelopes only as new peridinean subclass Karlodinia, as this happpened relatively early in peridinean evolution and they

are cytoskeletally distinctive; '*Karenia*' was preoccupied and invalid in zoological nomenclature so was not used in naming the subclass. The other peridinean subclass Dinophycidae embraces those with typical chloroplasts with triple envelopes and typical peridinean cortical morphology; the segregated dinokaryote class Sulcodinea shares triple-envelope peridinin plastids but has cortical morphology very different from typical Peridinea and arguably early diverging. Ciliary root structure of *Noctiluca* (Höhfeld and Melkonian 1995) and *Amphidinium* (Roberts et al. 1988) both of whose ventral ridge mts are BB, as well as 73-protein ribosomal trees (Bachvaroff et al. 2014) and 101-protein trees (Janouškovec et al. 2017) support retaining the third dinokaryote class Noctilucea, showing earlier divergence of first *Noctiluca*, then *Amphidinium* (Sulcodinea) compared with Peridinea sensu stricto.

These trees robustly show earlier divergence of *Oxyrrhis* than Syndinea, earlier divergence of Karlodinia than Dinophycidae, and holophyly and distinctiveness of successively broader Peridinoidia (thecate dinoflagellates), Dinophycidae (those typically with transverse cingulum and peridinin plastids), Peridinea (those with histone-like proten HLPI), Dinokaryota (liquid crystalline nuclear DNA organisation), Dinoflagellata (Phycodnavirus-like basic chromatin proteins), and Dinozoa [BB planar or typically gently curved in cross section; if strongly curved (*Perkinsus* **only**) not closed apically as a ring as in Apicomplexa], and holophyly of Apicomplexa, Sporozoa, Coccidiomorphea, Coccidia, and Hematozoa as here delimited, and that Perkinsozoa are dinozoan sisters of Dinoflagellata, not Apicomplexa. Therefore these distinct names should be applied precisely and never used loosely as synonyms as often done. Dinokaryotes all have relatively long centrioles compared with excavates but those of eudinea (a clade name suggested here for Peridinea plus Sulcodinea) elongated greatly after they diverged from Noctilucea and evolved a prominent cingulum; at this stage eudinea evolved striated pericentriolar annuli (absent in earlier dinoflagellates) to better anchor their giant centrioles.

Phylogenetically the new syndinan class Endodinea is distinct from Syndinea on rDNA trees (Cavalier-Smith 2014a), but relationships of the three syndinan classes remain ill-resolved as two are not on multigene trees, so we do not know whether their parasitism evolved once or more often; *Psammosa* unfortunately also not yet with multiprotein data, now grouped with *Colpovora* as class Myzodinea with distinctive ciliary tz (Table 1), and *Oxyrrhis* both make it clear that earliest dinoflagellates (superclass Eodina) were neither parasitic nor Peridinea-like in ciliary/root organisation.

However, dinoflagellates at least as early as the common ancestor of *Oxyrrhis* and Peridinea, but after they diverged from Perkinsozoa, evolved virus-related DNA-binding proteins that enabled nucleosome loss from bulk chromatin and paved the way for histone-depleted dinokaryote chromosomes (Gornik et al. 2012). Lateral gene transfer was involved here but it is not obvious that transfer direction was from virus to dinoflagellate as Gornik et al. supposed not the reverse, which I favour. Cell biologically the important thing is that dinoflagellates all retain normal histones as well, presumably primarily for protein-coding gene promoter regions (Marinov and Lynch 2015), a possibility entertained long ago (Cavalier-Smith 1993c).

SD7. Ciliate kinetids also reflect an excavate origin

All Myzozoa except *Noctiluca* are haploid, even the giant gregarines. Ciliophora (ancestral ciliates and derived

suctorians), sisters of Miozoa, by contrast became diploid in the germline but uniquely in eukaryotes evolved somatic macronuclei with macroploidy (manyfold multiplication of **most** but **not** all the genome, conceptually different from polyploidy: Cavalier-Smith 1985, 2004a). Macroploidy's key advantage is it multiplies transcribed genes per cell enabling cells to become giant yet still grow fast because gene copy number for mRNA synthesis ceases to limit cell growth rates. Giant cells with haploid or diploid nuclei invariably grow slowly because their low gene copy number sets an upper limit to mRNA synthesis rates, making cell cycles longer and longer, as larger cells must make more mRNA every cell cycle (and rRNA; but in haploids or diploids achieved by duplicating rDNA genes only, giving more copies in larger cells: Cavalier-Smith 1985).

Ciliophora also multiplied their cilia into numerous longitudinal rows (kineties), enabling much faster swimming than biciliates, and evolved a mouth supported by specialised oral kineties. The mouth was probably ancestrally apical, not ventral as Cavalier-Smith (2004a) proposed, as infraphylum Ventrata with ventral mouth is clearly derived (Gentekaki et al. 2017); pointlessly renaming Ventrata by a meaningless non-latinised 'Conthreep' unsuitable for a taxon was unwise (Adl et al. 2012). This combination of rapid cell cycle, rapid swimming, and large specialised mouths made ciliates the sharks of the protist world: large fast-swimming predators. Just as there are no photosynthetic sharks, ancestral ciliates rapidly became pure heterotrophs to exploit the new body plan. Many focus on hoovering up vast numbers of tiny bacterial prey as basking sharks swallow plankton. Others evolved raptorial adaptations using their multiciliary and associated cytoskeletal innovations, e.g. *Didinium* that can swallow *Paramecium* larger than themselves, and many evolved trichocysts and more elaborate extrusomes for defence or attack; some became sessile filter feeders and consequently evolved branching multicellurity convergently with some heterokonts of both phyla. Oft-mentioned 'typical cilates' (Lynn and Small 2002) are ventrates, not the ancestral body plan.

Ciliates probably ancestrally retained all loukozoan mt roots with no significant additions, just some reorientations, losses, and changes in mt numbers per band; centrioles were ancestrally paired, as they still are in subphylum Postciliodesmatophora and infraphylum Spirotrichia, but several lineages of Ventrata independently lost one per kinetid. Ciliary transformation must occur, but apparently involves no centriolar rotation. Postciliodesmatophora (e.g. karyorelictid *Geleia* and heterotrich *Eufolliculina*) have fibrous roots on C1 positionally reminiscent of excavate A, I, and C fibres that were likely incorporated early on as an essential part of the characteristic 'postciliary' cytoskeleton of this subphylum, which probably simultaneously lost the posterior singlet root (S). Kinetodesmal fibres by contrast are well developed only in Intramacronucleata and may be an ancestral character for them like their intramacronuclear spindle (contrasting with external spindle in heterotrichs and no macronuclear division in karyorelictids, three divergent consequences of macronuclear origin: Cavalier-Smith 2004a). The single mt nucleated between the centriole pair in some intramacronucleates (e.g. *Colpoda*: Lynn 1988 Fig. 2e; see also Lynn and Small 1981) may be a homologue of the S root of *Colponema* and loukozoan excavates reoriented with C1 when it became parallel to C1 when ciliary multiplication erased the ancestral groove. This singlet is absent in many ciliates (e.g. *Sicophora*, *Eufolliculina* in Lynn 1988 Fig. 2f,g; others in 1991) but was assumed by Moestrup (2000) to be a left anterior R4, which is absent in *Colponema* and loukozoan excavates (and I argue in ancestral chromists and ancestors of each of their four major clades), as he then erroneously thought that ancestral eukaryotes had two anterior mt bands and was

necessarily unaware of the generality of the posterior singlet in loukozoan excavates. A 2-anterior 2-posterior mt root pattern is derived even in Plantae (see below) and almost unknown in Protozoa, whose ancestor arguably had only two mt roots (Fig. 2). Okamoto and Keeling (2014a) followed Moestrup's possible misinterpretation, overlooking that Moestrup cited evidence for *Paramecium* having only anterior root R3 and had overlooked Lynn's drawings of actual kinetids showing that the apparent left anterior root in ciliates is either absent or usually only a singlet and therefore must not automatically be equated to heterokont R4. It is hard to distinguish between the possibility that the intramacronucleate singlet is a reoriented relict excavate singlet (slightly more likely I think) or (as Moestrup assumed) a multiply evolved R4 singlet precursor to the main multi-mt R2 which in species lacking S at least must be formed de novo during centriole maturation.

SD8. Heterokonts also shifted ingestion anteriorly

As noted in the main body of this paper, thrust-reversing tripartite tubular hairs (retronemes) form one or more often two rows on the anterior cilium only of almost all ciliated heterokonts (Cavalier-Smith 1986) that creates a very strong anterior water current towards the cell body bringing prey to the ciliary base where phagocytosis engulfs them, a novelty that radically changed the feeding mode of the ancestral heterokont and moved its ingestion site anteriorly. In loukozoan excavates the feeding current stems mainly from the posterior cilium in the feeding groove and is made more efficient by its ventral vane. *Colponema*'s retention of the vane shows it still remained in the common ancestor of Alveolata and Heterokonta, and was thus almost certainly also present in the ancestor of heterokonts that first evolved retronemes. The focus of ingestion then became the anterior end of the groove, immediately removing the selective advantage for retaining a ciliary vane - so it was lost in the ancestral heterokont, yet was understandably retained by one lineage (Colponemea) in their alveolate sisters that neither evolved retronemes nor any other novel feeding mode.

Setting aside for now the enigmatic early diverging *Platysulcus* for which no protein sequences are known (Shiratori et al. 2015), the primary divergence within heterokonts on multiprotein trees (Derelle et al. 2016) is between phyla Bigyra and Gyrista. Bigyra immediately lost photosynthesis; subphylum Opalozoa mostly still feed phagotrophically using retroneme-directed water currents to the anterior ciliary base, as probably do Eogyrea within subphylum Sagenista. Most Gyrista by contrast retain phototrophy (subphylum Ochrophytina) whether ancestral photophagotrophy as in chrysophytes and other groups that retained the flagellate body form or osmotrophic phototrophy in most lineages that became vegetatively non-ciliate, e.g. diatoms that evolved siliceous frustules or brown algae that evolved cell walls and became multicellular. Ancestral to ochrophytes is subphylum Bigyromonada of about six deep-branching seemingly non-algal lineages, all but two known only as environmental DNA lineages variously called mystery heterokonts (MH, Richards and Bass 2005) or marine stramenopiles (MAST: Massana et al. 2014). MH are reasonably assumed to be mostly phagoheterotrophic flagellates like all MH whose phenotype has been discovered (Cavalier-Smith and Scoble 2013); the deepest branching is MAST-2 but branching order of the the others varies amongst studies implying a rapid early radiation (Aleoshin et al. 2016; Cavalier-Smith and Scoble 2013; Massana et al. 2014; Shiratori et al. 2015) not resolvable until all are cultivated and we get multiprotein trees; only then can we say how often plastids were lost. One bigyromonad lineage evolved vegetative cell walls and osmoheterotrophy,

generating subphylum Pseudofungi (Oomycetes and hyphochytrids) retaining cilia only for zoospores. Another (Developea) remained relatively standard naked zooflagellates with marked ventral groove (Aleoshin et al. 2016), whereas *Pirsonia* with solid posterior ciliary hairs became specialised diatom predators with a posterior pseudopodium to penetrate the rimoportula and phagocytose cytoplasmic pieces (Schnepf and Schweikert 1996/7). Photosynthesis was lost several times within Ochrophytina; though most secondary heterotrophs retain leucoplasts (e.g. paraphysomonad chrysophytes or some pedinellids) as is likely for all, but it is unknown whether the heliozoan-like Actinophryida (derived from raphidophyte algae: Cavalier-Smith and Scoble 2013) or *Picophagus* grouped with photosynthetic *Synchroma* in Picophagea, closely related to chrysophytes, have leucoplasts or not.

Phagotrophy was twice lost in Bigyra, once in each subphylum. First by Labyrinthulea (subphylum Sagenista) when they evolved surface scales and osmotrophic net-like body, making a major marine clade often misclassified as protozoa or fungi (Cavalier-Smith 1997; Anderson and Cavalier-Smith 2012), distinct from their planktonic sisters (Eogyrea) (Derelle et al. 2016). Later within Opalozoa Opalinata became osmotrophic anaerobic parasites that transferred retronemes to the cell body (*Proteromonas*) or lost them. If Rictidia, the earliest branching lineage of opalozoan class Bikosea (Yubuki et al. 2010), is entirely anaerobic (likely), then class Bikosea diverged early into this anaerobic clade and the better known aerobic subclass Bicosidia. All four environmental sequences that group by rDNA with *Rictus* (Cavalier-Smith and Scoble 2013) were from anaerobic marine habitats (e.g. Behnke et al. 2010; Stoeck and Epstein 2003; Takishita et al. 2010). There are now two deep-branching anaerobic clades within Opalozoa: superclass Opalinata and subclass Rictidia, one in each infraphylum.

Cortical alveoli are widespread in Gyrista, notably in bigyromonads *Developayella* and *Develorapax* (Aleoshin et al. 2016; Tong 1995), in two early diverging ochrophyte classes (all Raphidomonadea; most Pinguiophyceae), and with more irregular shape in the early diverging oomycete *Eurychasma* (Sekimoto et al. 2008) - thus in all three subphyla. Pinguiophyte cortical alveoli are most extensive and obvious in *Glossomastix* (O'Kelly 2002) and *Pinguiococcus* (Andersen et al. 2002) and evident in the anterior part of *Phaeomonas* (Honda and Inouye 2002), but apparently lost in the loricate *Polypodochrysis* (Kawachi et al. 2002b) and tiny (1.5-3 μm) aciliate *Pinguiochrysis* (Kawachi et al. 2002a); lorica evolution and cell miniaturization probably made them unnecessary. Diatom and parmalean silica deposition vesicles and chrysomonad scale vesicles perhaps evolved from cortical alveoli. Coupled with presence of cortical alveoli in some Hacrobia (Cavalier-Smith et al. 2015a), and in Glaucophyta among Plantae, and their absence in all excavates, one can reasonably infer that they first evolved in the common ancestor of Chromista and Plantae (the first corticate) and many lineages independently lost them. In ochrophytes it is not surprising that ancestors of Fucistia and Eustigmatophyceae lost cortical alveoli when they independently evolved vegetative walls; as did red algae when walls evolved and Viridiplantae when scales evolved. Unless some MH flagellates have cortical alveoli, they were presumably lost in the ancestral bigyran (even alveolates lost them more than once, e.g. early in ciliate evolution by Karyorelictea). As I have always contended, cortical alveoli are probably a synapomorphy for corticates, not alveolates (Cavalier-Smith and Chao 2003). The protein

alveolin that strengthens them, so far found in alveolates only (Gould et al. 2008), might be a synapomorphy for alveolates - though genomic data for heterokonts with clearcut alveoli to test that are unavailable; alveolins must have evolved from a more distant precursor likely more widespread in corticates. Sporozoa at least form alveoli by fusion of specific Golgi-derived vesicles controlled by Rab11B, a novel alveolate-wide small GTPase paralogue, after apparently attaching to new daughter subpellicular mts (Agop-Nersesian et al. 2010); I am unconvinced that other corticates lack Rab11B.

Platysulcus is particularly important for early heterokont evolution, being sister to all others on an ML rDNA tree (Shiratori et al. 2015); but it could have been placed too deeply - its branch is the longest on the tree, entirely unbroken by relatives, and ML is more prone to long-branch artefacts than site-heterogeneous PhyloBayes CAT. Table 1 conservatively places *Platysulcus* in bigyran subphylum Opalozoa with the only other heterokonts that glide on their posterior cilium (*Caecitellus*, *Incisomonas*, Placididae). rDNA trees imply that all four gliding heterokont groups evolved gliding independently. Evolving gliding typically entails other changes. *Incisomonas* lost the anterior hairy cilium, whereas *Caecitellus* lost its hairs only and changed its beat pattern from undulating to oar-like (thus coming to mimic many Cercozoa; see below). Both lost the ancestral heterokont feeding mode by retronemal water currents, but placidids and *Platysulcus* kept the anterior cilium and its retronemes and were also cytoskeletally more conservative than *Caecitellus*.

SD9. Heterokont cytoskeletal evolution: the bigyran cytopharynx

Unlike in Myzozoa where prior evolution of BB and apical extrusomes in the ancestral chromist facilitated evolving an apical complex, in heterokonts origin of retronemes and consequential novel water currents focused food uptake not at the cell apex but at the base of retroneme-bearing C2. That explains why in bigyran subphylum Opalozoa a new mt-supported cytopharnyx evolved immediately behind the centrioles. A cytopharynx is found in many divergent lineages in class Bikosea, whose taxonomy was also much confused by excessive lumping but is becoming clarified (Cavalier-Smith and Chao 2006; Cavalier-Smith and Scoble 2013). As ultrastructure is unknown for phagotrophic Eogyrea some might have a cytopharynx - if they do, the ancestral bigyran evolved the cytopharynx very early when it lost plastids, and Labyrinthulea secondarily lost the cytopharynx when becoming non-flagellate osmotrophic benthic feeders, retaining cilia only for dispersal. From the relative position of Bicosidia and Placididea on rDNA trees the ancestral feeding pattern of Opalozoa was suspension feeding on bacteria or other small prey drawn to the cell by the retronemal water current.

As Eogyrea are sisters of Labyrinthulea on multiprotein trees (Derelle et al. 2016), phagotrophy at the base of hairy C2 must also be the ancestral state for Bigyra; as the Bigyra/Gyrista split is robustly the deepest amongst heterokonts, this feeding mode was ancestral for all heterokonts. These robust multigene trees show holophyly of Bigyra, Opalozoa, Bikosia, Placidozoa, Sagenista, Gyrista, Ochrophytina, Chrysista, Fucistia, Limnistia, Diatomista, Hypogyrista, Khakista as here delimited, and raphidomonads as sisters of Fucistia. This gives a sound overall phylogenetic framework for interpreting heterokont evolution; multigene data are still needed for Eustigmatophyceae, Chrysomerophyceae, Aurophyceae, bigyromonadads, hyphochytrids, and three placidozoan classes to complete it at class level.

Diversity and phylogeny of Opalozoa are still too imperfectly known to reconstruct cytopharyngeal evolution confidently or say whether the cytopharynx evolved in the ancestral lineage and was lost by those without it (simplest) or evolved later, even polyphyletically.

Three bikosean groups only (Rictida, Pseudodendromonadida, Caecitellidae) have a deep cytopharynx, unlike other heterokonts, aparently supported largely by modified R2 mts. As their cytopharynx is invariably at the opposite end from the cilia of the ventral feeding side of their roughly triangular cells, it may be homologous and a key innovation distinguishing Bikosea from sister group Placidozoa. Contrary to Karpov (2000), Bicoecida have a cytopharynx, located in the same position relative to $R2_o$ as in other Bikosea, but it is shallower. Given rDNA tree topology (Cavalier-Smith and Scoble 2013) it is simplest to assume Borokidae and Cafeteriidae (probably independently) lost a cytopharynx. *Cafeteria* has no cytopharynx, its ingestion site being a temporary cytostome in the same position on the ventral surface relative to cilia and cytoskeleton (Karpov et al. 2001).

Within Bikosea *Rictus* differs from Bicosidia in that its R2 outer mts curve round through 180º to pass back alongside but antiparallel to left posterior R1. In Bicosidia and Placididea R2 curves round to join R1 at the posterior end of the ventral face in a parallel association, exactly as in ancestral Loukozoa. *Rictus* is a derived exception, probably because it evolved raptorial feeding, unusual for Opalozoa. Alone in Bikosea, *Rictus* and *Caecitellus* independently evolved raptorial feeding on individual bacteria associated with surfaces and independently lost retronemes - no longer needed by raptorial feeders. *Caecitellus* finds attached bacteria whilst gliding on surfaces; non-gliding *Rictus* is largely sedentary within microbial films, where its permanent cytostome (larger than in other Bikosea: Yubuki et al. 2010) must help it catch bacteria efficiently; probably the distal part of its $R2_o$ became reflexed to support its large mouth. In Cavalier-Smith and Scoble (2013) trees, as in Park et al. (2006), but unlike Park and Simpson (2010), *Caecitellus* consistently formed a very weakly supported clade with *Halocafeteria*, which also lacks retronemes and has a definite cytostome but no cytopharynx. Possibly retronemes were lost first in their common ancestor and *Caecitellus* became a glider later, but if so why should *Halocafeteria* have lost an ancestral cytopharynx? If instead Anoecida ancestrally had no cytopharynx, like Bicosoecidae that group extremely weakly with them, and *Caecitellus* evolved a cytopharynx independently of *Rictus* and Pseudodendromonadida when it took up gliding, then there would be only two losses of the cytopharynx if it is a synapomorphy for Bikosea. If instead Caecitellidae ancestrally had a cytopharynx, there were independent losses in *Halocafeteria*, Cafeteriidae and *Boroka*. As root R2 is differently arranged distally in *Rictus*, the cytopharynx might have evolved separately in *Rictus*, *Caecitellus*, and Pseudodendromonadida and was not lost at all. Supporting this, all three feed atypically for heterokonts having modified the ancestral feeding mode.

Split R2 is well conserved throughout Opalozoa. My interpretation of bikosid root attachments differs from Karpov et al. (2001) who stated that R2 originates from C2 in *Boroka*, *Caecitellus*, and *Cafeteria*. That is somewhat misleading as it originates where both centrioles meet to the left of C1 base and posterior to (not on) C2. R2 seems to originate from dense fibrillar material that links C1 and C2 bases in all Bikosea and Placididea, not directly from either centriole, just as in the excavate metamonad

Hicanonectes (Park et al. 2009 fig. 19) and *Malawimonas* and Jakobea, yet is conventionally treated as a posterior centriolar root. This nucleation position for R2 is conserved even in hyphochytrid Pseudofungi that lost the posterior cilium: but they retained an extremely short C1 and its fibrous linker to C2 from which a reduced 2-mt R2 stems – by contrast R1 nucleated higher up the centriole (missing in hyphochytrids) has been lost. This emphasizes how conservative the heterokont R2 nucleation site is (except Raphidomonadea: see Cavalier-Smith and Scoble 2013): to retain R2 hyphochytrids had to keep a truncated C1 centriole and fibrous connector. All Opalozoa appear to retain split R2, left R1, and anterior R3. I suggest that the singlet mt (S) of *Boroka,* Bicoecida and *Rictus* is homologous with the loukozoan excavate singlet root; S was apparently lost twice independently in Bigyra - by Anoecida/Pseudodendromonadida (which rDNA trees show weakly together: Cavalier-Smith and Scoble 2013) within Bikosea and by Placidozoa.

The other heterokont phylum Gyrista probably ancestrally retained S but never evolved a cytopharynx (unless early branching uncultured lineages have one) as most focus on phototrophy, saprotrophy/parasitism or axopodial feeding. I suggest that the extra microtubule dorsal to the *Sulcochrysis* right posterior root (R2, but originally labelled R3: Honda et al. 1995 Figs 34, 42, 43) is probably homologous with the excavate and plant singlets discussed above in SD2; sequences are unavailable for *Sulcochrysis* whose tz rings (see SD11) clearly put it in Hypogyrista, and its seemingly primitive groove skeleton makes it likely to be a particularly early lineage diverging close to the base of ochrophytes (Table S1 places *Sulcochrysis* in Sulcophycidae as suggested by Cavalier-Smith and Scoble 2013). Though *Sulcochrysis* singlet a that is in line with R2 mts on the groove side might be supposed to correspond with the excavate singlet because it helps support the groove, its nucleation position does not support that; mt a might represent a reduced outer branch of split R2. The loricate chrysophyte *Epipyxis* reoriented its short posterior cilium forwards, evolving a phagotrophic feeding mode involving ciliary catching of prey brought by the anterior ciliary current, followed by anterior sliding of end mt f of its formerly posterior R2 to make a loop supporting the rim of a transient basket-like structure to surround the prey before phagocytosis (Andersen and Wetherbee 1992; Wetherbee and Andersen 1992). The synurid *Chrysosphaerella* also with anteriorly reoriented cilium involves R2 in feeding. These chrysophyte specialisations are convergent with the sessile feeding method of *Bicosoeca* (Bigyra).

SD10. The bypassing microtubular band in heterokonts

A bypassing 'root' not directly connected to either centriole (therefore not strictly a root: O'Kelly 1989) is located to the cell's right of both centrioles in a few lineages in both ochrophyte infraphyla: in Hypogyrista (infraphylum Diatomista) in the pinguiophyte *Phaeomonas* (1 mt: Honda and Inoue 2002) and pelagophyte *Ankylochrysis* (1 mt: Honda and Inoue 1985); in infraphylum Chrysista in three of four classes of superclass Fucistia - brown algae (Motomura 1989), the aurophyte *Phaeothamnion* (Andersen et al. 1998), the chrysomerophyte *Giraudyopsis* (1 mt: O'Kelly 1989), and in superclass Limnistia in a few ochromonad chrysophytes (O'Kelly 1989). The third chrysist superclass Raphidoistia has a uniquely derived kinetid in which R1 and R4 are lost, a prominent nonmt stiated rhizoplast connects the centrioles to the nucleus, anterior R3 has a unique somewhat MLS-like structure (Vesk and Moestrup 1987), and R2

is associated with a BB-like structure forming a rhizostyle that stretches from the nucleus to well anterior of the centrioles on their right - see references in Cavalier-Smith and Scoble (2013) who first argued that the raphidophyte rhizostyle (which we sometimes accidentally called 'axostyle', an overlooked Freudian slip) was a composite structure of R2 and a separate mt band possibly nucleated at the nucleus and that the latter mts at least may have been ancestral to actinophryid heliozoan axopodial axonemes. The R2 component of the raphidophyte rhizostyle is shorter than the BB-like component (which I now consider homologous with other BBs) and closer to the nuclear envelope where both are present. If raphidophyte BB is indeed homologous with actinophryid axopodia it is presumably nucleated at the nuclear envelope and not the plasma membrane, in which case it would be antiparallel to R2 root, a possibility first noted by Cavalier-Smith and Scoble (2013). Its phylogenetically broad distribution in both infraphyla and every superclass makes it likely that a BB was present in the ancestral ochrophyte but secondarily lost several times.

The oomycete *Lagena* has an identically positioned bypassing band (BB) of four mts, identical in position to the multi-mt BBs present in most Dinozoa (Okamoto and Keeling 2014b), making it likely that the ancestral halvarian had a BB that was lost in Colponemida, ciliates, and most Apicomplexa. Other oomycetes, e.g. *Phytophthora* lack BB mts but have an electron-dense cord-like body in precisely the same position (Barr and Desaulniers 1989) but absent from other protists. This unique oomycete cord is probably a relic of a fibrous adjunct to BB mts persisting after they were lost. Though oomycetes became walled saprotrophs/parasites, their zoospores retain a ventral groove, reduced in width as its feeding role was lost and its fibrous skeleton simplified, I fibres being lost.

Bigyra lack a precisely equivalent BB, but many (e.g. *Rictus*: Yubuki et al. 2010) have an extra singlet microtubule (X) that like BB is right of and parallel to R2; unlike BB it begins not anterior to both centrioles but beside (not on) C1. Conceivably X is a homologue of BB of Gyrista and evolved by slight backward shift of its nucleation point. However, its seems more likely that X is homologous with the extra mt (Em) that lies parallel to the right of posterior R2 of *Sulcochrysis* (Honda et al. 1995) that was suggested above to be homologous with excavate and plant R2-associated singlet mts, and that BB evolved instead from the excavate dorsal fan as proposed in this paper. Finding a heterokont having both a BB and a convincing R2-associated singlet would corroborate that interpretation.

If heterokont BBs are homologous with those of other chromists and all evolved from an excavate dorsal fan as postulated in this paper, then their mts should all have the same polarity. If my explanation of actinophryid axopodial origins (Cavlier-Smith and Scoble 2013) is correct, it implies that all BBs are antiparallel to centriolar root mts, as I show eleswhere is the case for the pellicle mts of Euglenozoa (Cavalier-Smith 2017). Tubulin hook decoration studies (which revealed mt polarity in Euglenozoa) are needed to determine the polarity of chromist BBs and of excavate dorsal fans to test the possibility that BBs evolved from dorsal fans and may have the same polarity as euglenozoan pellicles, which I also suggested evolved (independently) from dorsal fans (Cavalier-Smith 2017).

SD11. Evolution of heterokont ciliary transitional helix (TH) and tz rings

The TH arose at the same time as thrust-reversing retronemes, I suggest to provide firmer ciliary anchorage basally against extra stresses imposed by the greater thrust produced by retronemes. Strictly speaking, the TH is not in the tz as it forms a long sleeve around the CP complex base, analogously to the green plant basal cylinder. If we regard the distal transition plate (dTP) and CP base as together defining the upper limit of the tz compartment the TH is actually in a supra-tz region. Heterokonts differ from their sister alveolates in lacking a prominent dense axosome at the CP proximal end (single in Ciliophora; double in Miozoa, a previously overlooked synapomorphy for them), having instead just a basal density (sometimes scarcely visible). In the heterokont *Synura* the CP complex rotates as it also does in *Paramecium* (Mitchell 2007), so Cavalier-Smith and Oates (2012) suggested that CP rotation might be a synapomorphy for Halvaria. The ciliate axosome nests within a curved dTP, not associated with an outer dense collar present in many eukaryotes (Cavalier-Smith 2014b), the whole resembling a ball and socket joint that would allow free rotation of CP plus axosome, suggesting that ciliate CPs generally rotate as in *Paramecium*. The myzozoan double axosome should also allow CP rotation. The apicomonad cylinder that surrounds the CP base (best seen in *Voromonas* and *Colpodella edax* and *pseudoedax*: Mylnikov et al. 1998, 2000; Mylnikova and Mylnikov 2009) appears unrelated to the heterokont TH or green plant basal cylinder, being slenderer than either; if unlike them attached to CP rather than doublets, as some micrographs suggest, it could serve to make the basal end of the CP complex a snugger fit within the radial inward projections from the doublets as it rotates. Axosome-based CP rotation may be an ultrastructural/physiological synapomorphy for Alveolata; cortical alveoli are not their synapomorphy, being present in glaucophytes, raphidophyte heterokonts, and telonemid cryptists, i.e. a corticate synapomorphy. Alveolates were defined by the combination of cortical alveoli and tubular cristae (Cavalier-Smith 1991); but as raphidophytes have both that definition fell short.

However, I now doubt that heterokont CPs ancestrally rotated, as reexamination of ochrophyte TH diversity (Hibberd 1979) suggests that *Synura* is exceptional in (1) having a long TH with 8-9 gyres and (2) in the dense CP base being separated from the dTP central axosome by a substantial gap. Other ochrophytes have shorter TH (2-5 gyres) or none; in these the dense CP base lies directly over the TP's central density, apparently attached to it. It therefore seems likely that most ochrophytes have basally fixed CP which became unfixed in *Synura* to allow CP rotation and TH then elongated, making a more secure bushing within which CP's base rotates. *Synura* exhibits no obvious cross bridges between CP and TH, which is closer to and probably attached to the doublets, whereas species with no gap between CP and dTP have cross bridges between CP and TH, presumably to better anchor it and prevent rotation. The *Synura* cilia probably both modified their beat pattern in ways favouring CP rotation when the ancestral synurid posterior centriole reoriented to lie parallel to the anterior one.

The peri-CP TH is widely present in ochrophyte infraphylum Chrysista (lost by raphidophytes and brown algal zoids) but completely absent in infraphylum Diatomista. The TH of Chrysista has traditionally been regarded as single helix (Hibberd 1979; Kai et al. 2008) and has been contrasted with that of Pseudofungi and Bigyra which appears double or concertina-like in longitudinal section (LS) (Cavalier-Smith 1997). However, a few chrysists, notably the xanthophyte *Botrydiopsis alpina* short

cilium (Hibberd 1979 Fig. 11), display a concertina-like structure indistinguishable from that of oomycetes (e.g. *Haliphthoros* Beakes et al. 2011 Fig. 17e). Reexamination of numerous micrographs led me to conclude that many chrysists have an underlying zig-zag-like TH structure not fundamentally different from Bigyra and Pseudofungi. The difference between them appears to lie in the relative distribution of secondary dense material. In non-ochrophytes this appears to be relatively even across the zig zag, thus accentuating it, whereas in most chrysists it is concentrated in the mid zone between the zigzag points, thus tending to obscure the underlying zig-zag filamentous structure that is obvious in chrysists only when the dense matrix material is less prominent or more even. I suggest the unusual shortness of the *Botrydiopsis* posterior cilium reduced tz stresses, so less dense matrix was needed.

Recognition of the underlying homology of the filamentous substructure of the chrysist TH with that of Pseudofungi and Bigryra means that the TH was lost by the ancestor of Diatomista and is an ancestral character for Heterokonta in addition to retroneme thrust reversal. Originally Heterokonta were ranked as a phylum (Cavalier-Smith 1981, 1986) and Ochrophytina a subphylum (Cavalier-Smith 1986) but Corliss (1994) raised Heterokonta to subkingdom and Pseudofungi to phylum, which I accepted and also made Ochrophyta a phylum (Cavalier-Smith 1995b) to help gain acceptance from phycologists and mycologists who then resisted pseudofungi and ochrophytes being in one phylum; but a parallel paper reduced Heterokonta to an infrakingdom (Cavalier-Smith 1995a), now reduced to superphylum to accommodate Halvaria as infrakingdom (Table 1). TH unity in all heterokonts (always fundamentally 'double') reduces apparent ultrastructural disparity between ochrophytes and the rest, so Table 1 returns to my first preference for ranking ochrophytes and Pseudofungi as subphyla, thus reducing Gyrista to phylum rank; consequently both heterokont phyla are clades, unlike before. Differences between the three gyrist subphyla are not gains of any complex characters (needed to justify phylum rank) but predominantly losses: plastid losses by Pseudofungi and bigyromonads and phagotrophy loss by Pseudofungi. Pseudofungal gain of a cell wall was the cause of phagotrophy loss, as happened polyphyletically within ochrophytes where wall origin is not given more than class rank.

Instead of a TH, ochrophyte superclass Hypogyrista has two stacked rings in the tz proper immediately below the TP, often misleadingly called a TH. There is no reason to think they are either helical or homologous with the TH so should be called tz rings. They apparently became prominent in the common ancestor of classes Dictyochophyceae and Pinguiophyceae after it diverged from superclass Khakista which have neither TH nor prominent tz rings. I now suggest they are denser elaborations of more tenuous structures visible in this location in other ochrophytes (e.g. chrysophytes: Hibberd 1979 Figs 2, 3, 8, 9). Later studies (e.g. of the pinguiophyte *Phaeomonas*: Honda and Inoue 2002) and reexamination of many micrographs (e.g. Patterson 1985) make it clearer that these structures are two distinct rings, not a helix as formerly called (Andersen et al. 1993); thus contrary to an earlier idea (Cavalier-Smith and Chao 2006), tz rings are unrelated to the TH. Rings are not smoothly continuous, but consist of numerous granules (possibly 18: 9 opposite A tubules, as in the cercozoan hub-lattice discussed in the final section of this paper and SD12, plus 9 intermediate). Two sets of radial spokes (probably 9) connect each ring to the central hub beneath dTP (Honda and Inoue 2002, figs 39, 41). Positionally

hypogyrist rings correspond more to the distal concentric fibres of euglenoids (conceivably independent elaborations of a more widespread but more tenuous tz structure; see SD12). Contrary to previous assumptions, tz rings are not restricted to Hypogyrista. In LS the raphidophyte *Olisthodiscus* shows four distinct spots positionally equivalent to LS views of hypogyrist rings (Hara et al. 1985 Fig. 9); similar structures are probably present in all Raphidophycidae, deep branching chrysists that lack a TH (*Vacuolaria*, *Chattonella*, *Heterosigma*: Heywood 1978; Mignot 1976; Vesk and Moestrup 1987). Prominence of tz rings varies in raphidophytes and Hypogyrista, being most obvious in the pedinellid *Pteridomonas*, but at least as obvious in several raphidophytes as in some hypogyrists (e.g. the pedinellid *Actinomonas* (Larsen 1985) and *Pelagomonas*: Andersen et al. 1993). Virtually all ochrophytes and Pseudofungi have medium density material in the same position that could be evolutionarily related to the more differentiated rings of hypogyrists and raphidophytes. In some where two rings are not obvious one sees densities suggestive of one ring, e.g. in the chrysomerophyte *Giraudyopsis*, which lacks a TH, a cross-section shows an incomplete ring of granules in the outer part of a peripheral lattice surrounding a central hub (O'Kelly and Floyd 1985 fig. 16); the chrysomonad *Spumella* has one sub-plate ring less dense than its 4-gyre TH (Mignot 1977 fig. 5) and thus previously overlooked – it surrounds the central sub-dTP hub. Thus tz rings can be present whether TH is absent or present. Conceivably in a faintly developed form they are ancestral for Gyrista.

SD12. Chromist ciliary transition zone (tz) evolution

Just as heterokont tz evolved a characteristic ciliary TH (strictly supra-tz) different from and more complex than the simple ancestral excavate tz (CP axosome and underlying dense dTP a very short distance above the centriole/tz junction) that was retained by Ciliophora and many but not all Miozoa, so also did Rhizaria. Most distinctive is a hub-lattice structure at the extreme proximal end of the tz just above where centriolar C tubules end, which is probably a synapomorphy for Rhizaria (Cavalier-Smith et al. 2008, 2009). Also distinctive are two other structures with 9-fold symmetry at the distal end of the tz in a broad range of Cercozoa: the nonagonal fibre and distal hub-spoke structure (Cavalier-Smith et al. 2008). Originally I thought the nonagonal fibre was restricted to Cercozoa. but the glaucophyte *Cyanoptyche* has essentially the same structure throughout its elongated tz (Kies 1989) and fuzzy micrographs suggest *Cyanophora* also does (Mignot et al. 1969), so it may be a general glaucophyte feature. Unless convergent, it could be an ancestral corticate character or even present within the dense dTP that is probably an ancestral eukaryotic character (Cavalier-Smith 2014b; then called TP, but here dTP to avoid confusion with transit peptides) as dTP is present in *Tsukubamonas*, all excavates, Percolozoa, and kinetoplastid Euglenozoa.

Previously I suggested that the distal tz hub-spoke structure visible only in Cercozoa without a dTP may be present also in other Cercozoa and likely eukaryotes generally as the skeletal core of the dTP but obscured in most lineages by a dense matrix (Mignot et al. 1969). I now suggest that this may be true of the nonagonal fibre also and both structures may have been present in ancestral eukaryote dTP and differentially lost in rare lineages without a dTP. That conjecture is supported by the unusual tz structure of the biciliate metamonad *Kipferlia* which has a transition plate not in the usual distal position but proximally at the

centriole/tz junction so that the CP begins at that point well below the cell surface (Yubuki et al. 2013), as far as I am aware unlike all other eukaryotes except the discicristate *Percolomonas* (Fenchel and Patterson 1986). Most significantly, an overlooked nonagonal fibre is obvious in both *Kipferlia* cilia surrounding the CP throughout its intracellular region between the centriole and the transitional fibres attaching it to the cell surface (Yubuki et al. 2013 Figs 5A, 7DE). Its unusual conspicuousness results from the absence of the dense component of dTP and unique extension of the nonagonal fibre through the whole thickness of at least two sections. *Malawimonas* (O'Kelly and Nerad 2001 Fig. 13) and the jakobid *Andalucia incarcerata* (Simpson and Patterson 2001 Fig. 3i) have overlooked vertically less extensive nonagonal fibres at the level of the axosome immediately distal to transitional fibres, so they probably extend back at least to the ancestral excavate (sensu Fig. 2). No suitable micrographs are published that could show one in *Tsukubamonas*. The unusual *Goniomonas*-related cryptomonad *Hemiarma* also has a nonagonal fibre whose visibility may reflect the simplication of its tz dense structures compared with other cryptomonads (Shiratori and Ishida 2016).

Discicristates provide a comparative test of this hypothesis as their tz is short with a dTP in Percolozoa and euglenozoan subphylum Glycomonada; but euglenoids and Postgaardea (their likely sisters: Cavalier-Smith 2016), have a remarkably elongate tz (often 1-2 μm) whose structures may be hypertrophied axially and thus easier to see as each can fully occupy one or more ultrathin sections. Euglenoids have analogous 9-fold tz structures to the nonagonal fibre; their struts in *Entosiphon* sometimes appear to contact the B-tubule side of the A/B junction (not the A side as in neokaryote and probably *Andalucia* nonagonal fibres), and are much longer and finer than in excavate-derived nonagonal fibres (Solomon et al. 1987); other *Entosiphon* micrographs however show two radial connectors between the possible nonagonal fibre and doublets, one to A and one to B tubules (Brugerolle 1992 Fig. 8). That these may be variant structures at different levels is suggested by the long tz of *Calkinsia* (belonging to Postgaardea) where distally a hub-spoke structure nested within a nonagonal fibre is attached by struts to A tubules as in excavates and Cercozoa (Yubuki et al. 2009 Fig 6 D,L); somewhat more proximally *Calkinsia* has a slightly different hub-spoke structure and nonagonal fibre with 18 relatively short struts attached to doublets separately at A and B tubules.

I postulate that the exceptionally long tz of the euglenoid/postgaardiid clade repeats many times along its axis distal hub-spoke/nonagonal fibres that may be present in most eukaryotes only within dTP but obscured by its dense material. Absence of dTP in euglenoids/postgaardiids is probably illusory; instead of being lost its core fibrillar compoents are axially multiplied over at least 0.5 μm so that distinct distal and proximal components can be visually differentiated in thin sections. I postulate that the distal hub-spoke/nonagonal fibre structure of *Calkinsia* is homologous with that of Cercozoa and is an ancestral eukaryote character; the more proximal structure with two struts might be a variant specific to euglenoids/postgaardiids, but might instead represent a more general structure so far overlooked in short-tz organisms. Many eukaryote-wide cytoskeletal structures were first discovered in cells where they were hypertrophied, e.g. actin in muscles; assemblin and centrin in chlorophyte fibrous roots. Possibly the euglenoid tz offers such an opportunity for ultrastructurally dissecting tz proteins. Unfortunately trypanosome tomography did not include dTP (Lacomble et al. 2009). Cavalier-Smith and Oates (2012) previously suggested that there may be a more conserved underlying structure for tz than is obvious from their huge ultrastructural diversity that is phylogenetically so informative (Karpov

and Fokin 1995). There are variations in dTP appearance across chromists; some e.g. *Spongomonas* (Hibberd 1976) are of even thickness; others have a prominent central boss in the position where the cercozoan hub lies (e.g the hacrobian *Palpitomonas*: Yabuki et al. 2010); these differences may simply reflect differences in radial distribution of a dense matrix surrounding the filamentous core that could have basically the same structure in all eukaryotes.

I now suggest that protein SAS-6L (related to the globular domain of the SAS-6 centriolar hub-spoke protein) that is is present in the dTP region of *Trypanosoma* and the preconoid rings of *Toxoplasma* and *Plasmodium* (de Leon et al. 2013) probably forms key filamentous parts of either the distal tz hub-spoke structure (most likely) and/or the nonagonal fibre, and this function may have evolved even before centriolar triplets and ciliary CP mts (Cavalier-Smith 2014b). I also postulate that the distinct hub-spoke of the rhizarian hub-lattice might be constructed by a third, possibly Rhizaria-specific paralogue of SAS-6. The main evolutionary forces responsible for tz evolutionary diversification may have been (1) separation of axosome and dTP to allow CP rotation (Mitchell 2007), probably independently in alveolates and synurid heterokonts - and Viridiplantae for which their unique supra-tz dense cylinder (Cavalier-Smith 1974; Manton 1965) may I suggest provide a mechanically important bushing; and (2) tz elongation which was extreme in euglenoids when the long ciliary reservoir evolved, causing dTP loss, but less so in Glaucophyta (presumably because their anterior ciliary pocket is shallower) but also associated with dense dTP loss. The ancestral eukaryote tz was probably short and simple as in all Eozoa except the clearly derived euglenoid/postgaardean clade. It was also probably short with simple dense dTP and no prominent axosome in the ancestral chromist.

Several chromists independently elongated tz (much less so than euglenoids) and thus evolved distinct proximal and dTPs, the second proximal plate just above the centriole/tz junction. That is especially characteristic of many Hacrobia (Cavalier-Smith et al. 2015a) but independent harosan examples exist in Ciliophora (some even with three or four: Karpov and Fokin 1995), and Cercozoa (e.g. *Allapsa*: Cavalier-Smith and Oates 2012) and some Dinozoa have uniquely specialised structures, e.g. Myzodinea (the conical dTP variant discussed above) and *Perkinsus* (Coss et al. 2001). When a dense proximal transition plate is present it would obscure the region of the cercozoan hub-lattice.

Supplementary References (exclude references cited both in the printed paper and in the electronic supplement)

Adl SM et al. (2012) The revised classification of eukaryotes. J Eukaryot Microbiol 59:429-493 doi:10.1111/j.1550-7408.2012.00644.x

Agop-Nersesian C, Egarter S, Langsley G, Foth BJ, Ferguson DJ, Meissner M (2010) Biogenesis of the inner membrane complex is dependent on vesicular transport by the alveolate specific GTPase Rab11B. PLoS Pathog 6:e1001029 doi:10.1371/journal.ppat.1001029

Aléxéieff A (1918) Sur un flagellé coprozoite *Alphamonas coprocola* n. g., n. sp. Arch Zool exp et gén 57:1-11

Aléxéieff A (1924) Notes sur quelques protistes coprocoles. Arch Protistenk 50:27-49

Andersen RA (1987) Synurophyceae classis nov., a new class of algae. Am J Bot 74:337-353

Andersen RA, Potter D, Bailey JC (2002) *Pinguiococcus pyrenoidosus* gen. et sp. nov. (Pinguiophyceae), a new marine coccoid alga. Phycol Res 50:57-65

Andersen RA, Potter D, Bidigare RR, Latasa M, Rowan K, O'Kelly C (1998) Characterization and phylogenetic position of the enigmatic golden alga. *Phaeothamnion confervicola*: ultrastructure, pigment composition and partial SSU rRNA sequence. J Phycol 34:286-298

Andersen RA, Saunders GW, Paskind MP, Sexton J (1993) Ultrastructure and 18S rRNA gene sequence for *Pelagomonas calceolata* gen. et sp. nov. and the description of a new algal class, the Pelagophyceae *classis nov*. J Phycol 29:701-715

Andersen RA, Wetherbee R (1992) Microtubules of the flagellar aparatus are active in prey capture of the chrysophycean alga *Epipyxis pulchra.* Protoplasma 166:1-7

Barr D, J.S, Desaulniers NL (1989) The flagellar apparatus of the Oomycetes and Hypochytridiomycetes. In: The chromophyte algae: Problems and perspectives. Clarendon Press, Oxford, pp 343-355

Beakes GW, Glockling SL, Sekimoto S (2012) The evolutionary phylogeny of the oomycete "fungi". Protoplasma 249:3-19

Behnke A, Barger KJ, Bunge J, Stoeck T (2010) Spatio-temporal variations in protistan communities along an O/HS gradient in the anoxic Framvaren Fjord (Norway). FEMS Microbiol Ecol 72:89-102 doi:10.1111/j.1574-6941.2010.00836.x

Belgio E et al. (2017) High photochemical trapping efficiency in Photosystem I from the red clade algae *Chromera velia* and *Phaeodactylum tricornutum.* Biochim Biophys Acta 1858:56-63 doi:10.1016/j.bbabio.2016.10.002

Bina D et al. (2014) Novel type of red-shifted chlorophyll a antenna complex from *Chromera velia*: II. Biochemistry and spectroscopy. Biochim Biophys Acta 1837:802-810 doi:10.1016/j.bbabio.2014.01.011

Brugerolle G, (2002b) *Cryptophagus subtilis*: a new parasite of cryptophytes affiliated with the Perkinsozoa lineage. Eur J Protistol 37:379-390

Brugerolle G, Mignot J-P (1979) Observations sur le cycle l'ultrastructure et la position sytématique de *Spiromonas perforans* (*Bodo perforans* Hollande 1938). Flagellé parasite de *Chilomonas paramecium*: ses relations avec les dinoflagellés et sporozoaires. Protistologica 15:183-196

Cavalier-Smith T (1974) Basal body and flagellar development during the vegetative cell cycle and the sexual cycle of *Chlamydomonas reinhardii.* J Cell Sci 16:529-556

Cavalier-Smith T (1985) Cell volume and the evolution of genome size. In: Cavalier-Smith T (ed) The evolution of genome size. Wiley, Chichester, pp 105-184

Cavalier-Smith T (1987d) Glaucophyceae and the origin of plants. Evol Trends Plants 2:75-78.

Cavalier-Smith T (1993b) Evolution of the eukaryotic genome. In: Broda P, Oliver SG, Sims P (eds) The Eukaryotic Genome. Cambridge University Press, pp 333-385

Cavalier-Smith T (1995b) Zooflagellate phylogeny and classification. Cytology (St. Petersburg) 37:1010-1029

Cavalier-Smith T (1997) Sagenista and Bigyra, two phyla of heterotrophic heterokont chromists. Archiv Protistenk 148:253-267

Cavalier-Smith T (2016) Higher classification and phylogeny of Euglenozoa. Eur J Protistol 56:250-276 doi:10.1016/j.ejop.2016.09.003

Cavalier-Smith T (2017) Euglenoid pellicle morphogenesis and evolution in light of comparative ultrastructure and trypanosomatid biology: semi-conservative microtubule-strip duplication, strip shaping and transformation. Under review.

Cienkowski L (1865) Beiträge zur Kenntnis der Monaden. Archiv micr Anat 1:203-232 + Plates 212-214

Clayton MN (1990) Phylum Phaeophyta. In: Margulis L, Corliss JO, Melkonian M, Chapman DJ (eds) Handbook of Protoctista. Jones & Bartlett, Boston, pp 698-714

Corliss JO (1994) An interim utilitarian ("user friendly") hierarchical classification and characterisation of the protists. Acta Protozool 33:1-51

Coss CA, Robledo JA, Vasta GR (2001) Fine structure of clonally propagated in vitro life stages of a *Perkinsus* sp. isolated from the Baltic clam *Macoma balthica.* J Eukaryot Microbiol 48:38-51

de Leon JC et al. (2013) A SAS-6-like protein suggests that the *Toxoplasma* conoid complex evolved from flagellar components. Eukaryot Cell 12:1009-1019 doi:10.1128/EC.00096-13

Dubremetz JF (1975) La genèse des mérozoites chez la Coccidie *Eimeria necatrix*. Etude ultrastructurale. J Protozool 22:71-84

Dujardin F (1841) Histoire naturelle des zoophytes infusoires. Roret, Paris

Füssy Z, Masařová P, Kručinska J, Esson HJ, Oborník M (2016) Budding of the alveolate alga *Vitrella brassicaformis* resembles sexual and asexual processes in apicomplexan parasites. Protist 168:80-91 doi:10.1016/j.protis.2016.12.001

Gornik SG, Ford KL, Mulhern TD, Bacic A, McFadden GI, Waller RF (2012) Loss of nucleosomal DNA condensation coincides with appearance of a novel nuclear protein in dinoflagellates. Curr Biol 22:2303-2312 doi:10.1016/j.cub.2012.10.036

Gould SB, Tham WH, Cowman AF, McFadden GI, Waller RF (2008) Alveolins, a new family of cortical proteins that define the protist infrakingdom Alveolata. Mol Biol Evol 25:1219-1230

Guichard P et al. (2012) Cartwheel architecture of *Trichonympha* basal body. Science 337:553 doi:10.1126/science.1222789

Guichard P, Gonczy P (2016) Basal body structure in *Trichonympha.* Cilia 5:9 doi:10.1186/s13630-016-0031-7

Hara Y, Inoue I, Chihara M (1985) Morphology and ultrastructure of *Olisthodiscus luteus* (Raphidophyceae) with special reference to the taxonomy. Bot Mag Tokyo 98:251-262

Harper JD, Thuet J, Lechtreck KF, Hardham AR (2009) Proteins related to green algal striated fiber assemblin are present in stramenopiles and alveolates. Protoplasma 236:97-101 doi:10.1007/s00709-009-0041-z

Heidelberg KB, Nelson WC, Holm JB, Eisenkolb N, Andrade K, Emerson JB (2013) Characterization of eukaryotic microbial diversity in hypersaline Lake Tyrrell, Australia. Frontiers in microbiology 4:115 doi:10.3389/fmicb.2013.00115

Hess S, Melkonian M (2013) The mystery of clade X: *Orciraptor* gen. nov. and *Viridiraptor* gen. nov. are highly specialised, algivorous amoeboflagellates (Glissomonadida, Cercozoa). Protist 164:706-747 doi:10.1016/j.protis.2013.07.003

Heywood P (1978) Ultrastructure of mitosis in the chloromonadiophycean alga *Vacuolaria virescens*. J Cell Sci 31:37-51

Hibberd DJ (1976) The fine structure of the colonial colorless flagellates *Rhipidodendron splendidum* Stein and *Spongomonas uvella* Stein with special reference to the flagellar apparatus. J Protozool 23:374-385

Hibberd DJ (1979) The structure and phylogenetic significance of the flagellar transition region in the chlorophyll c-containing algae. BioSystems 11:243-267

Hilbert M et al. (2016) SAS-6 engineering reveals interdependence between cartwheel and microtubules in determining centriole architecture. Nat Cell Biol 18:393-403 doi:10.1038/ncb3329

Hirono M (2014) Cartwheel assembly. Philos Trans R Soc Biol Sci 369 doi:10.1098/rstb.2013.0458

Höhfeld I, Melkonian M Ultrastructure of the flagellar apparatus of *Noctiluca miliaris* Suriray swarmers (Dinophyceae). Phycologia 34:508-513

Honda D, Inoue I (1985) Ultrastructure and reconstruction of the flagellar apparatus architecture in *Ankylochrysis lutea* (Chrysophyceae, Sarcinochrysidales). 34:215-227

Honda D, Inoue I (2002) Ultrastructure and taxonomy of a marine photosynthetic stramenopile *Phaeomonas parva* gen. et sp. nov. (Pinguiophyceae) with emphasis on the flagellar apparatus architecture. Phycol Res 50:75-89

Honda D, Kawachi M, Inoue I (1995) *Sulcochrysis biplastida* gen. et sp. nov.: cell structure and absolute configuration of the flagellar apparatus of an enigmatic chromophyte alga. Phycol Res 43:1-16

Howe AT, Bass D, Vickerman K, Chao EE, Cavalier-Smith T (2009) Phylogeny, taxonomy, and astounding genetic diversity of Glissomonadida ord. nov., the dominant gliding zooflagellates in soil (Protozoa: Cercozoa). Protist 160:159-189

Hu K, Roos DS, Murray JM (2002) A novel polymer of tubulin forms the conoid of *Toxoplasma gondii.* J Cell Biol 156:1039-1050

Janouškovec J, Tikhonenkov DV, Burki F, Howe AT, Kolisko M, Mylnikov AP, Keeling PJ (2015) Factors mediating plastid dependency and the origins of parasitism in apicomplexans and their close relatives. Proc Natl Acad Sci USA 112:10200-10207 doi:10.1073/pnas.1423790112

Kai A, Yoshii Y, Nakayama T, Inouye I (2008) Aurearenophyceae classis nova, a new class of Heterokontophyta based on a new marine unicellular alga *Aurearena cruciata* gen. et sp. nov. inhabiting sandy beaches. Protist 159:435-457 doi:10.1016/j.protis.2007.12.003

Karpov SA (2000) Ultrastructure of the aloricate bicosoecid *Pseudobodo tremulans*, with revision of the order Bicosoecida. Protistology 1:101-109

Karpov SA, Fokin SA (1995) The structural diversity of the flagellar transition zone in heterotrophic flagellates and other protists. Cytology 37:1038-1052

Karpov SA, Sogin M, Silberman JD (2001) Rootlet homology, taxonomy, and phylogeny of bicosoecids based on 18S rRNA gene sequences. Protistology 2:34-47

Kawachi M, Atsumi N, Ikemoto H, Miyachi S (2002a) *Pinguiochrysis pyriformis* gen. et sp. nov. (Pinguiophyceae), a new picoplanktonic alga isolated from the Pacific Ocean. Phycol Res 50:49–56

Kawachi M, Noël M-H, Andersen RA (2002b) Reexamination of the marine 'chrysophyte' *Polypodochrysis teissieri* (Pinguiophyceae). Phycol Res 50:91–100

Kereïche S et al. (2008) Association of chlorophyll a/c(2) complexes to photosystem I and photosystem II in the cryptophyte *Rhodomonas* CS24. Biochim Biophys Acta 1777:1122-1128 doi:10.1016/j.bbabio.2008.04.045

Kies L (1976) Untersuchungen zur Feinstruktur und taxonomischen Einordnung von *Gloeochaete wittrockiana*, einer apoplastidalen capsalen Alge mit blaugrünen Endosymbionten (Cyanellen). Protoplasma 87:419-446

Kies L (1979) Zur systematischen Einordnung yon *Cyanophora paradoxa*, *Gloeochaete wittrockiana* und *Glaucocystis nostochinearum.* Ber Deutsch Bot Ges 92:445-454

Kies L (1989) Ultrastructure of *Cyanoptyche gloeocystis* f. *dispersa* (*Glaucocystophyceae*). Pl Syst Evol 164:65-73

Kies L, Kremer BP (1990) Phylum Glaucocystophyta. In: Margulis L, Corliss JO, Melkonian M, Chapman DJ (Eds), Handbook of Protoctista. Jones and Bartlett, Boston, pp. 152-166

Kuvardina ON, Leander BS, Aleshin VV, Mylnikov AP, Keeling PJ, Simdyanov TG (2002) The phylogeny of colpodellids (Alveolata) using small subunit rRNA gene sequences suggests they are the free-living sister group to apicomplexans. J Eukaryot Microbiol 49:498-504

Lacomble S, Vaughan S, Gadelha C, Morphew MK, Shaw MK, McIntosh JR, Gull K (2009) Three-dimensional cellular architecture of the flagellar pocket and associated cytoskeleton in trypanosomes revealed by electron microscope tomography. J Cell Sci 122:1081-1090 doi:10.1242/jcs.045740

Larsen J (1985) Ultrastructure and taxonomy of *Actinomonas pusilla*, a heterotrophic member of the Pedinellales (Chrysophyceae). Brit Phycol J 20:341-355

Leander BS, Kuvardina ON, Aleshin VV, Mylnikov AP, Keeling PJ (2003) Molecular phylogeny and surface morphology of *Colpodella edax* (Alveolata): insights into the phagotrophic ancestry of apicomplexans. J Euk Microbiol 50:334-340

Leliaert F et al. (2016) Chloroplast phylogenomic analyses reveal the deepest-branching lineage of the Chlorophyta, Palmophyllophyceae class. nov. Sci Rep 6:25367 doi:10.1038/srep25367

Llansola-Portoles MJ, Uragami C, Pascal AA, Bina D, Litvin R, Robert B (2016) Pigment structure in the FCP-like light-harvesting complex from *Chromera velia.* Biochim Biophys Acta 1857:1759-1765 doi:10.1016/j.bbabio.2016.08.006

Lynn D (1988) Cytoterminology of cortical components of ciliates: somatic and oral kinetids. BioSystems 21:299-307

Lynn D, Small EB (1981) Protist kinetids: structural conservatism, kinetid structure, and ancestral states. BioSystems 14:377-385

Manton I (1965) Some phyletic implications of flagellar structure in plants. Advances in Botanical Research 2:1-34

Marinov GK, Lynch M (2015) Diversity and divergence of dinoflagellate histone proteins. G3 (Bethesda) 6:397-422 doi:10.1534/g3.115.023275

Massana R, del Campo J, Sieracki ME, Audic S, Logares R (2014) Exploring the uncultured microeukaryote majority in the oceans: reevaluation of ribogroups within stramenopiles. ISME J 8:854-866 doi:10.1038/ismej.2013.204

Melkonian M (1989) Flagellar apparatus ultrastructure in *Mesostigma viride* (Prasinophyceae). Plant System Evol 164:93-122

Mignot J-P (1976) Compléments a l'étude des Chloromonadines. Ultrastructure de *Chattonella subsalsa* Biecheler flagellé d'eau saumâtre. Protistologica 12:279-293

Mignot J-P (1977) Étude ultrastructurale d'un flagellé du genre *Spumella* Cienk. (= *Heterochromonas* Pascher = Monas O.F. Miiller), chrysomonadine leucoplastidiée. Protistologica 13:219-231

Mignot J-P, Joyon L, Pringsheim EG (1969) Quelques particularités structurales de *Cyanophora paradoxa* Korsch., protozoaire flagellé. J Protozool 16:138-145

Mikhailov KV, Tikhonenkov DV, Janouškovec J, Diakin AY, Ofitserov MV, Mylnikov AP, Aleoshin VV (2015) Primary structure of 28S rRNA gene confirms monophyly of free-living heterotrophic and phototrophic apicomplexans (Alveolata). Biochemistry (Mosc) 80:1492-1499 doi:10.1134/S0006297915110115

Mizoguchi T, Kimura Y, Yoshitomi T, Tamiaki H (2011) The stereochemistry of chlorophyll-c(3) from the haptophyte *Emiliania huxleyi*: the (13(2)R)-enantiomers of chlorophylls-c are exclusively selected as the photosynthetically active pigments in chromophyte algae. Biochim Biophys Acta 1807:1467-1473 doi:10.1016/j.bbabio.2011.07.008

Moestrup Ø (1978) On the phylogenetic validity of the flagellar apparatus in green algae and other chlorophyll *a* and *b* containing plants. BioSystems 10:117-144

Motomura T (1989) Ultrastructural study of sperm in *Laminaria angustata* (Laminariales, Phaeophyta), especially on the flagellar apparatus. Jap J Phycol 37:105-116

Mylnikov AP (2000) The new marine carnivorous flagellate *Colpodella pontica* (Colpodellida, Protozoa). Zoolog Zhur 79:261-266 (In Russian)

Mylnikov AP (2009) Ultrastructure and phylogeny of colpodellids. Biol Bull 36:582-590

Mylnikov AP, Mylnikov AA (2007) *Colpodella pseudoedax* sp. n. (Protista, Colpodellida) — a new alveolate carnivorous flagellate. Vestnik zool 41:123-129 (In Russian)

Mylnikov AP, Mylnikova ZM, Tsvetkov AH (1998) The fine structure of carnivorous flagellate *Colpodella edax.* Biol Vnutr Vod 3:55-62 (In Russian)

Mylnikov AP, Mylnikova ZM, Tsvetkov AI (2000) Fine structure of a predatory flagellate *Colpodella* sp. Biol Vnutr vod 1:29-36

Mylnikova ZM, Mylnikov AP (2009) The morphology of predatory flagellate *Colpodella pseudoedax* Mylnikov et Mylnikov, 2007 (Colpodellida, Alveolata). Inland Water Biol 2:199-204

Okamoto N, Keeling PJ (2014a) The 3D structure of the apical complex and association with the flagellar apparatus revealed by serial TEM tomography in *Psammosa pacifica*, a distant relative of the Apicomplexa. PLoS One 9, e84653.

O'Kelly CJ (1989) The evolutionary origin of the brown algae: information from studies of motile cell ultrastructure. In: Green JC, Leadbeater BSC, Diver WL (eds) The chromophyte algae: problems and perspectives. Clarendon Press, Oxford, pp 255-278

O'Kelly CJ (1992) Flagellar apparatus architecture and the phylogeny of "green" algae: chlorophytes, euglenoids, glaucophytes. In: Menzel D (ed) Cytoskeleton of the algae. CRC Press, Boca Raton, Fla., pp 315-345

O'Kelly CJ (2002) *Glossomastix chrysoplasta* n. gen., n. sp. (Pinguiophyceae), a new coccoidal, colony-forming golden alga from southern Australia. Phycol Res 50:67-74

O'Kelly CJ, Floyd GL (1985) Absolute configuration analysis of the flagellar apparatus in *Giraudyopsis stellifer* (Chrysophyceae, Sarcinochrysidales) zoospores and its significance in the evolution of the Phaeophyceae. Phycologia 24:283-274

Pan H, Šlapeta J, Carter D, Chen M (2012) Phylogenetic analysis of the light-harvesting system in *Chromera velia.* Photosynth Res 111:19-28 doi:10.1007/s11120-011-9710-9

Park JS, Cho BC, B. SAG (2006) *Halocafeteria seosinensis* gen. et sp. nov. (Bicosoecida), a halophilic bacterivorous nanoflagellate isolated from a solar saltern. Extremophiles 10:493-504

Park JS, Kolisco M, Heiss AA, Simpson AGB (2009) Light microscopic observations, ultrastructure, and molecular phylogeny of *Hicanonectes teleskopos* n. g., n. sp., a deep-branching relative of diplomonads. J Euk Microbiol 56:373-384

Park JS, Simpson AG (2010) Characterization of halotolerant Bicosoecida and Placididea (Stramenopila) that are distinct from marine forms, and the phylogenetic pattern of salinity preference in heterotrophic stramenopiles. Environ Microbiol 12:1173-1184 doi:10.1111/j.1462-2920.2010.02158.x

Patterson DJ, Zölffel M (1991) Heterotrophic flagellates of uncertain taxonomic position. In: Patterson DJ, Larsen J (eds) The biology of free-living heterotrophic flagellates. Clarendon Press, Oxford, pp 427-476

Portman N, Šlapeta J (2014) The flagellar contribution to the apical complex: a new tool for the eukaryotic Swiss Army knife? Trends in parasitology 30:58-64 doi:10.1016/j.pt.2013.12.006

Richards TA, Bass D (2005) Molecular screening of free-living microbial eukaryotes: diversity and distribution using a meta-analysis. Curr Opin Microbiol 8:240-252

Roberts KR, Farmer MA, Schneider RM, Lemoine J (1988) The microtubular cytoskeleton of *Amphidinium rhynchocephalum* (Dinophyceae). J Phycol 24:544-553

Saville Kent W (1880-1882) A manual of the Infusoria. 3 vols Bogue, London.

Schnepf E, Deichgraber G (1984) ``Myzocytosis", a kind of endocytosis with implications to compartmentation in endosymbiosis. Naturwissenschaften 71:218-219

Schnepf E, Schweikert M (1996/7) *Pirsonia*, phagotrophic nanoflagellates incertae sedis, feeding on marine diatoms: attachment, fine structure and taxonomy. Arch Protistenk 147:361-371

Sekimoto S, Beakes GW, Gachon CM, Muller DG, Kupper FC, Honda D (2008) The development, ultrastructural cytology, and molecular phylogeny of the basal oomycete *Eurychasma dicksonii*, infecting the filamentous phaeophyte algae *Ectocarpus siliculosus* and *Pylaiella littoralis.* Protist 159:299-318 doi:10.1016/j.protis.2007.11.004

Ševčiková T et al. (2015) Updating algal evolutionary relationships through plastid genome sequencing: did alveolate plastids emerge through endosymbiosis of an ochrophyte? Sci Rep 5:10134 doi:10.1038/srep10134

Shiratori T, Ishida KI (2016) A new heterotrophic cryptomonad: *Hemiarma marina* n. g., n. sp. J Eukaryot Microbiol 63:804-812 doi:10.1111/jeu.12327

Sierra R et al. (2016) Evolutionary origins of rhizarian parasites. Mol Biol Evol 33:980-983 doi:10.1093/molbev/msv340

Simpson AGB, Patterson DJ (1996) Ultrastructure and identification of the predatory flagellate *Colpodella pugnax* Cienkowski (Apicomplexa) with a description of *Colpodella turpis* n. sp. and a review of the genus. Syst Parasitol 33: 187-198

Solomon JA, Walne PL, Kivic PA (1987) *Entosiphon sulcatum* (Euglenophyceae): flagellar roots of the basal body complex and reservoir region. J Phycol 23:85-98

Stoeck T, Epstein S (2003) Novel eukaryotic lineages inferred from small-subunit rRNA analyses of oxygen-depleted marine environments. Appl Environ Microbiol 69:2657-2663

Suda S (2003) Light microscopy and electron microscopy of *Nephroselmis spinosa* sp. nov. (Prasinophyceae, Chlorophyta). J Phycol 39:590-599

Takishita K, Kakizoe N, Yoshida T, Maruyama T (2010) Molecular evidence that phylogenetically diverged ciliates are active in microbial mats of deep-sea cold-seep sediment. J Eukaryot Microbiol 57:76-86 doi:10.1111/j.1550-7408.2009.00457.x

Tichy J et al. (2013) Light harvesting complexes of *Chromera velia*, photosynthetic relative of apicomplexan parasites. Biochim Biophys Acta 1827:723-729 doi:10.1016/j.bbabio.2013.02.002

Tong SM (1995) *Developayella elegans* nov. gen., spec., a new type of heterotrophic flagellate from marine plankton. Europ J Protistol 31:24-31

Vesk M, Moestrup Ø (1987) The flagellar root sytem in *Heterosigma akashiwo* (*Raphidophyceae*). Protoplasma 137:15-29

Wetherbee R, Andersen RA (1992) Flagella of a chrysophycean alga play an active role in prey capture and selection: Direct observations on *Epipyxis pulchra* using image enhanced video microscopy. Protoplasma 166:1-7

Wilcox LW (1989) Multilayered structures (MLSs) in two dinoflagellates, *Katodinium campylops* and *Woloszynskia pascheri.* J Phycol 25:785–789

Yabuki A, Inagaki Y, Ishida K (2010) *Palpitomonas bilix* gen. et sp. nov.: a novel deep-branching heterotroph possibly related to Archaeplastida or Hacrobia. Protist 161: 523-538

Yubuki N, Edgcomb VP, Bernhard JM, Leander BS (2009) Ultrastructure and molecular phylogeny of *Calkinsia aureus*: cellular identity of a novel clade of deep-sea euglenozoans with epibiotic bacteria. BMC Microbiol 9:16

Yubuki N, Leander BS, Silberman JD (2010) Ultrastructure and molecular phylogenetic position of a novel phagotrophic stramenopile from low oxygen environments: *Rictus lutensis* gen. et sp. nov. (Bicosoecida, incertae sedis). Protist 161:264-278 doi:10.1016/j.protis.2009.10.004
